# Supplementary material for: Sexual Dysfunction in Patients with Chronic Non-Genital Physical Disease: An Umbrella Review
Source: Int J Environ Res Public Health. 2025 Jan 24;22(2):157. doi: 10.3390/ijerph22020157 (PMC11855788; doi:10.3390/ijerph22020157)
Supplement: Supplementary file 1 [file ijerph-22-00157-s001.zip › reasonForExclusion.pdf]

Of the 77 items, we excluded 42 items that did not meet our criteria for the following reasons:

9 Poster session:

- 793-805- 854- 859 Chanloung, W., Kasitanon, N., Wichainun, R., & Louthrenoo, W. (2021). Thrombotic risk assessment in patients with systemic lupus erythematosus: Validation of the adjusted-Global Antiphospholipid Syndrome Score (aGAPSS) in Thai patients. *International Journal of Rheumatic Diseases*, *24*(12), 1510-1519.
- 853- Samaraweera, A. P. R., Clarke, M., Falah, Y., Pitiot, A., Tench, C. R., Dineen, R. A., ... & Evangelou, N. (2015, September). STAR-MS Study: Single Test to ARrive at MS diagnosis. A prospective, investigator blinded pilot study, assessing the accuracy of a single 3 Tesla MRI scan in predicting multiple sclerosis in cases of diagnostic uncertainty: Study protocol. In *MULTIPLE SCLEROSIS JOURNAL* (Vol. 21, pp. 369-370). 1 OLIVERS YARD, 55 CITY ROAD, LONDON EC1Y 1SP, ENGLAND: SAGE PUBLICATIONS LTD.
- 859-Jaruvongvanich, V., Vantanasiri, K., & Ungprasert, P. (2019). Sa1795–Sexual Function in Patients with Inflammatory Bowel Disease: A Systematic Review and Meta-Analysis. *Gastroenterology*, *156*(6), S-405.
- 878- Varney, P., Brown, M., Guo, W., Usmani, H., Marquez, J., Ayasse, M., ... & Haughton, A. (2021). 26673 A systematic review and meta-analysis of sexual dysfunction in patients with hidradenitis suppurativa. *Journal of the American Academy of Dermatology*, *85*(3), AB23.
- 1425- Ramalingam, K., Vincent, L., & Monga, A. (2014). OBESTITY, BARIATRIC SURGERY & SEXUAL FUNCTION: A SYSTEMATIC REVIEW. *INTERNATIONAL UROGYNECOLOGY JOURNAL*, *25*, S126-S127.
- 1692- Park, K. (2013, June). DIABETES MELLITUS AND FEMALE SEXUAL DYSFUNCTION. In *JOURNAL OF SEXUAL MEDICINE* (Vol. 10, pp. 194-194). 111 RIVER ST, HOBOKEN 07030-5774, NJ USA: WILEY-BLACKWELL.

5 Doublon :

- 718- Zhang, J., Wei, S., Zeng, Q., Wu, X., & Gan, H. (2021). Prevalence and risk factors of sexual dysfunction in patients with inflammatory bowel disease: Systematic review and meta-analysis. *International Journal of Colorectal Disease*, *36*(9), 2027-2038.
- 742-Pyrgidis, N., Mykoniatis, I., Sokolakis, I., Tishukov, M., Kalyvianakis, D., Sountoulides, P., ... & Hatzichristou, D. (2022). Sexual dysfunction in patients with end-stage renal disease: A systematic review and meta-analysis. *The Journal of Sexual Medicine*, *19*(5), S185.
- 1271- Stanton, A. M., Handy, A. B., & Meston, C. M. (2017). 028 A Systematic Review of Sexual Function in Adolescents and Young Adults Diagnosed with Cancer. *The Journal of Sexual Medicine*, *14*(6), e360.
- 2743- Arden-Close, E., Eiser, C., & Pacey, A. (2011). Sexual functioning in male survivors of lymphoma: a systematic review (CME). *The Journal of Sexual Medicine*, *8*(7), 1833-1840.
- 3837 - Steinke, E., Palm Johansen, P., Fridlund, B., & Broström, A. (2016). Determinants of sexual dysfunction and interventions for patients with obstructive sleep apnoea: a systematic review. *International journal of clinical practice*, *70*(1), 5-19.

7 Not a systematic review or meta-analysis :361- Knafo, R., Thombs, B. D., Jewett, L., Hudson, M., Wigley, F., & Haythornthwaite, J. A. (2009). (Not) talking about sex: a

systematic comparison of sexual impairment in women with systemic sclerosis and other chronic disease samples. *Rheumatology*, *48*(10), 1300-1303.

- 949- Rafati, Y., & Alzweri, L. (2022). Prevalence of Sexual Dysfunction in Parkinsonian Men: A Literature Review. *The Journal of Sexual Medicine*, *19*(4), S69.
- 2198- Mostafa, T., & Abdel-Hamid, I. A. (2021). Ejaculatory dysfunction in men with diabetes mellitus. *World Journal of Diabetes*, *12*(7), 954.
- 2203 - Roth, J. D., Spinoit, A. F., & Hoebeke, P. (2021). Sexual function and dysfunction in men with spina bifida. *Journal of Pediatric Urology*, *17*(2), 158-165.
- 2593- Botlani Esfahani, S., & Pal, S. (2019). Does metabolic syndrome impair sexual functioning in adults with overweight and obesity?. *International Journal of Sexual Health*, *31*(2), 170-185.
- 3400 - Bazzichi, L., Giacomelli, C., Rossi, A., Sernissi, F., Scarpellini, P., Consensi, A., & Bombardieri, S. (2012). Fibromyalgia and sexual problems. *Reumatismo*, *64*(4), 261-267.
- 500- Panjari, M., Bell, R. J., Burney, S., Bell, S., McMurrick, P. J., & Davis, S. R. (2012). Sexual function, incontinence, and wellbeing in women after rectal cancer—a review of the evidence. *The journal of sexual medicine*, *9*(11), 2749-2758.

5 Disease excluded from our research :41- Balzarro, M., Rubilotta, E., Mancini, V., Trabacchin, N., Oppezzi, L., Marzi, V. L., ... & Serati, M. (2019). Impact of overactive bladder-wet syndrome on female sexual function: a systematic review and meta-analysis. *Sexual Medicine Reviews*, *7*(4), 565-574.

- 55- Beesley, V. L., Alemayehu, C., & Webb, P. M. (2018). A systematic literature review of the prevalence of and risk factors for supportive care needs among women with gynaecological cancer and their caregivers. *Supportive Care in Cancer*, *26*(3), 701-710.
- 67- Bessa, A., Martin, R., Häggström, C., Enting, D., Amery, S., Khan, M. S., ... & Van Hemelrijck, M. (2020). Unmet needs in sexual health in bladder cancer patients: a systematic review of the evidence. *BMC urology*, *20*(1), 1-16.
- 159-de Souza, C., Santos, A. V. D. S. L., Rodrigues, E. C. G., & Dos Santos, M. A. (2021). Experience of sexuality in women with gynecological cancer: Meta-synthesis of qualitative studies. *Cancer Investigation*, *39*(8), 607-620.
- 3111- Wang, X., Yang, X., Cai, Y., Wang, S., & Weng, W. (2018). High prevalence of erectile dysfunction in diabetic men with depressive symptoms: a meta-analysis. *The Journal of Sexual Medicine*, *15*(7), 935-941.

6 full text not available:58- Bennett, C., Rebafka, A., Carrier, J., Cook, S., & Edwards, D. (2022). Impact of primary and recurrent genital herpes on the quality of life of young people and adults: a mixed methods systematic review. *JBI Evidence Synthesis*, *20*(6), 1406-1473.

- 266- He, W., Yang, Y., Liang, H., Huang, Z., & Jiang, J. (2022). Migraine Is Associated With High Risk of Erectile Dysfunction: A Systematic Review and Cumulative Analysis. *The Journal of Sexual Medicine*.
- 995-Jia, S. Z., Leng, J. H., Sun, P. R., & Lang, J. H. (2013). Prevalence and associated factors of female sexual dysfunction in women with endometriosis. *Obstetrics & Gynecology*, *121*(3), 601-606.
- 1884- Romano, L., Granata, L., Fusco, F., Napolitano, L., Cerbone, R., Priadko, K., ... & Romano, M. (2021). Sexual dysfunction in patients with chronic gastrointestinal and liver diseases: A neglected issue. *Sexual medicine reviews*.
- 2164 - 1. WCNR 2018 Poster Abstracts. Neurorehabilitation and Neural Repair. 2018;32(4-5):363-538. doi:10.1177/1545968318765498

- 3971- Champion, H. C. (2006). Erectile Dysfunction and Cardiovascular Disease: Carrots, Sticks, and Better Men's Health. *ADVANCED STUDIES IN MEDICINE*, *6*(4), 163.

10 Not measure the impact of chronic illness on sexuality :

- 6 - Abou-Kassem, D., Kurita, G. P., Sjøgren, P., & Diasso, P. D. (2022). Long-term opioid treatment and endocrine measures in patients with cancer-related pain: a systematic review. *Scandinavian Journal of Pain*.
- 32- Arthur, E. K. (2018, July). A systematic review of interventions for sexual well-being in women with gynecologic, anal, or rectal cancer. In *Oncology Nursing Forum* (Vol. 45, No. 4, pp. 469-482). Oncology Nursing Society.
- 79- Boothby, C. A., Dada, B. R., Rabi, D. M., Campbell, T. S., & Tang, K. L. (2018). The effect of cardiac rehabilitation attendance on sexual activity outcomes in cardiovascular disease patients: A systematic review. *Canadian Journal of Cardiology*, *34*(12), 1590-1599.
- 145- Crafa, A., Cannarella, R., Condorelli, R. A., La Vignera, S., & Calogero, A. E. (2020). Is there an association between vitamin D deficiency and erectile dysfunction? A systematic review and meta-analysis. *Nutrients*, *12*(5), 1411.
- 172- Du, X. L., Liu, L., Song, W., Zhou, X., & Lv, Z. T. (2016). Association between gout and erectile dysfunction: a systematic review and meta-analysis. *Plos one*, *11*(12), e0168784.
- 260- Hargrove, A., Penny, D. J., & Sawyer, S. M. (2005). Sexual and reproductive health in young people with congenital heart disease: a systematic review of the literature. *Pediatric cardiology*, *26*(6), 805-811.
- 527- Pöttgen, J., Rose, A., van de Vis, W., Engelbrecht, J., Pirard, M., Lau, S., ... & RiMS Special Interest Group Psychology and Neuropsychology. (2018). Sexual dysfunctions in MS in relation to neuropsychiatric aspects and its psychological treatment: a scoping review. *PloS one*, *13*(2), e0193381.
- 598- Shell, J. A. (2002, January). Evidence-based practice for symptom management in adults with cancer: sexual dysfunction. In *Oncology nursing forum* (Vol. 29, No. 1).
- 1124- Attar, F. S., Mohammad, M. A., & Almoamin, H. H. (2022). Long-term outcome of sexual function in sickle cell disease men with ischemic priapism: A systematic review. *Journal of Clinical Urology*, *15*(2), 114-117.

3905 - Courtois, F., Rodrigue, X., Côté, I., Boulet, M., Vézina, J. G., Charvier K., & Dahan, V. (2012). Sexual function and autonomic dysreflexia in men with spinal cord injuries: how should we treat?. *Spinal cord*, *50*(12), 869-877.
