# Supplementary material for: Sexual Dysfunction in Patients with Chronic Non-Genital Physical Disease: An Umbrella Review
Source: Int J Environ Res Public Health. 2025 Jan 24;22(2):157. doi: 10.3390/ijerph22020157 (PMC11855788; doi:10.3390/ijerph22020157)
Supplement: Supplementary file 1 [file ijerph-22-00157-s001.zip › searchEquationSupportMaterial.pdf]

Cochrane

([mh "Cardiovascular Diseases"] OR [mh "Heart diseases"] OR [mh "Pulmonary Embolism"] OR [mh "Phlebitis"] OR [mh "Hypercholesterolemia"] OR [mh "Hypertension"] OR [mh "Pulmonary arterial hypertension"] OR [mh "Pacemaker, Artificial"] OR [mh "Brain Neoplasms"] OR [mh "Melanoma"] OR [mh "Thyroid Diseases"] OR [mh "Adrenal Gland Diseases"] OR [mh "Pheochromocytoma"] OR [mh "Diabetes Mellitus"] OR [mh "Hyperpituitarism"] OR [mh "Diabetes Insipidus"] OR [mh "Obesity"] OR [mh "Lung diseases , Obstructive"] OR [mh "Sleep Apnea Syndromes"] OR [mh "Cystic Fibrosis"] OR [mh "Lung Diseases, Interstitial"] OR [mh "Rhinitis, Atrophic"] OR [mh "Ethmoid Sinusitis"] OR [mh "Vertigo"] OR [mh "Meniere Disease"] OR [mh "Nasal Polyps"] OR [mh "Otosclerosis"] OR [mh "Colonic polyps"] OR [mh "Stomach diverticulum"] OR [mh "Gastritis, Atrophic"] OR [mh "Gastroesophageal Reflux"] OR [mh "Esophageal Achalasia"] OR [mh "Hernia, Hiatal"] OR [mh "Colonic Diseases, Functional"] OR [mh "Celiac disease"] OR [mh "Colitis, Ulcerative"] OR [mh "Crohn Disease"] OR [mh "Liver Cirrhosis"] OR [mh "Back pain "] OR [mh "Neck Pain"] OR [mh "Intervertebral Disc Displacement"] OR [mh "Arthitris"] OR [mh "Sciatica"] OR [mh "Axial Spondyloarthritis"] OR [mh "Osteitis Deformans"] OR [mh "Bursitis"] OR [mh "Fatigue Syndrome, Chronic"] OR [mh "Crystal Arthropathies"] OR [mh "Scoliosis"] OR [mh "Reflex Sympathetic Dystrophy"] OR [mh "Headache Disorders"] OR [mh "Multiple sclerosis"] OR [mh "Epilepsy"] OR [mh "Alzheimer Disease"] OR [mh "Dementia, Vascular"] OR [mh "Parkinson Disease"] OR [mh "Trigeminal Neuralgia"] OR [mh "Guillain-Barre Syndrome"] OR [mh "Stroke"] OR [mh "Ischemic Attack, Transient"] OR [mh "Muscular Diseases"] OR [mh "Myasthenia Gravis"] OR [mh "Kidney Failure, Chronic"] OR [mh "Nephritis"] OR [mh "Polycystic Kidney Diseases"] OR [mh "Kidney Calculi"] OR [mh "Acne Vulgaris"] OR [mh "Rosacea"] OR [mh "Eczema"] OR [mh "Urticaria"] OR [mh "Psoriasis"] OR [mh "Vitiligo"] OR [mh "Alopecia"] OR [mh "Hidradenitis Suppurativa"] OR [mh "Linear IgA Bullous Dermatosis"] OR [mh "Herpes Simplex"] OR [mh "Macular Degeneration"] OR [mh "Low Tension Glaucoma"] OR [mh "Cataract"] OR [mh "Retinal Diseases"] OR [mh "Vitreous Detachment"] OR [mh "Vision Disorders"] OR [mh "Uveitis"] OR [mh "Scleritis"] OR [mh "Lupus Erythematosus, Systemic"] OR [mh "Antiphospholipid Syndrome"] OR [mh "Scleroderma, Systemic"] OR [mh "Granulomatosis with Polyangiitis"] OR [mh "Microscopic Polyangiitis"] OR [mh "Churg-Strauss Syndrome"] OR [mh "Cryoglobulinemia"] OR [mh "Arteritis"] OR [mh "Sarcoidosis"] OR [mh "Histiocytosis"] OR [mh "Ehlers-Danlos Syndrome"] OR [mh "Tuberculosis"] OR [mh "Acquired Immunodeficiency Syndrome"] OR [mh "Hepatitis, Chronic"] OR [mh "Lyme Disease"] OR [mh "Discitis"] OR [mh "Leukemia"] OR [mh "Hodgkin Disease"] OR [mh "Lymphoma, Large B-Cell, Diffuse"] OR [mh "Leukemia, Prolymphocytic, T-Cell"] OR [mh "Leukemia"] OR [mh "Leukemia, Myelogenous, Chronic, BCR-ABL Positive"] OR [mh "Multiple Myeloma"] OR [mh "Down Syndrome"] OR [mh "Myelodysplastic Syndromes"] OR [mh "Anemia, Hemolytic"] OR [mh "Anemia, Pernicious"] OR [mh "Purpura, Thrombocytopenic"] OR [mh "Thrombotic Microangiopathies"] OR [mh "Hemochromatosis "] OR [mh "Porphyrias"] OR [mh "Hemophilia A"] OR [mh "Fatigue Syndrome, Chronic"] OR [mh "Chronic Pain"] OR [mh "Bone Marrow Diseases"]) AND ([mh "Sexual dysfunction, physiological"] OR [mh "Sexual dysfunction, psychological"] OR [mh "Sexuality"] OR [mh "Sexual desire"] OR [mh "Sexual arousal"] OR [mh "Erectile Dysfunction"] OR [mh "Libido "] OR [mh "Orgasm"] OR [mh "Vaginismus"] OR [mh "Dyspareunia "])

PUBMED :

("Cardiovascular Diseases"[mh] OR "Heart diseases"[mh] OR "Pulmonary Embolism"[mh] OR "Phlebitis"[mh] OR "Hypercholesterolemia"[mh] OR "Hypertension"[mh] OR "Pulmonary arterial hypertension"[mh] OR "Pacemaker, Artificial/adverse effects"[mh] OR "Brain Neoplasms"[mh] OR

"Melanoma"[mh] OR "Thyroid Diseases"[mh] OR "Adrenal Gland Diseases"[mh] OR "Pheochromocytoma"[mh] OR "Diabetes Mellitus"[mh] OR "Hyperpituitarism"[mh] OR "Diabetes Insipidus"[mh] OR "Obesity"[mh] OR "Lung diseases , Obstructive"[mh] OR "Sleep Apnea Syndromes"[mh] OR "Cystic Fibrosis"[mh] OR "Lung Diseases, Interstitial"[mh] OR "Rhinitis, Atrophic"[mh] OR "Ethmoid Sinusitis"[mh] OR "Vertigo"[mh] OR "Meniere Disease"[mh] OR "Nasal Polyps"[mh] OR "Otosclerosis"[mh] OR "Colonic polyps"[mh] OR "Stomach diverticulum"[mh] OR "Gastritis, Atrophic"[mh] OR "Gastroesophageal Reflux"[mh] OR "Esophageal Achalasia"[mh] OR "Hernia, Hiatal"[mh] OR "Colonic Diseases, Functional"[mh] OR "Celiac disease"[mh] OR "Colitis, Ulcerative"[mh] OR "Crohn Disease"[mh] OR "Liver Cirrhosis"[mh] OR "Back pain "[mh] OR "Neck Pain"[mh] OR "Intervertebral Disc Displacement"[mh] OR "Arthitris"[mh] OR "Sciatica"[mh] OR "Axial Spondyloarthritis"[mh] OR "Osteitis Deformans"[mh] OR "Bursitis"[mh] OR "Fatigue Syndrome, Chronic"[mh] OR "Crystal Arthropathies"[mh] OR "Scoliosis"[mh] OR "Reflex Sympathetic Dystrophy"[mh] OR "Headache Disorders"[mh] OR "Multiple sclerosis"[mh] OR "Epilepsy"[mh] OR "Alzheimer Disease"[mh] OR "Dementia, Vascular"[mh] OR "Parkinson Disease"[mh] OR "Trigeminal Neuralgia"[mh] OR "Guillain-Barre Syndrome"[mh] OR "Stroke"[mh] OR "Ischemic Attack, Transient"[mh] OR "Muscular Diseases"[mh] OR "Myasthenia Gravis"[mh] OR "Kidney Failure, Chronic"[mh] OR "Nephritis"[mh] OR "Polycystic Kidney Diseases"[mh] OR "Kidney Calculi"[mh] OR "Acne Vulgaris"[mh] OR "Rosacea"[mh] OR "Eczema"[mh] OR "Urticaria"[mh] OR "Psoriasis"[mh] OR "Vitiligo"[mh] OR "Alopecia"[mh] OR "Hidradenitis Suppurativa"[mh] OR "Linear IgA Bullous Dermatosis"[mh] OR "Herpes Simplex"[mh] OR "Macular Degeneration"[mh] OR "Low Tension Glaucoma"[mh] OR "Cataract"[mh] OR "Retinal Diseases"[mh] OR "Vitreous Detachment"[mh] OR "Vision Disorders"[mh] OR "Uveitis"[mh] OR "Scleritis"[mh] OR "Lupus Erythematosus, Systemic"[mh] OR "Antiphospholipid Syndrome"[mh] OR "Scleroderma, Systemic"[mh] OR "Granulomatosis with Polyangiitis"[mh] OR "Microscopic Polyangiitis"[mh] OR "Churg-Strauss Syndrome"[mh] OR "Cryoglobulinemia"[mh] OR "Arteritis"[mh] OR "Sarcoidosis"[mh] OR "Histiocytosis"[mh] OR "Ehlers-Danlos Syndrome"[mh] OR "Tuberculosis"[mh] OR "Acquired Immunodeficiency Syndrome"[mh] OR "Hepatitis, Chronic"[mh] OR "Lyme Disease"[mh] OR "Discitis"[mh] OR "Leukemia"[mh] OR "Hodgkin Disease"[mh] OR "Lymphoma, Large B-Cell, Diffuse"[mh] OR "Leukemia, Prolymphocytic, T-Cell"[mh] OR "Leukemia, Myelogenous, Chronic, BCR-ABL Positive"[mh] OR "Multiple Myeloma"[mh] OR "Down Syndrome"[mh] OR "Myeloproliferative Syndrome, Transient"[Supplementary Concept] OR "Myelodysplastic Syndromes"[mh] OR "Anemia, Hemolytic"[mh] OR "Anemia, Pernicious"[mh] OR "Purpura, Thrombocytopenic"[mh] OR "Thrombotic Microangiopathies"[mh] OR "Hemochromatosis "[mh] OR "Porphyrias"[mh] OR "Hemophilia A"[mh] OR "Fatigue Syndrome, Chronic"[mh] OR "Chronic Pain"[mh] OR "Bone Marrow Diseases"[mh] OR "Basal Cell Epithelioma"[tiab] OR "Basal Cell Epitheliomas"[tiab] OR "Brain Metastase"[tiab] OR "Brain Metastases"[tiab] OR "cerebroma"[tiab] OR "Colon Adenocarcinoma"[tiab] OR "Colon Adenocarcinomas"[tiab] OR "dermatoma"[tiab] OR "encephalophyma"[tiab] OR "hypophysoma"[tiab] OR "kidney mass"[tiab] OR "kidney neoplastic mass"[tiab] OR "malignant melanomatosis"[tiab] OR "melanocarcinoma"[tiab] OR "Melanoma"[tiab] OR "melanomalignoma"[tiab] OR "Melanomas"[tiab] OR "naevocarcinoma"[tiab] OR "neoplastic lung"[tiab] OR "neoplastic mammary gland"[tiab] OR "nephroma"[tiab] OR "nevocarcinoma"[tiab] OR "Pituitary Adenoma"[tiab] OR "Pituitary Adenomas"[tiab] OR "rectal mass"[tiab] OR "rectum mass"[tiab] OR "renal mass"[tiab] OR "renal neoplastic mass"[tiab] OR "reninoma"[tiab] OR "Rodent Ulcer"[tiab] OR "Rodent Ulcers"[tiab] OR "squamous cell epithelioma"[tiab] OR "squamous epithelioma"[tiab] OR "Thyroid Adenoma"[tiab] OR "Thyroid Adenomas"[tiab] OR "angina"[tiab] OR "anginal attack"[tiab] OR "angiocardiopathy"[tiab] OR "angiocardiovascular disease"[tiab] OR "angiocardiovascular diseases"[tiab] OR "Angor Pectori"[tiab] OR "Aortic Valve Diseases"[tiab] OR "Aortic Valve Disorder"[tiab] OR "Aortic Valve Disorders"[tiab] OR "Arrhythmia"[tiab] OR "Arrythmia"[tiab] OR "Artificial Cardiac Pacemaker"[tiab] OR "Artificial Cardiac Pacemakers"[tiab] OR

"Artificial Pacemaker"[tiab] OR "Artificial Pacemakers"[tiab] OR "Atrial Fibrillation"[tiab] OR "Atrial Fibrillations"[tiab] OR "Auricular Fibrillation"[tiab] OR "Auricular Fibrillations"[tiab] OR "backward failure heart"[tiab] OR "backward failure, heart"[tiab] OR "Cardiac Arrest"[tiab] OR "Cardiac Arrhythmias"[tiab] OR "cardiac backward failure"[tiab] OR "cardiac conduction system disease"[tiab] OR "cardiac decompensation"[tiab] OR "Cardiac Disorders"[tiab] OR "cardiac disrhythmia"[tiab] OR "Cardiac Dysrhythmia"[tiab] OR "cardiac failure"[tiab] OR "cardiac failures"[tiab] OR "cardiac incompetence"[tiab] OR "cardiac incompetences"[tiab] OR "cardiac infarct"[tiab] OR "cardiac insufficien*"[tiab] OR "cardiac stand still"[tiab] OR "cardiac valve defect"[tiab] OR "cardiac valve disease"[tiab] OR "cardial decompensation"[tiab] OR "cardial infarct"[tiab] OR "cardial insufficiency"[tiab] OR "cardial valve disease"[tiab] OR "cardiovascular complication*"[tiab] OR "cardiovascular decompensation"[tiab] OR "cardiovascular disease"[tiab] OR "cardiovascular diseases"[tiab] OR "cardiovascular disorder"[tiab] OR "cardiovascular disorders"[tiab] OR "cardiovascular disturbance"[tiab] OR "cardiovascular disturbances"[tiab] OR "cardiovascular disturbancescardiovascular lesion*"[tiab] OR "cardiovascular failure"[tiab] OR "cardiovascular failures"[tiab] OR "cardiovascular incompetence"[tiab] OR "cardiovascular incompetences"[tiab] OR "cardiovascular insufficien*"[tiab] OR "cardiovascular lesion*"[tiab] OR "cardiovascular syndrome"[tiab] OR "cardiovascular syndromes"[tiab] OR "cardiovascular vegetative disorder"[tiab] OR "cardiovascular vegetative disorders"[tiab] OR "cholesteremia"[tiab] OR "cholesterinemia"[tiab] OR "cholesterolemia"[tiab] OR "Circulatory Disorders"[tiab] OR "complication cardiovascular"[tiab] OR "conduction defect"[tiab] OR "conduction defects"[tiab] OR "conduction disease"[tiab] OR "conduction diseases"[tiab] OR "conduction disorder"[tiab] OR "conduction disorders"[tiab] OR "conduction disturbance"[tiab] OR "conduction disturbances"[tiab] OR "Coronary Disorders"[tiab] OR "decompensatio cordis"[tiab] OR "decompensation, heart"[tiab] OR "ectopic heart rhythm"[tiab] OR "ectopic rhythm"[tiab] OR "Elevated Cholesterol"[tiab] OR "Elevated Cholesterols"[tiab] OR "heart aberrant conduction"[tiab] OR "heart arrhytmia"[tiab] OR "Heart Attack"[tiab] OR "Heart Attacks"[tiab] OR "heart backward failure"[tiab] OR "heart decompensation"[tiab] OR "heart disease"[tiab] OR "heart diseases"[tiab] OR "heart disorder"[tiab] OR "heart disorders"[tiab] OR "heart dysrhythmia"[tiab] OR "heart ectopic beat"[tiab] OR "heart ectopic ventricle contraction"[tiab] OR "heart failure"[tiab] OR "heart failures"[tiab] OR "heart incompetence"[tiab] OR "heart infarct"[tiab] OR "heart insufficien*"[tiab] OR "heart rhythm disorder"[tiab] OR "heart valve abnormalities"[tiab] OR "heart valve abnormality"[tiab] OR "heart valve defect"[tiab] OR "heart valve degeneration"[tiab] OR "Heart Valve Disease"[tiab] OR "heart valve diseases"[tiab] OR "heart valve lesion"[tiab] OR "Heart Valvular Disease"[tiab] OR "Heart Valvular Diseases"[tiab] OR "High Blood Pressure"[tiab] OR "High Blood Pressures"[tiab] OR "High Cholesterol Level"[tiab] OR "High Cholesterol Levels"[tiab] OR "Hypercholesteremia"[tiab] OR "Hypercholesteremias"[tiab] OR "hypercholesterinaemia"[tiab] OR "hypercholesterinemia"[tiab] OR "hypercholesterolaemia"[tiab] OR "Hypercholesterolemias"[tiab] OR "hypertensive disease"[tiab] OR "hypertensive effect"[tiab] OR "hypertensive pulmonary vascular disease"[tiab] OR "hypertensive response"[tiab] OR "insufficientia cardis"[tiab] OR "insufficientio cardis"[tiab] OR "lung embolism"[tiab] OR "lung embolization"[tiab] OR "lung embolus"[tiab] OR "lung emboly"[tiab] OR "lung microembolism"[tiab] OR "lung microembolization"[tiab] OR "lung microembolus"[tiab] OR "lung thromboembolism"[tiab] OR "major adverse cardiovascular event*"[tiab] OR "Myocardial Failure"[tiab] OR "Myocardial Infarct"[tiab] OR "myocardial insufficiency"[tiab] OR "Myocardial Infarcts"[tiab] OR "myocardium infarct"[tiab] OR "Periphlebitides"[tiab] OR "Periphlebitis"[tiab] OR "perivenous infection"[tiab] OR "Phlebitides"[tiab] OR "phlebitis superficialis"[tiab] OR "post phlebitis syndrome"[tiab] OR "postphlebitic disease"[tiab] OR "postphlebitis syndrome"[tiab] OR "Pulmonary Embolism"[tiab] OR "Pulmonary Embolisms"[tiab] OR "pulmonary embolization"[tiab] OR "pulmonary embolus"[tiab] OR "pulmonary hypertensive diseases"[tiab] OR "pulmonary hypertensive disorder"[tiab] OR "pulmonary

hypertensive disorders"[tiab] OR "pulmonary microembolism"[tiab] OR "pulmonary thromboembolic disease"[tiab] OR "Pulmonary Thromboembolism"[tiab] OR "Pulmonary Thromboembolisms"[tiab] OR "Raynauds Disease"[tiab] OR "Stenocardia"[tiab] OR "Stenocardias"[tiab] OR "superficial phlebitis"[tiab] OR "valvulopathies"[tiab] OR "valvulopathy"[tiab] OR "Vascular Disorders"[tiab] OR "Vasculitis"[tiab] OR "vein inflammation"[tiab] OR "venitis"[tiab] OR "venous inflammation"[tiab] OR "vitium cordis"[tiab] OR "Chronic Infectious Mononucleosis-Like Syndromes"[tiab] OR "chronic intractable pain"[tiab] OR "Royal Free Diseases"[tiab] OR "Systemic Exertion Intolerance Diseases"[tiab] OR "Chronic Pain"[tiab] OR "Chronic Pains"[tiab] OR "Achalasia"[tiab] OR "Achalasias"[tiab] OR "alcohol liver injury"[tiab] OR "alcoholic liver"[tiab] OR "Atrophic Gastritides"[tiab] OR "Atrophic Gastritis"[tiab] OR "autoimmune gastritis"[tiab] OR "bowel syndrome"[tiab] OR "cardiac herniation"[tiab] OR "cardioesophageal reflux"[tiab] OR "cardiooesophageal reflux"[tiab] OR "Cardiospasm"[tiab] OR "Cardiospasms"[tiab] OR "Celiac Disease"[tiab] OR "celiac syndrome"[tiab] OR "chronic inflammatory bowel diseases"[tiab] OR "chronic ulceration colon"[tiab] OR "cirrhosis"[tiab] OR "cleron disease"[tiab] OR "coeliac disease"[tiab] OR "coeliac syndrome"[tiab] OR "coeliaky"[tiab] OR "Colitis Gravis"[tiab] OR "colitis ulcerativa"[tiab] OR "colitis ulcerosa"[tiab] OR "colon disease"[tiab] OR "colon diseases"[tiab] OR "colon disorder"[tiab] OR "colon disorders"[tiab] OR "colon juvenile polyp"[tiab] OR "colon polypoid lesion"[tiab] OR "colon spasm"[tiab] OR "chronic colon ulceration"[tiab] OR "Colonic Polyp"[tiab] OR "Colonic Polyps"[tiab] OR "colonospasm"[tiab] OR "Crohns Disease"[tiab] OR "Crohn's Disease"[tiab] OR "Crohn's Enteritis"[tiab] OR "digestive disease"[tiab] OR "digestive diseases"[tiab] OR "digestive disorder"[tiab] OR "digestive disorders"[tiab] OR "digestive system disorders"[tiab] OR "enteritis regionalis"[tiab] OR "Esophageal Hernia*"[tiab] OR "Esophageal Reflux"[tiab] OR "esophageal regurgitation"[tiab] OR "esophagogastric reflux"[tiab] OR "esophagus hernia"[tiab] OR "esophagus reflux"[tiab] OR "functional colonic diseases"[tiab] OR "Gastric Acid Reflux"[tiab] OR "gastric atrophy"[tiab] OR "gastric regurgitation"[tiab] OR "Gastroduodenal Ulcer"[tiab] OR "Gastroduodenal Ulcers"[tiab] OR "gastroesophageal reflex"[tiab] OR "gastroesophageal regurgitation"[tiab] OR "gastroesophagus reflux"[tiab] OR "gastrointestinal disease"[tiab] OR "gastrointestinal diseases"[tiab] OR "gastrointestinal disorder"[tiab] OR "gastrointestinal disorders"[tiab] OR "gastrointestinal dysfunction*"[tiab] OR "gastrooesophageal reflex"[tiab] OR "gastrooesophageal reflux"[tiab] OR "Gastro-oesophageal Reflux"[tiab] OR "gastrooesophageal regurgitation"[tiab] OR "gee herter disease"[tiab] OR "gee thaysen disease"[tiab] OR "GERD"[tiab] OR "Gluten Enteropathies"[tiab] OR "Gluten Enteropathy"[tiab] OR "gluten induced enteropathy"[tiab] OR "gluten intolerance"[tiab] OR "Gluten Sensitive Enteropathy"[tiab] OR "Gluten-Sensitive Enteropathies"[tiab] OR "Gluten-Sensitive Enteropathy"[tiab] OR "Granulomatous Colitis"[tiab] OR "Granulomatous Enteritis"[tiab] OR "hernia hiatus esophagi"[tiab] OR "hiatal diaphragmatic hernia"[tiab] OR "Hiatal Hernia*"[tiab] OR "hiatus diaphragmatic hernia"[tiab] OR "hiatus esophagi hernia"[tiab] OR "Hiatus Hernia*"[tiab] OR "hiatus oesophageal hernia"[tiab] OR "hiatus oesophagus hernia"[tiab] OR "huebner herter disease"[tiab] OR "Idiopathic Proctocolitis"[tiab] OR "Ileocolitis"[tiab] OR "Inflammatory Bowel Disease"[tiab] OR "intestinal infantilism"[tiab] OR "Irritable Bowel Syndromes"[tiab] OR "Irritable Colon"[tiab] OR "Liver Fibrosis"[tiab] OR "Marginal Ulcer"[tiab] OR "Marginal Ulcers"[tiab] OR "Megaesophagus"[tiab] OR "morbus crohn"[tiab] OR "mucomembraneous colitis"[tiab] OR "mucomembranous colitis"[tiab] OR "mucosal colitis"[tiab] OR "Mucous Colitides"[tiab] OR "Mucous Colitis"[tiab] OR "oesophageal reflux"[tiab] OR "oesophageal regurgitation"[tiab] OR "oesophagogastric reflux"[tiab] OR "oesophagus reflux"[tiab] OR "Paraesophageal Hernia"[tiab] OR "para-esophageal hernia"[tiab] OR "Paraesophageal Hernias"[tiab] OR "paraesophageal herniation"[tiab] OR "para-esophageal herniation"[tiab] OR "paraoesophageal hernia"[tiab] OR "para-oesophageal hernia"[tiab] OR "paraoesophageal herniation"[tiab] OR "para-oesophageal herniation"[tiab] OR "Peptic Ulcers"[tiab] OR "Regional Enteritis"[tiab] OR "regional

enterocolitis"[tiab] OR "Regional Ileitides"[tiab] OR "Regional Ileitis"[tiab] OR "regurgitation, gastroesophageal"[tiab] OR "spastic colitis"[tiab] OR "spastic colon"[tiab] OR "Sprue"[tiab] OR "stomach hernia"[tiab] OR "Terminal Ileitis"[tiab] OR "Ulcerative Colitis"[tiab] OR "ulcerative colorectitis"[tiab] OR "ulcerative procto colitis"[tiab] OR "ulcerative proctocolitis"[tiab] OR "ulcerous colitis"[tiab] OR "unstable colon"[tiab] OR "acromegalia"[tiab] OR "Acromegalies"[tiab] OR "acromegalism"[tiab] OR "Acromegaly"[tiab] OR "Addison Disease"[tiab] OR "Addisons Disease"[tiab] OR "Addison's Disease"[tiab] OR "adipose tissue hyperplasia"[tiab] OR "adipositas"[tiab] OR "adiposity"[tiab] OR "adrenal cortex hyperplasia"[tiab] OR "adrenal cortical hyperplasia"[tiab] OR "adrenal failure"[tiab] OR "adrenal failures"[tiab] OR "adrenal gland disorders"[tiab] OR "adrenocortical hyperplasia"[tiab] OR "adrenocorticohyperplasia"[tiab] OR "akromegalia"[tiab] OR "Aldosteronism"[tiab] OR "antidiuretic hormone insufficiency"[tiab] OR "arenocortical hyperplasia"[tiab] OR "Basedow Disease"[tiab] OR "basedow syndrome"[tiab] OR "Basedows Disease"[tiab] OR "Basedow's Disease"[tiab] OR "chromaffin paraganglioma"[tiab] OR "chronic autoimmune thyroiditis"[tiab] OR "Chronic Lymphocytic Thyroiditides"[tiab] OR "Chronic Lymphocytic Thyroiditis"[tiab] OR "Conn Syndrome"[tiab] OR "Conns Syndrome"[tiab] OR "Conn's Syndrome"[tiab] OR "corpulency"[tiab] OR "Cushing syndrome"[tiab] OR "Cushings syndrome"[tiab] OR "Cushing's Syndrome"[tiab] OR "diabetes"[tiab] OR "diabetic"[tiab] OR "endocrinal disease"[tiab] OR "endocrinal diseases"[tiab] OR "endocrinal disorder"[tiab] OR "endocrinal disorders"[tiab] OR "endocrinal disturbance"[tiab] OR "endocrinal disturbances"[tiab] OR "endocrinal dysfunction*"[tiab] OR "endocrine disease"[tiab] OR "endocrine diseases"[tiab] OR "endocrine disorder"[tiab] OR "Endocrine Disorders"[tiab] OR "endocrine disturbance"[tiab] OR "endocrine disturbances"[tiab] OR "endocrine dysfunction*"[tiab] OR "endocrine gland disease"[tiab] OR "endocrine gland diseases"[tiab] OR "endocrine gland disorder"[tiab] OR "endocrine gland disorders"[tiab] OR "endocrine gland dysfunction*"[tiab] OR "endocrine syndrome"[tiab] OR "endocrine syndromes"[tiab] OR "endocrine system disease"[tiab] OR "endocrine system diseases"[tiab] OR "endocrine system disorder"[tiab] OR "endocrine system disorders"[tiab] OR "endocrine system dysfunction*"[tiab] OR "endocrinological disease"[tiab] OR "endocrinological diseases"[tiab] OR "endocrinological disorder"[tiab] OR "endocrinological disorders"[tiab] OR "endocrinological dysfunction*"[tiab] OR "excess body weight"[tiab] OR "Exophthalmic Goiter"[tiab] OR "Exophthalmic Goiters"[tiab] OR "exophthalmic goitre"[tiab] OR "exophthalmic hyperthyroidism"[tiab] OR "Extra Adrenal Pheochromocytoma"[tiab] OR "Extra-Adrenal Pheochromocytoma"[tiab] OR "familial hypoadrenocorticism"[tiab] OR "fat overload syndrome"[tiab] OR "feline hyperthyroidism"[tiab] OR "Graves Disease"[tiab] OR "Graves' Disease"[tiab] OR "Graves hyperthyroidism"[tiab] OR "graves syndrome"[tiab] OR "Graves's disease"[tiab] OR "Hashimoto Disease"[tiab] OR "hashimoto goiter"[tiab] OR "Hashimoto Struma"[tiab] OR "Hashimoto Syndrome"[tiab] OR "Hashimoto Thyroiditides"[tiab] OR "Hashimoto Thyroiditis"[tiab] OR "hashimoto thyroidosis"[tiab] OR "Hashimotos Disease"[tiab] OR "Hashimoto's Disease"[tiab] OR "Hashimoto's Struma"[tiab] OR "Hashimotos Syndrome"[tiab] OR "Hashimoto's Syndrome"[tiab] OR "Hashimoto's Syndromes"[tiab] OR "Hashimotos thyroiditis"[tiab] OR "Hashimoto's thyroiditis"[tiab] OR "hormonal disease"[tiab] OR "hormonal diseases"[tiab] OR "hormonal disorder"[tiab] OR "hormonal disorders"[tiab] OR "hormonal dysfunction*"[tiab] OR "hormone imbalance"[tiab] OR "hyperaldosterone"[tiab] OR "Hypercortisolism"[tiab] OR "hypermineralocorticidism"[tiab] OR "hypermineralocorticism"[tiab] OR "Hyperprolactinaemia*"[tiab] OR "Hyperprolactinemia"[tiab] OR "Hyperprolactinemias"[tiab] OR "hyperthyreoidism"[tiab] OR "hyperthyreosis"[tiab] OR "Hyperthyroid"[tiab] OR "hyperthyroidea"[tiab] OR "hyperthyroidosis"[tiab] OR "Hyperthyroids"[tiab] OR "hypogonadism"[tiab] OR "hypothyreoidism"[tiab] OR "hypothyreosis"[tiab] OR "hypothyroidea"[tiab] OR "hypothyroidism"[tiab] OR "Hypothyroidisms"[tiab] OR "hypothyroidosis"[tiab] OR "hypothyrosis"[tiab] OR "Inappropriate GH Secretion Syndrome"[tiab] OR

"Inappropriate GH Secretion Syndromes"[tiab] OR "Inappropriate Growth Hormone Secretion Syndrome"[tiab] OR "Inappropriate Growth Hormone Secretion Syndromes"[tiab] OR "Inappropriate Prolactin Secretion"[tiab] OR "Inappropriate Secretion Prolactin"[tiab] OR "lymphadenoid goiter"[tiab] OR "megalakria"[tiab] OR "mineralcorticoid excess syndrome"[tiab] OR "obesitas"[tiab] OR "obesity"[tiab] OR "overweight"[tiab] OR "phaeochromoblastoma"[tiab] OR "phaeochromocytoma"[tiab] OR "pheochromoblastoma"[tiab] OR "Pheochromocytomas"[tiab] OR "pheochromocytomata"[tiab] OR "pheochromocytomatosis"[tiab] OR "pheochromocytosis"[tiab] OR "polycystic ovary syndrome"[tiab] OR "polycystic ovary syndromes"[tiab] OR "Primary Adrenal Insufficiency"[tiab] OR "Primary Adrenocortical Insufficiencies"[tiab] OR "Primary Adrenocortical Insufficiency"[tiab] OR "Primary Hyperaldosteronism"[tiab] OR "Primary Hyperthyroidism"[tiab] OR "Primary Hypoadrenalism"[tiab] OR "Primary Hypoadrenalisms"[tiab] OR "Prolactin Hypersecretion Syndrome"[tiab] OR "Prolactin Hypersecretion Syndromes"[tiab] OR "Somatotropin Hypersecretion Syndrome"[tiab] OR "Somatotropin Hypersecretion Syndromes"[tiab] OR "struma hashimoto"[tiab] OR "thyroid deficiency"[tiab] OR "thyroid gland failure"[tiab] OR "thyroid gland hyperfunction"[tiab] OR "thyroid hyperfunction"[tiab] OR "thyroid insufficiency"[tiab] OR "Thyroid Stimulating Hormone Deficiency"[tiab] OR "thyroideal hyperfunction"[tiab] OR "Thyroid-Stimulating Hormone Deficiencies"[tiab] OR "Thyroid-Stimulating Hormone Deficiency"[tiab] OR "TSH Deficiencies"[tiab] OR "TSH Deficiency"[tiab] OR "Vasopressin Deficiency"[tiab] OR "angioleiomyoma"[tiab] OR "angiomyoma"[tiab] OR "elastomyofibroma"[tiab] OR "fibromyoma"[tiab] OR "fibromyomas"[tiab] OR "hemangioleiomyoma"[tiab] OR "hemangiomyoma"[tiab] OR "Impotence"[tiab] OR "leimyoma"[tiab] OR "Leiomyomas"[tiab] OR "leyomyoma"[tiab] OR "myofibroma"[tiab] OR "myofibromatosis"[tiab] OR "vascular leiomyoma"[tiab] OR "addison anaemia"[tiab] OR "addison anemia"[tiab] OR "addisonian anaemia"[tiab] OR "addisonian anemia"[tiab] OR "ahf deficiency"[tiab] OR "ahg deficiency"[tiab] OR "anaemia perniciosa"[tiab] OR "anemia perniciosa"[tiab] OR "B12 deficiency anaemia"[tiab] OR "B12 deficiency anemia"[tiab] OR "B12 deficient anaemia"[tiab] OR "B12 deficient anemia"[tiab] OR "B12 vitamin deficiency anemia"[tiab] OR "biermer anaemia"[tiab] OR "biermer anemia"[tiab] OR "biermer disease"[tiab] OR "blood disease"[tiab] OR "blood diseases"[tiab] OR "blood disorder"[tiab] OR "blood disorders"[tiab] OR "blood dysfunction*"[tiab] OR "classic haemophilia"[tiab] OR "cobalamin deficiency anaemia"[tiab] OR "cobalamin deficiency anemia"[tiab] OR "congenital antihaemophilic factor deficiency"[tiab] OR "congenital antihaemophilic globulin deficiency"[tiab] OR "congenital antihemophilic factor deficiency"[tiab] OR "congenital antihemophilic globulin deficiency"[tiab] OR "drepanocytemia"[tiab] OR "drepanocytic anaemia"[tiab] OR "drepanocytic anemia"[tiab] OR "drepanocytosis"[tiab] OR "haematologic disease"[tiab] OR "haematologic diseases"[tiab] OR "haematologic disorder"[tiab] OR "haematologic disorders"[tiab] OR "haematologic dysfunction*"[tiab] OR "haematological disease"[tiab] OR "haematological diseases"[tiab] OR "haematological disorder"[tiab] OR "haematological disorders"[tiab] OR "haematological dysfunction*"[tiab] OR "haemochromatosis"[tiab] OR "haemoglobin SS"[tiab] OR "haemolytic anaemia"[tiab] OR "haemolytic disease"[tiab] OR "haemolytic syndrome"[tiab] OR "haemophilia a"[tiab] OR "haemophilia vera"[tiab] OR "Hb SS disease"[tiab] OR "heart hemochromatosis"[tiab] OR "hemachromatosis"[tiab] OR "hematologic disease"[tiab] OR "hematologic diseases"[tiab] OR "hematologic disorder"[tiab] OR "hematologic disorders"[tiab] OR "hematologic dysfunction*"[tiab] OR "hematopathy"[tiab] OR "hemoglobin SS"[tiab] OR "hemolytic anemia"[tiab] OR "hemolytic disease"[tiab] OR "hemolytic syndrome"[tiab] OR "hemopathie"[tiab] OR "hemopathies"[tiab] OR "hemopathy"[tiab] OR "hemophylia type a"[tiab] OR "hereditary iron overload"[tiab] OR "idiopathic hemochromatosis"[tiab] OR "intermittent porphyria"[tiab] OR "iron overload disease"[tiab] OR "iron overload disorder"[tiab] OR "iron overload syndrome"[tiab] OR "latent porphyria"[tiab] OR "lymphatic disease"[tiab] OR "lymphatic diseases"[tiab] OR "lymphatic disorder"[tiab] OR "lymphatic disorders"[tiab] OR "lymphatic

dysfunction*"[tiab] OR "macrocytic hyperchromic anaemia"[tiab] OR "macrocytic hyperchromic anemia"[tiab] OR "meniscocytosis"[tiab] OR "pernicious anaemia"[tiab] OR "pernicious anemia"[tiab] OR "porphyrias"[tiab] OR "porphyric disease"[tiab] OR "primary anaemia"[tiab] OR "primary anemia"[tiab] OR "primary congenital hemochromatosis"[tiab] OR "recklinghausen applebaum disease"[tiab] OR "sickle anaemia"[tiab] OR "sickle anemia"[tiab] OR "sickle cell anaemia"[tiab] OR "siderochromatosis"[tiab] OR "sulfhemoglobinem*"[tiab] OR "sulphaemoglobinaem*"[tiab] OR "systemic porphyria"[tiab] OR "thrombocytopaenia purpura"[tiab] OR "thrombocytopenia purpura"[tiab] OR "thrombotic purpura"[tiab] OR "true haemophilia"[tiab] OR "vitamin b 12 deficiency anaemia"[tiab] OR "vitamin b 12 deficiency anemia"[tiab] OR "a2 myeloma"[tiab] OR "Acquired Hemolytic Anemias"[tiab] OR "Acquired Immune Deficiency Syndrome Virus"[tiab] OR "acquired immunodeficiency"[tiab] OR "Addison Anemias"[tiab] OR "Addisons Anemia"[tiab] OR "Addison's Anemias"[tiab] OR "AIDS"[tiab] OR "aleukaemia"[tiab] OR "aleukemia"[tiab] OR "Arthriti*"[tiab] OR "arthrochondritis"[tiab] OR "arthrosynovitis"[tiab] OR "B. burgdorferi Infection*"[tiab] OR "bone marrow dysplasia"[tiab] OR "Bone Marrow Fibros*"[tiab] OR "Borrelia burgdorferi Infection*"[tiab] OR "Bronze Diabete"[tiab] OR "Bronzed Cirrhoses"[tiab] OR "Chronic Hepatit*"[tiab] OR "chronic myeloleukaemia"[tiab] OR "chronic myeloleukemia"[tiab] OR "Discitides"[tiab] OR "discitis"[tiab] OR "disk space infection"[tiab] OR "Diskiti*"[tiab] OR "DLBCL"[tiab] OR "down disease"[tiab] OR "Downs syndrome"[tiab] OR "Down's syndrome"[tiab] OR "Dysmyelopoietic Syndrome*"[tiab] OR "endocardial inflammation"[tiab] OR "Endocarditi*"[tiab] OR "endo-carditis"[tiab] OR "endocardium inflammation"[tiab] OR "Erythremia*"[tiab] OR "Factor 8 Deficienc*"[tiab] OR "Factor VIII Deficienc*"[tiab] OR "Factor VIII defiency"[tiab] OR "Familial Hemochromatoses"[tiab] OR "Familial Hemochromatosis"[tiab] OR "Haemochromato"[tiab] OR "Haemochromatos"[tiab] OR "Haemolytic Anaemias"[tiab] OR "HbS Disease"[tiab] OR "Hematopoetic Myelodysplasias"[tiab] OR "hemoblastoma"[tiab] OR "Hemochromato"[tiab] OR "Hemochromatos"[tiab] OR "Hemoglobin S Disease"[tiab] OR "Hemoglobin S Diseases"[tiab] OR "Hemophilia"[tiab] OR "hemophilias"[tiab] OR "high-risk MDS"[tiab] OR "Hodgkin disease*"[tiab] OR "Hodgkin Granuloma*"[tiab] OR "hodgkin sclerosis"[tiab] OR "hodgkin's disease"[tiab] OR "Hodgkins Disease*"[tiab] OR "Hodgkins Granuloma"[tiab] OR "Hodgkin's Granuloma"[tiab] OR "HTLV-III"[tiab] OR "Human Immunodeficiency Virus*"[tiab] OR "Human T Cell Lymphotropic Virus Type III"[tiab] OR "Human T Lymphotropic Virus Type III"[tiab] OR "Human T-Cell Lymphotropic Virus Type III"[tiab] OR "Human T-Lymphotropic Virus Type III"[tiab] OR "infection by B. burgdorferi"[tiab] OR "infection by Borrelia burgdorferi"[tiab] OR "intervertebral disc infection"[tiab] OR "intervertebral disk infection"[tiab] OR "intervertebral disk inflammation"[tiab] OR "Iron Storage Disorder*"[tiab] OR "joint inflammation"[tiab] OR "Koch Disease*"[tiab] OR "Kochs Disease*"[tiab] OR "Koch's Disease*"[tiab] OR "langdon down syndrome"[tiab] OR "LAV-HTLV-III"[tiab] OR "leucaemia"[tiab] OR "leucemia"[tiab] OR "Leucocythaemia*"[tiab] OR "Leucocythemia*"[tiab] OR "leukaemia"[tiab] OR "Leukemia*"[tiab] OR "Lyme borrelioses"[tiab] OR "Lyme Borreliosis"[tiab] OR "Lyme's borrelioses"[tiab] OR "Lyme's borreliosis"[tiab] OR "Lymes disease"[tiab] OR "Lyme's disease"[tiab] OR "Lymphadenopathy Associated Virus*"[tiab] OR "Lymphadenopathy-Associated Virus*"[tiab] OR "lymphogranuloma maligne"[tiab] OR "lymphogranuloma malignum"[tiab] OR "lymphogranulomatosis"[tiab] OR "Malignant Granuloma*"[tiab] OR "Malignant Lymphogranuloma"[tiab] OR "Malignant Lymphogranulomas"[tiab] OR "Microangiopathic Anemias"[tiab] OR "Microangiopathic Hemolytic Anemias"[tiab] OR "mongolian idiocy"[tiab] OR "mongolism"[tiab] OR "mongoloid idiocy"[tiab] OR "mongoloidism"[tiab] OR "morbus hodgkin"[tiab] OR "myelodysplasia"[tiab] OR "myelodysplastic disease"[tiab] OR "myelodysplastic disorder"[tiab] OR "Myelodysplastic Syndrome*"[tiab] OR "Myelofibros*"[tiab] OR "Myeloid Metaplasia*"[tiab] OR "myeloplaxoma"[tiab] OR "Myeloscleros*"[tiab] OR "Nonleukemic Myelos*"[tiab] OR "oligoarthritis"[tiab] OR "Osler Vaquez Disease*"[tiab] OR "Osler-Vaquez Disease*"[tiab] OR

"Pernicious Anemias"[tiab] OR "Pigmentary Cirrhos*"[tiab] OR "Polyarthrit*"[tiab] OR "Polycythemia Ruba Vera*"[tiab] OR "Polycythemia Rubra Vera*"[tiab] OR "Primary Hemochromatosis"[tiab] OR "Primary Polycythemia*"[tiab] OR "reed sternberg disease"[tiab] OR "Sickle Cell Anemia"[tiab] OR "Sickle Cell Anemias"[tiab] OR "Sickle Cell Disease"[tiab] OR "Sickle Cell Diseases"[tiab] OR "sickle cell disorder"[tiab] OR "Sickle Cell Disorders"[tiab] OR "Sickling Disorder Due to Hemoglobin S"[tiab] OR "Spondylodisciti*"[tiab] OR "Spondylodiskiti*"[tiab] OR "TB disease"[tiab] OR "TB infection"[tiab] OR "Thrombocytopenic Purpura*"[tiab] OR "Thrombopenic Purpura*"[tiab] OR "Thrombotic Microangiopath*"[tiab] OR "translocation 15 21 22"[tiab] OR "trisomy 21 syndrome"[tiab] OR "Troisier Hanot Chauffard Syndrome"[tiab] OR "Troisier-Hanot-Chauffard Syndrome*"[tiab] OR "Tuberculos*"[tiab] OR "tuberculous infection"[tiab] OR "tuberculous lesion"[tiab] OR "Von Recklenhausen Applebaum Disease"[tiab] OR "Von Recklenhausen-Applebaum Diseases"[tiab] OR "Acute Autoimmune Neuropathies"[tiab] OR "Acute Autoimmune Neuropathy"[tiab] OR "Acute Cerebrovascular Accident*"[tiab] OR "acute cerebrovascular lesion"[tiab] OR "acute febrile polyneuritis"[tiab] OR "acute focal cerebral vasculopathy"[tiab] OR "Acute Infectious Polyneuritis"[tiab] OR "Acute Inflammatory Demyelinating Polyneuropath*"[tiab] OR "Acute Inflammatory Demyelinating Polyradiculoneuropath*"[tiab] OR "Acute Inflammatory Polyneuropath*"[tiab] OR "Acute Inflammatory Polyradiculoneuropath*"[tiab] OR "acute postinfective polyradiculoneuropathy"[tiab] OR "Acute Stroke*"[tiab] OR "Alzeimer"[tiab] OR "Alzheimer"[tiab] OR "Alzeimers disease"[tiab] OR "Alzeimer's disease"[tiab] OR "Alzheimer's Disease"[tiab] OR "Alzheimers Disease*"[tiab] OR "Alzheimer's Diseases"[tiab] OR "Alzheimer-Type Dementia"[tiab] OR "Anti-MuSK Myasthenia Gravis"[tiab] OR "Apoplex*"[tiab] OR "Arteriosclerotic Dementia*"[tiab] OR "Aura"[tiab] OR "Auras"[tiab] OR "autoimmune myasthenia gravis"[tiab] OR "Binswanger Disease*"[tiab] OR "Binswanger Encephalopath*"[tiab] OR "Binswangers Disease*"[tiab] OR "Binswanger's Disease*"[tiab] OR "Binswangers Encephalopath*"[tiab] OR "Binswanger's Encephalopath*"[tiab] OR "brain accident"[tiab] OR "brain attack"[tiab] OR "brain blood flow disturbance"[tiab] OR "brain insult"[tiab] OR "brain insultus"[tiab] OR "Brain TIA"[tiab] OR "Brain Vascular Accident*"[tiab] OR "Cephalgia Syndrome"[tiab] OR "Cephalgia Syndromes"[tiab] OR "cerebral insult"[tiab] OR "Cerebral Stroke*"[tiab] OR "cerebral vascular accident"[tiab] OR "cerebral vascular insufficiency"[tiab] OR "cerebro vascular accident"[tiab] OR "Cerebrovascular Accident"[tiab] OR "Cerebrovascular Accidents"[tiab] OR "cerebrovascular arrest"[tiab] OR "cerebrovascular failure"[tiab] OR "cerebrovascular injury"[tiab] OR "cerebrovascular insufficiency"[tiab] OR "cerebrovascular insult"[tiab] OR "Cerebrovascular Stroke*"[tiab] OR "cerebrum vascular accident"[tiab] OR "Chronic Daily Headache"[tiab] OR "Chronic Daily Headaches"[tiab] OR "Chronic Headache"[tiab] OR "Chronic Headaches"[tiab] OR "Chronic Progressive Subcortical Encephalopath*"[tiab] OR "comitial disease"[tiab] OR "congenital atonic sclerotic muscular dystrophy"[tiab] OR "congenital muscular dystrophy"[tiab] OR "CVA"[tiab] OR "CVAs"[tiab] OR "diffuse cortical sclerosis"[tiab] OR "Disseminated Sclerosis"[tiab] OR "Epileps*"[tiab] OR "epileptic"[tiab] OR "Epileptiform Neuralgia*"[tiab] OR "erb goldflam disease"[tiab] OR "falling sickness"[tiab] OR "Fisher syndrome"[tiab] OR "Fothergill Disease"[tiab] OR "Generalized Myasthenia Gravis"[tiab] OR "Guillain Barre"[tiab] OR "Guillain-Barré"[tiab] OR "Guillain-Barre"[tiab] OR "Guillain Barré Syndrome*"[tiab] OR "Headache Disorder"[tiab] OR "Headache Syndrome"[tiab] OR "Headache Syndromes"[tiab] OR "Idiopathic Parkinson Disease*"[tiab] OR "idiopathic parkinsonism"[tiab] OR "infectious neuronitis"[tiab] OR "inflammatory acute polyradiculoneuropathy"[tiab] OR "Inflammatory Polyneuropathy Acute"[tiab] OR "insular sclerosis"[tiab] OR "insultus cerebralis"[tiab] OR "Intractable Headache"[tiab] OR "Intractable Headaches"[tiab] OR "ischaemic attack"[tiab] OR "ischaemic cerebral attack"[tiab] OR "ischaemic seizure"[tiab] OR "ischemic attack"[tiab] OR "ischemic cerebral attack"[tiab] OR "ischemic seizure"[tiab] OR "lacunar dementia"[tiab] OR "Landry paralysis"[tiab] OR "Landry syndrome"[tiab] OR "Landry-Guillain-Barre Syndrome"[tiab] OR "Lewy

Body Parkinson Disease*"[tiab] OR "maternal myasthenia gravis"[tiab] OR "mini-stroke"[tiab] OR "multiinfarct dementia"[tiab] OR "multi-infarct dementia"[tiab] OR "multiinfarction dementia"[tiab] OR "multi-infarction dementia"[tiab] OR "multiple sclerosis"[tiab] OR "muscle dystrophia"[tiab] OR "muscle dystrophy"[tiab] OR "Muscle Specific Receptor Tyrosine Kinase Myasthenia Gravis"[tiab] OR "Muscle Specific Tyrosine Kinase Antibody Positive Myasthenia Gravis"[tiab] OR "Muscle-Specific Receptor Tyrosine Kinase Myasthenia Gravis"[tiab] OR "Muscle-Specific Tyrosine Kinase Antibody Positive Myasthenia Gravis"[tiab] OR "muscular dystrophia"[tiab] OR "muscular dystrophies"[tiab] OR "MuSK MG"[tiab] OR "MuSK Myasthenia Gravis"[tiab] OR "myasthenia gravis pseudoparalitica"[tiab] OR "myasthenia gravis pseudoparalytica"[tiab] OR "myodystrophia"[tiab] OR "myodystrophy"[tiab] OR "neonatal myasthenia gravis"[tiab] OR "nervous disease"[tiab] OR "nervous diseases"[tiab] OR "nervous disorder"[tiab] OR "nervous disorders"[tiab] OR "nervous dysfunction*"[tiab] OR "nervous system disease"[tiab] OR "nervous system diseases"[tiab] OR "nervous system disorder"[tiab] OR "nervous system disorders"[tiab] OR "nervous system dysfunction*"[tiab] OR "neurologic disease"[tiab] OR "neurologic diseases"[tiab] OR "neurologic disorder"[tiab] OR "neurologic disorders"[tiab] OR "neurologic dysfunction*"[tiab] OR "neurological disease"[tiab] OR "neurological diseases"[tiab] OR "neurological disorder"[tiab] OR "neurological disorders"[tiab] OR "neurological dysfunction*"[tiab] OR "neuromuscular disease"[tiab] OR "neuromuscular diseases"[tiab] OR "neuromuscular disorder"[tiab] OR "neuromuscular disorders"[tiab] OR "neuromuscular dysfunction*"[tiab] OR "Ocular Myasthenia Gravis"[tiab] OR "Paralysis Agitans"[tiab] OR "Parkinson dementia complex"[tiab] OR "Parkinsons disease"[tiab] OR "Parkinson disease"[tiab] OR "Parkinson's Disease*"[tiab] OR "Presenile Dementia"[tiab] OR "Primary Parkinsonism"[tiab] OR "Primary Senile Degenerative Dementia"[tiab] OR "sclerosis multiplex"[tiab] OR "Seizure Disorder*"[tiab] OR "Senile Dementia"[tiab] OR "stroke"[tiab] OR "Strokes"[tiab] OR "Subcortical Arteriosclerotic Encephalopath*"[tiab] OR "Subcortical Leukoencephalopathies"[tiab] OR "Subcortical Leukoencephalopathy"[tiab] OR "Tic Douloureux"[tiab] OR "transient brain ischaemia"[tiab] OR "transient brain ischemia"[tiab] OR "Transient Brain Stem Ischemia*"[tiab] OR "Transient Brainstem Ischemia*"[tiab] OR "Transient Cerebral Ischemia*"[tiab] OR "Transient Cerebral Ischaemia*"[tiab] OR "transient ischaemic attack*"[tiab] OR "transient ischaemic seizure*"[tiab] OR "Transient Ischemic Attack*"[tiab] OR "transient ischemic seizure*"[tiab] OR "Trifacial Neuralgia*"[tiab] OR "Trigeminal Neuralgia*"[tiab] OR "Vascular Dementia*"[tiab] OR "acute paranasal sinusitis"[tiab] OR "airway disease"[tiab] OR "airway diseases"[tiab] OR "airway disorder*"[tiab] OR "airway dysfunction*"[tiab] OR "Asthma*"[tiab] OR "Atrophic Rhinitides"[tiab] OR "Atrophic Rhinitis"[tiab] OR "Auditory Vertigo"[tiab] OR "Auditory Vertigos"[tiab] OR "Aural Vertigo"[tiab] OR "benign paroxysmal postural vertigo"[tiab] OR "benign postural paroxysmal vertigo"[tiab] OR "bronchitis chronica"[tiab] OR "cerebral vertigo"[tiab] OR "Chronic Airflow Obstruction*"[tiab] OR "chronic airway obstruction"[tiab] OR "Chronic Bronchitis"[tiab] OR "chronic bronchus infection"[tiab] OR "chronic emphysema"[tiab] OR "chronic obstructive bronchopulmonary disease"[tiab] OR "Chronic Obstructive Lung Disease"[tiab] OR "chronic obstructive lung disorder"[tiab] OR "Chronic Obstructive Pulmonary Disease"[tiab] OR "Chronic Obstructive Pulmonary Diseases"[tiab] OR "chronic obstructive pulmonary disorder"[tiab] OR "chronic pulmonary obstructive disease"[tiab] OR "chronic pulmonary obstructive disorder"[tiab] OR "COAD"[tiab] OR "cochlea hydrops"[tiab] OR "COPD"[tiab] OR "Cystic Fibrosis of Pancreas"[tiab] OR "diffuse parenchyma lung disease"[tiab] OR "Diffuse Parenchymal Lung Disease"[tiab] OR "Diffuse Parenchymal Lung Diseases"[tiab] OR "diffuse parenchymal pulmonary disease"[tiab] OR "diffuse parenchymal pulmonary disorder"[tiab] OR "endolymphatic hydrops"[tiab] OR "endolymphatic sac hydrops"[tiab] OR "Ethmoid Sinusitides"[tiab] OR "Ethmoid Sinusitis"[tiab] OR "Ethmoidal Sinusitides"[tiab] OR "Ethmoidal Sinusitis"[tiab] OR "fibrocystic disease"[tiab] OR "hydrops labyrinthi"[tiab] OR "Hypersomnia with Periodic Respiration"[tiab] OR "Interstitial Lung Disease"[tiab] OR "Interstitial Lung Diseases"[tiab] OR

"interstitial lung disorder"[tiab] OR "Interstitial Pneumonia"[tiab] OR "Interstitial Pneumonias"[tiab] OR "Interstitial Pneumonitides"[tiab] OR "Interstitial Pneumonitis"[tiab] OR "interstitial pneumopathy"[tiab] OR "interstitial pulmonary disease"[tiab] OR "interstitial pulmonary disorder"[tiab] OR "labyrinth hydrops"[tiab] OR "labyrinthal syndrome"[tiab] OR "lung allergy"[tiab] OR "lung chronic obstructive disease"[tiab] OR "Meniere Disease"[tiab] OR "Ménière Disease"[tiab] OR "Ménière Diseases"[tiab] OR "Meniere Syndrome"[tiab] OR "Ménière Vertigo"[tiab] OR "Menieres Disease"[tiab] OR "Meniere's Disease"[tiab] OR "Ménières Disease"[tiab] OR "Ménière's Disease"[tiab] OR "Ménière's Diseases"[tiab] OR "Menieres Syndrome"[tiab] OR "Meniere's Syndrome"[tiab] OR "Ménières Vertigo"[tiab] OR "Ménière's Vertigo"[tiab] OR "Ménière's Vertigos"[tiab] OR "Mucoviscidosis"[tiab] OR "mucoviscoidosis"[tiab] OR "nasal sinusitis"[tiab] OR "nocturnal apnea"[tiab] OR "nocturnal apnoea"[tiab] OR "obstructive chronic lung disease"[tiab] OR "obstructive chronic pulmonary disease"[tiab] OR "Otogenic Vertigo"[tiab] OR "Otogenic Vertigos"[tiab] OR "Otoscleroses"[tiab] OR "otosclerosis surgery"[tiab] OR "otosclerotic stapes"[tiab] OR "otosphongiosis"[tiab] OR "Otospongioses"[tiab] OR "Otospongiosis"[tiab] OR "Ozena"[tiab] OR "Ozenas"[tiab] OR "pancreas cystic disease"[tiab] OR "pancreas cystic fibrosis"[tiab] OR "Pancreas Fibrocystic Diseases"[tiab] OR "pancreas fibrosis"[tiab] OR "pancreatic cystic disease"[tiab] OR "Pancreatic Cystic Fibrosis"[tiab] OR "pancreatic fibrosis"[tiab] OR "paroxysmal labyrinthine vertigo"[tiab] OR "paroxysmal positional vertigo"[tiab] OR "pneumatosis"[tiab] OR "positional paroxysmal vertigo"[tiab] OR "Pulmonary Cystic Fibrosis"[tiab] OR "respiration disease"[tiab] OR "respiration diseases"[tiab] OR "respiration disorder"[tiab] OR "respiration disorders"[tiab] OR "respiration dysfunction*"[tiab] OR "respiration tract disease"[tiab] OR "respiration tract diseases"[tiab] OR "respiration tract disorder"[tiab] OR "respiration tract disorders"[tiab] OR "respiration tract dysfunction*"[tiab] OR "respiratory disease"[tiab] OR "respiratory diseases"[tiab] OR "respiratory disorder"[tiab] OR "respiratory disorders"[tiab] OR "respiratory illness*"[tiab] OR "respiratory tract disease"[tiab] OR "respiratory tract diseases"[tiab] OR "respiratory tract disorder"[tiab] OR "respiratory tract disorders"[tiab] OR "respiratory tract dysfunction"[tiab] OR "respiratory tract dysfunctioning"[tiab] OR "respiratory tract dysfunctions"[tiab] OR "rhinitis atrophica"[tiab] OR "sinusitis nasalis"[tiab] OR "Sleep Apnea"[tiab] OR "Sleep Apneas"[tiab] OR "sleep apnoea"[tiab] OR "Sleep Disordered Breathing"[tiab] OR "Sleep Hypopnea"[tiab] OR "Sleep Hypopneas"[tiab] OR "Sleep-Disordered Breathing"[tiab] OR "vertiginous disease"[tiab] OR "vertiginous disorder"[tiab] OR "vertiginous syndrome"[tiab] OR "vestibular vertigo"[tiab] OR "Age Related Osteoporosis"[tiab] OR "Age-Related Bone Loss"[tiab] OR "Age-Related Bone Losses"[tiab] OR "Age-Related Osteoporosis"[tiab] OR "Akureyri disease"[tiab] OR "Algodystrophic Syndrome"[tiab] OR "Algodystrophies"[tiab] OR "Algodystrophy"[tiab] OR "alibert bazin disease"[tiab] OR "arthropathic psoriasis"[tiab] OR "Arthroses"[tiab] OR "Arthrosis"[tiab] OR "axial spondylarthritis"[tiab] OR "Axial Spondyloarthritides"[tiab] OR "Axial Spondyloarthritis"[tiab] OR "AxSpA"[tiab] OR "Back Ache"[tiab] OR "Back Aches"[tiab] OR "Back Pain*"[tiab] OR "Backache"[tiab] OR "Backaches"[tiab] OR "backpain"[tiab] OR "beauvais disease"[tiab] OR "Bilateral Sciatica"[tiab] OR "Bilateral Sciaticas"[tiab] OR "Bone Paget Disease"[tiab] OR "Bone Pagets Disease"[tiab] OR "Calcium Pyrophosphate Deposition Disease"[tiab] OR "Calcium Pyrophosphate Dihydrate Deposition"[tiab] OR "Cervical Pain"[tiab] OR "Cervical Pains"[tiab] OR "Cervical Sympathetic Dystrophies"[tiab] OR "Cervical Sympathetic Dystrophy"[tiab] OR "Cervicalgia"[tiab] OR "Cervicalgias"[tiab] OR "Cervicodynia"[tiab] OR "Cervicodynias"[tiab] OR "chariot disease"[tiab] OR "Chondrocalcinoses"[tiab] OR "chronic articular rheumatism"[tiab] OR "chronic fatigue"[tiab] OR "Chronic Fatigue-Fibromyalgia Syndrome*"[tiab] OR "Chronic Infectious Mononucleosis Like Syndrome"[tiab] OR "Chronic Infectious Mononucleosis-Like Syndrome"[tiab] OR "chronic rheumatism"[tiab] OR "complex regional pain syndrome 1"[tiab] OR "complex regional pain syndrome type 1"[tiab] OR "CRPS 1"[tiab] OR "CRPS I"[tiab] OR "CRPS type 1"[tiab] OR "CRPS Type

I"[tiab] OR "CRPS-I"[tiab] OR "crystal arthropathies"[tiab] OR "crystalline arthropathy"[tiab] OR "degenerative joint disease"[tiab] OR "Diffuse Myofascial Pain Syndrome"[tiab] OR "disc hernia"[tiab] OR "Disc Herniation"[tiab] OR "Disc Herniations"[tiab] OR "disc prolapse"[tiab] OR "Disc Protrusion"[tiab] OR "Disc Protrusions"[tiab] OR "discal hernia"[tiab] OR "discal herniation"[tiab] OR "discus hernia"[tiab] OR "disk hernia"[tiab] OR "Disk Herniation"[tiab] OR "Disk Herniations"[tiab] OR "Disk Prolapse"[tiab] OR "Disk Prolapses"[tiab] OR "Disk Protrusion"[tiab] OR "Disk Protrusions"[tiab] OR "dorsalgia"[tiab] OR "epicondylalgia"[tiab] OR "epidemic neuromyasthenia"[tiab] OR "fatigue syndrome"[tiab] OR "fibro myalgia"[tiab] OR "Fibromyalgia Fibromyositis Syndrome"[tiab] OR "Fibromyalgia-Fibromyositis Syndrome"[tiab] OR "Fibromyalgia-Fibromyositis Syndromes"[tiab] OR "Fibromyalgias"[tiab] OR "Fibromyositis Fibromyalgia Syndrome"[tiab] OR "Fibromyositis-Fibromyalgia Syndrome"[tiab] OR "Fibromyositis-Fibromyalgia Syndromes"[tiab] OR "fibrositic nodule"[tiab] OR "Fibrositides"[tiab] OR "Fibrositis"[tiab] OR "hernia disci"[tiab] OR "hernia nuclei pulposi"[tiab] OR "Herniated Disc"[tiab] OR "Herniated Discs"[tiab] OR "Herniated Disk"[tiab] OR "Herniated Disks"[tiab] OR "herniated intervertebral disc"[tiab] OR "herniated intervertebral disk"[tiab] OR "herniated nucleus pulposus"[tiab] OR "herniated vertebral disc"[tiab] OR "herniated vertebral disk"[tiab] OR "hypertrophic infiltrative tendinitis"[tiab] OR "Iceland disease"[tiab] OR "Intervertebral Disc Displacement"[tiab] OR "Intervertebral Disc Displacements"[tiab] OR "Intervertebral Disk Displacement"[tiab] OR "Intervertebral Disk Displacements"[tiab] OR "intervertebral disk perforation"[tiab] OR "intervertebral disk rupture"[tiab] OR "intervertebral prolapse"[tiab] OR "Involutional Osteoporosis"[tiab] OR "ischias"[tiab] OR "ischiatic pain"[tiab] OR "Lateral Epicondylitides"[tiab] OR "Lateral Epicondylitis"[tiab] OR "Lateral Humeral Epicondylitides"[tiab] OR "Lateral Humeral Epicondylitis"[tiab] OR "loin pain"[tiab] OR "lowback pain"[tiab] OR "Lumbago"[tiab] OR "lumbal pain"[tiab] OR "lumbal syndrome"[tiab] OR "lumbalgesia"[tiab] OR "lumbalgia"[tiab] OR "lumbar pain"[tiab] OR "lumbar spine syndrome"[tiab] OR "lumbodynia"[tiab] OR "lumbosacral pain"[tiab] OR "lumbosacral root syndrome"[tiab] OR "lumbosacroiliac strain"[tiab] OR "Muscular Rheumatism"[tiab] OR "Myalgic Encephalomyelitis"[tiab] OR "Neck Ache"[tiab] OR "Neck Aches"[tiab] OR "Neck Pain*"[tiab] OR "Neckache"[tiab] OR "Neckaches"[tiab] OR "neuralgic shoulder amyotrophy"[tiab] OR "nodular tendinitis"[tiab] OR "nucleus pulposus hernia"[tiab] OR "Osseous Paget's Disease"[tiab] OR "osteitis deformans"[tiab] OR "Osteoarthritides"[tiab] OR "osteo-arthritis"[tiab] OR "Osteoarthroses"[tiab] OR "Osteoarthrosis"[tiab] OR "osteo-arthrosis"[tiab] OR "Osteoporoses"[tiab] OR "ostitis deformans"[tiab] OR "Paget Disease of Bone"[tiab] OR "Paget disease of the bone"[tiab] OR "Pagets bone disease"[tiab] OR "Paget's bone disease"[tiab] OR "Pagets disease of bone"[tiab] OR "Paget's Disease of Bone"[tiab] OR "Paget's disease of the bone"[tiab] OR "paralytic scoliosis"[tiab] OR "Post Traumatic Osteoporosis"[tiab] OR "posttraumatic dystrophy"[tiab] OR "post-traumatic dystrophy"[tiab] OR "Post-Traumatic Osteoporosis"[tiab] OR "Postviral Fatigue Syndromes"[tiab] OR "Primary Fibromyalgia"[tiab] OR "primary osteoarthritis"[tiab] OR "progressive scoliosis"[tiab] OR "Prolapsed Disc"[tiab] OR "Prolapsed Discs"[tiab] OR "Prolapsed Disk"[tiab] OR "Prolapsed Disks"[tiab] OR "Protruded Disc"[tiab] OR "Protruded Discs"[tiab] OR "Protruded Disk"[tiab] OR "Protruded Disks"[tiab] OR "Pseudogout"[tiab] OR "Psoriasis Arthropathica"[tiab] OR "psoriasis pustulosa arthropathica"[tiab] OR "Psoriatic Arthropathies"[tiab] OR "Psoriatic Arthropathy"[tiab] OR "psoriatic rheumatism"[tiab] OR "Reflex Sympathetic Dystrophies"[tiab] OR "Reflex Sympathetic Dystrophy"[tiab] OR "rheumarthritis"[tiab] OR "rheumatic disease"[tiab] OR "rheumatic diseases"[tiab] OR "rheumatoid disease"[tiab] OR "rheumatoid diseases"[tiab] OR "rheumatoid inflammation"[tiab] OR "rheumatological disease"[tiab] OR "rheumatological diseases"[tiab] OR "rheumatological disorder"[tiab] OR "rheumatological disorders"[tiab] OR "Royal Free Disease"[tiab] OR "Sciatic Neuralgia"[tiab] OR "Sciatic Neuralgias"[tiab] OR "sciatic pain"[tiab] OR "Scolioses"[tiab] OR "Secondary Fibromyalgia"[tiab] OR "Senile Osteoporosis"[tiab] OR "shoulder arm syndrome"[tiab]

OR "Shoulder Hand Syndrome"[tiab] OR "Shoulder-Hand Syndrome"[tiab] OR "Shoulder-Hand Syndromes"[tiab] OR "Slipped Disc"[tiab] OR "Slipped Discs"[tiab] OR "Slipped Disk"[tiab] OR "Slipped Disks"[tiab] OR "slipped intervertebral disc"[tiab] OR "slipped vertebral disc"[tiab] OR "spinal disk disease"[tiab] OR "Sudek Atrophy"[tiab] OR "Sudek's Atrophies"[tiab] OR "Sudeks Atrophy"[tiab] OR "Sudek's Atrophy"[tiab] OR "sympathetic dystrophy syndrome"[tiab] OR "Sympathetic Reflex Dystrophia"[tiab] OR "Sympathetic Reflex Dystrophias"[tiab] OR "sympathetic reflex dystrophy"[tiab] OR "Systemic Exertion Intolerance Disease"[tiab] OR "tendinopathy"[tiab] OR "tendinosis"[tiab] OR "tendonitis"[tiab] OR "tendonopathy"[tiab] OR "Tennis Elbow"[tiab] OR "Tennis Elbows"[tiab] OR "tenonitis"[tiab] OR "tenontitis"[tiab] OR "tenositis"[tiab] OR "Type I Complex Regional Pain Syndrome"[tiab] OR "Vertebrogenic Pain Syndrome"[tiab] OR "Vertebrogenic Pain Syndromes"[tiab] OR "Yuppie flu"[tiab] OR "Acne Inversa*"[tiab] OR "acne juvenilis"[tiab] OR "Acne Rosacea"[tiab] OR "Chronic Bullous Disease of Childhood"[tiab] OR "cutaneous disease"[tiab] OR "cutaneous diseases"[tiab] OR "cutaneus disorder"[tiab] OR "cutaneus disorders"[tiab] OR "dermal disease"[tiab] OR "dermal diseases"[tiab] OR "dermal disorder"[tiab] OR "dermal disorders"[tiab] OR "Drug induced Linear IgA Bullous Dermatos*"[tiab] OR "Drug-induced Linear IgA Bullous Dermatos*"[tiab] OR "Eczema*"[tiab] OR "Erythematotelangiectatic Rosacea"[tiab] OR "Granulomatous Rosacea"[tiab] OR "herpes"[tiab] OR "hidradenitis suppurativa"[tiab] OR "Hives"[tiab] OR "juvenile acne"[tiab] OR "Linear IgA Dermatos*"[tiab] OR "Linear IgA IgG Bullous Dermatos*"[tiab] OR "Linear IgA IgG Dermatos*"[tiab] OR "Ocular Rosacea"[tiab] OR "Palmoplantaris Pustulosis"[tiab] OR "Papulopustular Rosacea"[tiab] OR "Phymatous Rosacea"[tiab] OR "Psoriases"[tiab] OR "psoriasiform dermatitis"[tiab] OR "psoriasiform dermatosis"[tiab] OR "psoriasiform lesion"[tiab] OR "psoriasiform rash"[tiab] OR "psoriasiform skin rash"[tiab] OR "psoriatic epidermis"[tiab] OR "psoriatic skin"[tiab] OR "Pustular Psoriasis of Palms and Soles"[tiab] OR "Pustulosis of Palms and Soles"[tiab] OR "Pustulosis Palmaris et Plantaris"[tiab] OR "rhinophyma"[tiab] OR "rozacea"[tiab] OR "skin and connective tissue disease"[tiab] OR "skin and connective tissue diseases"[tiab] OR "skin and connective tissue disorder"[tiab] OR "skin and connective tissue disorders"[tiab] OR "skin disease"[tiab] OR "skin diseases"[tiab] OR "skin disorder"[tiab] OR "skin disorders"[tiab] OR "Suppurative Hidradeniti*"[tiab] OR "urticary"[tiab] OR "weal"[tiab] OR "wheal"[tiab] OR "whealing"[tiab] OR "willan lepra"[tiab] OR "active TB"[tiab] OR "Adamantiades-Behcet Disease*"[tiab] OR "Allergic Angiit*"[tiab] OR "Allergic Granulomatos*"[tiab] OR "Allergic Granulomatous and Angiitis"[tiab] OR "Allergic Granulomatous Angiit*"[tiab] OR "allergic granulomatous angitis"[tiab] OR "anonymous artery occlusion"[tiab] OR "Anti Phospholipid Antibody Syndrome*"[tiab] OR "Anti Phospholipid Syndrome*"[tiab] OR "Antiphospholipid Antibody Syndrome*"[tiab] OR "Anti-Phospholipid Antibody Syndrome*"[tiab] OR "antiphospholipid syndrome"[tiab] OR "Anti-Phospholipid Syndrome*"[tiab] OR "aorta arch syndrome"[tiab] OR "aortic arch syndromes"[tiab] OR "Aortitis Syndrome*"[tiab] OR "APLA syndrome"[tiab] OR "arteritis brachiocephalica"[tiab] OR "arteritis nodosa"[tiab] OR "autoimmune disease"[tiab] OR "autoimmune diseases"[tiab] OR "Behcet Disease*"[tiab] OR "Behçet Disease*"[tiab] OR "Behcet syndrome"[tiab] OR "behcet ulcer"[tiab] OR "Behcets disease"[tiab] OR "Behcet's Disease*"[tiab] OR "Behcets syndrome"[tiab] OR "Behcet's Syndrome*"[tiab] OR "besnier boeck syndrome"[tiab] OR "Besnier-Boeck Disease*"[tiab] OR "Besnier-Boeck-Schaumann Syndrome*"[tiab] OR "Boeck Disease*"[tiab] OR "Boecks Disease*"[tiab] OR "Boeck's Disease*"[tiab] OR "brachiocephalic arteritis"[tiab] OR "brachiocephalic artery occlusion"[tiab] OR "brachiocephalic ischaemia"[tiab] OR "brachiocephalic ischemia"[tiab] OR "brachiocephalic trunk occlusion"[tiab] OR "brachiocephalic vascular occlusion"[tiab] OR "Church Strauss syndrome"[tiab] OR "Churg Strauss"[tiab] OR "Churg-Strauss Syndrome"[tiab] OR "Cranial Arterit*"[tiab] OR "cryoglobulinaemia"[tiab] OR "Cryoglobulinemias"[tiab] OR "cryoimmunoglobulinaemia"[tiab] OR "cryoimmunoglobulinemia"[tiab] OR "Cutis Elastica"[tiab] OR "dacryosialoadenopathia atrophicans"[tiab] OR "EDS IV"[tiab] OR "Ehlers Danlos"[tiab] OR "Ehlers-Danlos Disease*"[tiab] OR

"Ehlers-Danlos syndrome"[tiab] OR "eosinophilic GPA"[tiab] OR "eosinophilic granulomatosis polyangiitis"[tiab] OR "eosinophilic granulomatosis polyangitis"[tiab] OR "eosinophilic granulomatous angiitis"[tiab] OR "Eosinophilic Granulomatous Vasculit*"[tiab] OR "erythematodes visceralis"[tiab] OR "Essential Polyarterit*"[tiab] OR "generalised scleroderma"[tiab] OR "generalized scleroderma"[tiab] OR "Giant Cell Aortic Arteritis"[tiab] OR "Giant Cell Aortiti*"[tiab] OR "giant cell arteriitis"[tiab] OR "Giant Cell Arteriti*"[tiab] OR "gougerot houwer sjoegren syndrome"[tiab] OR "gougerot mulock houwer sjoegren syndrome"[tiab] OR "Gougerot Sjoegren syndrome"[tiab] OR "Gougerot-Sjogren syndrome"[tiab] OR "granulomatosis and polyangiitis"[tiab] OR "granulomatosis and polyangitis"[tiab] OR "Granulomatosis with Polyangiit*"[tiab] OR "granulomatosis with polyangitis"[tiab] OR "granulomatous allergic angitis"[tiab] OR "granulomatous polyangiitis"[tiab] OR "granulomatous polyangitis"[tiab] OR "Horton arteritis"[tiab] OR "Horton Disease"[tiab] OR "Horton's arteritis"[tiab] OR "Hortons Disease"[tiab] OR "Horton's Disease"[tiab] OR "Hughes Syndrome*"[tiab] OR "innominate arterial ligation"[tiab] OR "innominate artery ligation"[tiab] OR "innominate artery occlusion"[tiab] OR "jungling syndrome"[tiab] OR "kussmaul maier disease"[tiab] OR "kussmaul syndrome"[tiab] OR "Libman Sacks Disease*"[tiab] OR "Libman-Sacks Disease*"[tiab] OR "lupovisceritis"[tiab] OR "lymphogranuloma benignum"[tiab] OR "malignant dermatovisceritism"[tiab] OR "martorell syndrome"[tiab] OR "Microscopic Polyangiitides"[tiab] OR "microscopic polyarteritis"[tiab] OR "mikulicz radecki syndrome"[tiab] OR "mixed cryoglobulinemia"[tiab] OR "morbus Wegener"[tiab] OR "mucoserous dyssecretosis"[tiab] OR "mukilicz radecki syndrome"[tiab] OR "multisystem disease"[tiab] OR "multisystem diseases"[tiab] OR "multisystem disorder"[tiab] OR "multisystem disorders"[tiab] OR "multisystem dysfunction*"[tiab] OR "necrotising respiratory granulomatosis"[tiab] OR "Necrotizing Arterit*"[tiab] OR "necrotizing respiratory granulomatosis"[tiab] OR "nodular periarteritis"[tiab] OR "nodular polyarteritis"[tiab] OR "oculobuccopharyngeal dryness"[tiab] OR "Old Silk Route Disease*"[tiab] OR "panarteriitis nodosa"[tiab] OR "panarteritis nodosa"[tiab] OR "periarterial fibrosis"[tiab] OR "periarteriitis nodosa"[tiab] OR "Periarteritis Nodosa"[tiab] OR "pneumogenic granulomatosis"[tiab] OR "poliarteritis nodosa"[tiab] OR "polyarteriitis nodosa"[tiab] OR "progressive scleroderma"[tiab] OR "progressive sclerodermia"[tiab] OR "Pulseless Disease*"[tiab] OR "reversed coarctation"[tiab] OR "rheumatic sialosis"[tiab] OR "sarcoid"[tiab] OR "sarcoidoses"[tiab] OR "Schaumann Disease*"[tiab] OR "Schaumann Syndrome*"[tiab] OR "Schaumann's Syndrome*"[tiab] OR "sicca syndrome"[tiab] OR "sjoegren disease"[tiab] OR "sjogren disease"[tiab] OR "sjogren syndrome"[tiab] OR "sjogren's syndrome"[tiab] OR "lupus"[tiab] OR "Strauss Churg syndrome"[tiab] OR "systemic disease"[tiab] OR "systemic diseases"[tiab] OR "systemic disorder*"[tiab] OR "systemic dysfunction*"[tiab] OR "systemic progressive sclerosis"[tiab] OR "Systemic Scleroderma"[tiab] OR "Systemic Sclerosis"[tiab] OR "takayasu arteriopathy"[tiab] OR "Takayasu Arteritis"[tiab] OR "Takayasu Disease*"[tiab] OR "takayasu ohnishi syndrome"[tiab] OR "Takayasu Syndrome*"[tiab] OR "Takayasus Arteritis"[tiab] OR "Takayasu's Arteritis"[tiab] OR "Temporal Arterit*"[tiab] OR "Triple Symptom Complex"[tiab] OR "Triple Symptom Complices"[tiab] OR "Triple-Symptom Complex"[tiab] OR "Wegener disease"[tiab] OR "Wegener granuloma"[tiab] OR "Wegener Granulomatosis"[tiab] OR "Wegener Klinger Churg syndrome"[tiab] OR "Wegener Klinger granulomatosis"[tiab] OR "Wegener syndrome"[tiab] OR "Wegener's disease"[tiab] OR "Wegener's GPA"[tiab] OR "Wegener's granuloma"[tiab] OR "Wegener's Granulomatosis"[tiab] OR "Wegner granulomatosis"[tiab] OR "Young Female Arterit*"[tiab] OR "Anti GBM Disease*"[tiab] OR "Anti Glomerular Basement Membrane Disease"[tiab] OR "Anti-GBM Disease*"[tiab] OR "bladder incontinence"[tiab] OR "chronic disease kidney function"[tiab] OR "Chronic Kidney Failure*"[tiab] OR "chronic nephropathy"[tiab] OR "Chronic Renal Failure*"[tiab] OR "cystic kidney"[tiab] OR "End Stage Kidney Disease*"[tiab] OR "End Stage Renal Disease*"[tiab] OR "End Stage Renal Failure*"[tiab] OR "End-Stage Kidney Disease*"[tiab] OR "End-Stage Renal Disease*"[tiab] OR "End-Stage Renal Failure*"[tiab] OR

"ESRD"[tiab] OR "familial nephrolithiasis"[tiab] OR "goodpasture disease"[tiab] OR "goodpasture renopulmonary syndrome"[tiab] OR "Goodpasture Syndrome*"[tiab] OR "Goodpastures Syndrome*"[tiab] OR "Goodpasture's Syndrome*"[tiab] OR "incontinentia urinae"[tiab] OR "involuntary urinary loss"[tiab] OR "involuntary urination"[tiab] OR "involuntary urine loss"[tiab] OR "Kidney Calcul*"[tiab] OR "kidney calix stone"[tiab] OR "kidney calyx stone"[tiab] OR "kidney chronic failure"[tiab] OR "kidney cystic disease"[tiab] OR "kidney disease"[tiab] OR "kidney diseases"[tiab] OR "kidney disorder"[tiab] OR "kidney disorders"[tiab] OR "kidney failure"[tiab] OR "kidney failures"[tiab] OR "kidney insufficien*"[tiab] OR "kidney lithiasis"[tiab] OR "kidney multicystic aplasia"[tiab] OR "kidney multicystic disease"[tiab] OR "kidney pelvis stone"[tiab] OR "kidney polycystosis"[tiab] OR "Kidney Stone*"[tiab] OR "leakage of urine"[tiab] OR "Lung Purpura with Nephritis"[tiab] OR "male genital disorder"[tiab] OR "male genital disorders"[tiab] OR "male infertility"[tiab] OR "Nephrolith"[tiab] OR "pneumorenal syndrome"[tiab] OR "Polycystic Kidney"[tiab] OR "Polycystic Kidneys"[tiab] OR "prostate adenoma"[tiab] OR "Renal Calcul*"[tiab] OR "renal cystic disease"[tiab] OR "renal disease"[tiab] OR "renal diseases"[tiab] OR "renal disorder"[tiab] OR "renal disorders"[tiab] OR "renal failure"[tiab] OR "renal failures"[tiab] OR "renal insufficien*"[tiab] OR "renal pelvis stone"[tiab] OR "renal polycystic disease"[tiab] OR "renal stone"[tiab] OR "renolithiasis"[tiab] OR "unwanted urine loss"[tiab] OR "urinary incontinence"[tiab] OR "urinary leakage"[tiab] OR "urine incontinence"[tiab] OR "urine leakage"[tiab] OR "urine wetting"[tiab] OR "urologic disease*"[tiab] OR "urologic disorder*"[tiab] OR "urological disease*"[tiab] OR "urological disorder*"[tiab] OR "Age Related Macular Degeneration"[tiab] OR "Age-Related Macular Degeneration*"[tiab] OR "atrophia maculae luteae"[tiab] OR "bilateral macular degeneration"[tiab] OR "Cataract*"[tiab] OR "chronic uveitis"[tiab] OR "corpus vitreum detachment"[tiab] OR "Day Blindness"[tiab] OR "degeneratio maculae luteae retinae"[tiab] OR "detachment corporis vitrei"[tiab] OR "detachment vitreous"[tiab] OR "disciform macular degeneration"[tiab] OR "Episclerit*"[tiab] OR "eye disease"[tiab] OR "eye diseases"[tiab] OR "eye disorder"[tiab] OR "eye disorders"[tiab] OR "eye dysfunction"[tiab] OR "eye dysfunctioning"[tiab] OR "eye dysfunctions"[tiab] OR "Hemeralopia*"[tiab] OR "heredomacular degeneration"[tiab] OR "immunogenic uveitis"[tiab] OR "junius kuhnt disease"[tiab] OR "lens clouding"[tiab] OR "Lens Opacit*"[tiab] OR "Macropsia*"[tiab] OR "macula atrophy"[tiab] OR "macula bilateral degeneration"[tiab] OR "macula degeneration"[tiab] OR "macula lutea atrophy"[tiab] OR "macula lutea degeneration"[tiab] OR "macula lutea disciform degeneration"[tiab] OR "macula lutea retina atrophy"[tiab] OR "macula lutea retina degeneration"[tiab] OR "macula retina atrophy"[tiab] OR "macula retina degeneration"[tiab] OR "macular atrophy"[tiab] OR "Macular Degenerations"[tiab] OR "macular disciform degeneration"[tiab] OR "Macular Dystroph*"[tiab] OR "Maculopath*"[tiab] OR "Metamorphopsia*"[tiab] OR "Micropsia*"[tiab] OR "Myopia*"[tiab] OR "Nearsightedness*"[tiab] OR "Necrotizing Sclerit*"[tiab] OR "ocular disease"[tiab] OR "ocular diseases"[tiab] OR "ocular disorder"[tiab] OR "ocular disorders"[tiab] OR "ocular dysfunction*"[tiab] OR "ophthalmic disease"[tiab] OR "ophthalmic diseases"[tiab] OR "ophthalmic disorder"[tiab] OR "ophthalmic disorders"[tiab] OR "ophthalmic dysfunction*"[tiab] OR "ophthalmologic disease"[tiab] OR "ophthalmologic diseases"[tiab] OR "ophthalmologic disorder"[tiab] OR "ophthalmologic disorders"[tiab] OR "ophthalmologic dysfunction*"[tiab] OR "panuveitis"[tiab] OR "posterior capsule opacification"[tiab] OR "posterior uveitis"[tiab] OR "Presbyopias"[tiab] OR "Pseudoaphakia*"[tiab] OR "retina macula disciform degeneration"[tiab] OR "retinal diseases"[tiab] OR "Scleritides"[tiab] OR "secondary scleritis"[tiab] OR "suppurative uveitis"[tiab] OR "Uveitides"[tiab] OR "Vision Disabilit*"[tiab] OR "vision disorder"[tiab] OR "vision disorders"[tiab] OR "vision disturbance"[tiab] OR "visual disorder"[tiab] OR "visual disorders"[tiab] OR "visual disturbance"[tiab] OR "Visual Impairment*"[tiab] OR "vitreous detachment"[tiab] OR "sarcoidosis"[tiab] OR "neoplasia"[tiab] OR "lymphoma*"[tiab] OR "hypertension"[tiab] OR "infarction*"[tiab] OR "glaucom*"[tiab] OR

"cancer*"[tiab] OR "carcinoma*"[tiab] OR "neoplasm*"[tiab] OR "tumor*"[tiab] OR "tumour*"[tiab] OR "Horton syndrome"[tiab] OR "Horton's syndrome"[tiab] OR "Gastro-esophageal reflux"[tiab] OR "gastroesophageal reflux"[tiab] OR "GORD"[tiab]) AND ("Sexual dysfunction, physiological"[mh] OR "Sexual dysfunction, psychological"[mh] OR "Sexuality"[mh:noexp] OR "Sexual desire"[mh] OR "Sexual arousal"[mh] OR "Erectile Dysfunction"[mh] OR "Libido "[mh] OR "Orgasm"[mh] OR "Vaginismus"[mh] OR "Dyspareunia "[mh] OR "coitus"[tiab] OR "desire"[tiab] OR "dyspareunia"[tiab] OR "ego-dystonic homosexuality"[tiab] OR "ejaculatio praecox"[tiab] OR "ejaculatio praecoxs"[tiab] OR "ejaculatio precox"[tiab] OR "erectile dysfunction*"[tiab] OR "erection"[tiab] OR "frigidities"[tiab] OR "frigidity"[tiab] OR "genital disorder"[tiab] OR "genital disorders"[tiab] OR "go-dystonic homosexuality"[tiab] OR "libido"[tiab] OR "orgasm"[tiab] OR "orgasms"[tiab] OR "premature ejaculation"[tiab] OR "premature ejaculations"[tiab] OR "psychosexuality"[tiab] OR "sex abnormalit*"[tiab] OR "sex arousal"[tiab] OR "sex disorder"[tiab] OR "sex disorders"[tiab] OR "sex drive"[tiab] OR "sex dysfunction*"[tiab] OR "sex insufficienc*"[tiab] OR "sex problem"[tiab] OR "sex problems"[tiab] OR "vaginismus"[tiab] OR "sexual"[tiab] OR "psychosexual"[tiab] OR "sexuality"[tiab]) AND ("adult"[mh] OR "adult"[tiab] OR "adults"[tiab] OR "old people"[tiab] OR "elderly"[tiab] OR "frail"[tiab] OR "frailness"[tiab] OR "frailty"[tiab] OR "old age"[tiab] OR "old patients"[tiab] OR "old person"[tiab] OR "old persons"[tiab] OR "older adult"[tiab] OR "older adults"[tiab] OR "older patient"[tiab] OR "older patients"[tiab] OR "older people"[tiab] OR "older person"[tiab] OR "older persons"[tiab] OR "senior people"[tiab] OR "senior person"[tiab] OR "senior persons"[tiab] OR "seniors"[tiab]) AND ("Systematic review"[pt] OR "Meta-Analysis"[pt] OR "scoping review*"[tiab] OR "systematic review*"[tiab] OR "systematic literature review*"[tiab] OR "systematic narrative review*"[tiab] OR "systematic qualitative review*"[tiab] OR "systematic evidence review*"[tiab] OR "systematic quantitative review*"[tiab] OR "systematic meta-review*"[tiab] OR "systematic critical review*"[tiab] OR "systematic mapping review*"[tiab] OR "systematic cochrane review*"[tiab] OR "systematic electronic literature search*"[tiab] OR "PRISMA"[tiab] OR "systematic descriptive review*"[tiab] OR "systematic analys*"[tiab] OR "targeted literature review*"[tiab] OR "meta-synthes*"[tiab] OR "comprehensive review*"[tiab] OR "mixed studies review*"[tiab] OR "sistematic review*"[tiab] OR "umbrella review*"[tiab] OR "mini-review*"[tiab] OR "rapid literature review*"[tiab] OR "rapid review*"[tiab] OR "integrative review*"[tiab] OR "systematic and critical analysis review*"[tiab] OR "systematically review evidence"[tiab] OR "systematic search*"[tiab] OR "systematic methodological review*"[tiab] OR "metaanalys*"[tiab] OR "meta-analytic review*"[tiab] OR "meta-analys*"[tiab] OR "cochrane review*"[tiab])

EMBASE :

('Cardiovascular Diseases'/exp OR 'heart infarction'/exp OR 'Angina pectoris'/exp OR 'Heart failure'/exp OR 'lung embolism'/exp OR 'Phlebitis'/exp OR 'Hypercholesterolemia'/exp OR 'Hypertension'/exp OR 'pulmonary hypertension'/exp OR 'heart arrhythmia'/exp OR 'heart muscle conduction disturbance'/exp OR 'valvular heart disease'/exp OR 'brain tumor'/exp OR 'colon tumor'/exp OR 'rectum tumor'/exp OR 'stomach tumor'/exp OR 'liver tumor'/exp OR 'gallbladder tumor'/exp OR 'bone tumor'/exp OR 'melanoma'/exp OR 'skin tumor'/exp OR 'squamous cell carcinoma'/exp OR 'esophagus tumor'/exp OR 'head and neck tumor'/exp OR 'pancreas tumor'/exp OR 'lung tumor'/exp OR 'kidney tumor'/exp OR 'thyroid tumor'/exp OR 'bladder tumor'/exp OR 'hypophysis tumor'/exp OR 'Graves Disease'/exp OR 'Hashimoto Disease'/exp OR 'hyperthyroidism'/exp OR 'hypothyroidism'/exp OR 'Addison Disease'/exp OR 'pheochromocytoma'/exp OR 'diabetes mellitus'/exp OR 'Cushing syndrome'/exp OR 'acromegaly'/exp OR 'hyperprolactinemia'/exp OR 'diabetes insipidus'/exp OR 'hyperaldosteronism'/exp OR 'Obesity'/exp OR 'chronic bronchitis'/exp OR 'chronic obstructive lung disease'/exp OR 'Emphysema'/exp OR 'asthma'/exp OR 'sleep disordered breathing'/exp OR

'inflammation of the lungs'/exp OR 'interstitial lung disease'/exp OR 'atrophic rhinitis'/exp OR 'paranasal sinusitis'/exp OR 'Vertigo'/exp OR 'Meniere disease'/exp OR 'benign paroxysmal positional vertigo'/exp OR 'nose polyp'/exp OR 'otosclerosis'/exp OR 'colon polyp'/exp OR 'stomach diverticulosis'/exp OR 'atrophic gastritis'/exp OR 'gastroesophageal reflux'/exp OR 'hiatus hernia'/exp OR 'irritable colon'/exp OR 'Celiac disease'/exp OR 'ulcerative colitis'/exp OR 'Crohn Disease'/exp OR 'Liver Cirrhosis'/exp OR 'osteoarthritis'/exp OR 'backache'/exp OR 'neck pain'/exp OR 'low back pain'/exp OR 'intervertebral disk hernia'/exp OR 'sciatica'/exp OR 'rheumatoid arthritis'/exp OR 'axial spondyloarthritis'/exp OR 'psoriatic arthritis'/exp OR 'Paget bone disease'/exp OR 'tendinitis'/exp OR 'Periarthritis'/exp OR 'epicondylitis'/exp OR 'Bursitis'/exp OR 'fibromyalgia'/exp OR 'chronic fatigue syndrome'/exp OR 'crystal arthropathy'/exp OR 'Scoliosis'/exp OR 'complex regional pain syndrome type I'/exp OR 'Headache Disorders'/exp OR 'multiple sclerosis'/exp OR 'Epilepsy'/exp OR 'Alzheimer disease'/exp OR 'multiinfarct dementia'/exp OR 'Parkinson disease'/exp OR 'Guillain Barre syndrome'/exp OR 'cerebrovascular accident'/exp OR 'transient ischemic attack'/exp OR 'muscular dystrophy'/exp OR 'myasthenia gravis'/exp OR 'Goodpasture syndrom'/exp OR 'kidney polycystic disease'/exp OR 'urine incontinence'/exp OR 'Leiomyoma'/exp OR 'nephrolithiasis'/exp OR 'acne vulgaris'/exp OR 'rosacea'/exp OR 'eczema'/exp OR 'urticaria'/exp OR 'Psoriasis'/exp OR 'Vitiligo'/exp OR 'alopecia'/exp OR 'suppurative hidradenitis'/exp OR 'linear iga bullous dermatosis'/exp OR 'herpes simplex'/exp OR 'Macular Degeneration'/exp OR 'Low Tension Glaucoma'/exp OR 'Cataract'/exp OR 'retina disease'/exp OR 'vitreous body detachment'/exp OR 'visual disorder'/exp OR 'uveitis'/exp OR 'Scleritis'/exp OR 'systemic lupus erythematosus'/exp OR 'antiphospholipid syndrome'/exp OR 'Sjoegren syndrome'/exp OR 'systemic sclerosis'/exp OR 'Wegener granulomatosis'/exp OR 'microscopic polyangiitis'/exp OR 'Churg Strauss syndrome'/exp OR 'cryoglobulinemia'/exp OR 'polyarteritis nodosa'/exp OR 'giant cell arteritis'/exp OR 'aortic arch syndrome'/exp OR 'Behcet disease'/exp OR 'sarcoidosis'/exp OR 'Ehlers Danlos syndrome'/exp OR 'tuberculosis'/exp OR 'acquired immune deficiency syndrome'/exp OR 'chronic hepatitis B'/exp OR 'chronic hepatitis C'/exp OR 'Lyme Disease'/exp OR 'Endocarditis'/exp OR 'arthritis'/exp OR 'diskitis'/exp OR 'acute leukemia'/exp OR 'Hodgkin disease'/exp OR 'diffuse large B cell lymphoma'/exp OR 'prolymphocytic leukemia'/exp OR 'leukemia'/exp OR 'chronic myeloid leukemia'/exp OR 'myeloma'/exp OR 'Down syndrome'/exp OR 'myelodysplastic syndrome'/exp OR 'bone marrow suppression'/exp OR 'hemolytic anemia'/exp OR 'pernicious anemia'/exp OR 'thrombocytopenic purpura'/exp OR 'thrombotic microangiopathy'/exp OR 'hemochromatosis'/exp OR 'sickle cell anemia'/exp OR 'porphyria'/exp OR 'hemophilia A'/exp OR 'chronic fatigue syndrome'/exp OR 'chronic pain'/exp OR 'Basal Cell Epithelioma':ti,ab OR 'Basal Cell Epitheliomas':ti,ab OR 'Brain Metastase':ti,ab OR 'Brain Metastases':ti,ab OR 'cerebroma':ti,ab OR 'Colon Adenocarcinoma':ti,ab OR 'Colon Adenocarcinomas':ti,ab OR 'dermatoma':ti,ab OR 'encephalophyma':ti,ab OR 'hypophysoma':ti,ab OR 'kidney mass':ti,ab OR 'kidney neoplastic mass':ti,ab OR 'malignant melanomatosis':ti,ab OR 'melanocarcinoma':ti,ab OR 'Melanoma':ti,ab OR 'melanomalignoma':ti,ab OR 'Melanomas':ti,ab OR 'naevocarcinoma':ti,ab OR 'neoplastic lung':ti,ab OR 'neoplastic mammary gland':ti,ab OR 'nephroma':ti,ab OR 'nevocarcinoma':ti,ab OR 'Pituitary Adenoma':ti,ab OR 'Pituitary Adenomas':ti,ab OR 'rectal mass':ti,ab OR 'rectum mass':ti,ab OR 'renal mass':ti,ab OR 'renal neoplastic mass':ti,ab OR 'reninoma':ti,ab OR 'Rodent Ulcer':ti,ab OR 'Rodent Ulcers':ti,ab OR 'squamous cell epithelioma':ti,ab OR 'squamous epithelioma':ti,ab OR 'Thyroid Adenoma':ti,ab OR 'Thyroid Adenomas':ti,ab OR 'angina':ti,ab OR 'anginal attack':ti,ab OR 'angiocardiopathy':ti,ab OR 'angiocardiovascular disease':ti,ab OR 'angiocardiovascular diseases':ti,ab OR 'Angor Pectori':ti,ab OR 'Aortic Valve Diseases':ti,ab OR 'Aortic Valve Disorder':ti,ab OR 'Aortic Valve Disorders':ti,ab OR 'Arrhythmia':ti,ab OR 'Arrythmia':ti,ab OR 'Artificial Cardiac Pacemaker':ti,ab OR 'Artificial Cardiac Pacemakers':ti,ab OR 'Artificial Pacemaker':ti,ab OR 'Artificial Pacemakers':ti,ab OR 'Atrial Fibrillation':ti,ab OR 'Atrial Fibrillations':ti,ab OR 'Auricular Fibrillation':ti,ab OR 'Auricular Fibrillations':ti,ab OR 'backward failure

heart':ti,ab OR 'backward failure, heart':ti,ab OR 'Cardiac Arrest':ti,ab OR 'Cardiac Arrhythmias':ti,ab OR 'cardiac backward failure':ti,ab OR 'cardiac conduction system disease':ti,ab OR 'cardiac decompensation':ti,ab OR 'Cardiac Disorders':ti,ab OR 'cardiac disrhythmia':ti,ab OR 'Cardiac Dysrhythmia':ti,ab OR 'cardiac failure':ti,ab OR 'cardiac failures':ti,ab OR 'cardiac incompetence':ti,ab OR 'cardiac incompetences':ti,ab OR 'cardiac infarct':ti,ab OR 'cardiac insufficien*':ti,ab OR 'cardiac stand still':ti,ab OR 'cardiac valve defect':ti,ab OR 'cardiac valve disease':ti,ab OR 'cardial decompensation':ti,ab OR 'cardial infarct':ti,ab OR 'cardial insufficiency':ti,ab OR 'cardial valve disease':ti,ab OR 'cardiovascular complication*':ti,ab OR 'cardiovascular decompensation':ti,ab OR 'cardiovascular disease':ti,ab OR 'cardiovascular diseases':ti,ab OR 'cardiovascular disorder':ti,ab OR 'cardiovascular disorders':ti,ab OR 'cardiovascular disturbance':ti,ab OR 'cardiovascular disturbances':ti,ab OR 'cardiovascular disturbancescardiovascular lesion*':ti,ab OR 'cardiovascular failure':ti,ab OR 'cardiovascular failures':ti,ab OR 'cardiovascular incompetence':ti,ab OR 'cardiovascular incompetences':ti,ab OR 'cardiovascular insufficien*':ti,ab OR 'cardiovascular lesion*':ti,ab OR 'cardiovascular syndrome':ti,ab OR 'cardiovascular syndromes':ti,ab OR 'cardiovascular vegetative disorder':ti,ab OR 'cardiovascular vegetative disorders':ti,ab OR 'cholesteremia':ti,ab OR 'cholesterinemia':ti,ab OR 'cholesterolemia':ti,ab OR 'Circulatory Disorders':ti,ab OR 'complication cardiovascular':ti,ab OR 'conduction defect':ti,ab OR 'conduction defects':ti,ab OR 'conduction disease':ti,ab OR 'conduction diseases':ti,ab OR 'conduction disorder':ti,ab OR 'conduction disorders':ti,ab OR 'conduction disturbance':ti,ab OR 'conduction disturbances':ti,ab OR 'Coronary Disorders':ti,ab OR 'decompensatio cordis':ti,ab OR 'decompensation, heart':ti,ab OR 'ectopic heart rhythm':ti,ab OR 'ectopic rhythm':ti,ab OR 'Elevated Cholesterol':ti,ab OR 'Elevated Cholesterols':ti,ab OR 'heart aberrant conduction':ti,ab OR 'heart arrhytmia':ti,ab OR 'Heart Attack':ti,ab OR 'Heart Attacks':ti,ab OR 'heart backward failure':ti,ab OR 'heart decompensation':ti,ab OR 'heart disease':ti,ab OR 'heart diseases':ti,ab OR 'heart disorder':ti,ab OR 'heart disorders':ti,ab OR 'heart dysrhythmia':ti,ab OR 'heart ectopic beat':ti,ab OR 'heart ectopic ventricle contraction':ti,ab OR 'heart failure':ti,ab OR 'heart failures':ti,ab OR 'heart incompetence':ti,ab OR 'heart infarct':ti,ab OR 'heart insufficien*':ti,ab OR 'heart rhythm disorder':ti,ab OR 'heart valve abnormalities':ti,ab OR 'heart valve abnormality':ti,ab OR 'heart valve defect':ti,ab OR 'heart valve degeneration':ti,ab OR 'Heart Valve Disease':ti,ab OR 'heart valve diseases':ti,ab OR 'heart valve lesion':ti,ab OR 'Heart Valvular Disease':ti,ab OR 'Heart Valvular Diseases':ti,ab OR 'High Blood Pressure':ti,ab OR 'High Blood Pressures':ti,ab OR 'High Cholesterol Level':ti,ab OR 'High Cholesterol Levels':ti,ab OR 'Hypercholesteremia':ti,ab OR 'Hypercholesteremias':ti,ab OR 'hypercholesterinaemia':ti,ab OR 'hypercholesterinemia':ti,ab OR 'hypercholesterolaemia':ti,ab OR 'Hypercholesterolemias':ti,ab OR 'hypertensive disease':ti,ab OR 'hypertensive effect':ti,ab OR 'hypertensive pulmonary vascular disease':ti,ab OR 'hypertensive response':ti,ab OR 'insufficientia cardis':ti,ab OR 'insufficientio cardis':ti,ab OR 'lung embolism':ti,ab OR 'lung embolization':ti,ab OR 'lung embolus':ti,ab OR 'lung emboly':ti,ab OR 'lung microembolism':ti,ab OR 'lung microembolization':ti,ab OR 'lung microembolus':ti,ab OR 'lung thromboembolism':ti,ab OR 'major adverse cardiovascular event*':ti,ab OR 'Myocardial Failure':ti,ab OR 'Myocardial Infarct':ti,ab OR 'myocardial insufficiency':ti,ab OR 'Myocardial Infarcts':ti,ab OR 'myocardium infarct':ti,ab OR 'Periphlebitides':ti,ab OR 'Periphlebitis':ti,ab OR 'perivenous infection':ti,ab OR 'Phlebitides':ti,ab OR 'phlebitis superficialis':ti,ab OR 'post phlebitis syndrome':ti,ab OR 'postphlebitic disease':ti,ab OR 'postphlebitis syndrome':ti,ab OR 'Pulmonary Embolism':ti,ab OR 'Pulmonary Embolisms':ti,ab OR 'pulmonary embolization':ti,ab OR 'pulmonary embolus':ti,ab OR 'pulmonary hypertensive diseases':ti,ab OR 'pulmonary hypertensive disorder':ti,ab OR 'pulmonary hypertensive disorders':ti,ab OR 'pulmonary microembolism':ti,ab OR 'pulmonary thromboembolic disease':ti,ab OR 'Pulmonary Thromboembolism':ti,ab OR 'Pulmonary Thromboembolisms':ti,ab OR 'Raynauds Disease':ti,ab OR 'Stenocardia':ti,ab OR 'Stenocardias':ti,ab

OR 'superficial phlebitis':ti,ab OR 'valvulopathies':ti,ab OR 'valvulopathy':ti,ab OR 'Vascular Disorders':ti,ab OR 'Vasculitis':ti,ab OR 'vein inflammation':ti,ab OR 'venitis':ti,ab OR 'venous inflammation':ti,ab OR 'vitium cordis':ti,ab OR 'Chronic Infectious Mononucleosis-Like Syndromes':ti,ab OR 'chronic intractable pain':ti,ab OR 'Royal Free Diseases':ti,ab OR 'Systemic Exertion Intolerance Diseases':ti,ab OR 'Chronic Pain':ti,ab OR 'Chronic Pains':ti,ab OR 'Achalasia':ti,ab OR 'Achalasias':ti,ab OR 'alcohol liver injury':ti,ab OR 'alcoholic liver':ti,ab OR 'Atrophic Gastritides':ti,ab OR 'Atrophic Gastritis':ti,ab OR 'autoimmune gastritis':ti,ab OR 'bowel syndrome':ti,ab OR 'cardiac herniation':ti,ab OR 'cardioesophageal reflux':ti,ab OR 'cardiooesophageal reflux':ti,ab OR 'Cardiospasm':ti,ab OR 'Cardiospasms':ti,ab OR 'Celiac Disease':ti,ab OR 'celiac syndrome':ti,ab OR 'chronic inflammatory bowel diseases':ti,ab OR 'chronic ulceration colon':ti,ab OR 'cirrhosis':ti,ab OR 'cleron disease':ti,ab OR 'coeliac disease':ti,ab OR 'coeliac syndrome':ti,ab OR 'coeliaky':ti,ab OR 'Colitis Gravis':ti,ab OR 'colitis ulcerativa':ti,ab OR 'colitis ulcerosa':ti,ab OR 'colon disease':ti,ab OR 'colon diseases':ti,ab OR 'colon disorder':ti,ab OR 'colon disorders':ti,ab OR 'colon juvenile polyp':ti,ab OR 'colon polypoid lesion':ti,ab OR 'colon spasm':ti,ab OR 'chronic colon ulceration':ti,ab OR 'Colonic Polyp':ti,ab OR 'Colonic Polyps':ti,ab OR 'colonospasm':ti,ab OR 'Crohns Disease':ti,ab OR 'Crohn`s Disease':ti,ab OR 'Crohn`s Enteritis':ti,ab OR 'digestive disease':ti,ab OR 'digestive diseases':ti,ab OR 'digestive disorder':ti,ab OR 'digestive disorders':ti,ab OR 'digestive system disorders':ti,ab OR 'enteritis regionalis':ti,ab OR 'Esophageal Hernia*':ti,ab OR 'Esophageal Reflux':ti,ab OR 'esophageal regurgitation':ti,ab OR 'esophagogastric reflux':ti,ab OR 'esophagus hernia':ti,ab OR 'esophagus reflux':ti,ab OR 'functional colonic diseases':ti,ab OR 'Gastric Acid Reflux':ti,ab OR 'gastric atrophy':ti,ab OR 'gastric regurgitation':ti,ab OR 'Gastroduodenal Ulcer':ti,ab OR 'Gastroduodenal Ulcers':ti,ab OR 'gastroesophageal reflex':ti,ab OR 'gastroesophageal regurgitation':ti,ab OR 'gastroesophagus reflux':ti,ab OR 'gastrointestinal disease':ti,ab OR 'gastrointestinal diseases':ti,ab OR 'gastrointestinal disorder':ti,ab OR 'gastrointestinal disorders':ti,ab OR 'gastrointestinal dysfunction*':ti,ab OR 'gastrooesophageal reflex':ti,ab OR 'gastrooesophageal reflux':ti,ab OR 'Gastro-oesophageal Reflux':ti,ab OR 'gastrooesophageal regurgitation':ti,ab OR 'gee herter disease':ti,ab OR 'gee thaysen disease':ti,ab OR 'GERD':ti,ab OR 'Gluten Enteropathies':ti,ab OR 'Gluten Enteropathy':ti,ab OR 'gluten induced enteropathy':ti,ab OR 'gluten intolerance':ti,ab OR 'Gluten Sensitive Enteropathy':ti,ab OR 'Gluten-Sensitive Enteropathies':ti,ab OR 'Gluten-Sensitive Enteropathy':ti,ab OR 'Granulomatous Colitis':ti,ab OR 'Granulomatous Enteritis':ti,ab OR 'hernia hiatus esophagi':ti,ab OR 'hiatal diaphragmatic hernia':ti,ab OR 'Hiatal Hernia*':ti,ab OR 'hiatus diaphragmatic hernia':ti,ab OR 'hiatus esophagi hernia':ti,ab OR 'Hiatus Hernia*':ti,ab OR 'hiatus oesophageal hernia':ti,ab OR 'hiatus oesophagus hernia':ti,ab OR 'huebner herter disease':ti,ab OR 'Idiopathic Proctocolitis':ti,ab OR 'Ileocolitis':ti,ab OR 'Inflammatory Bowel Disease':ti,ab OR 'intestinal infantilism':ti,ab OR 'Irritable Bowel Syndromes':ti,ab OR 'Irritable Colon':ti,ab OR 'Liver Fibrosis':ti,ab OR 'Marginal Ulcer':ti,ab OR 'Marginal Ulcers':ti,ab OR 'Megaesophagus':ti,ab OR 'morbus crohn':ti,ab OR 'mucomembraneous colitis':ti,ab OR 'mucomembranous colitis':ti,ab OR 'mucosal colitis':ti,ab OR 'Mucous Colitides':ti,ab OR 'Mucous Colitis':ti,ab OR 'oesophageal reflux':ti,ab OR 'oesophageal regurgitation':ti,ab OR 'oesophagogastric reflux':ti,ab OR 'oesophagus reflux':ti,ab OR 'Paraesophageal Hernia':ti,ab OR 'para-esophageal hernia':ti,ab OR 'Paraesophageal Hernias':ti,ab OR 'paraesophageal herniation':ti,ab OR 'para-esophageal herniation':ti,ab OR 'paraoesophageal hernia':ti,ab OR 'para-oesophageal hernia':ti,ab OR 'paraoesophageal herniation':ti,ab OR 'para-oesophageal herniation':ti,ab OR 'Peptic Ulcers':ti,ab OR 'Regional Enteritis':ti,ab OR 'regional enterocolitis':ti,ab OR 'Regional Ileitides':ti,ab OR 'Regional Ileitis':ti,ab OR 'regurgitation, gastroesophageal':ti,ab OR 'spastic colitis':ti,ab OR 'spastic colon':ti,ab OR 'Sprue':ti,ab OR 'stomach hernia':ti,ab OR 'Terminal Ileitis':ti,ab OR 'Ulcerative Colitis':ti,ab OR 'ulcerative colorectitis':ti,ab OR 'ulcerative procto colitis':ti,ab OR 'ulcerative proctocolitis':ti,ab OR 'ulcerous colitis':ti,ab OR 'unstable colon':ti,ab OR 'acromegalia':ti,ab OR

'Acromegalies':ti,ab OR 'acromegalism':ti,ab OR 'Acromegaly':ti,ab OR 'Addison Disease':ti,ab OR 'Addisons Disease':ti,ab OR 'Addison`s Disease':ti,ab OR 'adipose tissue hyperplasia':ti,ab OR 'adipositas':ti,ab OR 'adiposity':ti,ab OR 'adrenal cortex hyperplasia':ti,ab OR 'adrenal cortical hyperplasia':ti,ab OR 'adrenal failure':ti,ab OR 'adrenal failures':ti,ab OR 'adrenal gland disorders':ti,ab OR 'adrenocortical hyperplasia':ti,ab OR 'adrenocorticohyperplasia':ti,ab OR 'akromegalia':ti,ab OR 'Aldosteronism':ti,ab OR 'antidiuretic hormone insufficiency':ti,ab OR 'arenocortical hyperplasia':ti,ab OR 'Basedow Disease':ti,ab OR 'basedow syndrome':ti,ab OR 'Basedows Disease':ti,ab OR 'Basedow`s Disease':ti,ab OR 'chromaffin paraganglioma':ti,ab OR 'chronic autoimmune thyroiditis':ti,ab OR 'Chronic Lymphocytic Thyroiditides':ti,ab OR 'Chronic Lymphocytic Thyroiditis':ti,ab OR 'Conn Syndrome':ti,ab OR 'Conns Syndrome':ti,ab OR 'Conn`s Syndrome':ti,ab OR 'corpulency':ti,ab OR 'Cushing syndrome':ti,ab OR 'Cushings syndrome':ti,ab OR 'Cushing`s Syndrome':ti,ab OR 'diabetes':ti,ab OR 'diabetic':ti,ab OR 'endocrinal disease':ti,ab OR 'endocrinal diseases':ti,ab OR 'endocrinal disorder':ti,ab OR 'endocrinal disorders':ti,ab OR 'endocrinal disturbance':ti,ab OR 'endocrinal disturbances':ti,ab OR 'endocrinal dysfunction*':ti,ab OR 'endocrine disease':ti,ab OR 'endocrine diseases':ti,ab OR 'endocrine disorder':ti,ab OR 'Endocrine Disorders':ti,ab OR 'endocrine disturbance':ti,ab OR 'endocrine disturbances':ti,ab OR 'endocrine dysfunction*':ti,ab OR 'endocrine gland disease':ti,ab OR 'endocrine gland diseases':ti,ab OR 'endocrine gland disorder':ti,ab OR 'endocrine gland disorders':ti,ab OR 'endocrine gland dysfunction*':ti,ab OR 'endocrine syndrome':ti,ab OR 'endocrine syndromes':ti,ab OR 'endocrine system disease':ti,ab OR 'endocrine system diseases':ti,ab OR 'endocrine system disorder':ti,ab OR 'endocrine system disorders':ti,ab OR 'endocrine system dysfunction*':ti,ab OR 'endocrinological disease':ti,ab OR 'endocrinological diseases':ti,ab OR 'endocrinological disorder':ti,ab OR 'endocrinological disorders':ti,ab OR 'endocrinological dysfunction*':ti,ab OR 'excess body weight':ti,ab OR 'Exophthalmic Goiter':ti,ab OR 'Exophthalmic Goiters':ti,ab OR 'exophthalmic goitre':ti,ab OR 'exophthalmic hyperthyroidism':ti,ab OR 'Extra Adrenal Pheochromocytoma':ti,ab OR 'Extra-Adrenal Pheochromocytoma':ti,ab OR 'familial hypoadrenocorticism':ti,ab OR 'fat overload syndrome':ti,ab OR 'feline hyperthyroidism':ti,ab OR 'Graves Disease':ti,ab OR 'Graves` Disease':ti,ab OR 'Graves hyperthyroidism':ti,ab OR 'graves syndrome':ti,ab OR 'Graves`s disease':ti,ab OR 'Hashimoto Disease':ti,ab OR 'hashimoto goiter':ti,ab OR 'Hashimoto Struma':ti,ab OR 'Hashimoto Syndrome':ti,ab OR 'Hashimoto Thyroiditides':ti,ab OR 'Hashimoto Thyroiditis':ti,ab OR 'hashimoto thyroidosis':ti,ab OR 'Hashimotos Disease':ti,ab OR 'Hashimoto`s Disease':ti,ab OR 'Hashimoto`s Struma':ti,ab OR 'Hashimotos Syndrome':ti,ab OR 'Hashimoto`s Syndrome':ti,ab OR 'Hashimoto`s Syndromes':ti,ab OR 'Hashimotos thyroiditis':ti,ab OR 'Hashimoto`s thyroiditis':ti,ab OR 'hormonal disease':ti,ab OR 'hormonal diseases':ti,ab OR 'hormonal disorder':ti,ab OR 'hormonal disorders':ti,ab OR 'hormonal dysfunction*':ti,ab OR 'hormone imbalance':ti,ab OR 'hyperaldosterone':ti,ab OR 'Hypercortisolism':ti,ab OR 'hypermineralocorticidism':ti,ab OR 'hypermineralocorticism':ti,ab OR 'Hyperprolactinaemia*':ti,ab OR 'Hyperprolactinemia':ti,ab OR 'Hyperprolactinemias':ti,ab OR 'hyperthyreoidism':ti,ab OR 'hyperthyreosis':ti,ab OR 'Hyperthyroid':ti,ab OR 'hyperthyroidea':ti,ab OR 'hyperthyroidosis':ti,ab OR 'Hyperthyroids':ti,ab OR 'hypogonadism':ti,ab OR 'hypothyreoidism':ti,ab OR 'hypothyreosis':ti,ab OR 'hypothyroidea':ti,ab OR 'hypothyroidism':ti,ab OR 'Hypothyroidisms':ti,ab OR 'hypothyroidosis':ti,ab OR 'hypothyrosis':ti,ab OR 'Inappropriate GH Secretion Syndrome':ti,ab OR 'Inappropriate GH Secretion Syndromes':ti,ab OR 'Inappropriate Growth Hormone Secretion Syndrome':ti,ab OR 'Inappropriate Growth Hormone Secretion Syndromes':ti,ab OR 'Inappropriate Prolactin Secretion':ti,ab OR 'Inappropriate Secretion Prolactin':ti,ab OR 'lymphadenoid goiter':ti,ab OR 'megalakria':ti,ab OR 'mineralcorticoid excess syndrome':ti,ab OR 'obesitas':ti,ab OR 'obesity':ti,ab OR 'overweight':ti,ab OR 'phaeochromoblastoma':ti,ab OR 'phaeochromocytoma':ti,ab OR 'pheochromoblastoma':ti,ab OR 'Pheochromocytomas':ti,ab OR 'pheochromocytomata':ti,ab OR 'pheochromocytomatosis':ti,ab OR

'pheochromocytosis':ti,ab OR 'polycystic ovary syndrome':ti,ab OR 'polycystic ovary syndromes':ti,ab OR 'Primary Adrenal Insufficiency':ti,ab OR 'Primary Adrenocortical Insufficiencies':ti,ab OR 'Primary Adrenocortical Insufficiency':ti,ab OR 'Primary Hyperaldosteronism':ti,ab OR 'Primary Hyperthyroidism':ti,ab OR 'Primary Hypoadrenalism':ti,ab OR 'Primary Hypoadrenalisms':ti,ab OR 'Prolactin Hypersecretion Syndrome':ti,ab OR 'Prolactin Hypersecretion Syndromes':ti,ab OR 'Somatotropin Hypersecretion Syndrome':ti,ab OR 'Somatotropin Hypersecretion Syndromes':ti,ab OR 'struma hashimoto':ti,ab OR 'thyroid deficiency':ti,ab OR 'thyroid gland failure':ti,ab OR 'thyroid gland hyperfunction':ti,ab OR 'thyroid hyperfunction':ti,ab OR 'thyroid insufficiency':ti,ab OR 'Thyroid Stimulating Hormone Deficiency':ti,ab OR 'thyroideal hyperfunction':ti,ab OR 'Thyroid-Stimulating Hormone Deficiencies':ti,ab OR 'Thyroid-Stimulating Hormone Deficiency':ti,ab OR 'TSH Deficiencies':ti,ab OR 'TSH Deficiency':ti,ab OR 'Vasopressin Deficiency':ti,ab OR 'angioleiomyoma':ti,ab OR 'angiomyoma':ti,ab OR 'elastomyofibroma':ti,ab OR 'fibromyoma':ti,ab OR 'fibromyomas':ti,ab OR 'hemangioleiomyoma':ti,ab OR 'hemangiomyoma':ti,ab OR 'Impotence':ti,ab OR 'leimyoma':ti,ab OR 'Leiomyomas':ti,ab OR 'leyomyoma':ti,ab OR 'myofibroma':ti,ab OR 'myofibromatosis':ti,ab OR 'vascular leiomyoma':ti,ab OR 'addison anaemia':ti,ab OR 'addison anemia':ti,ab OR 'addisonian anaemia':ti,ab OR 'addisonian anemia':ti,ab OR 'ahf deficiency':ti,ab OR 'ahg deficiency':ti,ab OR 'anaemia perniciosa':ti,ab OR 'anemia perniciosa':ti,ab OR 'B12 deficiency anaemia':ti,ab OR 'B12 deficiency anemia':ti,ab OR 'B12 deficient anaemia':ti,ab OR 'B12 deficient anemia':ti,ab OR 'B12 vitamin deficiency anemia':ti,ab OR 'biermer anaemia':ti,ab OR 'biermer anemia':ti,ab OR 'biermer disease':ti,ab OR 'blood disease':ti,ab OR 'blood diseases':ti,ab OR 'blood disorder':ti,ab OR 'blood disorders':ti,ab OR 'blood dysfunction*':ti,ab OR 'classic haemophilia':ti,ab OR 'cobalamin deficiency anaemia':ti,ab OR 'cobalamin deficiency anemia':ti,ab OR 'congenital antihaemophilic factor deficiency':ti,ab OR 'congenital antihaemophilic globulin deficiency':ti,ab OR 'congenital antihemophilic factor deficiency':ti,ab OR 'congenital antihemophilic globulin deficiency':ti,ab OR 'drepanocytemia':ti,ab OR 'drepanocytic anaemia':ti,ab OR 'drepanocytic anemia':ti,ab OR 'drepanocytosis':ti,ab OR 'haematologic disease':ti,ab OR 'haematologic diseases':ti,ab OR 'haematologic disorder':ti,ab OR 'haematologic disorders':ti,ab OR 'haematologic dysfunction*':ti,ab OR 'haematological disease':ti,ab OR 'haematological diseases':ti,ab OR 'haematological disorder':ti,ab OR 'haematological disorders':ti,ab OR 'haematological dysfunction*':ti,ab OR 'haemochromatosis':ti,ab OR 'haemoglobin SS':ti,ab OR 'haemolytic anaemia':ti,ab OR 'haemolytic disease':ti,ab OR 'haemolytic syndrome':ti,ab OR 'haemophilia a':ti,ab OR 'haemophilia vera':ti,ab OR 'Hb SS disease':ti,ab OR 'heart hemochromatosis':ti,ab OR 'hemachromatosis':ti,ab OR 'hematologic disease':ti,ab OR 'hematologic diseases':ti,ab OR 'hematologic disorder':ti,ab OR 'hematologic disorders':ti,ab OR 'hematologic dysfunction*':ti,ab OR 'hematopathy':ti,ab OR 'hemoglobin SS':ti,ab OR 'hemolytic anemia':ti,ab OR 'hemolytic disease':ti,ab OR 'hemolytic syndrome':ti,ab OR 'hemopathie':ti,ab OR 'hemopathies':ti,ab OR 'hemopathy':ti,ab OR 'hemophylia type a':ti,ab OR 'hereditary iron overload':ti,ab OR 'idiopathic hemochromatosis':ti,ab OR 'intermittent porphyria':ti,ab OR 'iron overload disease':ti,ab OR 'iron overload disorder':ti,ab OR 'iron overload syndrome':ti,ab OR 'latent porphyria':ti,ab OR 'lymphatic disease':ti,ab OR 'lymphatic diseases':ti,ab OR 'lymphatic disorder':ti,ab OR 'lymphatic disorders':ti,ab OR 'lymphatic dysfunction*':ti,ab OR 'macrocytic hyperchromic anaemia':ti,ab OR 'macrocytic hyperchromic anemia':ti,ab OR 'meniscocytosis':ti,ab OR 'pernicious anaemia':ti,ab OR 'pernicious anemia':ti,ab OR 'porphyrias':ti,ab OR 'porphyric disease':ti,ab OR 'primary anaemia':ti,ab OR 'primary anemia':ti,ab OR 'primary congenital hemochromatosis':ti,ab OR 'recklinghausen applebaum disease':ti,ab OR 'sickle anaemia':ti,ab OR 'sickle anemia':ti,ab OR 'sickle cell anaemia':ti,ab OR 'siderochromatosis':ti,ab OR 'sulfhemoglobinem*':ti,ab OR 'sulphaemoglobinaem*':ti,ab OR 'systemic porphyria':ti,ab OR 'thrombocytopaenia purpura':ti,ab OR 'thrombocytopenia purpura':ti,ab OR 'thrombotic purpura':ti,ab OR 'true haemophilia':ti,ab OR 'vitamin b 12 deficiency anaemia':ti,ab OR

'vitamin b 12 deficiency anemia':ti,ab OR 'a2 myeloma':ti,ab OR 'Acquired Hemolytic Anemias':ti,ab OR 'Acquired Immune Deficiency Syndrome Virus':ti,ab OR 'acquired immunodeficiency':ti,ab OR 'Addison Anemias':ti,ab OR 'Addisons Anemia':ti,ab OR 'Addison`s Anemias':ti,ab OR 'AIDS':ti,ab OR 'aleukaemia':ti,ab OR 'aleukemia':ti,ab OR 'Arthriti*':ti,ab OR 'arthrochondritis':ti,ab OR 'arthrosynovitis':ti,ab OR 'B. burgdorferi Infection*':ti,ab OR 'bone marrow dysplasia':ti,ab OR 'Bone Marrow Fibros*':ti,ab OR 'Borrelia burgdorferi Infection*':ti,ab OR 'Bronze Diabete':ti,ab OR 'Bronzed Cirrhoses':ti,ab OR 'Chronic Hepatit*':ti,ab OR 'chronic myeloleukaemia':ti,ab OR 'chronic myeloleukemia':ti,ab OR 'Discitides':ti,ab OR 'discitis':ti,ab OR 'disk space infection':ti,ab OR 'Diskiti*':ti,ab OR 'DLBCL':ti,ab OR 'down disease':ti,ab OR 'Downs syndrome':ti,ab OR 'Down`s syndrome':ti,ab OR 'Dysmyelopoietic Syndrome*':ti,ab OR 'endocardial inflammation':ti,ab OR 'Endocarditi*':ti,ab OR 'endo-carditis':ti,ab OR 'endocardium inflammation':ti,ab OR 'Erythremia*':ti,ab OR 'Factor 8 Deficienc*':ti,ab OR 'Factor VIII Deficienc*':ti,ab OR 'Factor VIII defiency':ti,ab OR 'Familial Hemochromatoses':ti,ab OR 'Familial Hemochromatosis':ti,ab OR 'Haemochromato':ti,ab OR 'Haemochromatos':ti,ab OR 'Haemolytic Anaemias':ti,ab OR 'HbS Disease':ti,ab OR 'Hematopoetic Myelodysplasias':ti,ab OR 'hemoblastoma':ti,ab OR 'Hemochromato':ti,ab OR 'Hemochromatos':ti,ab OR 'Hemoglobin S Disease':ti,ab OR 'Hemoglobin S Diseases':ti,ab OR 'Hemophilia':ti,ab OR 'hemophilias':ti,ab OR 'high-risk MDS':ti,ab OR 'Hodgkin disease*':ti,ab OR 'Hodgkin Granuloma*':ti,ab OR 'hodgkin sclerosis':ti,ab OR 'hodgkin`s disease':ti,ab OR 'Hodgkins Disease*':ti,ab OR 'Hodgkins Granuloma':ti,ab OR 'Hodgkin`s Granuloma':ti,ab OR 'HTLV-III':ti,ab OR 'Human Immunodeficiency Virus*':ti,ab OR 'Human T Cell Lymphotropic Virus Type III':ti,ab OR 'Human T Lymphotropic Virus Type III':ti,ab OR 'Human T-Cell Lymphotropic Virus Type III':ti,ab OR 'Human T-Lymphotropic Virus Type III':ti,ab OR 'infection by B. burgdorferi':ti,ab OR 'infection by Borrelia burgdorferi':ti,ab OR 'intervertebral disc infection':ti,ab OR 'intervertebral disk infection':ti,ab OR 'intervertebral disk inflammation':ti,ab OR 'Iron Storage Disorder*':ti,ab OR 'joint inflammation':ti,ab OR 'Koch Disease*':ti,ab OR 'Kochs Disease*':ti,ab OR 'Koch`s Disease*':ti,ab OR 'langdon down syndrome':ti,ab OR 'LAV-HTLV-III':ti,ab OR 'leucaemia':ti,ab OR 'leucemia':ti,ab OR 'Leucocythaemia*':ti,ab OR 'Leucocythemia*':ti,ab OR 'leukaemia':ti,ab OR 'Leukemia*':ti,ab OR 'Lyme borrelioses':ti,ab OR 'Lyme Borreliosis':ti,ab OR 'Lyme`s borrelioses':ti,ab OR 'Lyme`s borreliosis':ti,ab OR 'Lymes disease':ti,ab OR 'Lyme`s disease':ti,ab OR 'Lymphadenopathy Associated Virus*':ti,ab OR 'Lymphadenopathy-Associated Virus*':ti,ab OR 'lymphogranuloma maligne':ti,ab OR 'lymphogranuloma malignum':ti,ab OR 'lymphogranulomatosis':ti,ab OR 'Malignant Granuloma*':ti,ab OR 'Malignant Lymphogranuloma':ti,ab OR 'Malignant Lymphogranulomas':ti,ab OR 'Microangiopathic Anemias':ti,ab OR 'Microangiopathic Hemolytic Anemias':ti,ab OR 'mongolian idiocy':ti,ab OR 'mongolism':ti,ab OR 'mongoloid idiocy':ti,ab OR 'mongoloidism':ti,ab OR 'morbus hodgkin':ti,ab OR 'myelodysplasia':ti,ab OR 'myelodysplastic disease':ti,ab OR 'myelodysplastic disorder':ti,ab OR 'Myelodysplastic Syndrome*':ti,ab OR 'Myelofibros*':ti,ab OR 'Myeloid Metaplasia*':ti,ab OR 'myeloplaxoma':ti,ab OR 'Myeloscleros*':ti,ab OR 'Nonleukemic Myelos*':ti,ab OR 'oligoarthritis':ti,ab OR 'Osler Vaquez Disease*':ti,ab OR 'Osler-Vaquez Disease*':ti,ab OR 'Pernicious Anemias':ti,ab OR 'Pigmentary Cirrhos*':ti,ab OR 'Polyarthrit*':ti,ab OR 'Polycythemia Ruba Vera*':ti,ab OR 'Polycythemia Rubra Vera*':ti,ab OR 'Primary Hemochromatosis':ti,ab OR 'Primary Polycythemia*':ti,ab OR 'reed sternberg disease':ti,ab OR 'Sickle Cell Anemia':ti,ab OR 'Sickle Cell Anemias':ti,ab OR 'Sickle Cell Disease':ti,ab OR 'Sickle Cell Diseases':ti,ab OR 'sickle cell disorder':ti,ab OR 'Sickle Cell Disorders':ti,ab OR 'Sickling Disorder Due to Hemoglobin S':ti,ab OR 'Spondylodisciti*':ti,ab OR 'Spondylodiskiti*':ti,ab OR 'TB disease':ti,ab OR 'TB infection':ti,ab OR 'Thrombocytopenic Purpura*':ti,ab OR 'Thrombopenic Purpura*':ti,ab OR 'Thrombotic Microangiopath*':ti,ab OR 'translocation 15 21 22':ti,ab OR 'trisomy 21 syndrome':ti,ab OR 'Troisier Hanot Chauffard Syndrome':ti,ab OR 'Troisier-Hanot-Chauffard Syndrome*':ti,ab OR 'Tuberculos*':ti,ab OR 'tuberculous infection':ti,ab OR 'tuberculous lesion':ti,ab OR 'Von

Recklenhausen Applebaum Disease':ti,ab OR 'Von Recklenhausen-Applebaum Diseases':ti,ab OR 'Acute Autoimmune Neuropathies':ti,ab OR 'Acute Autoimmune Neuropathy':ti,ab OR 'Acute Cerebrovascular Accident*':ti,ab OR 'acute cerebrovascular lesion':ti,ab OR 'acute febrile polyneuritis':ti,ab OR 'acute focal cerebral vasculopathy':ti,ab OR 'Acute Infectious Polyneuritis':ti,ab OR 'Acute Inflammatory Demyelinating Polyneuropath*':ti,ab OR 'Acute Inflammatory Demyelinating Polyradiculoneuropath*':ti,ab OR 'Acute Inflammatory Polyneuropath*':ti,ab OR 'Acute Inflammatory Polyradiculoneuropath*':ti,ab OR 'acute postinfective polyradiculoneuropathy':ti,ab OR 'Acute Stroke*':ti,ab OR 'Alzeimer':ti,ab OR 'Alzheimer':ti,ab OR 'Alzeimers disease':ti,ab OR 'Alzeimer`s disease':ti,ab OR 'Alzheimer`s Disease':ti,ab OR 'Alzheimers Disease*':ti,ab OR 'Alzheimer`s Diseases':ti,ab OR 'Alzheimer-Type Dementia':ti,ab OR 'Anti-MuSK Myasthenia Gravis':ti,ab OR 'Apoplex*':ti,ab OR 'Arteriosclerotic Dementia*':ti,ab OR 'Aura':ti,ab OR 'Auras':ti,ab OR 'autoimmune myasthenia gravis':ti,ab OR 'Binswanger Disease*':ti,ab OR 'Binswanger Encephalopath*':ti,ab OR 'Binswangers Disease*':ti,ab OR 'Binswanger`s Disease*':ti,ab OR 'Binswangers Encephalopath*':ti,ab OR 'Binswanger`s Encephalopath*':ti,ab OR 'brain accident':ti,ab OR 'brain attack':ti,ab OR 'brain blood flow disturbance':ti,ab OR 'brain insult':ti,ab OR 'brain insultus':ti,ab OR 'Brain TIA':ti,ab OR 'Brain Vascular Accident*':ti,ab OR 'Cephalgia Syndrome':ti,ab OR 'Cephalgia Syndromes':ti,ab OR 'cerebral insult':ti,ab OR 'Cerebral Stroke*':ti,ab OR 'cerebral vascular accident':ti,ab OR 'cerebral vascular insufficiency':ti,ab OR 'cerebro vascular accident':ti,ab OR 'Cerebrovascular Accident':ti,ab OR 'Cerebrovascular Accidents':ti,ab OR 'cerebrovascular arrest':ti,ab OR 'cerebrovascular failure':ti,ab OR 'cerebrovascular injury':ti,ab OR 'cerebrovascular insufficiency':ti,ab OR 'cerebrovascular insult':ti,ab OR 'Cerebrovascular Stroke*':ti,ab OR 'cerebrum vascular accident':ti,ab OR 'Chronic Daily Headache':ti,ab OR 'Chronic Daily Headaches':ti,ab OR 'Chronic Headache':ti,ab OR 'Chronic Headaches':ti,ab OR 'Chronic Progressive Subcortical Encephalopath*':ti,ab OR 'comitial disease':ti,ab OR 'congenital atonic sclerotic muscular dystrophy':ti,ab OR 'congenital muscular dystrophy':ti,ab OR 'CVA':ti,ab OR 'CVAs':ti,ab OR 'diffuse cortical sclerosis':ti,ab OR 'Disseminated Sclerosis':ti,ab OR 'Epileps*':ti,ab OR 'epileptic':ti,ab OR 'Epileptiform Neuralgia*':ti,ab OR 'erb goldflam disease':ti,ab OR 'falling sickness':ti,ab OR 'Fisher syndrome':ti,ab OR 'Fothergill Disease':ti,ab OR 'Generalized Myasthenia Gravis':ti,ab OR 'Guillain Barre':ti,ab OR 'Guillain-Barré':ti,ab OR 'Guillain-Barre':ti,ab OR 'Guillain Barré Syndrome*':ti,ab OR 'Headache Disorder':ti,ab OR 'Headache Syndrome':ti,ab OR 'Headache Syndromes':ti,ab OR 'Idiopathic Parkinson Disease*':ti,ab OR 'idiopathic parkinsonism':ti,ab OR 'infectious neuronitis':ti,ab OR 'inflammatory acute polyradiculoneuropathy':ti,ab OR 'Inflammatory Polyneuropathy Acute':ti,ab OR 'insular sclerosis':ti,ab OR 'insultus cerebralis':ti,ab OR 'Intractable Headache':ti,ab OR 'Intractable Headaches':ti,ab OR 'ischaemic attack':ti,ab OR 'ischaemic cerebral attack':ti,ab OR 'ischaemic seizure':ti,ab OR 'ischemic attack':ti,ab OR 'ischemic cerebral attack':ti,ab OR 'ischemic seizure':ti,ab OR 'lacunar dementia':ti,ab OR 'Landry paralysis':ti,ab OR 'Landry syndrome':ti,ab OR 'Landry-Guillain-Barre Syndrome':ti,ab OR 'Lewy Body Parkinson Disease*':ti,ab OR 'maternal myasthenia gravis':ti,ab OR 'mini-stroke':ti,ab OR 'multiinfarct dementia':ti,ab OR 'multi-infarct dementia':ti,ab OR 'multiinfarction dementia':ti,ab OR 'multi-infarction dementia':ti,ab OR 'multiple sclerosis':ti,ab OR 'muscle dystrophia':ti,ab OR 'muscle dystrophy':ti,ab OR 'Muscle Specific Receptor Tyrosine Kinase Myasthenia Gravis':ti,ab OR 'Muscle Specific Tyrosine Kinase Antibody Positive Myasthenia Gravis':ti,ab OR 'Muscle-Specific Receptor Tyrosine Kinase Myasthenia Gravis':ti,ab OR 'Muscle-Specific Tyrosine Kinase Antibody Positive Myasthenia Gravis':ti,ab OR 'muscular dystrophia':ti,ab OR 'muscular dystrophies':ti,ab OR 'MuSK MG':ti,ab OR 'MuSK Myasthenia Gravis':ti,ab OR 'myasthenia gravis pseudoparalitica':ti,ab OR 'myasthenia gravis pseudoparalytica':ti,ab OR 'myodystrophia':ti,ab OR 'myodystrophy':ti,ab OR 'neonatal myasthenia gravis':ti,ab OR 'nervous disease':ti,ab OR 'nervous diseases':ti,ab OR 'nervous disorder':ti,ab OR 'nervous disorders':ti,ab OR 'nervous dysfunction*':ti,ab OR 'nervous system disease':ti,ab OR 'nervous system diseases':ti,ab OR 'nervous system

disorder':ti,ab OR 'nervous system disorders':ti,ab OR 'nervous system dysfunction*':ti,ab OR 'neurologic disease':ti,ab OR 'neurologic diseases':ti,ab OR 'neurologic disorder':ti,ab OR 'neurologic disorders':ti,ab OR 'neurologic dysfunction*':ti,ab OR 'neurological disease':ti,ab OR 'neurological diseases':ti,ab OR 'neurological disorder':ti,ab OR 'neurological disorders':ti,ab OR 'neurological dysfunction*':ti,ab OR 'neuromuscular disease':ti,ab OR 'neuromuscular diseases':ti,ab OR 'neuromuscular disorder':ti,ab OR 'neuromuscular disorders':ti,ab OR 'neuromuscular dysfunction*':ti,ab OR 'Ocular Myasthenia Gravis':ti,ab OR 'Paralysis Agitans':ti,ab OR 'Parkinson dementia complex':ti,ab OR 'Parkinsons disease':ti,ab OR 'Parkinson disease':ti,ab OR 'Parkinson`s Disease*':ti,ab OR 'Presenile Dementia':ti,ab OR 'Primary Parkinsonism':ti,ab OR 'Primary Senile Degenerative Dementia':ti,ab OR 'sclerosis multiplex':ti,ab OR 'Seizure Disorder*':ti,ab OR 'Senile Dementia':ti,ab OR 'stroke':ti,ab OR 'Strokes':ti,ab OR 'Subcortical Arteriosclerotic Encephalopath*':ti,ab OR 'Subcortical Leukoencephalopathies':ti,ab OR 'Subcortical Leukoencephalopathy':ti,ab OR 'Tic Douloureux':ti,ab OR 'transient brain ischaemia':ti,ab OR 'transient brain ischemia':ti,ab OR 'Transient Brain Stem Ischemia*':ti,ab OR 'Transient Brainstem Ischemia*':ti,ab OR 'Transient Cerebral Ischemia*':ti,ab OR 'Transient Cerebral Ischaemia*':ti,ab OR 'transient ischaemic attack*':ti,ab OR 'transient ischaemic seizure*':ti,ab OR 'Transient Ischemic Attack*':ti,ab OR 'transient ischemic seizure*':ti,ab OR 'Trifacial Neuralgia*':ti,ab OR 'Trigeminal Neuralgia*':ti,ab OR 'Vascular Dementia*':ti,ab OR 'acute paranasal sinusitis':ti,ab OR 'airway disease':ti,ab OR 'airway diseases':ti,ab OR 'airway disorder*':ti,ab OR 'airway dysfunction*':ti,ab OR 'Asthma*':ti,ab OR 'Atrophic Rhinitides':ti,ab OR 'Atrophic Rhinitis':ti,ab OR 'Auditory Vertigo':ti,ab OR 'Auditory Vertigos':ti,ab OR 'Aural Vertigo':ti,ab OR 'benign paroxysmal postural vertigo':ti,ab OR 'benign postural paroxysmal vertigo':ti,ab OR 'bronchitis chronica':ti,ab OR 'cerebral vertigo':ti,ab OR 'Chronic Airflow Obstruction*':ti,ab OR 'chronic airway obstruction':ti,ab OR 'Chronic Bronchitis':ti,ab OR 'chronic bronchus infection':ti,ab OR 'chronic emphysema':ti,ab OR 'chronic obstructive bronchopulmonary disease':ti,ab OR 'Chronic Obstructive Lung Disease':ti,ab OR 'chronic obstructive lung disorder':ti,ab OR 'Chronic Obstructive Pulmonary Disease':ti,ab OR 'Chronic Obstructive Pulmonary Diseases':ti,ab OR 'chronic obstructive pulmonary disorder':ti,ab OR 'chronic pulmonary obstructive disease':ti,ab OR 'chronic pulmonary obstructive disorder':ti,ab OR 'COAD':ti,ab OR 'cochlea hydrops':ti,ab OR 'COPD':ti,ab OR 'Cystic Fibrosis of Pancreas':ti,ab OR 'diffuse parenchyma lung disease':ti,ab OR 'Diffuse Parenchymal Lung Disease':ti,ab OR 'Diffuse Parenchymal Lung Diseases':ti,ab OR 'diffuse parenchymal pulmonary disease':ti,ab OR 'diffuse parenchymal pulmonary disorder':ti,ab OR 'endolymphatic hydrops':ti,ab OR 'endolymphatic sac hydrops':ti,ab OR 'Ethmoid Sinusitides':ti,ab OR 'Ethmoid Sinusitis':ti,ab OR 'Ethmoidal Sinusitides':ti,ab OR 'Ethmoidal Sinusitis':ti,ab OR 'fibrocystic disease':ti,ab OR 'hydrops labyrinthi':ti,ab OR 'Hypersomnia with Periodic Respiration':ti,ab OR 'Interstitial Lung Disease':ti,ab OR 'Interstitial Lung Diseases':ti,ab OR 'interstitial lung disorder':ti,ab OR 'Interstitial Pneumonia':ti,ab OR 'Interstitial Pneumonias':ti,ab OR 'Interstitial Pneumonitides':ti,ab OR 'Interstitial Pneumonitis':ti,ab OR 'interstitial pneumopathy':ti,ab OR 'interstitial pulmonary disease':ti,ab OR 'interstitial pulmonary disorder':ti,ab OR 'labyrinth hydrops':ti,ab OR 'labyrinthal syndrome':ti,ab OR 'lung allergy':ti,ab OR 'lung chronic obstructive disease':ti,ab OR 'Meniere Disease':ti,ab OR 'Ménière Disease':ti,ab OR 'Ménière Diseases':ti,ab OR 'Meniere Syndrome':ti,ab OR 'Ménière Vertigo':ti,ab OR 'Menieres Disease':ti,ab OR 'Meniere`s Disease':ti,ab OR 'Ménières Disease':ti,ab OR 'Ménière`s Disease':ti,ab OR 'Ménière`s Diseases':ti,ab OR 'Menieres Syndrome':ti,ab OR 'Meniere`s Syndrome':ti,ab OR 'Ménières Vertigo':ti,ab OR 'Ménière`s Vertigo':ti,ab OR 'Ménière`s Vertigos':ti,ab OR 'Mucoviscidosis':ti,ab OR 'mucoviscoidosis':ti,ab OR 'nasal sinusitis':ti,ab OR 'nocturnal apnea':ti,ab OR 'nocturnal apnoea':ti,ab OR 'obstructive chronic lung disease':ti,ab OR 'obstructive chronic pulmonary disease':ti,ab OR 'Otogenic Vertigo':ti,ab OR 'Otogenic Vertigos':ti,ab OR 'Otoscleroses':ti,ab OR 'otosclerosis surgery':ti,ab OR 'otosclerotic stapes':ti,ab OR 'otosphongiosis':ti,ab OR 'Otospongioses':ti,ab OR

'Otospongiosis':ti,ab OR 'Ozena':ti,ab OR 'Ozenas':ti,ab OR 'pancreas cystic disease':ti,ab OR 'pancreas cystic fibrosis':ti,ab OR 'Pancreas Fibrocystic Diseases':ti,ab OR 'pancreas fibrosis':ti,ab OR 'pancreatic cystic disease':ti,ab OR 'Pancreatic Cystic Fibrosis':ti,ab OR 'pancreatic fibrosis':ti,ab OR 'paroxysmal labyrinthine vertigo':ti,ab OR 'paroxysmal positional vertigo':ti,ab OR 'pneumatosis':ti,ab OR 'positional paroxysmal vertigo':ti,ab OR 'Pulmonary Cystic Fibrosis':ti,ab OR 'respiration disease':ti,ab OR 'respiration diseases':ti,ab OR 'respiration disorder':ti,ab OR 'respiration disorders':ti,ab OR 'respiration dysfunction*':ti,ab OR 'respiration tract disease':ti,ab OR 'respiration tract diseases':ti,ab OR 'respiration tract disorder':ti,ab OR 'respiration tract disorders':ti,ab OR 'respiration tract dysfunction*':ti,ab OR 'respiratory disease':ti,ab OR 'respiratory diseases':ti,ab OR 'respiratory disorder':ti,ab OR 'respiratory disorders':ti,ab OR 'respiratory illness*':ti,ab OR 'respiratory tract disease':ti,ab OR 'respiratory tract diseases':ti,ab OR 'respiratory tract disorder':ti,ab OR 'respiratory tract disorders':ti,ab OR 'respiratory tract dysfunction':ti,ab OR 'respiratory tract dysfunctioning':ti,ab OR 'respiratory tract dysfunctions':ti,ab OR 'rhinitis atrophica':ti,ab OR 'sinusitis nasalis':ti,ab OR 'Sleep Apnea':ti,ab OR 'Sleep Apneas':ti,ab OR 'sleep apnoea':ti,ab OR 'Sleep Disordered Breathing':ti,ab OR 'Sleep Hypopnea':ti,ab OR 'Sleep Hypopneas':ti,ab OR 'Sleep-Disordered Breathing':ti,ab OR 'vertiginous disease':ti,ab OR 'vertiginous disorder':ti,ab OR 'vertiginous syndrome':ti,ab OR 'vestibular vertigo':ti,ab OR 'Age Related Osteoporosis':ti,ab OR 'Age-Related Bone Loss':ti,ab OR 'Age-Related Bone Losses':ti,ab OR 'Age-Related Osteoporosis':ti,ab OR 'Akureyri disease':ti,ab OR 'Algodystrophic Syndrome':ti,ab OR 'Algodystrophies':ti,ab OR 'Algodystrophy':ti,ab OR 'alibert bazin disease':ti,ab OR 'arthropathic psoriasis':ti,ab OR 'Arthroses':ti,ab OR 'Arthrosis':ti,ab OR 'axial spondylarthritis':ti,ab OR 'Axial Spondyloarthritides':ti,ab OR 'Axial Spondyloarthritis':ti,ab OR 'AxSpA':ti,ab OR 'Back Ache':ti,ab OR 'Back Aches':ti,ab OR 'Back Pain*':ti,ab OR 'Backache':ti,ab OR 'Backaches':ti,ab OR 'backpain':ti,ab OR 'beauvais disease':ti,ab OR 'Bilateral Sciatica':ti,ab OR 'Bilateral Sciaticas':ti,ab OR 'Bone Paget Disease':ti,ab OR 'Bone Pagets Disease':ti,ab OR 'Calcium Pyrophosphate Deposition Disease':ti,ab OR 'Calcium Pyrophosphate Dihydrate Deposition':ti,ab OR 'Cervical Pain':ti,ab OR 'Cervical Pains':ti,ab OR 'Cervical Sympathetic Dystrophies':ti,ab OR 'Cervical Sympathetic Dystrophy':ti,ab OR 'Cervicalgia':ti,ab OR 'Cervicalgias':ti,ab OR 'Cervicodynia':ti,ab OR 'Cervicodynias':ti,ab OR 'chariot disease':ti,ab OR 'Chondrocalcinoses':ti,ab OR 'chronic articular rheumatism':ti,ab OR 'chronic fatigue':ti,ab OR 'Chronic Fatigue-Fibromyalgia Syndrome*':ti,ab OR 'Chronic Infectious Mononucleosis Like Syndrome':ti,ab OR 'Chronic Infectious Mononucleosis-Like Syndrome':ti,ab OR 'chronic rheumatism':ti,ab OR 'complex regional pain syndrome 1':ti,ab OR 'complex regional pain syndrome type 1':ti,ab OR 'CRPS 1':ti,ab OR 'CRPS I':ti,ab OR 'CRPS type 1':ti,ab OR 'CRPS Type I':ti,ab OR 'CRPS-I':ti,ab OR 'crystal arthropathies':ti,ab OR 'crystalline arthropathy':ti,ab OR 'degenerative joint disease':ti,ab OR 'Diffuse Myofascial Pain Syndrome':ti,ab OR 'disc hernia':ti,ab OR 'Disc Herniation':ti,ab OR 'Disc Herniations':ti,ab OR 'disc prolapse':ti,ab OR 'Disc Protrusion':ti,ab OR 'Disc Protrusions':ti,ab OR 'discal hernia':ti,ab OR 'discal herniation':ti,ab OR 'discus hernia':ti,ab OR 'disk hernia':ti,ab OR 'Disk Herniation':ti,ab OR 'Disk Herniations':ti,ab OR 'Disk Prolapse':ti,ab OR 'Disk Prolapses':ti,ab OR 'Disk Protrusion':ti,ab OR 'Disk Protrusions':ti,ab OR 'dorsalgia':ti,ab OR 'epicondylalgia':ti,ab OR 'epidemic neuromyasthenia':ti,ab OR 'fatigue syndrome':ti,ab OR 'fibro myalgia':ti,ab OR 'Fibromyalgia Fibromyositis Syndrome':ti,ab OR 'Fibromyalgia-Fibromyositis Syndrome':ti,ab OR 'Fibromyalgia-Fibromyositis Syndromes':ti,ab OR 'Fibromyalgias':ti,ab OR 'Fibromyositis Fibromyalgia Syndrome':ti,ab OR 'Fibromyositis-Fibromyalgia Syndrome':ti,ab OR 'Fibromyositis-Fibromyalgia Syndromes':ti,ab OR 'fibrositic nodule':ti,ab OR 'Fibrositides':ti,ab OR 'Fibrositis':ti,ab OR 'hernia disci':ti,ab OR 'hernia nuclei pulposi':ti,ab OR 'Herniated Disc':ti,ab OR 'Herniated Discs':ti,ab OR 'Herniated Disk':ti,ab OR 'Herniated Disks':ti,ab OR 'herniated intervertebral disc':ti,ab OR 'herniated intervertebral disk':ti,ab OR 'herniated nucleus pulposus':ti,ab OR 'herniated vertebral disc':ti,ab OR 'herniated vertebral disk':ti,ab OR 'hypertrophic infiltrative

tendinitis':ti,ab OR 'Iceland disease':ti,ab OR 'Intervertebral Disc Displacement':ti,ab OR 'Intervertebral Disc Displacements':ti,ab OR 'Intervertebral Disk Displacement':ti,ab OR 'Intervertebral Disk Displacements':ti,ab OR 'intervertebral disk perforation':ti,ab OR 'intervertebral disk rupture':ti,ab OR 'intervertebral prolapse':ti,ab OR 'Involutional Osteoporosis':ti,ab OR 'ischias':ti,ab OR 'ischiatic pain':ti,ab OR 'Lateral Epicondylitides':ti,ab OR 'Lateral Epicondylitis':ti,ab OR 'Lateral Humeral Epicondylitides':ti,ab OR 'Lateral Humeral Epicondylitis':ti,ab OR 'loin pain':ti,ab OR 'lowback pain':ti,ab OR 'Lumbago':ti,ab OR 'lumbal pain':ti,ab OR 'lumbal syndrome':ti,ab OR 'lumbalgesia':ti,ab OR 'lumbalgia':ti,ab OR 'lumbar pain':ti,ab OR 'lumbar spine syndrome':ti,ab OR 'lumbodynia':ti,ab OR 'lumbosacral pain':ti,ab OR 'lumbosacral root syndrome':ti,ab OR 'lumbosacroiliac strain':ti,ab OR 'Muscular Rheumatism':ti,ab OR 'Myalgic Encephalomyelitis':ti,ab OR 'Neck Ache':ti,ab OR 'Neck Aches':ti,ab OR 'Neck Pain*':ti,ab OR 'Neckache':ti,ab OR 'Neckaches':ti,ab OR 'neuralgic shoulder amyotrophy':ti,ab OR 'nodular tendinitis':ti,ab OR 'nucleus pulposus hernia':ti,ab OR 'Osseous Paget`s Disease':ti,ab OR 'osteitis deformans':ti,ab OR 'Osteoarthritides':ti,ab OR 'osteo-arthritis':ti,ab OR 'Osteoarthroses':ti,ab OR 'Osteoarthrosis':ti,ab OR 'osteo-arthrosis':ti,ab OR 'Osteoporoses':ti,ab OR 'ostitis deformans':ti,ab OR 'Paget Disease of Bone':ti,ab OR 'Paget disease of the bone':ti,ab OR 'Pagets bone disease':ti,ab OR 'Paget`s bone disease':ti,ab OR 'Pagets disease of bone':ti,ab OR 'Paget`s Disease of Bone':ti,ab OR 'Paget`s disease of the bone':ti,ab OR 'paralytic scoliosis':ti,ab OR 'Post Traumatic Osteoporosis':ti,ab OR 'posttraumatic dystrophy':ti,ab OR 'post-traumatic dystrophy':ti,ab OR 'Post-Traumatic Osteoporosis':ti,ab OR 'Postviral Fatigue Syndromes':ti,ab OR 'Primary Fibromyalgia':ti,ab OR 'primary osteoarthritis':ti,ab OR 'progressive scoliosis':ti,ab OR 'Prolapsed Disc':ti,ab OR 'Prolapsed Discs':ti,ab OR 'Prolapsed Disk':ti,ab OR 'Prolapsed Disks':ti,ab OR 'Protruded Disc':ti,ab OR 'Protruded Discs':ti,ab OR 'Protruded Disk':ti,ab OR 'Protruded Disks':ti,ab OR 'Pseudogout':ti,ab OR 'Psoriasis Arthropathica':ti,ab OR 'psoriasis pustulosa arthropathica':ti,ab OR 'Psoriatic Arthropathies':ti,ab OR 'Psoriatic Arthropathy':ti,ab OR 'psoriatic rheumatism':ti,ab OR 'Reflex Sympathetic Dystrophies':ti,ab OR 'Reflex Sympathetic Dystrophy':ti,ab OR 'rheumarthritis':ti,ab OR 'rheumatic disease':ti,ab OR 'rheumatic diseases':ti,ab OR 'rheumatoid disease':ti,ab OR 'rheumatoid diseases':ti,ab OR 'rheumatoid inflammation':ti,ab OR 'rheumatological disease':ti,ab OR 'rheumatological diseases':ti,ab OR 'rheumatological disorder':ti,ab OR 'rheumatological disorders':ti,ab OR 'Royal Free Disease':ti,ab OR 'Sciatic Neuralgia':ti,ab OR 'Sciatic Neuralgias':ti,ab OR 'sciatic pain':ti,ab OR 'Scolioses':ti,ab OR 'Secondary Fibromyalgia':ti,ab OR 'Senile Osteoporosis':ti,ab OR 'shoulder arm syndrome':ti,ab OR 'Shoulder Hand Syndrome':ti,ab OR 'Shoulder-Hand Syndrome':ti,ab OR 'Shoulder-Hand Syndromes':ti,ab OR 'Slipped Disc':ti,ab OR 'Slipped Discs':ti,ab OR 'Slipped Disk':ti,ab OR 'Slipped Disks':ti,ab OR 'slipped intervertebral disc':ti,ab OR 'slipped vertebral disc':ti,ab OR 'spinal disk disease':ti,ab OR 'Sudek Atrophy':ti,ab OR 'Sudek`s Atrophies':ti,ab OR 'Sudeks Atrophy':ti,ab OR 'Sudek`s Atrophy':ti,ab OR 'sympathetic dystrophy syndrome':ti,ab OR 'Sympathetic Reflex Dystrophia':ti,ab OR 'Sympathetic Reflex Dystrophias':ti,ab OR 'sympathetic reflex dystrophy':ti,ab OR 'Systemic Exertion Intolerance Disease':ti,ab OR 'tendinopathy':ti,ab OR 'tendinosis':ti,ab OR 'tendonitis':ti,ab OR 'tendonopathy':ti,ab OR 'Tennis Elbow':ti,ab OR 'Tennis Elbows':ti,ab OR 'tenonitis':ti,ab OR 'tenontitis':ti,ab OR 'tenositis':ti,ab OR 'Type I Complex Regional Pain Syndrome':ti,ab OR 'Vertebrogenic Pain Syndrome':ti,ab OR 'Vertebrogenic Pain Syndromes':ti,ab OR 'Yuppie flu':ti,ab OR 'Acne Inversa*':ti,ab OR 'acne juvenilis':ti,ab OR 'Acne Rosacea':ti,ab OR 'Chronic Bullous Disease of Childhood':ti,ab OR 'cutaneous disease':ti,ab OR 'cutaneous diseases':ti,ab OR 'cutaneus disorder':ti,ab OR 'cutaneus disorders':ti,ab OR 'dermal disease':ti,ab OR 'dermal diseases':ti,ab OR 'dermal disorder':ti,ab OR 'dermal disorders':ti,ab OR 'Drug induced Linear IgA Bullous Dermatos*':ti,ab OR 'Drug-induced Linear IgA Bullous Dermatos*':ti,ab OR 'Eczema*':ti,ab OR 'Erythematotelangiectatic Rosacea':ti,ab OR 'Granulomatous Rosacea':ti,ab OR 'herpes':ti,ab OR 'hidradenitis suppurativa':ti,ab OR 'Hives':ti,ab OR 'juvenile acne':ti,ab OR 'Linear IgA

Dermatos*':ti,ab OR 'Linear IgA IgG Bullous Dermatos*':ti,ab OR 'Linear IgA IgG Dermatos*':ti,ab OR 'Ocular Rosacea':ti,ab OR 'Palmoplantaris Pustulosis':ti,ab OR 'Papulopustular Rosacea':ti,ab OR 'Phymatous Rosacea':ti,ab OR 'Psoriases':ti,ab OR 'psoriasiform dermatitis':ti,ab OR 'psoriasiform dermatosis':ti,ab OR 'psoriasiform lesion':ti,ab OR 'psoriasiform rash':ti,ab OR 'psoriasiform skin rash':ti,ab OR 'psoriatic epidermis':ti,ab OR 'psoriatic skin':ti,ab OR 'Pustular Psoriasis of Palms and Soles':ti,ab OR 'Pustulosis of Palms and Soles':ti,ab OR 'Pustulosis Palmaris et Plantaris':ti,ab OR 'rhinophyma':ti,ab OR 'rozacea':ti,ab OR 'skin and connective tissue disease':ti,ab OR 'skin and connective tissue diseases':ti,ab OR 'skin and connective tissue disorder':ti,ab OR 'skin and connective tissue disorders':ti,ab OR 'skin disease':ti,ab OR 'skin diseases':ti,ab OR 'skin disorder':ti,ab OR 'skin disorders':ti,ab OR 'Suppurative Hidradeniti*':ti,ab OR 'urticary':ti,ab OR 'weal':ti,ab OR 'wheal':ti,ab OR 'whealing':ti,ab OR 'willan lepra':ti,ab OR 'active TB':ti,ab OR 'Adamantiades-Behcet Disease*':ti,ab OR 'Allergic Angiit*':ti,ab OR 'Allergic Granulomatos*':ti,ab OR 'Allergic Granulomatous and Angiitis':ti,ab OR 'Allergic Granulomatous Angiit*':ti,ab OR 'allergic granulomatous angitis':ti,ab OR 'anonymous artery occlusion':ti,ab OR 'Anti Phospholipid Antibody Syndrome*':ti,ab OR 'Anti Phospholipid Syndrome*':ti,ab OR 'Antiphospholipid Antibody Syndrome*':ti,ab OR 'Anti-Phospholipid Antibody Syndrome*':ti,ab OR 'antiphospholipid syndrome':ti,ab OR 'Anti-Phospholipid Syndrome*':ti,ab OR 'aorta arch syndrome':ti,ab OR 'aortic arch syndromes':ti,ab OR 'Aortitis Syndrome*':ti,ab OR 'APLA syndrome':ti,ab OR 'arteritis brachiocephalica':ti,ab OR 'arteritis nodosa':ti,ab OR 'autoimmune disease':ti,ab OR 'autoimmune diseases':ti,ab OR 'Behcet Disease*':ti,ab OR 'Behçet Disease*':ti,ab OR 'Behcet syndrome':ti,ab OR 'behcet ulcer':ti,ab OR 'Behcets disease':ti,ab OR 'Behcet`s Disease*':ti,ab OR 'Behcets syndrome':ti,ab OR 'Behcet`s Syndrome*':ti,ab OR 'besnier boeck syndrome':ti,ab OR 'Besnier-Boeck Disease*':ti,ab OR 'Besnier-Boeck-Schaumann Syndrome*':ti,ab OR 'Boeck Disease*':ti,ab OR 'Boecks Disease*':ti,ab OR 'Boeck`s Disease*':ti,ab OR 'brachiocephalic arteritis':ti,ab OR 'brachiocephalic artery occlusion':ti,ab OR 'brachiocephalic ischaemia':ti,ab OR 'brachiocephalic ischemia':ti,ab OR 'brachiocephalic trunk occlusion':ti,ab OR 'brachiocephalic vascular occlusion':ti,ab OR 'Church Strauss syndrome':ti,ab OR 'Churg Strauss':ti,ab OR 'Churg-Strauss Syndrome':ti,ab OR 'Cranial Arterit*':ti,ab OR 'cryoglobulinaemia':ti,ab OR 'Cryoglobulinemias':ti,ab OR 'cryoimmunoglobulinaemia':ti,ab OR 'cryoimmunoglobulinemia':ti,ab OR 'Cutis Elastica':ti,ab OR 'dacryosialoadenopathia atrophicans':ti,ab OR 'EDS IV':ti,ab OR 'Ehlers Danlos':ti,ab OR 'Ehlers-Danlos Disease*':ti,ab OR 'Ehlers-Danlos syndrome':ti,ab OR 'eosinophilic GPA':ti,ab OR 'eosinophilic granulomatosis polyangiitis':ti,ab OR 'eosinophilic granulomatosis polyangitis':ti,ab OR 'eosinophilic granulomatous angiitis':ti,ab OR 'Eosinophilic Granulomatous Vasculit*':ti,ab OR 'erythematodes visceralis':ti,ab OR 'Essential Polyarterit*':ti,ab OR 'generalised scleroderma':ti,ab OR 'generalized scleroderma':ti,ab OR 'Giant Cell Aortic Arteritis':ti,ab OR 'Giant Cell Aortiti*':ti,ab OR 'giant cell arteriitis':ti,ab OR 'Giant Cell Arteriti*':ti,ab OR 'gougerot houwer sjoegren syndrome':ti,ab OR 'gougerot mulock houwer sjoegren syndrome':ti,ab OR 'Gougerot Sjoegren syndrome':ti,ab OR 'Gougerot-Sjogren syndrome':ti,ab OR 'granulomatosis and polyangiitis':ti,ab OR 'granulomatosis and polyangitis':ti,ab OR 'Granulomatosis with Polyangiit*':ti,ab OR 'granulomatosis with polyangitis':ti,ab OR 'granulomatous allergic angitis':ti,ab OR 'granulomatous polyangiitis':ti,ab OR 'granulomatous polyangitis':ti,ab OR 'Horton arteritis':ti,ab OR 'Horton Disease':ti,ab OR 'Horton`s arteritis':ti,ab OR 'Hortons Disease':ti,ab OR 'Horton`s Disease':ti,ab OR 'Hughes Syndrome*':ti,ab OR 'innominate arterial ligation':ti,ab OR 'innominate artery ligation':ti,ab OR 'innominate artery occlusion':ti,ab OR 'jungling syndrome':ti,ab OR 'kussmaul maier disease':ti,ab OR 'kussmaul syndrome':ti,ab OR 'Libman Sacks Disease*':ti,ab OR 'Libman-Sacks Disease*':ti,ab OR 'lupovisceritis':ti,ab OR 'lymphogranuloma benignum':ti,ab OR 'malignant dermatovisceritism':ti,ab OR 'martorell syndrome':ti,ab OR 'Microscopic Polyangiitides':ti,ab OR 'microscopic polyarteritis':ti,ab OR 'mikulicz radecki syndrome':ti,ab OR 'mixed cryoglobulinemia':ti,ab OR 'morbus Wegener':ti,ab OR 'mucoserous

dyssecretosis':ti,ab OR 'mukilicz radecki syndrome':ti,ab OR 'multisystem disease':ti,ab OR 'multisystem diseases':ti,ab OR 'multisystem disorder':ti,ab OR 'multisystem disorders':ti,ab OR 'multisystem dysfunction*':ti,ab OR 'necrotising respiratory granulomatosis':ti,ab OR 'Necrotizing Arterit*':ti,ab OR 'necrotizing respiratory granulomatosis':ti,ab OR 'nodular periarteritis':ti,ab OR 'nodular polyarteritis':ti,ab OR 'oculobuccopharyngeal dryness':ti,ab OR 'Old Silk Route Disease*':ti,ab OR 'panarteriitis nodosa':ti,ab OR 'panarteritis nodosa':ti,ab OR 'periarterial fibrosis':ti,ab OR 'periarteriitis nodosa':ti,ab OR 'Periarteritis Nodosa':ti,ab OR 'pneumogenic granulomatosis':ti,ab OR 'poliarteritis nodosa':ti,ab OR 'polyarteriitis nodosa':ti,ab OR 'progressive scleroderma':ti,ab OR 'progressive sclerodermia':ti,ab OR 'Pulseless Disease*':ti,ab OR 'reversed coarctation':ti,ab OR 'rheumatic sialosis':ti,ab OR 'sarcoid':ti,ab OR 'sarcoidoses':ti,ab OR 'Schaumann Disease*':ti,ab OR 'Schaumann Syndrome*':ti,ab OR 'Schaumann`s Syndrome*':ti,ab OR 'sicca syndrome':ti,ab OR 'sjoegren disease':ti,ab OR 'sjogren disease':ti,ab OR 'sjogren syndrome':ti,ab OR 'sjogren`s syndrome':ti,ab OR 'lupus':ti,ab OR 'Strauss Churg syndrome':ti,ab OR 'systemic disease':ti,ab OR 'systemic diseases':ti,ab OR 'systemic disorder*':ti,ab OR 'systemic dysfunction*':ti,ab OR 'systemic progressive sclerosis':ti,ab OR 'Systemic Scleroderma':ti,ab OR 'Systemic Sclerosis':ti,ab OR 'takayasu arteriopathy':ti,ab OR 'Takayasu Arteritis':ti,ab OR 'Takayasu Disease*':ti,ab OR 'takayasu ohnishi syndrome':ti,ab OR 'Takayasu Syndrome*':ti,ab OR 'Takayasus Arteritis':ti,ab OR 'Takayasu`s Arteritis':ti,ab OR 'Temporal Arterit*':ti,ab OR 'Triple Symptom Complex':ti,ab OR 'Triple Symptom Complices':ti,ab OR 'Triple-Symptom Complex':ti,ab OR 'Wegener disease':ti,ab OR 'Wegener granuloma':ti,ab OR 'Wegener Granulomatosis':ti,ab OR 'Wegener Klinger Churg syndrome':ti,ab OR 'Wegener Klinger granulomatosis':ti,ab OR 'Wegener syndrome':ti,ab OR 'Wegener`s disease':ti,ab OR 'Wegener`s GPA':ti,ab OR 'Wegener`s granuloma':ti,ab OR 'Wegener`s Granulomatosis':ti,ab OR 'Wegner granulomatosis':ti,ab OR 'Young Female Arterit*':ti,ab OR 'Anti GBM Disease*':ti,ab OR 'Anti Glomerular Basement Membrane Disease':ti,ab OR 'Anti-GBM Disease*':ti,ab OR 'bladder incontinence':ti,ab OR 'chronic disease kidney function':ti,ab OR 'Chronic Kidney Failure*':ti,ab OR 'chronic nephropathy':ti,ab OR 'Chronic Renal Failure*':ti,ab OR 'cystic kidney':ti,ab OR 'End Stage Kidney Disease*':ti,ab OR 'End Stage Renal Disease*':ti,ab OR 'End Stage Renal Failure*':ti,ab OR 'End-Stage Kidney Disease*':ti,ab OR 'End-Stage Renal Disease*':ti,ab OR 'End-Stage Renal Failure*':ti,ab OR 'ESRD':ti,ab OR 'familial nephrolithiasis':ti,ab OR 'goodpasture disease':ti,ab OR 'goodpasture renopulmonary syndrome':ti,ab OR 'Goodpasture Syndrome*':ti,ab OR 'Goodpastures Syndrome*':ti,ab OR 'Goodpasture`s Syndrome*':ti,ab OR 'incontinentia urinae':ti,ab OR 'involuntary urinary loss':ti,ab OR 'involuntary urination':ti,ab OR 'involuntary urine loss':ti,ab OR 'Kidney Calcul*':ti,ab OR 'kidney calix stone':ti,ab OR 'kidney calyx stone':ti,ab OR 'kidney chronic failure':ti,ab OR 'kidney cystic disease':ti,ab OR 'kidney disease':ti,ab OR 'kidney diseases':ti,ab OR 'kidney disorder':ti,ab OR 'kidney disorders':ti,ab OR 'kidney failure':ti,ab OR 'kidney failures':ti,ab OR 'kidney insufficien*':ti,ab OR 'kidney lithiasis':ti,ab OR 'kidney multicystic aplasia':ti,ab OR 'kidney multicystic disease':ti,ab OR 'kidney pelvis stone':ti,ab OR 'kidney polycystosis':ti,ab OR 'Kidney Stone*':ti,ab OR 'leakage of urine':ti,ab OR 'Lung Purpura with Nephritis':ti,ab OR 'male genital disorder':ti,ab OR 'male genital disorders':ti,ab OR 'male infertility':ti,ab OR 'Nephrolith':ti,ab OR 'pneumorenal syndrome':ti,ab OR 'Polycystic Kidney':ti,ab OR 'Polycystic Kidneys':ti,ab OR 'prostate adenoma':ti,ab OR 'Renal Calcul*':ti,ab OR 'renal cystic disease':ti,ab OR 'renal disease':ti,ab OR 'renal diseases':ti,ab OR 'renal disorder':ti,ab OR 'renal disorders':ti,ab OR 'renal failure':ti,ab OR 'renal failures':ti,ab OR 'renal insufficien*':ti,ab OR 'renal pelvis stone':ti,ab OR 'renal polycystic disease':ti,ab OR 'renal stone':ti,ab OR 'renolithiasis':ti,ab OR 'unwanted urine loss':ti,ab OR 'urinary incontinence':ti,ab OR 'urinary leakage':ti,ab OR 'urine incontinence':ti,ab OR 'urine leakage':ti,ab OR 'urine wetting':ti,ab OR 'urologic disease*':ti,ab OR 'urologic disorder*':ti,ab OR 'urological disease*':ti,ab OR 'urological disorder*':ti,ab OR 'Age Related Macular Degeneration':ti,ab OR 'Age-Related Macular Degeneration*':ti,ab OR 'atrophia maculae

luteae':ti,ab OR 'bilateral macular degeneration':ti,ab OR 'Cataract*':ti,ab OR 'chronic uveitis':ti,ab OR 'corpus vitreum detachment':ti,ab OR 'Day Blindness':ti,ab OR 'degeneratio maculae luteae retinae':ti,ab OR 'detachment corporis vitrei':ti,ab OR 'detachment vitreous':ti,ab OR 'disciform macular degeneration':ti,ab OR 'Episclerit*':ti,ab OR 'eye disease':ti,ab OR 'eye diseases':ti,ab OR 'eye disorder':ti,ab OR 'eye disorders':ti,ab OR 'eye dysfunction':ti,ab OR 'eye dysfunctioning':ti,ab OR 'eye dysfunctions':ti,ab OR 'Hemeralopia*':ti,ab OR 'heredomacular degeneration':ti,ab OR 'immunogenic uveitis':ti,ab OR 'junius kuhnt disease':ti,ab OR 'lens clouding':ti,ab OR 'Lens Opacit*':ti,ab OR 'Macropsia*':ti,ab OR 'macula atrophy':ti,ab OR 'macula bilateral degeneration':ti,ab OR 'macula degeneration':ti,ab OR 'macula lutea atrophy':ti,ab OR 'macula lutea degeneration':ti,ab OR 'macula lutea disciform degeneration':ti,ab OR 'macula lutea retina atrophy':ti,ab OR 'macula lutea retina degeneration':ti,ab OR 'macula retina atrophy':ti,ab OR 'macula retina degeneration':ti,ab OR 'macular atrophy':ti,ab OR 'Macular Degenerations':ti,ab OR 'macular disciform degeneration':ti,ab OR 'Macular Dystroph*':ti,ab OR 'Maculopath*':ti,ab OR 'Metamorphopsia*':ti,ab OR 'Micropsia*':ti,ab OR 'Myopia*':ti,ab OR 'Nearsightedness*':ti,ab OR 'Necrotizing Sclerit*':ti,ab OR 'ocular disease':ti,ab OR 'ocular diseases':ti,ab OR 'ocular disorder':ti,ab OR 'ocular disorders':ti,ab OR 'ocular dysfunction*':ti,ab OR 'ophthalmic disease':ti,ab OR 'ophthalmic diseases':ti,ab OR 'ophthalmic disorder':ti,ab OR 'ophthalmic disorders':ti,ab OR 'ophthalmic dysfunction*':ti,ab OR 'ophthalmologic disease':ti,ab OR 'ophthalmologic diseases':ti,ab OR 'ophthalmologic disorder':ti,ab OR 'ophthalmologic disorders':ti,ab OR 'ophthalmologic dysfunction*':ti,ab OR 'panuveitis':ti,ab OR 'posterior capsule opacification':ti,ab OR 'posterior uveitis':ti,ab OR 'Presbyopias':ti,ab OR 'Pseudoaphakia*':ti,ab OR 'retina macula disciform degeneration':ti,ab OR 'retinal diseases':ti,ab OR 'Scleritides':ti,ab OR 'secondary scleritis':ti,ab OR 'suppurative uveitis':ti,ab OR 'Uveitides':ti,ab OR 'Vision Disabilit*':ti,ab OR 'vision disorder':ti,ab OR 'vision disorders':ti,ab OR 'vision disturbance':ti,ab OR 'visual disorder':ti,ab OR 'visual disorders':ti,ab OR 'visual disturbance':ti,ab OR 'Visual Impairment*':ti,ab OR 'vitreous detachment':ti,ab OR 'sarcoidosis':ti,ab OR 'neoplasia':ti,ab OR 'lymphoma*':ti,ab OR 'hypertension':ti,ab OR 'infarction*':ti,ab OR 'glaucom*':ti,ab OR 'cancer*':ti,ab OR 'carcinoma*':ti,ab OR 'neoplasm*':ti,ab OR 'tumor*':ti,ab OR 'tumour*':ti,ab OR 'Horton syndrome':ti,ab OR 'Horton`s syndrome':ti,ab OR 'Gastro-esophageal reflux':ti,ab OR 'gastroesophageal reflux':ti,ab OR 'GORD':ti,ab) AND ('sexual dysfunction'/exp OR 'psychosexual disorder'/exp OR 'sexuality'/de OR 'sexual desire'/exp OR 'sexual arousal'/exp OR 'erectile dysfunction'/exp OR 'libido'/exp OR 'orgasm'/exp OR 'vaginism'/exp OR 'dyspareunia'/exp OR 'sexual arousal disorder'/exp OR 'libido disorder'/exp OR 'orgasm disorder'/exp OR 'coitus':ti,ab OR 'desire':ti,ab OR 'dyspareunia':ti,ab OR 'ego-dystonic homosexuality':ti,ab OR 'ejaculatio praecox':ti,ab OR 'ejaculatio praecoxs':ti,ab OR 'ejaculatio precox':ti,ab OR 'erectile dysfunction*':ti,ab OR 'erection':ti,ab OR 'frigidities':ti,ab OR 'frigidity':ti,ab OR 'genital disorder':ti,ab OR 'genital disorders':ti,ab OR 'go-dystonic homosexuality':ti,ab OR 'libido':ti,ab OR 'orgasm':ti,ab OR 'orgasms':ti,ab OR 'premature ejaculation':ti,ab OR 'premature ejaculations':ti,ab OR 'psychosexuality':ti,ab OR 'sex abnormalit*':ti,ab OR 'sex arousal':ti,ab OR 'sex disorder':ti,ab OR 'sex disorders':ti,ab OR 'sex drive':ti,ab OR 'sex dysfunction*':ti,ab OR 'sex insufficienc*':ti,ab OR 'sex problem':ti,ab OR 'sex problems':ti,ab OR 'vaginismus':ti,ab OR 'sexual':ti,ab OR 'psychosexual':ti,ab OR 'sexuality':ti,ab) AND ('adult'/exp OR 'adult':ti,ab OR 'adults':ti,ab OR 'old people':ti,ab OR 'elderly':ti,ab OR 'frail':ti,ab OR 'frailness':ti,ab OR 'frailty':ti,ab OR 'old age':ti,ab OR 'old patients':ti,ab OR 'old person':ti,ab OR 'old persons':ti,ab OR 'older adult':ti,ab OR 'older adults':ti,ab OR 'older patient':ti,ab OR 'older patients':ti,ab OR 'older people':ti,ab OR 'older person':ti,ab OR 'older persons':ti,ab OR 'senior people':ti,ab OR 'senior person':ti,ab OR 'senior persons':ti,ab OR 'seniors':ti,ab) AND ('systematic review'/exp OR 'meta analysis'/exp OR 'scoping review'/exp OR 'scoping review*':ti,ab OR 'systematic review*':ti,ab OR 'systematic literature review*':ti,ab OR 'systematic narrative review*':ti,ab OR 'systematic qualitative review*':ti,ab OR 'systematic evidence

review*':ti,ab OR 'systematic quantitative review*':ti,ab OR 'systematic meta-review*':ti,ab OR 'systematic critical review*':ti,ab OR 'systematic mapping review*':ti,ab OR 'systematic cochrane review*':ti,ab OR 'systematic electronic literature search*':ti,ab OR 'PRISMA':ti,ab OR 'systematic descriptive review*':ti,ab OR 'systematic analys*':ti,ab OR 'targeted literature review*':ti,ab OR 'meta-synthes*':ti,ab OR 'comprehensive review*':ti,ab OR 'mixed studies review*':ti,ab OR 'sistematic review*':ti,ab OR 'umbrella review*':ti,ab OR 'mini-review*':ti,ab OR 'rapid literature review*':ti,ab OR 'rapid review*':ti,ab OR 'integrative review*':ti,ab OR 'systematic and critical analysis review*':ti,ab OR 'systematically review evidence':ti,ab OR 'systematic search*':ti,ab OR 'systematic methodological review*':ti,ab OR 'metaanalys*':ti,ab OR 'meta-analytic review*':ti,ab OR 'meta-analys*':ti,ab OR 'cochrane review*':ti,ab)

PSYCHINFO :

(SU.EXPLODE("male genital disorders") OR SU.EXPLODE("endocrine sexual disorders") OR SU.EXPLODE("cardiovascular disorders") OR SU.EXPLODE("endocrine disorders") OR SU.EXPLODE("obesity") OR SU.EXPLODE("arthritis") OR SU.EXPLODE("kidney diseases") OR SU.EXPLODE("digestive system disorders") OR SU.EXPLODE("nervous system disorders") OR SU.EXPLODE("skin disorders") OR SU.EXPLODE("eye disorders") OR SU.EXPLODE("neoplasms") OR SU.EXPLODE("respiratory tract disorders") OR SU.EXPLODE("blood and lymphatic disorders") OR SU.EXPLODE("infectious disorders") OR MESH("Cardiovascular Diseases") OR MESH("Heart diseases") OR MESH("Pulmonary Embolism") OR MESH("Phlebitis") OR MESH("Hypercholesterolemia") OR MESH("Hypertension") OR MESH("Pulmonary arterial hypertension") OR MESH("Pacemaker, Artificial/adverse effects") OR MESH("Brain Neoplasms") OR MESH("Melanoma") OR MESH("Thyroid Diseases") OR MESH("Adrenal Gland Diseases") OR MESH("Pheochromocytoma") OR MESH("Diabetes Mellitus") OR MESH("Hyperpituitarism") OR MESH("Diabetes Insipidus") OR MESH("Obesity") OR MESH("Lung diseases , Obstructive") OR MESH("Sleep Apnea Syndromes") OR MESH("Cystic Fibrosis") OR MESH("Lung Diseases, Interstitial") OR MESH("Rhinitis, Atrophic") OR MESH("Ethmoid Sinusitis") OR MESH("Vertigo") OR MESH("Meniere Disease") OR MESH("Nasal Polyps") OR MESH("Otosclerosis") OR MESH("Colonic polyps") OR MESH("Stomach diverticulum") OR MESH("Gastritis, Atrophic") OR MESH("Gastroesophageal Reflux") OR MESH("Esophageal Achalasia") OR MESH("Hernia, Hiatal") OR MESH("Colonic Diseases, Functional") OR MESH("Celiac disease") OR MESH("Colitis, Ulcerative") OR MESH("Crohn Disease") OR MESH("Liver Cirrhosis") OR MESH("Back pain ") OR MESH("Neck Pain") OR MESH("Intervertebral Disc Displacement") OR MESH("Arthitris") OR MESH("Sciatica") OR MESH("Axial Spondyloarthritis") OR MESH("Osteitis Deformans") OR MESH("Bursitis") OR MESH("Fatigue Syndrome, Chronic") OR MESH("Crystal Arthropathies") OR MESH("Scoliosis") OR MESH("Reflex Sympathetic Dystrophy") OR MESH("Headache Disorders") OR MESH("Multiple sclerosis") OR MESH("Epilepsy") OR MESH("Alzheimer Disease") OR MESH("Dementia, Vascular") OR MESH("Parkinson Disease") OR MESH("Trigeminal Neuralgia") OR MESH("Guillain-Barre Syndrome") OR MESH("Stroke") OR MESH("Ischemic Attack, Transient") OR MESH("Muscular Diseases") OR MESH("Myasthenia Gravis") OR MESH("Kidney Failure, Chronic") OR MESH("Nephritis") OR MESH("Polycystic Kidney Diseases") OR MESH("Kidney Calculi") OR MESH("Acne Vulgaris") OR MESH("Rosacea") OR MESH("Eczema") OR MESH("Urticaria") OR MESH("Psoriasis") OR MESH("Vitiligo") OR MESH("Alopecia") OR MESH("Hidradenitis Suppurativa") OR MESH("Linear IgA Bullous Dermatosis") OR MESH("Herpes Simplex") OR MESH("Macular Degeneration") OR MESH("Low Tension Glaucoma") OR MESH("Cataract") OR MESH("Retinal Diseases") OR MESH("Vitreous Detachment") OR MESH("Vision Disorders") OR MESH("Uveitis") OR MESH("Scleritis") OR MESH("Lupus Erythematosus, Systemic"

) OR MESH("Antiphospholipid Syndrome") OR MESH("Scleroderma, Systemic") OR MESH("Granulomatosis with Polyangiitis") OR MESH("Microscopic Polyangiitis") OR MESH("Churg-Strauss Syndrome") OR MESH("Cryoglobulinemia") OR MESH("Arteritis") OR MESH("Sarcoidosis") OR MESH("Histiocytosis") OR MESH("Ehlers-Danlos Syndrome") OR MESH("Tuberculosis") OR MESH("Acquired Immunodeficiency Syndrome") OR MESH("Hepatitis, Chronic") OR MESH("Lyme Disease") OR MESH("Discitis") OR MESH("Leukemia") OR MESH("Hodgkin Disease") OR MESH("Lymphoma, Large B-Cell, Diffuse") OR MESH("Leukemia, Prolymphocytic, T-Cell") OR MESH("Leukemia") OR MESH("Leukemia, Myelogenous, Chronic, BCR-ABL Positive") OR MESH("Multiple Myeloma") OR MESH("Down Syndrome") OR MESH("Myeloproliferative Syndrome, Transient") OR MESH("Myelodysplastic Syndromes") OR MESH("Anemia, Hemolytic") OR MESH("Anemia, Pernicious") OR MESH("Purpura, Thrombocytopenic") OR MESH("Thrombotic Microangiopathies") OR MESH("Hemochromatosis ") OR MESH("Porphyrias") OR MESH("Hemophilia A") OR MESH("Fatigue Syndrome, Chronic") OR MESH("Chronic Pain") OR TIAB("Basal Cell Epithelioma") OR TIAB("Basal Cell Epitheliomas") OR TIAB("Brain Metastase") OR TIAB("Brain Metastases") OR TIAB("cerebroma") OR TIAB("Colon Adenocarcinoma") OR TIAB("Colon Adenocarcinomas") OR TIAB("dermatoma") OR TIAB("encephalophyma") OR TIAB("hypophysoma") OR TIAB("kidney mass") OR TIAB("kidney neoplastic mass") OR TIAB("malignant melanomatosis") OR TIAB("melanocarcinoma") OR TIAB("Melanoma") OR TIAB("melanomalignoma") OR TIAB("Melanomas") OR TIAB("naevocarcinoma") OR TIAB("neoplastic lung") OR TIAB("neoplastic mammary gland") OR TIAB("nephroma") OR TIAB("nevocarcinoma") OR TIAB("Pituitary Adenoma") OR TIAB("Pituitary Adenomas") OR TIAB("rectal mass") OR TIAB("rectum mass") OR TIAB("renal mass") OR TIAB("renal neoplastic mass") OR TIAB("reninoma") OR TIAB("Rodent Ulcer") OR TIAB("Rodent Ulcers") OR TIAB("squamous cell epithelioma") OR TIAB("squamous epithelioma") OR TIAB("Thyroid Adenoma") OR TIAB("Thyroid Adenomas") OR TIAB("angina") OR TIAB("anginal attack") OR TIAB("angiocardiopathy") OR TIAB("angiocardiovascular disease") OR TIAB("angiocardiovascular diseases") OR TIAB("Angor Pectori") OR TIAB("Aortic Valve Diseases") OR TIAB("Aortic Valve Disorder") OR TIAB("Aortic Valve Disorders") OR TIAB("Arrhythmia") OR TIAB("Arrythmia") OR TIAB("Artificial Cardiac Pacemaker") OR TIAB("Artificial Cardiac Pacemakers") OR TIAB("Artificial Pacemaker") OR TIAB("Artificial Pacemakers") OR TIAB("Atrial Fibrillation") OR TIAB("Atrial Fibrillations") OR TIAB("Auricular Fibrillation") OR TIAB("Auricular Fibrillations") OR TIAB("backward failure heart") OR TIAB("backward failure, heart") OR TIAB("Cardiac Arrest") OR TIAB("Cardiac Arrhythmias") OR TIAB("cardiac backward failure") OR TIAB("cardiac conduction system disease") OR TIAB("cardiac decompensation") OR TIAB("Cardiac Disorders") OR TIAB("cardiac disrhythmia") OR TIAB("Cardiac Dysrhythmia") OR TIAB("cardiac failure") OR TIAB("cardiac failures") OR TIAB("cardiac incompetence") OR TIAB("cardiac incompetences") OR TIAB("cardiac infarct") OR TIAB("cardiac insufficiencies") OR TIAB("cardiac insufficiency") OR TIAB("cardiac stand still") OR TIAB("cardiac valve defect") OR TIAB("cardiac valve disease") OR TIAB("cardial decompensation") OR TIAB("cardial infarct") OR TIAB("cardial insufficiency") OR TIAB("cardial valve disease") OR TIAB("cardiovascular complication") OR TIAB("cardiovascular complications") OR TIAB("cardiovascular decompensation") OR TIAB("cardiovascular disease") OR TIAB("cardiovascular diseases") OR TIAB("cardiovascular disorder") OR TIAB("cardiovascular disorders") OR TIAB("cardiovascular disturbance") OR TIAB("cardiovascular disturbances") OR TIAB("cardiovascular disturbancescardiovascular lesion") OR TIAB("cardiovascular disturbancescardiovascular lesions") OR TIAB("cardiovascular failure") OR TIAB("cardiovascular failures") OR TIAB("cardiovascular incompetence") OR TIAB("cardiovascular incompetences") OR TIAB("cardiovascular insufficiency") OR TIAB("cardiovascular insufficiencies") OR TIAB("cardiovascular lesion") OR TIAB("cardiovascular lesions") OR TIAB("cardiovascular syndrome") OR TIAB("cardiovascular syndromes") OR TIAB("cardiovascular vegetative disorder") OR TIAB(

"cardiovascular vegetative disorders") OR TIAB("cholesteremia") OR TIAB("cholesterinemia") OR TIAB("cholesterolemia") OR TIAB("Circulatory Disorders") OR TIAB("complication cardiovascular") OR TIAB("conduction defect") OR TIAB("conduction defects") OR TIAB("conduction disease") OR TIAB("conduction diseases") OR TIAB("conduction disorder") OR TIAB("conduction disorders") OR TIAB("conduction disturbance") OR TIAB("conduction disturbances") OR TIAB("Coronary Disorders") OR TIAB("decompensatio cordis") OR TIAB("decompensation, heart") OR TIAB("ectopic heart rhythm") OR TIAB("ectopic rhythm") OR TIAB("Elevated Cholesterol") OR TIAB("Elevated Cholesterols") OR TIAB("heart aberrant conduction") OR TIAB("heart arrhytmia") OR TIAB("Heart Attack") OR TIAB("Heart Attacks") OR TIAB("heart backward failure") OR TIAB("heart decompensation") OR TIAB("heart disease") OR TIAB("heart diseases") OR TIAB("heart disorder") OR TIAB("heart disorders") OR TIAB("heart dysrhythmia") OR TIAB("heart ectopic beat") OR TIAB("heart ectopic ventricle contraction") OR TIAB("heart failure") OR TIAB("heart failures") OR TIAB("heart incompetence") OR TIAB("heart infarct") OR TIAB("heart insufficiencies") OR TIAB("heart insufficiency") OR TIAB("heart rhythm disorder") OR TIAB("heart valve abnormalities") OR TIAB("heart valve abnormality") OR TIAB("heart valve defect") OR TIAB("heart valve degeneration") OR TIAB("Heart Valve Disease") OR TIAB("heart valve diseases") OR TIAB("heart valve lesion") OR TIAB("Heart Valvular Disease") OR TIAB("Heart Valvular Diseases") OR TIAB("High Blood Pressure") OR TIAB("High Blood Pressures") OR TIAB("High Cholesterol Level") OR TIAB("High Cholesterol Levels") OR TIAB("Hypercholesteremia") OR TIAB("Hypercholesteremias") OR TIAB("hypercholesterinaemia") OR TIAB("hypercholesterinemia") OR TIAB("hypercholesterolaemia") OR TIAB("Hypercholesterolemias") OR TIAB("hypertensive disease") OR TIAB("hypertensive effect") OR TIAB("hypertensive pulmonary vascular disease") OR TIAB("hypertensive response") OR TIAB("insufficientia cardis") OR TIAB("insufficientio cardis") OR TIAB("lung embolism") OR TIAB("lung embolization") OR TIAB("lung embolus") OR TIAB("lung emboly") OR TIAB("lung microembolism") OR TIAB("lung microembolization") OR TIAB("lung microembolus") OR TIAB("lung thromboembolism") OR TIAB("major adverse cardiovascular event") OR TIAB("major adverse cardiovascular events") OR TIAB("Myocardial Failure") OR TIAB("Myocardial Infarct") OR TIAB("myocardial insufficiency") OR TIAB("Myocardial lnfarcts") OR TIAB("myocardium infarct") OR TIAB("Periphlebitides") OR TIAB("Periphlebitis") OR TIAB("perivenous infection") OR TIAB("Phlebitides") OR TIAB("phlebitis superficialis") OR TIAB("post phlebitis syndrome") OR TIAB("postphlebitic disease") OR TIAB("postphlebitis syndrome") OR TIAB("Pulmonary Embolism") OR TIAB("Pulmonary Embolisms") OR TIAB("pulmonary embolization") OR TIAB("pulmonary embolus") OR TIAB("pulmonary hypertensive diseases") OR TIAB("pulmonary hypertensive disorder") OR TIAB("pulmonary hypertensive disorders") OR TIAB("pulmonary microembolism") OR TIAB("pulmonary thromboembolic disease") OR TIAB("Pulmonary Thromboembolism") OR TIAB("Pulmonary Thromboembolisms") OR TIAB("Raynauds Disease") OR TIAB("Stenocardia") OR TIAB("Stenocardias") OR TIAB("superficial phlebitis") OR TIAB("valvulopathies") OR TIAB("valvulopathy") OR TIAB("Vascular Disorders") OR TIAB("Vasculitis") OR TIAB("vein inflammation") OR TIAB("venitis") OR TIAB("venous inflammation") OR TIAB("vitium cordis") OR TIAB("Chronic Infectious Mononucleosis-Like Syndromes") OR TIAB("chronic intractable pain") OR TIAB("Royal Free Diseases") OR TIAB("Systemic Exertion Intolerance Diseases") OR TIAB("Chronic Pain") OR TIAB("Chronic Pains") OR TIAB("Achalasia") OR TIAB("Achalasias") OR TIAB("alcohol liver injury") OR TIAB("alcoholic liver") OR TIAB("Atrophic Gastritides") OR TIAB("Atrophic Gastritis") OR TIAB("autoimmune gastritis") OR TIAB("bowel syndrome") OR TIAB("cardiac herniation") OR TIAB("cardioesophageal reflux") OR TIAB("cardiooesophageal reflux") OR TIAB("Cardiospasm") OR TIAB("Cardiospasms") OR TIAB("Celiac Disease") OR TIAB("celiac syndrome") OR TIAB("chronic inflammatory bowel diseases") OR TIAB("chronic ulceration colon") OR TIAB("cirrhosis") OR TIAB("cleron disease") OR TIAB("coeliac disease") OR TIAB("coeliac syndrome") OR TIAB("coeliaky") OR

TIAB("Colitis Gravis") OR TIAB("colitis ulcerativa") OR TIAB("colitis ulcerosa") OR TIAB("colon disease") OR TIAB("colon diseases") OR TIAB("colon disorder") OR TIAB("colon disorders") OR TIAB("colon juvenile polyp") OR TIAB("colon polypoid lesion") OR TIAB("colon spasm") OR TIAB("chronic colon ulceration") OR TIAB("Colonic Polyp") OR TIAB("Colonic Polyps") OR TIAB("colonospasm") OR TIAB("Crohns Disease") OR TIAB("Crohn's Disease") OR TIAB("Crohn's Enteritis") OR TIAB("digestive disease") OR TIAB("digestive diseases") OR TIAB("digestive disorder") OR TIAB("digestive disorders") OR TIAB("digestive system disorders") OR TIAB("enteritis regionalis") OR TIAB("Esophageal Hernia") OR TIAB("Esophageal Hernias") OR TIAB("Esophageal Reflux") OR TIAB("esophageal regurgitation") OR TIAB("esophagogastric reflux") OR TIAB("esophagus hernia") OR TIAB("esophagus reflux") OR TIAB("functional colonic diseases") OR TIAB("Gastric Acid Reflux") OR TIAB("gastric atrophy") OR TIAB("gastric regurgitation") OR TIAB("Gastroduodenal Ulcer") OR TIAB("Gastroduodenal Ulcers") OR TIAB("gastroesophageal reflex") OR TIAB("gastroesophageal regurgitation") OR TIAB("gastroesophagus reflux") OR TIAB("gastrointestinal disease") OR TIAB("gastrointestinal diseases") OR TIAB("gastrointestinal disorder") OR TIAB("gastrointestinal disorders") OR TIAB("gastrointestinal dysfunctioning") OR TIAB("gastrointestinal dysfunction") OR TIAB("gastrointestinal dysfunctions") OR TIAB("gastrooesophageal reflex") OR TIAB("gastrooesophageal reflux") OR TIAB("Gastro-oesophageal Reflux") OR TIAB("gastrooesophageal regurgitation") OR TIAB("gee herter disease") OR TIAB("gee thaysen disease") OR TIAB("GERD") OR TIAB("Gluten Enteropathies") OR TIAB("Gluten Enteropathy") OR TIAB("gluten induced enteropathy") OR TIAB("gluten intolerance") OR TIAB("Gluten Sensitive Enteropathy") OR TIAB("Gluten-Sensitive Enteropathies") OR TIAB("Gluten-Sensitive Enteropathy") OR TIAB("Granulomatous Colitis") OR TIAB("Granulomatous Enteritis") OR TIAB("hernia hiatus esophagi") OR TIAB("hiatal diaphragmatic hernia") OR TIAB("hiatal esophageal herniation") OR TIAB("Hiatal Hernia") OR TIAB("Hiatal Hernias") OR TIAB("hiatal herniation") OR TIAB("hiatus diaphragmatic hernia") OR TIAB("hiatus esophagi hernia") OR TIAB("Hiatus Hernia") OR TIAB("Hiatus Hernias") OR TIAB("hiatus herniation") OR TIAB("hiatus oesophageal hernia") OR TIAB("hiatus oesophagus hernia") OR TIAB("huebner herter disease") OR TIAB("Idiopathic Proctocolitis") OR TIAB("Ileocolitis") OR TIAB("Inflammatory Bowel Disease") OR TIAB("intestinal infantilism") OR TIAB("Irritable Bowel Syndromes") OR TIAB("Irritable Colon") OR TIAB("Liver Fibrosis") OR TIAB("Marginal Ulcer") OR TIAB("Marginal Ulcers") OR TIAB("Megaesophagus") OR TIAB("morbus crohn") OR TIAB("mucomembraneous colitis") OR TIAB("mucomembranous colitis") OR TIAB("mucosal colitis") OR TIAB("Mucous Colitides") OR TIAB("Mucous Colitis") OR TIAB("oesophageal reflux") OR TIAB("oesophageal regurgitation") OR TIAB("oesophagogastric reflux") OR TIAB("oesophagus reflux") OR TIAB("Paraesophageal Hernia") OR TIAB("para-esophageal hernia") OR TIAB("Paraesophageal Hernias") OR TIAB("paraesophageal herniation") OR TIAB("para-esophageal herniation") OR TIAB("paraoesophageal hernia") OR TIAB("para-oesophageal hernia") OR TIAB("paraoesophageal herniation") OR TIAB("para-oesophageal herniation") OR TIAB("Peptic Ulcers") OR TIAB("Regional Enteritis") OR TIAB("regional enterocolitis") OR TIAB("Regional Ileitides") OR TIAB("Regional Ileitis") OR TIAB("regurgitation, gastroesophageal") OR TIAB("spastic colitis") OR TIAB("spastic colon") OR TIAB("Sprue") OR TIAB("stomach hernia") OR TIAB("Terminal Ileitis") OR TIAB("Ulcerative Colitis") OR TIAB("ulcerative colorectitis") OR TIAB("ulcerative procto colitis") OR TIAB("ulcerative proctocolitis") OR TIAB("ulcerous colitis") OR TIAB("unstable colon") OR TIAB("acromegalia") OR TIAB("Acromegalies") OR TIAB("acromegalism") OR TIAB("Acromegaly") OR TIAB("Addison Disease") OR TIAB("Addisons Disease") OR TIAB("Addison's Disease") OR TIAB("adipose tissue hyperplasia") OR TIAB("adipositas") OR TIAB("adiposity") OR TIAB("adrenal cortex hyperplasia") OR TIAB("adrenal cortical hyperplasia") OR TIAB("adrenal failure") OR TIAB("adrenal failures") OR TIAB("adrenal gland disorders") OR TIAB("adrenocortical hyperplasia") OR TIAB("adrenocorticohyperplasia") OR TIAB("akromegalia") OR

TIAB("Aldosteronism") OR TIAB("antidiuretic hormone insufficiency") OR TIAB("arenocortical hyperplasia") OR TIAB("Basedow Disease") OR TIAB("basedow syndrome") OR TIAB("Basedows Disease") OR TIAB("Basedow's Disease") OR TIAB("chromaffin paraganglioma") OR TIAB("chronic autoimmune thyroiditis") OR TIAB("Chronic Lymphocytic Thyroiditides") OR TIAB("Chronic Lymphocytic Thyroiditis") OR TIAB("Conn Syndrome") OR TIAB("Conns Syndrome") OR TIAB("Conn's Syndrome") OR TIAB("corpulency") OR TIAB("Cushing syndrome") OR TIAB("Cushings syndrome") OR TIAB("Cushing's Syndrome") OR TIAB("diabetes") OR TIAB("diabetic") OR TIAB("endocrinal disease") OR TIAB("endocrinal diseases") OR TIAB("endocrinal disorder") OR TIAB("endocrinal disorders") OR TIAB("endocrinal disturbance") OR TIAB("endocrinal disturbances") OR TIAB("endocrinal dysfunctioning") OR TIAB("endocrinal dysfunction") OR TIAB("endocrinal dysfunctions") OR TIAB("endocrine disease") OR TIAB("endocrine diseases") OR TIAB("endocrine disorder") OR TIAB("Endocrine Disorders") OR TIAB("endocrine disturbance") OR TIAB("endocrine disturbances") OR TIAB("endocrine dysfunctioning") OR TIAB("endocrine dysfunction") OR TIAB("endocrine dysfunctions") OR TIAB("endocrine gland disease") OR TIAB("endocrine gland diseases") OR TIAB("endocrine gland disorder") OR TIAB("endocrine gland disorders") OR TIAB("endocrine gland dysfunctioning") OR TIAB("endocrine gland dysfunction") OR TIAB("endocrine gland dysfunctions") OR TIAB("endocrine syndrome") OR TIAB("endocrine syndromes") OR TIAB("endocrine system disease") OR TIAB("endocrine system diseases") OR TIAB("endocrine system disorder") OR TIAB("endocrine system disorders") OR TIAB("endocrine system dysfunctioning") OR TIAB("endocrine system dysfunction") OR TIAB("endocrine system dysfunctions") OR TIAB("endocrinological disease") OR TIAB("endocrinological diseases") OR TIAB("endocrinological disorder") OR TIAB("endocrinological disorders") OR TIAB("endocrinological dysfunctioning") OR TIAB("endocrinological dysfunction") OR TIAB("endocrinological dysfunctions") OR TIAB("excess body weight") OR TIAB("Exophthalmic Goiter") OR TIAB("Exophthalmic Goiters") OR TIAB("exophthalmic goitre") OR TIAB("exophthalmic hyperthyroidism") OR TIAB("Extra Adrenal Pheochromocytoma") OR TIAB("Extra-Adrenal Pheochromocytoma") OR TIAB("familial hypoadrenocorticism") OR TIAB("fat overload syndrome") OR TIAB("feline hyperthyroidism") OR TIAB("Graves Disease") OR TIAB("Graves' Disease") OR TIAB("Graves hyperthyroidism") OR TIAB("graves syndrome") OR TIAB("Graves's disease") OR TIAB("Hashimoto Disease") OR TIAB("hashimoto goiter") OR TIAB("Hashimoto Struma") OR TIAB("Hashimoto Syndrome") OR TIAB("Hashimoto Thyroiditides") OR TIAB("Hashimoto Thyroiditis") OR TIAB("hashimoto thyroidosis") OR TIAB("Hashimotos Disease") OR TIAB("Hashimoto's Disease") OR TIAB("Hashimoto's Struma") OR TIAB("Hashimotos Syndrome") OR TIAB("Hashimoto's Syndrome") OR TIAB("Hashimoto's Syndromes") OR TIAB("Hashimotos thyroiditis") OR TIAB("Hashimoto's thyroiditis") OR TIAB("hormonal disease") OR TIAB("hormonal diseases") OR TIAB("hormonal disorder") OR TIAB("hormonal disorders") OR TIAB("hormonal dysfunctioning") OR TIAB("hormonal dysfunction") OR TIAB("hormonal dysfunctions") OR TIAB("hormone imbalance") OR TIAB("hyperaldosterone") OR TIAB("Hypercortisolism") OR TIAB("hypermineralocorticidism") OR TIAB("hypermineralocorticism") OR TIAB("Hyperprolactinaemia") OR TIAB("Hyperprolactinaemias") OR TIAB("Hyperprolactinemia") OR TIAB("Hyperprolactinemias") OR TIAB("hyperthyreoidism") OR TIAB("hyperthyreosis") OR TIAB("Hyperthyroid") OR TIAB("hyperthyroidea") OR TIAB("hyperthyroidosis") OR TIAB("Hyperthyroids") OR TIAB("hypogonadism") OR TIAB("hypothyreoidism") OR TIAB("hypothyreosis") OR TIAB("hypothyroidea") OR TIAB("hypothyroidism") OR TIAB("Hypothyroidisms") OR TIAB("hypothyroidosis") OR TIAB("hypothyrosis") OR TIAB("Inappropriate GH Secretion Syndrome") OR TIAB("Inappropriate GH Secretion Syndromes") OR TIAB("Inappropriate Growth Hormone Secretion Syndrome") OR TIAB("Inappropriate Growth Hormone Secretion Syndromes") OR TIAB("Inappropriate Prolactin Secretion") OR TIAB("Inappropriate Secretion Prolactin") OR TIAB("lymphadenoid goiter") OR

TIAB("megalakria") OR TIAB("mineralcorticoid excess syndrome") OR TIAB("obesitas") OR TIAB("obesity") OR TIAB("overweight") OR TIAB("phaeochromoblastoma") OR TIAB("phaeochromocytoma") OR TIAB("pheochromoblastoma") OR TIAB("Pheochromocytomas") OR TIAB("pheochromocytomata") OR TIAB("pheochromocytomatosis") OR TIAB("pheochromocytosis") OR TIAB("polycystic ovary syndrome") OR TIAB("polycystic ovary syndromes") OR TIAB("Primary Adrenal Insufficiency") OR TIAB("Primary Adrenocortical Insufficiencies") OR TIAB("Primary Adrenocortical Insufficiency") OR TIAB("Primary Hyperaldosteronism") OR TIAB("Primary Hyperthyroidism") OR TIAB("Primary Hypoadrenalism") OR TIAB("Primary Hypoadrenalisms") OR TIAB("Prolactin Hypersecretion Syndrome") OR TIAB("Prolactin Hypersecretion Syndromes") OR TIAB("Somatotropin Hypersecretion Syndrome") OR TIAB("Somatotropin Hypersecretion Syndromes") OR TIAB("struma hashimoto") OR TIAB("thyroid deficiency") OR TIAB("thyroid gland failure") OR TIAB("thyroid gland hyperfunction") OR TIAB("thyroid hyperfunction") OR TIAB("thyroid insufficiency") OR TIAB("Thyroid Stimulating Hormone Deficiency") OR TIAB("thyroideal hyperfunction") OR TIAB("Thyroid-Stimulating Hormone Deficiencies") OR TIAB("Thyroid-Stimulating Hormone Deficiency") OR TIAB("TSH Deficiencies") OR TIAB("TSH Deficiency") OR TIAB("Vasopressin Deficiency") OR TIAB("angioleiomyoma") OR TIAB("angiomyoma") OR TIAB("elastomyofibroma") OR TIAB("fibromyoma") OR TIAB("fibromyomas") OR TIAB("hemangioleiomyoma") OR TIAB("hemangiomyoma") OR TIAB("Impotence") OR TIAB("leimyoma") OR TIAB("Leiomyomas") OR TIAB("leyomyoma") OR TIAB("myofibroma") OR TIAB("myofibromatosis") OR TIAB("vascular leiomyoma") OR TIAB("addison anaemia") OR TIAB("addison anemia") OR TIAB("addisonian anaemia") OR TIAB("addisonian anemia") OR TIAB("ahf deficiency") OR TIAB("ahg deficiency") OR TIAB("anaemia perniciosa") OR TIAB("anemia perniciosa") OR TIAB("B12 deficiency anaemia") OR TIAB("B12 deficiency anemia") OR TIAB("B12 deficient anaemia") OR TIAB("B12 deficient anemia") OR TIAB("B12 vitamin deficiency anemia") OR TIAB("biermer anaemia") OR TIAB("biermer anemia") OR TIAB("biermer disease") OR TIAB("blood disease") OR TIAB("blood diseases") OR TIAB("blood disorder") OR TIAB("blood disorders") OR TIAB("blood dysfunctioning") OR TIAB("blood dysfunction") OR TIAB("blood dysfunctions") OR TIAB("classic haemophilia") OR TIAB("cobalamin deficiency anaemia") OR TIAB("cobalamin deficiency anemia") OR TIAB("congenital antihaemophilic factor deficiency") OR TIAB("congenital antihaemophilic globulin deficiency") OR TIAB("congenital antihemophilic factor deficiency") OR TIAB("congenital antihemophilic globulin deficiency") OR TIAB("drepanocytemia") OR TIAB("drepanocytic anaemia") OR TIAB("drepanocytic anemia") OR TIAB("drepanocytosis") OR TIAB("haematologic disease") OR TIAB("haematologic diseases") OR TIAB("haematologic disorder") OR TIAB("haematologic disorders") OR TIAB("haematologic dysfunctioning") OR TIAB("haematologic dysfunction") OR TIAB("haematologic dysfunctions") OR TIAB("haematological disease") OR TIAB("haematological diseases") OR TIAB("haematological disorder") OR TIAB("haematological disorders") OR TIAB("haematological dysfunctioning") OR TIAB("haematological dysfunction") OR TIAB("haematological dysfunctions") OR TIAB("haemochromatosis") OR TIAB("haemoglobin SS") OR TIAB("haemolytic anaemia") OR TIAB("haemolytic disease") OR TIAB("haemolytic syndrome") OR TIAB("haemophilia a") OR TIAB("haemophilia vera") OR TIAB("Hb SS disease") OR TIAB("heart hemochromatosis") OR TIAB("hemachromatosis") OR TIAB("hematologic disease") OR TIAB("hematologic diseases") OR TIAB("hematologic disorder") OR TIAB("hematologic disorders") OR TIAB("hematologic dysfunctioning") OR TIAB("hematologic dysfunction") OR TIAB("hematologic dysfunctions") OR TIAB("hematopathy") OR TIAB("hemoglobin SS") OR TIAB("hemolytic anemia") OR TIAB("hemolytic disease") OR TIAB("hemolytic syndrome") OR TIAB("hemopathie") OR TIAB("hemopathies") OR TIAB("hemopathy") OR TIAB("hemophylia type a") OR TIAB("hereditary iron overload") OR TIAB("idiopathic hemochromatosis") OR TIAB("intermittent porphyria") OR TIAB("iron overload disease") OR TIAB("iron overload disorder") OR TIAB("iron overload syndrome") OR

TIAB("latent porphyria") OR TIAB("lymphatic disease") OR TIAB("lymphatic diseases") OR TIAB("lymphatic disorder") OR TIAB("lymphatic disorders") OR TIAB("lymphatic dysfunctioning") OR TIAB("lymphatic dysfunction") OR TIAB("lymphatic dysfunctions") OR TIAB("macrocytic hyperchromic anaemia") OR TIAB("macrocytic hyperchromic anemia") OR TIAB("meniscocytosis") OR TIAB("pernicious anaemia") OR TIAB("pernicious anemia") OR TIAB("porphyrias") OR TIAB("porphyric disease") OR TIAB("primary anaemia") OR TIAB("primary anemia") OR TIAB("primary congenital hemochromatosis") OR TIAB("recklinghausen applebaum disease") OR TIAB("sickle anaemia") OR TIAB("sickle anemia") OR TIAB("sickle cell anaemia") OR TIAB("siderochromatosis") OR TIAB("sulfhemoglobinem") OR TIAB("sulfhemoglobinems") OR TIAB("sulphaemoglobinaem") OR TIAB("sulphaemoglobinaems") OR TIAB("systemic porphyria") OR TIAB("thrombocytopaenia purpura") OR TIAB("thrombocytopenia purpura") OR TIAB("thrombotic purpura") OR TIAB("true haemophilia") OR TIAB("vitamin b 12 deficiency anaemia") OR TIAB("vitamin b 12 deficiency anemia") OR TIAB("a2 myeloma") OR TIAB("Acquired Hemolytic Anemias") OR TIAB("Acquired Immune Deficiency Syndrome Virus") OR TIAB("acquired immunodeficiency") OR TIAB("Addison Anemias") OR TIAB("Addisons Anemia") OR TIAB("Addison's Anemias") OR TIAB("AIDS") OR TIAB("aleukaemia") OR TIAB("aleukemia") OR TIAB("Arthritides") OR TIAB("arthrochondritis") OR TIAB("arthrosynovitis") OR TIAB("B. burgdorferi Infection") OR TIAB("B. burgdorferi Infections") OR TIAB("bone marrow dysplasia") OR TIAB("Bone Marrow Fibrosis") OR TIAB("Bone Marrow Fibroses") OR TIAB("Borrelia burgdorferi Infection") OR TIAB("Borrelia burgdorferi Infections") OR TIAB("Bronze Diabete") OR TIAB("Bronzed Cirrhoses") OR TIAB("Chronic Hepatitis") OR TIAB("Chronic Hepatitides") OR TIAB("Chronic Hepatic") OR TIAB("chronic myeloleukaemia") OR TIAB("chronic myeloleukemia") OR TIAB("Discitides") OR TIAB("discitis") OR TIAB("disk space infection") OR TIAB("Diskitis") OR TIAB("Diskitides") OR TIAB("DLBCL") OR TIAB("down disease") OR TIAB("Downs syndrome") OR TIAB("Down's syndrome") OR TIAB("Dysmyelopoietic Syndrome") OR TIAB("Dysmyelopoietic Syndromes") OR TIAB("endocardial inflammation") OR TIAB("Endocarditis") OR TIAB("Endocarditides") OR TIAB("endo-carditis") OR TIAB("endocardium inflammation") OR TIAB("Erythremia") OR TIAB("Erythremias") OR TIAB("Factor 8 Deficiency") OR TIAB("Factor 8 Deficiencies") OR TIAB("Factor VIII Deficiency") OR TIAB("Factor VIII Deficiencies") OR TIAB("Factor VIII defiency") OR TIAB("Familial Hemochromatoses") OR TIAB("Familial Hemochromatosis") OR TIAB("Haemochromato") OR TIAB("Haemochromatos") OR TIAB("Haemolytic Anaemias") OR TIAB("HbS Disease") OR TIAB("Hematopoetic Myelodysplasias") OR TIAB("hemoblastoma") OR TIAB("Hemochromato") OR TIAB("Hemochromatos") OR TIAB("Hemoglobin S Disease") OR TIAB("Hemoglobin S Diseases") OR TIAB("Hemophilia") OR TIAB("hemophilias") OR TIAB("high-risk MDS") OR TIAB("Hodgkin disease") OR TIAB("Hodgkin diseases") OR TIAB("Hodgkin Granuloma") OR TIAB("Hodgkin Granulomas") OR TIAB("hodgkin sclerosis") OR TIAB("hodgkin's disease") OR TIAB("Hodgkins Disease") OR TIAB("Hodgkins Diseases") OR TIAB("Hodgkins Granuloma") OR TIAB("Hodgkin's Granuloma") OR TIAB("HTLV-III") OR TIAB("Human Immunodeficiency Virus") OR TIAB("Human Immunodeficiency Viruses") OR TIAB("Human T Cell Lymphotropic Virus Type III") OR TIAB("Human T Lymphotropic Virus Type III") OR TIAB("Human T-Cell Lymphotropic Virus Type III") OR TIAB("Human T-Lymphotropic Virus Type III") OR TIAB("infection by B. burgdorferi") OR TIAB("infection by Borrelia burgdorferi") OR TIAB("intervertebral disc infection") OR TIAB("intervertebral disk infection") OR TIAB("intervertebral disk inflammation") OR TIAB("Iron Storage Disorder") OR TIAB("Iron Storage Disorders") OR TIAB("joint inflammation") OR TIAB("Koch Disease") OR TIAB("Koch Diseases") OR TIAB("Kochs Disease") OR TIAB("Kochs Diseases") OR TIAB("Koch's Disease") OR TIAB("Koch's Diseases") OR TIAB("langdon down syndrome") OR TIAB("LAV-HTLV-III") OR TIAB("leucaemia") OR TIAB("leucemia") OR TIAB("Leucocythaemia") OR TIAB("Leucocythaemias") OR TIAB("Leucocythemia") OR TIAB("Leucocythemias") OR TIAB("leukaemia") OR TIAB("Leukemia") OR TIAB("Leukemias") OR TIAB("Lyme borrelioses") OR TIAB("Lyme Borreliosis") OR TIAB("Lyme's

borrelioses") OR TIAB("Lyme's borreliosis") OR TIAB("Lymes disease") OR TIAB("Lyme's disease") OR TIAB("Lymphadenopathy Associated Virus") OR TIAB("Lymphadenopathy Associated Viruses") OR TIAB("Lymphadenopathy-Associated Virus") OR TIAB("Lymphadenopathy-Associated Viruses") OR TIAB("lymphogranuloma maligne") OR TIAB("lymphogranuloma malignum") OR TIAB("lymphogranulomatosis") OR TIAB("Malignant Granuloma") OR TIAB("Malignant Granulomas") OR TIAB("malignant granulomatosis") OR TIAB("Malignant Lymphogranuloma") OR TIAB("Malignant Lymphogranulomas") OR TIAB("Microangiopathic Anemias") OR TIAB("Microangiopathic Hemolytic Anemias") OR TIAB("mongolian idiocy") OR TIAB("mongolism") OR TIAB("mongoloid idiocy") OR TIAB("mongoloidism") OR TIAB("morbus hodgkin") OR TIAB("myelodysplasia") OR TIAB("myelodysplastic disease") OR TIAB("myelodysplastic disorder") OR TIAB("myelodysplastic syndromes") OR TIAB("Myelofibrosis") OR TIAB("Myelofibroses") OR TIAB("Myeloid Metaplasia") OR TIAB("Myeloid Metaplasias") OR TIAB("myeloplaxoma") OR TIAB("Myelosclerosis") OR TIAB("Myeloscleroses") OR TIAB("Nonleukemic Myelosis") OR TIAB("Nonleukemic Myeloses") OR TIAB("oligoarthritis") OR TIAB("Osler Vaquez Disease") OR TIAB("Osler Vaquez Diseases") OR TIAB("Osler-Vaquez Disease") OR TIAB("Osler-Vaquez Diseases") OR TIAB("Pernicious Anemias") OR TIAB("Pigmentary Cirrhoses") OR TIAB("Polyarthritis") OR TIAB("Polyarthritides") OR TIAB("Polycythemia Ruba Vera") OR TIAB("Polycythemia Ruba Veras") OR TIAB("Polycythemia Rubra Vera") OR TIAB("Polycythemia Rubra Veras") OR TIAB("Primary Hemochromatosis") OR TIAB("Primary Polycythemia") OR TIAB("Primary Polycythemias") OR TIAB("reed sternberg disease") OR TIAB("Sickle Cell Anemia") OR TIAB("Sickle Cell Anemias") OR TIAB("Sickle Cell Disease") OR TIAB("Sickle Cell Diseases") OR TIAB("sickle cell disorder") OR TIAB("Sickle Cell Disorders") OR TIAB("Sickling Disorder Due to Hemoglobin S") OR TIAB("Spondylodiscitis") OR TIAB("Spondylodiscitides") OR TIAB("Spondylodiskitis") OR TIAB("Spondylodiskitides") OR TIAB("TB disease") OR TIAB("TB infection") OR TIAB("Thrombocytopenic Purpura") OR TIAB("Thrombocytopenic Purpuras") OR TIAB("Thrombopenic Purpura") OR TIAB("Thrombopenic Purpuras") OR TIAB("Thrombotic Microangiopathy") OR TIAB("Thrombotic Microangiopathies") OR TIAB("translocation 15 21 22") OR TIAB("trisomy 21 syndrome") OR TIAB("Troisier Hanot Chauffard Syndrome") OR TIAB("Troisier-Hanot-Chauffard Syndrome") OR TIAB("Troisier-Hanot-Chauffard Syndromes") OR TIAB("Tuberculoses") OR TIAB("tuberculous infection") OR TIAB("tuberculous lesion") OR TIAB("Von Recklenhausen Applebaum Disease") OR TIAB("Von Recklenhausen-Applebaum Diseases") OR TIAB("Acute Autoimmune Neuropathies") OR TIAB("Acute Autoimmune Neuropathy") OR TIAB("acute cerebrovascular lesion") OR TIAB("acute febrile polyneuritis") OR TIAB("acute focal cerebral vasculopathy") OR TIAB("Acute Infectious Polyneuritis") OR TIAB("Acute Inflammatory Demyelinating Polyneuropathy") OR TIAB("Acute Inflammatory Demyelinating Polyneuropathies") OR TIAB("Acute Inflammatory Demyelinating Polyradiculoneuropathy") OR TIAB("Acute Inflammatory Demyelinating Polyradiculoneuropathies") OR TIAB("Acute Inflammatory Polyneuropathy") OR TIAB("Acute Inflammatory Polyneuropathies") OR TIAB("Acute Inflammatory Polyradiculoneuropathy") OR TIAB("Acute Inflammatory Polyradiculoneuropathies") OR TIAB("acute postinfective polyradiculoneuropathy") OR TIAB("Alzeimer") OR TIAB("Alzheimer") OR TIAB("Alzeimers disease") OR TIAB("Alzeimer's disease") OR TIAB("Alzheimer's Disease") OR TIAB("Alzheimers Disease") OR TIAB("Alzheimers Diseases") OR TIAB("Alzheimer's Diseases") OR TIAB("Alzheimer-Type Dementia") OR TIAB("Anti-MuSK Myasthenia Gravis") OR TIAB("Apoplexies") OR TIAB("apoplexia") OR TIAB("apoplexy") OR TIAB("Arteriosclerotic Dementias") OR TIAB("Aura") OR TIAB("Auras") OR TIAB("autoimmune myasthenia gravis") OR TIAB("Binswanger Disease") OR TIAB("Binswanger Diseases") OR TIAB("Binswanger Encephalopathies") OR TIAB("Binswanger Encephalopathy") OR TIAB("Binswangers Disease") OR TIAB("Binswangers Diseases") OR TIAB("Binswanger's Disease") OR TIAB("Binswanger's Diseases") OR TIAB("Binswangers Encephalopathy") OR TIAB("Binswangers Encephalopathies") OR TIAB("Binswanger's Encephalopathy") OR TIAB(

"Binswanger's Encephalopathies") OR TIAB("brain accident") OR TIAB("brain attack") OR TIAB("brain blood flow disturbance") OR TIAB("brain insult") OR TIAB("brain insultus") OR TIAB("Brain TIA") OR TIAB("Brain Vascular Accident") OR TIAB("Brain Vascular Accidents") OR TIAB("Cephalgia Syndrome") OR TIAB("Cephalgia Syndromes") OR TIAB("cerebral insult") OR TIAB("cerebral vascular accident") OR TIAB("cerebral vascular insufficiency") OR TIAB("cerebro vascular accident") OR TIAB("Cerebrovascular Accident") OR TIAB("Cerebrovascular Accidents") OR TIAB("cerebrovascular arrest") OR TIAB("cerebrovascular failure") OR TIAB("cerebrovascular injury") OR TIAB("cerebrovascular insufficiency") OR TIAB("cerebrovascular insult") OR TIAB("cerebrum vascular accident") OR TIAB("Chronic Daily Headache") OR TIAB("Chronic Daily Headaches") OR TIAB("Chronic Headache") OR TIAB("Chronic Headaches") OR TIAB("Chronic Progressive Subcortical Encephalopathy") OR TIAB("Chronic Progressive Subcortical Encephalopathies") OR TIAB("comitial disease") OR TIAB("congenital atonic sclerotic muscular dystrophy") OR TIAB("congenital muscular dystrophy") OR TIAB("CVA") OR TIAB("CVAs") OR TIAB("diffuse cortical sclerosis") OR TIAB("Disseminated Sclerosis") OR TIAB("Epilepsy") OR TIAB("Epilepsies") OR TIAB("epilepsia") OR TIAB("epileptic") OR TIAB("Epileptiform Neuralgia") OR TIAB("Epileptiform Neuralgias") OR TIAB("erb goldflam disease") OR TIAB("falling sickness") OR TIAB("Fisher syndrome") OR TIAB("Fothergill Disease") OR TIAB("Generalized Myasthenia Gravis") OR TIAB("Guillain Barre") OR TIAB("Guillain-Barré") OR TIAB("Guillain-Barre") OR TIAB("Guillain Barré Syndrome") OR TIAB("Guillain Barré Syndromes") OR TIAB("Headache Disorder") OR TIAB("Headache Syndrome") OR TIAB("Headache Syndromes") OR TIAB("Idiopathic Parkinson Diseases") OR TIAB("idiopathic parkinsonism") OR TIAB("infectious neuronitis") OR TIAB("inflammatory acute polyradiculoneuropathy") OR TIAB("Inflammatory Polyneuropathy Acute") OR TIAB("insular sclerosis") OR TIAB("insultus cerebralis") OR TIAB("Intractable Headache") OR TIAB("Intractable Headaches") OR TIAB("ischaemic attack") OR TIAB("ischaemic cerebral attack") OR TIAB("ischaemic seizure") OR TIAB("ischemic attack") OR TIAB("ischemic cerebral attack") OR TIAB("ischemic seizure") OR TIAB("lacunar dementia") OR TIAB("Landry paralysis") OR TIAB("Landry syndrome") OR TIAB("Landry-Guillain-Barre Syndrome") OR TIAB("Lewy Body Parkinson Diseases") OR TIAB("maternal myasthenia gravis") OR TIAB("mini-stroke") OR TIAB("multiinfarct dementia") OR TIAB("multi-infarct dementia") OR TIAB("multiinfarction dementia") OR TIAB("multi-infarction dementia") OR TIAB("multiple sclerosis") OR TIAB("muscle dystrophia") OR TIAB("muscle dystrophy") OR TIAB("Muscle Specific Receptor Tyrosine Kinase Myasthenia Gravis") OR TIAB("Muscle Specific Tyrosine Kinase Antibody Positive Myasthenia Gravis") OR TIAB("Muscle-Specific Receptor Tyrosine Kinase Myasthenia Gravis") OR TIAB("Muscle-Specific Tyrosine Kinase Antibody Positive Myasthenia Gravis") OR TIAB("muscular dystrophia") OR TIAB("muscular dystrophies") OR TIAB("MuSK MG") OR TIAB("MuSK Myasthenia Gravis") OR TIAB("myasthenia gravis pseudoparalitica") OR TIAB("myasthenia gravis pseudoparalytica") OR TIAB("myodystrophia") OR TIAB("myodystrophy") OR TIAB("neonatal myasthenia gravis") OR TIAB("nervous disease") OR TIAB("nervous diseases") OR TIAB("nervous disorder") OR TIAB("nervous disorders") OR TIAB("nervous dysfunctioning") OR TIAB("nervous dysfunction") OR TIAB("nervous dysfunctions") OR TIAB("nervous system disease") OR TIAB("nervous system diseases") OR TIAB("nervous system disorder") OR TIAB("nervous system disorders") OR TIAB("nervous system dysfunctioning") OR TIAB("nervous system dysfunction") OR TIAB("nervous system dysfunctions") OR TIAB("neurologic disease") OR TIAB("neurologic diseases") OR TIAB("neurologic disorder") OR TIAB("neurologic disorders") OR TIAB("neurologic dysfunctioning") OR TIAB("neurologic dysfunction") OR TIAB("neurologic dysfunctions") OR TIAB("neurological disease") OR TIAB("neurological diseases") OR TIAB("neurological disorder") OR TIAB("neurological disorders") OR TIAB("neurological dysfunctioning") OR TIAB("neurological dysfunction") OR TIAB("neurological dysfunctions") OR TIAB("neuromuscular disease") OR TIAB("neuromuscular diseases") OR TIAB("neuromuscular

disorder") OR TIAB("neuromuscular disorders") OR TIAB("neuromuscular dysfunctioning") OR TIAB("neuromuscular dysfunction") OR TIAB("neuromuscular dysfunctions") OR TIAB("Ocular Myasthenia Gravis") OR TIAB("Paralysis Agitans") OR TIAB("Parkinson dementia complex") OR TIAB("Parkinsons disease") OR TIAB("Parkinson disease") OR TIAB("Parkinson's Disease") OR TIAB("Parkinson's Diseases") OR TIAB("Presenile Dementia") OR TIAB("Primary Parkinsonism") OR TIAB("Primary Senile Degenerative Dementia") OR TIAB("sclerosis multiplex") OR TIAB("Seizure Disorder") OR TIAB("Seizure Disorders") OR TIAB("Senile Dementia") OR TIAB("stroke") OR TIAB("Strokes") OR TIAB("Subcortical Arteriosclerotic Encephalopathy") OR TIAB("Subcortical Arteriosclerotic Encephalopathies") OR TIAB("Subcortical Leukoencephalopathies") OR TIAB("Subcortical Leukoencephalopathy") OR TIAB("Tic Douloureux") OR TIAB("transient brain ischaemia") OR TIAB("transient brain ischemia") OR TIAB("Transient Brain Stem Ischemia") OR TIAB("Transient Brain Stem Ischemias") OR TIAB("Transient Brainstem Ischemia") OR TIAB("Transient Brainstem Ischemias") OR TIAB("Transient Cerebral Ischemia") OR TIAB("Transient Cerebral Ischemias") OR TIAB("Transient Cerebral Ischaemia") OR TIAB("Transient Cerebral Ischaemias") OR TIAB("transient ischaemic attacks") OR TIAB("transient ischaemic seizures") OR TIAB("Transient Ischemic Attacks") OR TIAB("transient ischemic seizures") OR TIAB("Trifacial Neuralgia") OR TIAB("Trifacial Neuralgias") OR TIAB("Trigeminal Neuralgia") OR TIAB("Trigeminal Neuralgias") OR TIAB("Vascular Dementia") OR TIAB("Vascular Dementias") OR TIAB("acute paranasal sinusitis") OR TIAB("airway disease") OR TIAB("airway diseases") OR TIAB("airway disorder") OR TIAB("airway disorders") OR TIAB("airway dysfunctioning") OR TIAB("airway dysfunction") OR TIAB("airway dysfunctions") OR TIAB("Asthma") OR TIAB("Asthmas") OR TIAB("asthmatic") OR TIAB("Atrophic Rhinitides") OR TIAB("Atrophic Rhinitis") OR TIAB("Auditory Vertigo") OR TIAB("Auditory Vertigos") OR TIAB("Aural Vertigo") OR TIAB("benign paroxysmal postural vertigo") OR TIAB("benign postural paroxysmal vertigo") OR TIAB("bronchitis chronica") OR TIAB("cerebral vertigo") OR TIAB("Chronic Airflow Obstruction") OR TIAB("Chronic Airflow Obstructions") OR TIAB("chronic airway obstruction") OR TIAB("Chronic Bronchitis") OR TIAB("chronic bronchus infection") OR TIAB("chronic emphysema") OR TIAB("chronic obstructive bronchopulmonary disease") OR TIAB("Chronic Obstructive Lung Disease") OR TIAB("chronic obstructive lung disorder") OR TIAB("Chronic Obstructive Pulmonary Disease") OR TIAB("Chronic Obstructive Pulmonary Diseases") OR TIAB("chronic obstructive pulmonary disorder") OR TIAB("chronic pulmonary obstructive disease") OR TIAB("chronic pulmonary obstructive disorder") OR TIAB("COAD") OR TIAB("cochlea hydrops") OR TIAB("COPD") OR TIAB("Cystic Fibrosis of Pancreas") OR TIAB("diffuse parenchyma lung disease") OR TIAB("Diffuse Parenchymal Lung Disease") OR TIAB("Diffuse Parenchymal Lung Diseases") OR TIAB("diffuse parenchymal pulmonary disease") OR TIAB("diffuse parenchymal pulmonary disorder") OR TIAB("endolymphatic hydrops") OR TIAB("endolymphatic sac hydrops") OR TIAB("Ethmoid Sinusitides") OR TIAB("Ethmoid Sinusitis") OR TIAB("Ethmoidal Sinusitides") OR TIAB("Ethmoidal Sinusitis") OR TIAB("fibrocystic disease") OR TIAB("hydrops labyrinthi") OR TIAB("Hypersomnia with Periodic Respiration") OR TIAB("Interstitial Lung Disease") OR TIAB("Interstitial Lung Diseases") OR TIAB("interstitial lung disorder") OR TIAB("Interstitial Pneumonia") OR TIAB("Interstitial Pneumonias") OR TIAB("Interstitial Pneumonitides") OR TIAB("Interstitial Pneumonitis") OR TIAB("interstitial pneumopathy") OR TIAB("interstitial pulmonary disease") OR TIAB("interstitial pulmonary disorder") OR TIAB("labyrinth hydrops") OR TIAB("labyrinthal syndrome") OR TIAB("lung allergy") OR TIAB("lung chronic obstructive disease") OR TIAB("Meniere Disease") OR TIAB("Ménière Disease") OR TIAB("Ménière Diseases") OR TIAB("Meniere Syndrome") OR TIAB("Ménière Vertigo") OR TIAB("Menieres Disease") OR TIAB("Meniere's Disease") OR TIAB("Ménières Disease") OR TIAB("Ménière's Disease") OR TIAB("Ménière's Diseases") OR TIAB("Menieres Syndrome") OR TIAB("Meniere's Syndrome") OR TIAB("Ménières Vertigo") OR TIAB("Ménière's

Vertigo") OR TIAB("Ménière's Vertigos") OR TIAB("Mucoviscidosis") OR TIAB("mucoviscoidosis") OR TIAB("nasal sinusitis") OR TIAB("nocturnal apnea") OR TIAB("nocturnal apnoea") OR TIAB("obstructive chronic lung disease") OR TIAB("obstructive chronic pulmonary disease") OR TIAB("Otogenic Vertigo") OR TIAB("Otogenic Vertigos") OR TIAB("Otoscleroses") OR TIAB("otosclerosis surgery") OR TIAB("otosclerotic stapes") OR TIAB("otosphongiosis") OR TIAB("Otospongioses") OR TIAB("Otospongiosis") OR TIAB("Ozena") OR TIAB("Ozenas") OR TIAB("pancreas cystic disease") OR TIAB("pancreas cystic fibrosis") OR TIAB("Pancreas Fibrocystic Diseases") OR TIAB("pancreas fibrosis") OR TIAB("pancreatic cystic disease") OR TIAB("Pancreatic Cystic Fibrosis") OR TIAB("pancreatic fibrosis") OR TIAB("paroxysmal labyrinthine vertigo") OR TIAB("paroxysmal positional vertigo") OR TIAB("pneumatosis") OR TIAB("positional paroxysmal vertigo") OR TIAB("Pulmonary Cystic Fibrosis") OR TIAB("respiration disease") OR TIAB("respiration diseases") OR TIAB("respiration disorder") OR TIAB("respiration disorders") OR TIAB("respiration dysfunctioning") OR TIAB("respiration dysfunction") OR TIAB("respiration dysfunctions") OR TIAB("respiration tract disease") OR TIAB("respiration tract diseases") OR TIAB("respiration tract disorder") OR TIAB("respiration tract disorders") OR TIAB("respiration tract dysfunctioning") OR TIAB("respiration tract dysfunction") OR TIAB("respiration tract dysfunctions") OR TIAB("respiratory disease") OR TIAB("respiratory diseases") OR TIAB("respiratory disorder") OR TIAB("respiratory disorders") OR TIAB("respiratory illness") OR TIAB("respiratory illnesses") OR TIAB("respiratory tract disease") OR TIAB("respiratory tract diseases") OR TIAB("respiratory tract disorder") OR TIAB("respiratory tract disorders") OR TIAB("respiratory tract dysfunction") OR TIAB("respiratory tract dysfunctioning") OR TIAB("respiratory tract dysfunctions") OR TIAB("rhinitis atrophica") OR TIAB("sinusitis nasalis") OR TIAB("Sleep Apnea") OR TIAB("Sleep Apneas") OR TIAB("sleep apnoea") OR TIAB("Sleep Disordered Breathing") OR TIAB("Sleep Hypopnea") OR TIAB("Sleep Hypopneas") OR TIAB("Sleep-Disordered Breathing") OR TIAB("vertiginous disease") OR TIAB("vertiginous disorder") OR TIAB("vertiginous syndrome") OR TIAB("vestibular vertigo") OR TIAB("Age Related Osteoporosis") OR TIAB("Age-Related Bone Loss") OR TIAB("Age-Related Bone Losses") OR TIAB("Age-Related Osteoporosis") OR TIAB("Akureyri disease") OR TIAB("Algodystrophic Syndrome") OR TIAB("Algodystrophies") OR TIAB("Algodystrophy") OR TIAB("alibert bazin disease") OR TIAB("Arthritic Psoriasis") OR TIAB("arthritis") OR TIAB("arthropathic psoriasis") OR TIAB("Arthroses") OR TIAB("Arthrosis") OR TIAB("axial spondylarthritis") OR TIAB("Axial Spondyloarthritides") OR TIAB("Axial Spondyloarthritis") OR TIAB("AxSpA") OR TIAB("Back Ache") OR TIAB("Back Aches") OR TIAB("Back Pain") OR TIAB("Back Pains") OR TIAB("Backache") OR TIAB("Backaches") OR TIAB("backpain") OR TIAB("beauvais disease") OR TIAB("Bilateral Sciatica") OR TIAB("Bilateral Sciaticas") OR TIAB("Bone Paget Disease") OR TIAB("Bone Pagets Disease") OR TIAB("Calcium Pyrophosphate Deposition Disease") OR TIAB("Calcium Pyrophosphate Dihydrate Deposition") OR TIAB("Cervical Pain") OR TIAB("Cervical Pains") OR TIAB("Cervical Sympathetic Dystrophies") OR TIAB("Cervical Sympathetic Dystrophy") OR TIAB("Cervicalgia") OR TIAB("Cervicalgias") OR TIAB("Cervicodynia") OR TIAB("Cervicodynias") OR TIAB("chariot disease") OR TIAB("Chondrocalcinoses") OR TIAB("chronic articular rheumatism") OR TIAB("chronic fatigue") OR TIAB("Chronic Fatigue-Fibromyalgia Syndromes") OR TIAB("Chronic Infectious Mononucleosis Like Syndrome") OR TIAB("Chronic Infectious Mononucleosis-Like Syndrome") OR TIAB("chronic rheumatism") OR TIAB("complex regional pain syndrome 1") OR TIAB("complex regional pain syndrome type 1") OR TIAB("CRPS 1") OR TIAB("CRPS I") OR TIAB("CRPS type 1") OR TIAB("CRPS Type I") OR TIAB("CRPS-I") OR TIAB("crystal arthropathies") OR TIAB("crystalline arthropathy") OR TIAB("degenerative joint disease") OR TIAB("Diffuse Myofascial Pain Syndrome") OR TIAB("disc hernia") OR TIAB("Disc Herniation") OR TIAB("Disc Herniations") OR TIAB("disc prolapse") OR TIAB("Disc Protrusion") OR TIAB("Disc Protrusions") OR TIAB("discal hernia") OR TIAB("discal herniation") OR TIAB("discus hernia") OR TIAB("disk hernia") OR TIAB("Disk Herniation") OR TIAB(

"Disk Herniations") OR TIAB("Disk Prolapse") OR TIAB("Disk Prolapses") OR TIAB("Disk Protrusion") OR TIAB("Disk Protrusions") OR TIAB("dorsalgia") OR TIAB("epicondylalgia") OR TIAB("epidemic neuromyasthenia") OR TIAB("fatigue syndrome") OR TIAB("chronic Fatigue-Fibromyalgia Syndrome") OR TIAB("fibro myalgia") OR TIAB("Fibromyalgia Fibromyositis Syndrome") OR TIAB("Fibromyalgia-Fibromyositis Syndrome") OR TIAB("Fibromyalgia-Fibromyositis Syndromes") OR TIAB("Fibromyalgias") OR TIAB("Fibromyositis Fibromyalgia Syndrome") OR TIAB("Fibromyositis-Fibromyalgia Syndrome") OR TIAB("Fibromyositis-Fibromyalgia Syndromes") OR TIAB("fibrositic nodule") OR TIAB("Fibrositides") OR TIAB("Fibrositis") OR TIAB("hernia disci") OR TIAB("hernia nuclei pulposi") OR TIAB("Herniated Disc") OR TIAB("Herniated Discs") OR TIAB("Herniated Disk") OR TIAB("Herniated Disks") OR TIAB("herniated intervertebral disc") OR TIAB("herniated intervertebral disk") OR TIAB("herniated nucleus pulposus") OR TIAB("herniated vertebral disc") OR TIAB("herniated vertebral disk") OR TIAB("hypertrophic infiltrative tendinitis") OR TIAB("Iceland disease") OR TIAB("Intervertebral Disc Displacement") OR TIAB("Intervertebral Disc Displacements") OR TIAB("Intervertebral Disk Displacement") OR TIAB("Intervertebral Disk Displacements") OR TIAB("intervertebral disk perforation") OR TIAB("intervertebral disk rupture") OR TIAB("intervertebral prolapse") OR TIAB("Involutional Osteoporosis") OR TIAB("ischias") OR TIAB("ischiatic pain") OR TIAB("Lateral Epicondylitides") OR TIAB("Lateral Epicondylitis") OR TIAB("Lateral Humeral Epicondylitides") OR TIAB("Lateral Humeral Epicondylitis") OR TIAB("loin pain") OR TIAB("lowback pain") OR TIAB("Lumbago") OR TIAB("lumbal pain") OR TIAB("lumbal syndrome") OR TIAB("lumbalgesia") OR TIAB("lumbalgia") OR TIAB("lumbar pain") OR TIAB("lumbar spine syndrome") OR TIAB("lumbodynia") OR TIAB("lumbosacral pain") OR TIAB("lumbosacral root syndrome") OR TIAB("lumbosacroiliac strain") OR TIAB("Muscular Rheumatism") OR TIAB("Myalgic Encephalomyelitis") OR TIAB("Neck Ache") OR TIAB("Neck Aches") OR TIAB("Neck Pain") OR TIAB("Neck Pains") OR TIAB("Neckache") OR TIAB("Neckaches") OR TIAB("neuralgic shoulder amyotrophy") OR TIAB("nodular tendinitis") OR TIAB("nucleus pulposus hernia") OR TIAB("Osseous Paget's Disease") OR TIAB("osteitis deformans") OR TIAB("Osteoarthritides") OR TIAB("osteo-arthritis") OR TIAB("Osteoarthroses") OR TIAB("Osteoarthrosis") OR TIAB("osteo-arthrosis") OR TIAB("Osteoporoses") OR TIAB("ostitis deformans") OR TIAB("Paget Disease of Bone") OR TIAB("Paget disease of the bone") OR TIAB("Pagets bone disease") OR TIAB("Paget's bone disease") OR TIAB("Pagets disease of bone") OR TIAB("Paget's Disease of Bone") OR TIAB("Paget's disease of the bone") OR TIAB("paralytic scoliosis") OR TIAB("Post Traumatic Osteoporosis") OR TIAB("posttraumatic dystrophy") OR TIAB("post-traumatic dystrophy") OR TIAB("Post-Traumatic Osteoporosis") OR TIAB("Postviral Fatigue Syndromes") OR TIAB("Primary Fibromyalgia") OR TIAB("primary osteoarthritis") OR TIAB("progressive scoliosis") OR TIAB("Prolapsed Disc") OR TIAB("Prolapsed Discs") OR TIAB("Prolapsed Disk") OR TIAB("Prolapsed Disks") OR TIAB("Protruded Disc") OR TIAB("Protruded Discs") OR TIAB("Protruded Disk") OR TIAB("Protruded Disks") OR TIAB("Pseudogout") OR TIAB("Psoriasis Arthropathica") OR TIAB("psoriasis pustulosa arthropathica") OR TIAB("Psoriatic Arthropathies") OR TIAB("Psoriatic Arthropathy") OR TIAB("psoriatic rheumatism") OR TIAB("Reflex Sympathetic Dystrophies") OR TIAB("Reflex Sympathetic Dystrophy") OR TIAB("rheumarthritis") OR TIAB("rheumatic disease") OR TIAB("rheumatic diseases") OR TIAB("rheumatoid disease") OR TIAB("rheumatoid diseases") OR TIAB("rheumatoid inflammation") OR TIAB("rheumatological disease") OR TIAB("rheumatological diseases") OR TIAB("rheumatological disorder") OR TIAB("rheumatological disorders") OR TIAB("Royal Free Disease") OR TIAB("Sciatic Neuralgia") OR TIAB("Sciatic Neuralgias") OR TIAB("sciatic pain") OR TIAB("Scolioses") OR TIAB("Secondary Fibromyalgia") OR TIAB("Senile Osteoporosis") OR TIAB("shoulder arm syndrome") OR TIAB("Shoulder Hand Syndrome") OR TIAB("Shoulder-Hand Syndrome") OR TIAB("Shoulder-Hand Syndromes") OR TIAB("Slipped Disc") OR TIAB("Slipped Discs") OR TIAB("Slipped Disk") OR TIAB(

"Slipped Disks") OR TIAB("slipped intervertebral disc") OR TIAB("slipped vertebral disc") OR TIAB("spinal disk disease") OR TIAB("Sudek Atrophy") OR TIAB("Sudek's Atrophies") OR TIAB("Sudeks Atrophy") OR TIAB("Sudek's Atrophy") OR TIAB("sympathetic dystrophy syndrome") OR TIAB("Sympathetic Reflex Dystrophia") OR TIAB("Sympathetic Reflex Dystrophias") OR TIAB("sympathetic reflex dystrophy") OR TIAB("Systemic Exertion Intolerance Disease") OR TIAB("tendinopathy") OR TIAB("tendinosis") OR TIAB("tendonitis") OR TIAB("tendonopathy") OR TIAB("Tennis Elbow") OR TIAB("Tennis Elbows") OR TIAB("tenonitis") OR TIAB("tenontitis") OR TIAB("tenositis") OR TIAB("Type I Complex Regional Pain Syndrome") OR TIAB("Vertebrogenic Pain Syndrome") OR TIAB("Vertebrogenic Pain Syndromes") OR TIAB("Yuppie flu") OR TIAB("Acne Inversa") OR TIAB("Acne Inversas") OR TIAB("acne juvenilis") OR TIAB("Acne Rosacea") OR TIAB("Chronic Bullous Disease of Childhood") OR TIAB("cutaneous disease") OR TIAB("cutaneous diseases") OR TIAB("cutaneus disorder") OR TIAB("cutaneus disorders") OR TIAB("dermal disease") OR TIAB("dermal diseases") OR TIAB("dermal disorder") OR TIAB("dermal disorders") OR TIAB("Drug induced Linear IgA Bullous Dermatosis") OR TIAB("Drug induced Linear IgA Bullous Dermatoses") OR TIAB("Drug-induced Linear IgA Bullous Dermatosis") OR TIAB("Drug-induced Linear IgA Bullous Dermatoses") OR TIAB("Eczema") OR TIAB("Eczemas") OR TIAB("eczematoid syndrome") OR TIAB("Eczematous Dermatitides") OR TIAB("eczematous dermatitis") OR TIAB("eczematous eruption") OR TIAB("eczematous skin") OR TIAB("Erythematotelangiectatic Rosacea") OR TIAB("Granulomatous Rosacea") OR TIAB("herpes") OR TIAB("hidradenitis suppurativa") OR TIAB("Hives") OR TIAB("juvenile acne") OR TIAB("Linear IgA Dermatosis") OR TIAB("Linear IgA Dermatoses") OR TIAB("Linear IgA IgG Bullous Dermatosis") OR TIAB("Linear IgA IgG Bullous Dermatoses") OR TIAB("Linear IgA IgG Dermatosis") OR TIAB("Linear IgA IgG Dermatoses") OR TIAB("Ocular Rosacea") OR TIAB("Palmoplantaris Pustulosis") OR TIAB("Papulopustular Rosacea") OR TIAB("Phymatous Rosacea") OR TIAB("Psoriases") OR TIAB("psoriasiform dermatitis") OR TIAB("psoriasiform dermatosis") OR TIAB("psoriasiform lesion") OR TIAB("psoriasiform rash") OR TIAB("psoriasiform skin rash") OR TIAB("psoriatic epidermis") OR TIAB("psoriatic skin") OR TIAB("Pustular Psoriasis of Palms and Soles") OR TIAB("Pustulosis of Palms and Soles") OR TIAB("Pustulosis Palmaris et Plantaris") OR TIAB("rhinophyma") OR TIAB("rozacea") OR TIAB("skin and connective tissue disease") OR TIAB("skin and connective tissue diseases") OR TIAB("skin and connective tissue disorder") OR TIAB("skin and connective tissue disorders") OR TIAB("skin disease") OR TIAB("skin diseases") OR TIAB("skin disorder") OR TIAB("skin disorders") OR TIAB("Suppurative Hidradenitis") OR TIAB("Suppurative Hidradenitides") OR TIAB("urticary") OR TIAB("weal") OR TIAB("wheal") OR TIAB("whealing") OR TIAB("willan lepra") OR TIAB("active TB") OR TIAB("Adamantiades-Behcet Disease") OR TIAB("Adamantiades-Behcet Diseases") OR TIAB("Allergic Angiitis") OR TIAB("Allergic Angiitides") OR TIAB("Allergic Granulomatoses") OR TIAB("allergic granulomatosis") OR TIAB("Allergic Granulomatous and Angiitis") OR TIAB("Allergic Granulomatous Angiitides") OR TIAB("allergic granulomatous angiitis") OR TIAB("allergic granulomatous angitis") OR TIAB("anonymous artery occlusion") OR TIAB("Anti Phospholipid Antibody Syndrome") OR TIAB("Anti Phospholipid Antibody Syndromes") OR TIAB("Anti Phospholipid Syndrome") OR TIAB("Anti Phospholipid Syndromes") OR TIAB("Antiphospholipid Antibody Syndrome") OR TIAB("Antiphospholipid Antibody Syndromes") OR TIAB("Anti-Phospholipid Antibody Syndrome") OR TIAB("Anti-Phospholipid Antibody Syndromes") OR TIAB("antiphospholipid syndrome") OR TIAB("Anti-Phospholipid Syndrome") OR TIAB("Anti-Phospholipid Syndromes") OR TIAB("aorta arch syndrome") OR TIAB("aortic arch syndromes") OR TIAB("Aortitis Syndrome") OR TIAB("Aortitis Syndromes") OR TIAB("APLA syndrome") OR TIAB("arteritis brachiocephalica") OR TIAB("arteritis nodosa") OR TIAB("autoimmune disease") OR TIAB("autoimmune diseases") OR TIAB("Behcet Disease") OR TIAB("Behcet Diseases") OR TIAB("Behçet Disease") OR TIAB("Behçet Diseases") OR TIAB("Behcet syndrome") OR TIAB("behcet ulcer") OR

TIAB("Behcets disease") OR TIAB("Behcet's Disease") OR TIAB("Behcet's Diseases") OR TIAB("Behcets syndrome") OR TIAB("Behcet's Syndrome") OR TIAB("Behcet's Syndromes") OR TIAB("besnier boeck syndrome") OR TIAB("Besnier-Boeck Disease") OR TIAB("Besnier-Boeck Diseases") OR TIAB("Besnier-Boeck-Schaumann Syndrome") OR TIAB("Besnier-Boeck-Schaumann Syndromes") OR TIAB("Boeck Disease") OR TIAB("Boeck Diseases") OR TIAB("Boecks Disease") OR TIAB("Boecks Diseases") OR TIAB("Boeck's Disease") OR TIAB("Boeck's Diseases") OR TIAB("brachiocephalic arteritis") OR TIAB("brachiocephalic artery occlusion") OR TIAB("brachiocephalic ischaemia") OR TIAB("brachiocephalic ischemia") OR TIAB("brachiocephalic trunk occlusion") OR TIAB("brachiocephalic vascular occlusion") OR TIAB("Church Strauss syndrome") OR TIAB("Churg Strauss") OR TIAB("Churg-Strauss Syndrome") OR TIAB("Cranial Arteritis") OR TIAB("Cranial Arteritides") OR TIAB("cryoglobulinaemia") OR TIAB("Cryoglobulinemias") OR TIAB("cryoimmunoglobulinaemia") OR TIAB("cryoimmunoglobulinemia") OR TIAB("Cutis Elastica") OR TIAB("dacryosialoadenopathia atrophicans") OR TIAB("EDS IV") OR TIAB("Ehlers Danlos") OR TIAB("Ehlers-Danlos Disease") OR TIAB("Ehlers-Danlos Diseases") OR TIAB("Ehlers-Danlos syndrome") OR TIAB("eosinophilic GPA") OR TIAB("eosinophilic granulomatosis polyangiitis") OR TIAB("eosinophilic granulomatosis polyangitis") OR TIAB("eosinophilic granulomatous angiitis") OR TIAB("Eosinophilic Granulomatous Vasculitides") OR TIAB("erythematodes visceralis") OR TIAB("Essential Polyarteritis") OR TIAB("Essential Polyarteritides") OR TIAB("generalised scleroderma") OR TIAB("generalized scleroderma") OR TIAB("Giant Cell Aortic Arteritis") OR TIAB("Giant Cell Aortitis") OR TIAB("Giant Cell Aortitides") OR TIAB("giant cell arteriitis") OR TIAB("Giant Cell Arteritides") OR TIAB("giant cell arteritis") OR TIAB("gougerot houwer sjoegren syndrome") OR TIAB("gougerot mulock houwer sjoegren syndrome") OR TIAB("Gougerot Sjoegren syndrome") OR TIAB("Gougerot-Sjogren syndrome") OR TIAB("granulomatosis and polyangiitis") OR TIAB("granulomatosis and polyangitis") OR TIAB("Granulomatosis with Polyangiitides") OR TIAB("granulomatosis with polyangitis") OR TIAB("granulomatous allergic angitis") OR TIAB("granulomatous polyangiitis") OR TIAB("granulomatous polyangitis") OR TIAB("Horton arteritis") OR TIAB("Horton Disease") OR TIAB("Horton's arteritis") OR TIAB("Hortons Disease") OR TIAB("Horton's Disease") OR TIAB("Hughes Syndrome") OR TIAB("Hughes Syndromes") OR TIAB("innominate arterial ligation") OR TIAB("innominate artery ligation") OR TIAB("innominate artery occlusion") OR TIAB("jungling syndrome") OR TIAB("kussmaul maier disease") OR TIAB("kussmaul syndrome") OR TIAB("Libman Sacks Disease") OR TIAB("Libman Sacks Diseases") OR TIAB("Libman-Sacks Disease") OR TIAB("Libman-Sacks Diseases") OR TIAB("lupovisceritis") OR TIAB("lymphogranuloma benignum") OR TIAB("malignant dermatovisceritism") OR TIAB("martorell syndrome") OR TIAB("Microscopic Polyangiitides") OR TIAB("microscopic polyarteritis") OR TIAB("mikulicz radecki syndrome") OR TIAB("mixed cryoglobulinemia") OR TIAB("morbus Wegener") OR TIAB("mucoserous dyssecretosis") OR TIAB("mukilicz radecki syndrome") OR TIAB("multisystem disease") OR TIAB("multisystem diseases") OR TIAB("multisystem disorder") OR TIAB("multisystem disorders") OR TIAB("multisystem dysfunctioning") OR TIAB("multisystem dysfunction") OR TIAB("multisystem dysfunctions") OR TIAB("necrotising respiratory granulomatosis") OR TIAB("Necrotizing Arteritis") OR TIAB("Necrotizing Arteritides") OR TIAB("necrotizing respiratory granulomatosis") OR TIAB("nodular periarteritis") OR TIAB("nodular polyarteritis") OR TIAB("oculobuccopharyngeal dryness") OR TIAB("Old Silk Route Disease") OR TIAB("Old Silk Route Diseases") OR TIAB("panarteriitis nodosa") OR TIAB("panarteritis nodosa") OR TIAB("periarterial fibrosis") OR TIAB("periarteriitis nodosa") OR TIAB("Periarteritis Nodosa") OR TIAB("pneumogenic granulomatosis") OR TIAB("poliarteritis nodosa") OR TIAB("polyarteriitis nodosa") OR TIAB("progressive scleroderma") OR TIAB("progressive sclerodermia") OR TIAB("Pulseless Disease") OR TIAB("Pulseless Diseases") OR TIAB("reversed coarctation") OR TIAB("rheumatic sialosis") OR TIAB("sarcoid") OR TIAB("sarcoidoses") OR TIAB("Schaumann Disease")

OR TIAB("Schaumann Diseases") OR TIAB("Schaumann Syndrome") OR TIAB("Schaumann Syndromes") OR TIAB("Schaumann's Syndrome") OR TIAB("Schaumann's Syndromes") OR TIAB("sicca syndrome") OR TIAB("sjoegren disease") OR TIAB("sjogren disease") OR TIAB("sjogren syndrome") OR TIAB("sjogren's syndrome") OR TIAB("lupus") OR TIAB("Strauss Churg syndrome") OR TIAB("systemic disease") OR TIAB("systemic diseases") OR TIAB("systemic disorder") OR TIAB("systemic disorders") OR TIAB("systemic dysfunctioning") OR TIAB("systemic dysfunction") OR TIAB("systemic dysfunctions") OR TIAB("systemic progressive sclerosis") OR TIAB("Systemic Scleroderma") OR TIAB("Systemic Sclerosis") OR TIAB("takayasu arteriopathy") OR TIAB("Takayasu Arteritis") OR TIAB("Takayasu Disease") OR TIAB("Takayasu Diseases") OR TIAB("takayasu ohnishi syndrome") OR TIAB("Takayasu Syndrome") OR TIAB("Takayasu Syndromes") OR TIAB("Takayasus Arteritis") OR TIAB("Takayasu's Arteritis") OR TIAB("Temporal Arteritis") OR TIAB("Temporal Arteritides") OR TIAB("Triple Symptom Complex") OR TIAB("Triple Symptom Complices") OR TIAB("Triple-Symptom Complex") OR TIAB("Wegener disease") OR TIAB("Wegener granuloma") OR TIAB("Wegener Granulomatosis") OR TIAB("Wegener Klinger Churg syndrome") OR TIAB("Wegener Klinger granulomatosis") OR TIAB("Wegener syndrome") OR TIAB("Wegener's disease") OR TIAB("Wegener's GPA") OR TIAB("Wegener's granuloma") OR TIAB("Wegener's Granulomatosis") OR TIAB("Wegner granulomatosis") OR TIAB("Young Female Arteritis") OR TIAB("Young Female Arteritides") OR TIAB("Anti GBM Disease*") OR TIAB("Anti Glomerular Basement Membrane Disease") OR TIAB("Anti-GBM Disease*") OR TIAB("bladder incontinence") OR TIAB("chronic disease kidney function") OR TIAB("Chronic Kidney Failure*") OR TIAB("chronic nephropathy") OR TIAB("Chronic Renal Failure*") OR TIAB("cystic kidney") OR TIAB("End Stage Kidney Disease*") OR TIAB("End Stage Renal Disease*") OR TIAB("End Stage Renal Failure*") OR TIAB("End-Stage Kidney Disease*") OR TIAB("End-Stage Renal Disease*") OR TIAB("End-Stage Renal Failure*") OR TIAB("ESRD") OR TIAB("familial nephrolithiasis") OR TIAB("goodpasture disease") OR TIAB("goodpasture renopulmonary syndrome") OR TIAB("Goodpasture Syndrome*") OR TIAB("Goodpastures Syndrome*") OR TIAB("Goodpasture's Syndrome*") OR TIAB("incontinentia urinae") OR TIAB("involuntary urinary loss") OR TIAB("involuntary urination") OR TIAB("involuntary urine loss") OR TIAB("Kidney Calcul*") OR TIAB("kidney calix stone") OR TIAB("kidney calyx stone") OR TIAB("kidney chronic failure") OR TIAB("kidney cystic disease") OR TIAB("kidney disease") OR TIAB("kidney diseases") OR TIAB("kidney disorder") OR TIAB("kidney disorders") OR TIAB("kidney failure") OR TIAB("kidney failures") OR TIAB("kidney insufficien*") OR TIAB("kidney lithiasis") OR TIAB("kidney multicystic aplasia") OR TIAB("kidney multicystic disease") OR TIAB("kidney pelvis stone") OR TIAB("kidney polycystosis") OR TIAB("Kidney Stone*") OR TIAB("leakage of urine") OR TIAB("Lung Purpura with Nephritis") OR TIAB("male genital disorder") OR TIAB("male genital disorders") OR TIAB("male infertility") OR TIAB("Nephrolith") OR TIAB("pneumorenal syndrome") OR TIAB("Polycystic Kidney") OR TIAB("Polycystic Kidneys") OR TIAB("prostate adenoma") OR TIAB("Renal Calcul*") OR TIAB("renal cystic disease") OR TIAB("renal disease") OR TIAB("renal diseases") OR TIAB("renal disorder") OR TIAB("renal disorders") OR TIAB("renal failure") OR TIAB("renal failures") OR TIAB("renal insufficien*") OR TIAB("renal pelvis stone") OR TIAB("renal polycystic disease") OR TIAB("renal stone") OR TIAB("renolithiasis") OR TIAB("unwanted urine loss") OR TIAB("urinary incontinence") OR TIAB("urinary leakage") OR TIAB("urine incontinence") OR TIAB("urine leakage") OR TIAB("urine wetting") OR TIAB("urologic disease*") OR TIAB("urologic disorder*") OR TIAB("urological disease*") OR TIAB("urological disorder*") OR TIAB("Age Related Macular Degeneration") OR TIAB("Age-Related Macular Degeneration*") OR TIAB("atrophia maculae luteae") OR TIAB("bilateral macular degeneration") OR TIAB("Cataract*") OR TIAB("chronic uveitis") OR TIAB("corpus vitreum detachment") OR TIAB("Day Blindness") OR TIAB("degeneratio maculae luteae retinae") OR TIAB("detachment corporis vitrei") OR TIAB("detachment vitreous") OR TIAB("disciform macular degeneration") OR TIAB(

"Episclerit*") OR TIAB("eye disease") OR TIAB("eye diseases") OR TIAB("eye disorder") OR TIAB("eye disorders") OR TIAB("eye dysfunction") OR TIAB("eye dysfunctioning") OR TIAB("eye dysfunctions") OR TIAB("Hemeralopia*") OR TIAB("heredomacular degeneration") OR TIAB("immunogenic uveitis") OR TIAB("junius kuhnt disease") OR TIAB("lens clouding") OR TIAB("Lens Opacit*") OR TIAB("Macropsia*") OR TIAB("macula atrophy") OR TIAB("macula bilateral degeneration") OR TIAB("macula degeneration") OR TIAB("macula lutea atrophy") OR TIAB("macula lutea degeneration") OR TIAB("macula lutea disciform degeneration") OR TIAB("macula lutea retina atrophy") OR TIAB("macula lutea retina degeneration") OR TIAB("macula retina atrophy") OR TIAB("macula retina degeneration") OR TIAB("macular atrophy") OR TIAB("Macular Degenerations") OR TIAB("macular disciform degeneration") OR TIAB("Macular Dystroph*") OR TIAB("Maculopath*") OR TIAB("Metamorphopsia*") OR TIAB("Micropsia*") OR TIAB("Myopia*") OR TIAB("Nearsightedness*") OR TIAB("Necrotizing Sclerit*") OR TIAB("ocular disease") OR TIAB("ocular diseases") OR TIAB("ocular disorder") OR TIAB("ocular disorders") OR TIAB("ocular dysfunction*") OR TIAB("ophthalmic disease") OR TIAB("ophthalmic diseases") OR TIAB("ophthalmic disorder") OR TIAB("ophthalmic disorders") OR TIAB("ophthalmic dysfunction*") OR TIAB("ophthalmologic disease") OR TIAB("ophthalmologic diseases") OR TIAB("ophthalmologic disorder") OR TIAB("ophthalmologic disorders") OR TIAB("ophthalmologic dysfunction*") OR TIAB("panuveitis") OR TIAB("posterior capsule opacification") OR TIAB("posterior uveitis") OR TIAB("Presbyopias") OR TIAB("Pseudoaphakia*") OR TIAB("retina macula disciform degeneration") OR TIAB("retinal diseases") OR TIAB("Scleritides") OR TIAB("secondary scleritis") OR TIAB("suppurative uveitis") OR TIAB("Uveitides") OR TIAB("Vision Disabilit*") OR TIAB("vision disorder") OR TIAB("vision disorders") OR TIAB("vision disturbance") OR TIAB("visual disorder") OR TIAB("visual disorders") OR TIAB("visual disturbance") OR TIAB("Visual Impairment*") OR TIAB("vitreous detachment") OR TIAB("sarcoidosis") OR TIAB("neoplasia") OR TIAB("lymphoma*") OR TIAB("Tuberculosis") OR TIAB("myelodysplastic syndrome") OR TIAB("hypertension") OR TIAB("infarction*") OR TIAB("glaucom*") OR TIAB("cancer*") OR TIAB("carcinoma*") OR TIAB("neoplasm*") OR TIAB("tumor*") OR TIAB("tumour*") OR TIAB("Horton syndrome") OR TIAB("Horton's syndrome") OR TIAB("granulomatosis with polyangiitis") OR TIAB("Gastro-esophageal reflux") OR TIAB("gastroesophageal reflux") OR TIAB("GORD")) AND (SU.EXPLODE("desire") OR SU.EXPLODE("sexual satisfaction") OR SU.EXPLODE("sexual intercourse (human)") OR SU.EXPLODE("sexual health") OR SU.EXPLODE("genital disorders") OR SU.EXPLODE("sexual function disturbances") OR SU.EXPLODE("dyspareunia") OR SU.EXPLODE("female sexual dysfunction") OR SU.EXPLODE("vaginismus") OR SU.EXPLODE("libido") OR SU.EXPLODE("orgasm") OR SU.EXPLODE("male orgasm") OR SU.EXPLODE("erectile dysfunction") OR SU.EXPLODE("inhibited sexual desire") OR SU.EXPLODE("sexual arousal") OR SU.EXPLODE("premature ejaculation") OR SU.EXPLODE("female orgasm") OR SU.EXPLODE("erection (penis)") OR SU.EXPLODE("psychosexual behavior") OR MESH("Sexual dysfunction, physiological") OR MESH("Sexual dysfunction, psychological") OR MESH("Sexuality") OR MESH("Sexual arousal") OR MESH("Erectile Dysfunction") OR MESH("Libido ") OR MESH("Orgasm") OR MESH("Vaginismus") OR MESH("Dyspareunia ") OR TIAB("coitus") OR TIAB("desire") OR TIAB("dyspareunia") OR TIAB("ego-dystonic homosexuality") OR TIAB("ejaculatio praecox") OR TIAB("ejaculatio praecoxs") OR TIAB("ejaculatio precox") OR TIAB("erectile dysfunction*") OR TIAB("erection") OR TIAB("frigidities") OR TIAB("frigidity") OR TIAB("genital disorder") OR TIAB("genital disorders") OR TIAB("go-dystonic homosexuality") OR TIAB("libido") OR TIAB("orgasm") OR TIAB("orgasms") OR TIAB("premature ejaculation") OR TIAB("premature ejaculations") OR TIAB("psychosexuality") OR TIAB("sex abnormalit*") OR TIAB("sex arousal") OR TIAB("sex disorder") OR TIAB("sex disorders") OR TIAB("sex drive") OR TIAB("sex dysfunction*") OR TIAB("sex insufficienc*") OR TIAB("sex problem") OR TIAB("sex problems") OR TIAB("vaginismus") OR TIAB("sexual") OR TIAB("psychosexual") OR TIAB("sexuality")) AND

(SU.EXPLODE("Emerging Adulthood") OR SU.EXPLODE("Middle Adulthood") OR SU.EXPLODE("Older Adulthood") OR MESH("adult") OR TIAB("adult") OR TIAB("adults") OR TIAB("old people") OR TIAB("elderly") OR TIAB("frail") OR TIAB("frailness") OR TIAB("frailty") OR TIAB("old age") OR TIAB("old patients") OR TIAB("old person") OR TIAB("old persons") OR TIAB("older adult") OR TIAB("older adults") OR TIAB("older patient") OR TIAB("older patients") OR TIAB("older people") OR TIAB("older person") OR TIAB("older persons") OR TIAB("senior people") OR TIAB("senior person") OR TIAB("senior persons") OR TIAB("seniors")) AND (SU.EXPLODE("meta analysis") OR SU.EXPLODE("Systematic Review") OR TIAB("scoping review*") OR TIAB("systematic review*") OR TIAB("systematic literature review*") OR TIAB("systematic narrative review*") OR TIAB("systematic qualitative review*") OR TIAB("systematic evidence review*") OR TIAB("systematic quantitative review*") OR TIAB("systematic meta-review*") OR TIAB("systematic critical review*") OR TIAB("systematic mapping review*") OR TIAB("systematic cochrane review*") OR TIAB("systematic electronic literature search*") OR TIAB("PRISMA") OR TIAB("systematic descriptive review*") OR TIAB("systematic analys*") OR TIAB("targeted literature review*") OR TIAB("meta-synthes*") OR TIAB("comprehensive review*") OR TIAB("mixed studies review*") OR TIAB("sistematic review*") OR TIAB("umbrella review*") OR TIAB("mini-review*") OR TIAB("rapid literature review*") OR TIAB("rapid review*") OR TIAB("integrative review*") OR TIAB("systematic and critical analysis review*") OR TIAB("systematically review evidence") OR TIAB("systematic search*") OR TIAB("systematic methodological review*") OR TIAB("metaanalys*") OR TIAB("meta-analytic review*") OR TIAB("meta-analys*") OR TIAB("cochrane review*")).

SCOPUS :

(KEY ({Acne Vulgaris}) OR KEY ({acquired immune deficiency syndrome}) OR KEY ({Acquired Immunodeficiency Syndrome}) OR KEY ({acromegaly}) OR KEY ({acute leukemia}) OR KEY ({Addison Disease}) OR KEY ({Adrenal Gland Diseases}) OR KEY ({alopecia}) OR KEY ({Alzheimer disease}) OR KEY ({Anemia, Hemolytic}) OR KEY ({Anemia, Pernicious}) OR KEY ({Angina pectoris}) OR KEY ({antiphospholipid syndrome}) OR KEY ({aortic arch syndrome}) OR KEY ({Arteritis}) OR KEY ({arthritis}) OR KEY ({asthma}) OR KEY ({atrophic gastritis}) OR KEY ({atrophic rhinitis}) OR KEY ({axial spondyloarthritis}) OR KEY ({Back pain }) OR KEY ({backache}) OR KEY ({Behcet disease}) OR KEY ({benign paroxysmal positional vertigo}) OR KEY ({bladder tumor}) OR KEY ({Bone Marrow Diseases}) OR KEY ({bone marrow suppression}) OR KEY ({bone tumor}) OR KEY ({Brain Neoplasms}) OR KEY ({brain tumor}) OR KEY ({Bursitis}) OR KEY ({Cardiovascular Diseases}) OR KEY ({Cataract}) OR KEY ({Celiac disease}) OR KEY ({cerebrovascular accident}) OR KEY ({chronic bronchitis}) OR KEY ({chronic fatigue syndrome}) OR KEY ({chronic hepatitis B}) OR KEY ({chronic hepatitis C}) OR KEY ({chronic kidney failure}) OR KEY ({chronic myeloid leukemia}) OR KEY ({chronic obstructive lung disease}) OR KEY ({chronic pain}) OR KEY ({Churg Strauss syndrome}) OR KEY ({Churg-Strauss Syndrome}) OR KEY ({Colitis, Ulcerative}) OR KEY ({colon polyp}) OR KEY ({colon tumor}) OR KEY ({Colonic Diseases, Functional}) OR KEY ({Colonic polyps}) OR KEY ({complex regional pain syndrome type I}) OR KEY ({Crohn Disease}) OR KEY ({cryoglobulinemia}) OR KEY ({Crystal Arthropathies}) OR KEY ({crystal arthropathy}) OR KEY ({Cushing syndrome}) OR KEY ({Dementia, Vascular}) OR KEY ({diabetes insipidus}) OR KEY ({diabetes mellitus}) OR KEY ({diffuse large B cell lymphoma}) OR KEY ({Discitis}) OR KEY ({diskitis}) OR KEY ({Down syndrome}) OR KEY ({eczema}) OR KEY ({Ehlers Danlos syndrome}) OR KEY ({Ehlers-Danlos Syndrome}) OR KEY ({Emphysema}) OR KEY ({Endocarditis}) OR KEY ({epicondylitis}) OR KEY ({Epilepsy}) OR KEY ({Esophageal Achalasia}) OR KEY ({esophagus tumor}) OR KEY ({Ethmoid Sinusitis}) OR KEY ({Fatigue Syndrome, Chronic}) OR KEY ({fibromyalgia}) OR KEY ({gallbladder tumor}) OR KEY ({Gastritis,

Atrophic}) OR KEY ({gastroesophageal reflux}) OR KEY ({giant cell arteritis}) OR KEY ({Goodpasture syndrom}) OR KEY ({Granulomatosis with Polyangiitis}) OR KEY ({Graves Disease}) OR KEY ({Guillain Barre syndrome}) OR KEY ({Guillain-Barre Syndrome}) OR KEY ({Hashimoto Disease}) OR KEY ({head and neck tumor}) OR KEY ({Headache Disorders}) OR KEY ({heart arrhythmia}) OR KEY ({Heart diseases}) OR KEY ({Heart failure}) OR KEY ({heart infarction}) OR KEY ({heart muscle conduction disturbance}) OR KEY ({hemochromatosis}) OR KEY ({hemolytic anemia}) OR KEY ({hemophilia A}) OR KEY ({Hepatitis, Chronic}) OR KEY ({Hernia, Hiatal}) OR KEY ({herpes simplex}) OR KEY ({hiatus hernia}) OR KEY ({Hidradenitis Suppurativa}) OR KEY ({Histiocytosis}) OR KEY ({Hodgkin disease}) OR KEY ({hyperaldosteronism}) OR KEY ({Hypercholesterolemia}) OR KEY ({Hyperpituitarism}) OR KEY ({hyperprolactinemia}) OR KEY ({Hypertension}) OR KEY ({hyperthyroidism}) OR KEY ({hypophysis tumor}) OR KEY ({hypothyroidism}) OR KEY ({inflammation of the lungs}) OR KEY ({interstitial lung disease}) OR KEY ({Intervertebral Disc Displacement}) OR KEY ({intervertebral disk hernia}) OR KEY ({irritable colon}) OR KEY ({Ischemic Attack, Transient}) OR KEY ({Kidney Calculi}) OR KEY ({Kidney Failure, Chronic}) OR KEY ({kidney polycystic disease}) OR KEY ({kidney tumor}) OR KEY ({Leiomyoma}) OR KEY ({leukemia}) OR KEY ({Leukemia, Myelogenous, Chronic, BCR-ABL Positive}) OR KEY ({Leukemia, Prolymphocytic, T-Cell}) OR KEY ({Linear IgA Bullous Dermatosis}) OR KEY ({Liver Cirrhosis}) OR KEY ({liver tumor}) OR KEY ({low back pain}) OR KEY ({Low Tension Glaucoma}) OR KEY ({Lung diseases , Obstructive}) OR KEY ({Lung Diseases, Interstitial}) OR KEY ({lung embolism}) OR KEY ({lung tumor}) OR KEY ({Lupus Erythematosus, Systemic}) OR KEY ({Lyme Disease}) OR KEY ({Lymphoma, Large B-Cell, Diffuse}) OR KEY ({Macular Degeneration}) OR KEY ({melanoma}) OR KEY ({Meniere disease}) OR KEY ({microscopic polyangiitis}) OR KEY ({multiinfarct dementia}) OR KEY ({Multiple Myeloma}) OR KEY ({multiple sclerosis}) OR KEY ({Muscular Diseases}) OR KEY ({muscular dystrophy}) OR KEY ({myasthenia gravis}) OR KEY ({myelodysplastic syndrome}) OR KEY ({Myelodysplastic Syndromes}) OR KEY ({myeloma}) OR KEY ({Myeloproliferative Syndrome, Transient}) OR KEY ({Nasal Polyps}) OR KEY ({neck pain}) OR KEY ({Nephritis}) OR KEY ({nephrolithiasis}) OR KEY ({nose polyp}) OR KEY ({Obesity}) OR KEY ({Osteitis Deformans}) OR KEY ({osteoarthritis}) OR KEY ({otosclerosis}) OR KEY ({Pacemaker, Artificial/adverse effects}) OR KEY ({Paget bone disease}) OR KEY ({pancreas tumor}) OR KEY ({paranasal sinusitis}) OR KEY ({Parkinson disease}) OR KEY ({Periarthritis}) OR KEY ({pernicious anemia}) OR KEY ({pheochromocytoma}) OR KEY ({Phlebitis}) OR KEY ({polyarteritis nodosa}) OR KEY ({Polycystic Kidney Diseases}) OR KEY ({porphyria}) OR KEY ({Porphyrias}) OR KEY ({prolymphocytic leukemia}) OR KEY ({Psoriasis}) OR KEY ({psoriatic arthritis}) OR KEY ({Pulmonary arterial hypertension}) OR KEY ({Pulmonary Embolism}) OR KEY ({pulmonary hypertension}) OR KEY ({Purpura, Thrombocytopenic}) OR KEY ({rectum tumor}) OR KEY ({Reflex Sympathetic Dystrophy}) OR KEY ({retina disease}) OR KEY ({Retinal Diseases}) OR KEY ({rheumatoid arthritis}) OR KEY ({Rhinitis, Atrophic}) OR KEY ({rosacea}) OR KEY ({sarcoidosis}) OR KEY ({sciatica}) OR KEY ({Scleritis}) OR KEY ({Scleroderma, Systemic}) OR KEY ({Scoliosis}) OR KEY ({sickle cell anemia}) OR KEY ({Sjoegren syndrome}) OR KEY ({skin tumor}) OR KEY ({Sleep Apnea Syndromes}) OR KEY ({sleep disordered breathing}) OR KEY ({squamous cell carcinoma}) OR KEY ({stomach diverticulosis}) OR KEY ({Stomach diverticulum}) OR KEY ({stomach tumor}) OR KEY ({Stroke}) OR KEY ({suppurative hidradenitis}) OR KEY ({systemic lupus erythematosus}) OR KEY ({systemic sclerosis}) OR KEY ({tendinitis}) OR KEY ({thrombocytopenic purpura}) OR KEY ({Thrombotic Microangiopathies}) OR KEY ({thrombotic microangiopathy}) OR KEY ({Thyroid Diseases}) OR KEY ({thyroid tumor}) OR KEY ({transient ischemic attack}) OR KEY ({Trigeminal Neuralgia}) OR KEY ({tuberculosis}) OR KEY ({ulcerative colitis}) OR KEY ({urine incontinence}) OR KEY ({urticaria}) OR KEY ({uveitis}) OR KEY ({valvular heart disease}) OR KEY ({Vertigo}) OR KEY ({Vision Disorders}) OR KEY ({visual disorder}) OR KEY ({Vitiligo}) OR KEY ({vitreous body detachment}) OR KEY ({Vitreous Detachment}) OR KEY ({Wegener granulomatosis}) OR TITLE-ABS ({Basal Cell Epithelioma}) OR

TITLE-ABS ({Basal Cell Epitheliomas}) OR TITLE-ABS ({Brain Metastase}) OR TITLE-ABS ({Brain Metastases}) OR TITLE-ABS ({cerebroma}) OR TITLE-ABS ({Colon Adenocarcinoma}) OR TITLE-ABS ({Colon Adenocarcinomas}) OR TITLE-ABS ({dermatoma}) OR TITLE-ABS ({encephalophyma}) OR TITLE-ABS ({hypophysoma}) OR TITLE-ABS ({kidney mass}) OR TITLE-ABS ({kidney neoplastic mass}) OR TITLE-ABS ({malignant melanomatosis}) OR TITLE-ABS ({melanocarcinoma}) OR TITLE-ABS ({Melanoma}) OR TITLE-ABS ({melanomalignoma}) OR TITLE-ABS ({Melanomas}) OR TITLE-ABS ({naevocarcinoma}) OR TITLE-ABS ({neoplastic lung}) OR TITLE-ABS ({neoplastic mammary gland}) OR TITLE-ABS ({nephroma}) OR TITLE-ABS ({nevocarcinoma}) OR TITLE-ABS ({Pituitary Adenoma}) OR TITLE-ABS ({Pituitary Adenomas}) OR TITLE-ABS ({rectal mass}) OR TITLE-ABS ({rectum mass}) OR TITLE-ABS ({renal mass}) OR TITLE-ABS ({renal neoplastic mass}) OR TITLE-ABS ({reninoma}) OR TITLE-ABS ({Rodent Ulcer}) OR TITLE-ABS ({Rodent Ulcers}) OR TITLE-ABS ({squamous cell epithelioma}) OR TITLE-ABS ({squamous epithelioma}) OR TITLE-ABS ({Thyroid Adenoma}) OR TITLE-ABS ({Thyroid Adenomas}) OR TITLE-ABS ({angina}) OR TITLE-ABS ({anginal attack}) OR TITLE-ABS ({angiocardiopathy}) OR TITLE-ABS ({angiocardiovascular disease}) OR TITLE-ABS ({angiocardiovascular diseases}) OR TITLE-ABS ({Angor Pectori}) OR TITLE-ABS ({Aortic Valve Diseases}) OR TITLE-ABS ({Aortic Valve Disorder}) OR TITLE-ABS ({Aortic Valve Disorders}) OR TITLE-ABS ({Arrhythmia}) OR TITLE-ABS ({Arrythmia}) OR TITLE-ABS ({Artificial Cardiac Pacemaker}) OR TITLE-ABS ({Artificial Cardiac Pacemakers}) OR TITLE-ABS ({Artificial Pacemaker}) OR TITLE-ABS ({Artificial Pacemakers}) OR TITLE-ABS ({Atrial Fibrillation}) OR TITLE-ABS ({Atrial Fibrillations}) OR TITLE-ABS ({Auricular Fibrillation}) OR TITLE-ABS ({Auricular Fibrillations}) OR TITLE-ABS ({backward failure heart}) OR TITLE-ABS ({backward failure, heart}) OR TITLE-ABS ({Cardiac Arrest}) OR TITLE-ABS ({Cardiac Arrhythmias}) OR TITLE-ABS ({cardiac backward failure}) OR TITLE-ABS ({cardiac conduction system disease}) OR TITLE-ABS ({cardiac decompensation}) OR TITLE-ABS ({Cardiac Disorders}) OR TITLE-ABS ({cardiac disrhythmia}) OR TITLE-ABS ({Cardiac Dysrhythmia}) OR TITLE-ABS ({cardiac failure}) OR TITLE-ABS ({cardiac failures}) OR TITLE-ABS ({cardiac incompetence}) OR TITLE-ABS ({cardiac incompetences}) OR TITLE-ABS ({cardiac infarct}) OR TITLE-ABS ({cardiac insufficien*}) OR TITLE-ABS ({cardiac stand still}) OR TITLE-ABS ({cardiac valve defect}) OR TITLE-ABS ({cardiac valve disease}) OR TITLE-ABS ({cardial decompensation}) OR TITLE-ABS ({cardial infarct}) OR TITLE-ABS ({cardial insufficiency}) OR TITLE-ABS ({cardial valve disease}) OR TITLE-ABS ({cardiovascular complication*}) OR TITLE-ABS ({cardiovascular decompensation}) OR TITLE-ABS ({cardiovascular disease}) OR TITLE-ABS ({cardiovascular diseases}) OR TITLE-ABS ({cardiovascular disorder}) OR TITLE-ABS ({cardiovascular disorders}) OR TITLE-ABS ({cardiovascular disturbance}) OR TITLE-ABS ({cardiovascular disturbances}) OR TITLE-ABS ({cardiovascular disturbancescardiovascular lesion*}) OR TITLE-ABS ({cardiovascular failure}) OR TITLE-ABS ({cardiovascular failures}) OR TITLE-ABS ({cardiovascular incompetence}) OR TITLE-ABS ({cardiovascular incompetences}) OR TITLE-ABS ({cardiovascular insufficien*}) OR TITLE-ABS ({cardiovascular lesion*}) OR TITLE-ABS ({cardiovascular syndrome}) OR TITLE-ABS ({cardiovascular syndromes}) OR TITLE-ABS ({cardiovascular vegetative disorder}) OR TITLE-ABS ({cardiovascular vegetative disorders}) OR TITLE-ABS ({cholesteremia}) OR TITLE-ABS ({cholesterinemia}) OR TITLE-ABS ({cholesterolemia}) OR TITLE-ABS ({Circulatory Disorders}) OR TITLE-ABS ({complication cardiovascular}) OR TITLE-ABS ({conduction defect}) OR TITLE-ABS ({conduction defects}) OR TITLE-ABS ({conduction disease}) OR TITLE-ABS ({conduction diseases}) OR TITLE-ABS ({conduction disorder}) OR TITLE-ABS ({conduction disorders}) OR TITLE-ABS ({conduction disturbance}) OR TITLE-ABS ({conduction disturbances}) OR TITLE-ABS ({Coronary Disorders}) OR TITLE-ABS ({decompensatio cordis}) OR TITLE-ABS ({decompensation, heart}) OR TITLE-ABS ({ectopic heart rhythm}) OR TITLE-ABS ({ectopic rhythm}) OR TITLE-ABS ({Elevated Cholesterol}) OR TITLE-ABS ({Elevated Cholesterols}) OR TITLE-ABS ({heart aberrant conduction}) OR TITLE-ABS ({heart arrhytmia}) OR TITLE-ABS ({Heart Attack}) OR TITLE-ABS ({Heart Attacks}) OR TITLE-ABS ({heart

backward failure}) OR TITLE-ABS ({heart decompensation}) OR TITLE-ABS ({heart disease}) OR TITLE-ABS ({heart diseases}) OR TITLE-ABS ({heart disorder}) OR TITLE-ABS ({heart disorders}) OR TITLE-ABS ({heart dysrhythmia}) OR TITLE-ABS ({heart ectopic beat}) OR TITLE-ABS ({heart ectopic ventricle contraction}) OR TITLE-ABS ({heart failure}) OR TITLE-ABS ({heart failures}) OR TITLE-ABS ({heart incompetence}) OR TITLE-ABS ({heart infarct}) OR TITLE-ABS ({heart insufficien*}) OR TITLE-ABS ({heart rhythm disorder}) OR TITLE-ABS ({heart valve abnormalities}) OR TITLE-ABS ({heart valve abnormality}) OR TITLE-ABS ({heart valve defect}) OR TITLE-ABS ({heart valve degeneration}) OR TITLE-ABS ({Heart Valve Disease}) OR TITLE-ABS ({heart valve diseases}) OR TITLE-ABS ({heart valve lesion}) OR TITLE-ABS ({Heart Valvular Disease}) OR TITLE-ABS ({Heart Valvular Diseases}) OR TITLE-ABS ({High Blood Pressure}) OR TITLE-ABS ({High Blood Pressures}) OR TITLE-ABS ({High Cholesterol Level}) OR TITLE-ABS ({High Cholesterol Levels}) OR TITLE-ABS ({Hypercholesteremia}) OR TITLE-ABS ({Hypercholesteremias}) OR TITLE-ABS ({hypercholesterinaemia}) OR TITLE-ABS ({hypercholesterinemia}) OR TITLE-ABS ({hypercholesterolaemia}) OR TITLE-ABS ({Hypercholesterolemias}) OR TITLE-ABS ({hypertensive disease}) OR TITLE-ABS ({hypertensive effect}) OR TITLE-ABS ({hypertensive pulmonary vascular disease}) OR TITLE-ABS ({hypertensive response}) OR TITLE-ABS ({insufficientia cardis}) OR TITLE-ABS ({insufficientio cardis}) OR TITLE-ABS ({lung embolism}) OR TITLE-ABS ({lung embolization}) OR TITLE-ABS ({lung embolus}) OR TITLE-ABS ({lung emboly}) OR TITLE-ABS ({lung microembolism}) OR TITLE-ABS ({lung microembolization}) OR TITLE-ABS ({lung microembolus}) OR TITLE-ABS ({lung thromboembolism}) OR TITLE-ABS ({major adverse cardiovascular event*}) OR TITLE-ABS ({Myocardial Failure}) OR TITLE-ABS ({Myocardial Infarct}) OR TITLE-ABS ({myocardial insufficiency}) OR TITLE-ABS ({Myocardial lnfarcts}) OR TITLE-ABS ({myocardium infarct}) OR TITLE-ABS ({Periphlebitides}) OR TITLE-ABS ({Periphlebitis}) OR TITLE-ABS ({perivenous infection}) OR TITLE-ABS ({Phlebitides}) OR TITLE-ABS ({phlebitis superficialis}) OR TITLE-ABS ({post phlebitis syndrome}) OR TITLE-ABS ({postphlebitic disease}) OR TITLE-ABS ({postphlebitis syndrome}) OR TITLE-ABS ({Pulmonary Embolism}) OR TITLE-ABS ({Pulmonary Embolisms}) OR TITLE-ABS ({pulmonary embolization}) OR TITLE-ABS ({pulmonary embolus}) OR TITLE-ABS ({pulmonary hypertensive diseases}) OR TITLE-ABS ({pulmonary hypertensive disorder}) OR TITLE-ABS ({pulmonary hypertensive disorders}) OR TITLE-ABS ({pulmonary microembolism}) OR TITLE-ABS ({pulmonary thromboembolic disease}) OR TITLE-ABS ({Pulmonary Thromboembolism}) OR TITLE-ABS ({Pulmonary Thromboembolisms}) OR TITLE-ABS ({Raynauds Disease}) OR TITLE-ABS ({Stenocardia}) OR TITLE-ABS ({Stenocardias}) OR TITLE-ABS ({superficial phlebitis}) OR TITLE-ABS ({valvulopathies}) OR TITLE-ABS ({valvulopathy}) OR TITLE-ABS ({Vascular Disorders}) OR TITLE-ABS ({Vasculitis}) OR TITLE-ABS ({vein inflammation}) OR TITLE-ABS ({venitis}) OR TITLE-ABS ({venous inflammation}) OR TITLE-ABS ({vitium cordis}) OR TITLE-ABS ({Chronic Infectious Mononucleosis-Like Syndromes}) OR TITLE-ABS ({chronic intractable pain}) OR TITLE-ABS ({Royal Free Diseases}) OR TITLE-ABS ({Systemic Exertion Intolerance Diseases}) OR TITLE-ABS ({Chronic Pain}) OR TITLE-ABS ({Chronic Pains}) OR TITLE-ABS ({Achalasia}) OR TITLE-ABS ({Achalasias}) OR TITLE-ABS ({alcohol liver injury}) OR TITLE-ABS ({alcoholic liver}) OR TITLE-ABS ({Atrophic Gastritides}) OR TITLE-ABS ({Atrophic Gastritis}) OR TITLE-ABS ({autoimmune gastritis}) OR TITLE-ABS ({bowel syndrome}) OR TITLE-ABS ({cardiac herniation}) OR TITLE-ABS ({cardioesophageal reflux}) OR TITLE-ABS ({cardiooesophageal reflux}) OR TITLE-ABS ({Cardiospasm}) OR TITLE-ABS ({Cardiospasms}) OR TITLE-ABS ({Celiac Disease}) OR TITLE-ABS ({celiac syndrome}) OR TITLE-ABS ({chronic inflammatory bowel diseases}) OR TITLE-ABS ({chronic ulceration colon}) OR TITLE-ABS ({cirrhosis}) OR TITLE-ABS ({cleron disease}) OR TITLE-ABS ({coeliac disease}) OR TITLE-ABS ({coeliac syndrome}) OR TITLE-ABS ({coeliaky}) OR TITLE-ABS ({Colitis Gravis}) OR TITLE-ABS ({colitis ulcerativa}) OR TITLE-ABS ({colitis ulcerosa}) OR TITLE-ABS ({colon disease}) OR TITLE-ABS ({colon diseases}) OR TITLE-ABS ({colon disorder}) OR TITLE-ABS ({colon disorders}) OR TITLE-ABS ({colon juvenile polyp}) OR TITLE-ABS ({colon polypoid lesion}) OR TITLE-ABS ({colon spasm})

OR TITLE-ABS ({chronic colon ulceration}) OR TITLE-ABS ({Colonic Polyp}) OR TITLE-ABS ({Colonic Polyps}) OR TITLE-ABS ({colonospasm}) OR TITLE-ABS ({Crohns Disease}) OR TITLE-ABS ({Crohn's Disease}) OR TITLE-ABS ({Crohn's Enteritis}) OR TITLE-ABS ({digestive disease}) OR TITLE-ABS ({digestive diseases}) OR TITLE-ABS ({digestive disorder}) OR TITLE-ABS ({digestive disorders}) OR TITLE-ABS ({digestive system disorders}) OR TITLE-ABS ({enteritis regionalis}) OR TITLE-ABS ({Esophageal Hernia*}) OR TITLE-ABS ({Esophageal Reflux}) OR TITLE-ABS ({esophageal regurgitation}) OR TITLE-ABS ({esophagogastric reflux}) OR TITLE-ABS ({esophagus hernia}) OR TITLE-ABS ({esophagus reflux}) OR TITLE-ABS ({functional colonic diseases}) OR TITLE-ABS ({Gastric Acid Reflux}) OR TITLE-ABS ({gastric atrophy}) OR TITLE-ABS ({gastric regurgitation}) OR TITLE-ABS ({Gastroduodenal Ulcer}) OR TITLE-ABS ({Gastroduodenal Ulcers}) OR TITLE-ABS ({gastroesophageal reflex}) OR TITLE-ABS ({gastroesophageal regurgitation}) OR TITLE-ABS ({gastroesophagus reflux}) OR TITLE-ABS ({gastrointestinal disease}) OR TITLE-ABS ({gastrointestinal diseases}) OR TITLE-ABS ({gastrointestinal disorder}) OR TITLE-ABS ({gastrointestinal disorders}) OR TITLE-ABS ({gastrointestinal dysfunction*}) OR TITLE-ABS ({gastrooesophageal reflex}) OR TITLE-ABS ({gastrooesophageal reflux}) OR TITLE-ABS ({Gastro-oesophageal Reflux}) OR TITLE-ABS ({gastrooesophageal regurgitation}) OR TITLE-ABS ({gee herter disease}) OR TITLE-ABS ({gee thaysen disease}) OR TITLE-ABS ({GERD}) OR TITLE-ABS ({Gluten Enteropathies}) OR TITLE-ABS ({Gluten Enteropathy}) OR TITLE-ABS ({gluten induced enteropathy}) OR TITLE-ABS ({gluten intolerance}) OR TITLE-ABS ({Gluten Sensitive Enteropathy}) OR TITLE-ABS ({Gluten-Sensitive Enteropathies}) OR TITLE-ABS ({Gluten-Sensitive Enteropathy}) OR TITLE-ABS ({Granulomatous Colitis}) OR TITLE-ABS ({Granulomatous Enteritis}) OR TITLE-ABS ({hernia hiatus esophagi}) OR TITLE-ABS ({hiatal diaphragmatic hernia}) OR TITLE-ABS ({Hiatal Hernia*}) OR TITLE-ABS ({hiatus diaphragmatic hernia}) OR TITLE-ABS ({hiatus esophagi hernia}) OR TITLE-ABS ({Hiatus Hernia*}) OR TITLE-ABS ({hiatus oesophageal hernia}) OR TITLE-ABS ({hiatus oesophagus hernia}) OR TITLE-ABS ({huebner herter disease}) OR TITLE-ABS ({Idiopathic Proctocolitis}) OR TITLE-ABS ({Ileocolitis}) OR TITLE-ABS ({Inflammatory Bowel Disease}) OR TITLE-ABS ({intestinal infantilism}) OR TITLE-ABS ({Irritable Bowel Syndromes}) OR TITLE-ABS ({Irritable Colon}) OR TITLE-ABS ({Liver Fibrosis}) OR TITLE-ABS ({Marginal Ulcer}) OR TITLE-ABS ({Marginal Ulcers}) OR TITLE-ABS ({Megaesophagus}) OR TITLE-ABS ({morbus crohn}) OR TITLE-ABS ({mucomembraneous colitis}) OR TITLE-ABS ({mucomembranous colitis}) OR TITLE-ABS ({mucosal colitis}) OR TITLE-ABS ({Mucous Colitides}) OR TITLE-ABS ({Mucous Colitis}) OR TITLE-ABS ({oesophageal reflux}) OR TITLE-ABS ({oesophageal regurgitation}) OR TITLE-ABS ({oesophagogastric reflux}) OR TITLE-ABS ({oesophagus reflux}) OR TITLE-ABS ({Paraesophageal Hernia}) OR TITLE-ABS ({para-esophageal hernia}) OR TITLE-ABS ({Paraesophageal Hernias}) OR TITLE-ABS ({paraesophageal herniation}) OR TITLE-ABS ({para-esophageal herniation}) OR TITLE-ABS ({paraoesophageal hernia}) OR TITLE-ABS ({para-oesophageal hernia}) OR TITLE-ABS ({paraoesophageal herniation}) OR TITLE-ABS ({para-oesophageal herniation}) OR TITLE-ABS ({Peptic Ulcers}) OR TITLE-ABS ({Regional Enteritis}) OR TITLE-ABS ({regional enterocolitis}) OR TITLE-ABS ({Regional Ileitides}) OR TITLE-ABS ({Regional Ileitis}) OR TITLE-ABS ({regurgitation, gastroesophageal}) OR TITLE-ABS ({spastic colitis}) OR TITLE-ABS ({spastic colon}) OR TITLE-ABS ({Sprue}) OR TITLE-ABS ({stomach hernia}) OR TITLE-ABS ({Terminal Ileitis}) OR TITLE-ABS ({Ulcerative Colitis}) OR TITLE-ABS ({ulcerative colorectitis}) OR TITLE-ABS ({ulcerative procto colitis}) OR TITLE-ABS ({ulcerative proctocolitis}) OR TITLE-ABS ({ulcerous colitis}) OR TITLE-ABS ({unstable colon}) OR TITLE-ABS ({acromegalia}) OR TITLE-ABS ({Acromegalies}) OR TITLE-ABS ({acromegalism}) OR TITLE-ABS ({Acromegaly}) OR TITLE-ABS ({Addison Disease}) OR TITLE-ABS ({Addisons Disease}) OR TITLE-ABS ({Addison's Disease}) OR TITLE-ABS ({adipose tissue hyperplasia}) OR TITLE-ABS ({adipositas}) OR TITLE-ABS ({adiposity}) OR TITLE-ABS ({adrenal cortex hyperplasia}) OR TITLE-ABS ({adrenal cortical hyperplasia}) OR TITLE-ABS ({adrenal failure}) OR TITLE-ABS ({adrenal failures}) OR TITLE-ABS ({adrenal gland disorders}) OR TITLE-ABS ({adrenocortical

hyperplasia}) OR TITLE-ABS ({adrenocorticohyperplasia}) OR TITLE-ABS ({akromegalia}) OR TITLE-ABS ({Aldosteronism}) OR TITLE-ABS ({antidiuretic hormone insufficiency}) OR TITLE-ABS ({arenocortical hyperplasia}) OR TITLE-ABS ({Basedow Disease}) OR TITLE-ABS ({basedow syndrome}) OR TITLE-ABS ({Basedows Disease}) OR TITLE-ABS ({Basedow's Disease}) OR TITLE-ABS ({chromaffin paraganglioma}) OR TITLE-ABS ({chronic autoimmune thyroiditis}) OR TITLE-ABS ({Chronic Lymphocytic Thyroiditides}) OR TITLE-ABS ({Chronic Lymphocytic Thyroiditis}) OR TITLE-ABS ({Conn Syndrome}) OR TITLE-ABS ({Conns Syndrome}) OR TITLE-ABS ({Conn's Syndrome}) OR TITLE-ABS ({corpulency}) OR TITLE-ABS ({Cushing syndrome}) OR TITLE-ABS ({Cushings syndrome}) OR TITLE-ABS ({Cushing's Syndrome}) OR TITLE-ABS ({diabetes}) OR TITLE-ABS ({diabetic}) OR TITLE-ABS ({endocrinal disease}) OR TITLE-ABS ({endocrinal diseases}) OR TITLE-ABS ({endocrinal disorder}) OR TITLE-ABS ({endocrinal disorders}) OR TITLE-ABS ({endocrinal disturbance}) OR TITLE-ABS ({endocrinal disturbances}) OR TITLE-ABS ({endocrinal dysfunction*}) OR TITLE-ABS ({endocrine disease}) OR TITLE-ABS ({endocrine diseases}) OR TITLE-ABS ({endocrine disorder}) OR TITLE-ABS ({Endocrine Disorders}) OR TITLE-ABS ({endocrine disturbance}) OR TITLE-ABS ({endocrine disturbances}) OR TITLE-ABS ({endocrine dysfunction*}) OR TITLE-ABS ({endocrine gland disease}) OR TITLE-ABS ({endocrine gland diseases}) OR TITLE-ABS ({endocrine gland disorder}) OR TITLE-ABS ({endocrine gland disorders}) OR TITLE-ABS ({endocrine gland dysfunction*}) OR TITLE-ABS ({endocrine syndrome}) OR TITLE-ABS ({endocrine syndromes}) OR TITLE-ABS ({endocrine system disease}) OR TITLE-ABS ({endocrine system diseases}) OR TITLE-ABS ({endocrine system disorder}) OR TITLE-ABS ({endocrine system disorders}) OR TITLE-ABS ({endocrine system dysfunction*}) OR TITLE-ABS ({endocrinological disease}) OR TITLE-ABS ({endocrinological diseases}) OR TITLE-ABS ({endocrinological disorder}) OR TITLE-ABS ({endocrinological disorders}) OR TITLE-ABS ({endocrinological dysfunction*}) OR TITLE-ABS ({excess body weight}) OR TITLE-ABS ({Exophthalmic Goiter}) OR TITLE-ABS ({Exophthalmic Goiters}) OR TITLE-ABS ({exophthalmic goitre}) OR TITLE-ABS ({exophthalmic hyperthyroidism}) OR TITLE-ABS ({Extra Adrenal Pheochromocytoma}) OR TITLE-ABS ({Extra-Adrenal Pheochromocytoma}) OR TITLE-ABS ({familial hypoadrenocorticism}) OR TITLE-ABS ({fat overload syndrome}) OR TITLE-ABS ({feline hyperthyroidism}) OR TITLE-ABS ({Graves Disease}) OR TITLE-ABS ({Graves' Disease}) OR TITLE-ABS ({Graves hyperthyroidism}) OR TITLE-ABS ({graves syndrome}) OR TITLE-ABS ({Graves's disease}) OR TITLE-ABS ({Hashimoto Disease}) OR TITLE-ABS ({hashimoto goiter}) OR TITLE-ABS ({Hashimoto Struma}) OR TITLE-ABS ({Hashimoto Syndrome}) OR TITLE-ABS ({Hashimoto Thyroiditides}) OR TITLE-ABS ({Hashimoto Thyroiditis}) OR TITLE-ABS ({hashimoto thyroidosis}) OR TITLE-ABS ({Hashimotos Disease}) OR TITLE-ABS ({Hashimoto's Disease}) OR TITLE-ABS ({Hashimoto's Struma}) OR TITLE-ABS ({Hashimotos Syndrome}) OR TITLE-ABS ({Hashimoto's Syndrome}) OR TITLE-ABS ({Hashimoto's Syndromes}) OR TITLE-ABS ({Hashimotos thyroiditis}) OR TITLE-ABS ({Hashimoto's thyroiditis}) OR TITLE-ABS ({hormonal disease}) OR TITLE-ABS ({hormonal diseases}) OR TITLE-ABS ({hormonal disorder}) OR TITLE-ABS ({hormonal disorders}) OR TITLE-ABS ({hormonal dysfunction*}) OR TITLE-ABS ({hormone imbalance}) OR TITLE-ABS ({hyperaldosterone}) OR TITLE-ABS ({Hypercortisolism}) OR TITLE-ABS ({hypermineralocorticidism}) OR TITLE-ABS ({hypermineralocorticism}) OR TITLE-ABS ({Hyperprolactinaemia*}) OR TITLE-ABS ({Hyperprolactinemia}) OR TITLE-ABS ({Hyperprolactinemias}) OR TITLE-ABS ({hyperthyreoidism}) OR TITLE-ABS ({hyperthyreosis}) OR TITLE-ABS ({Hyperthyroid}) OR TITLE-ABS ({hyperthyroidea}) OR TITLE-ABS ({hyperthyroidosis}) OR TITLE-ABS ({Hyperthyroids}) OR TITLE-ABS ({hypogonadism}) OR TITLE-ABS ({hypothyreoidism}) OR TITLE-ABS ({hypothyreosis}) OR TITLE-ABS ({hypothyroidea}) OR TITLE-ABS ({hypothyroidism}) OR TITLE-ABS ({Hypothyroidisms}) OR TITLE-ABS ({hypothyroidosis}) OR TITLE-ABS ({hypothyrosis}) OR TITLE-ABS ({Inappropriate GH Secretion Syndrome}) OR TITLE-ABS ({Inappropriate GH Secretion Syndromes}) OR TITLE-ABS ({Inappropriate Growth Hormone Secretion Syndrome}) OR TITLE-ABS ({Inappropriate Growth Hormone Secretion

Syndromes}) OR TITLE-ABS ({Inappropriate Prolactin Secretion}) OR TITLE-ABS ({Inappropriate Secretion Prolactin}) OR TITLE-ABS ({lymphadenoid goiter}) OR TITLE-ABS ({megalakria}) OR TITLE-ABS ({mineralcorticoid excess syndrome}) OR TITLE-ABS ({obesitas}) OR TITLE-ABS ({obesity}) OR TITLE-ABS ({overweight}) OR TITLE-ABS ({phaeochromoblastoma}) OR TITLE-ABS ({phaeochromocytoma}) OR TITLE-ABS ({pheochromoblastoma}) OR TITLE-ABS ({Pheochromocytomas}) OR TITLE-ABS ({pheochromocytomata}) OR TITLE-ABS ({pheochromocytomatosis}) OR TITLE-ABS ({pheochromocytosis}) OR TITLE-ABS ({polycystic ovary syndrome}) OR TITLE-ABS ({polycystic ovary syndromes}) OR TITLE-ABS ({Primary Adrenal Insufficiency}) OR TITLE-ABS ({Primary Adrenocortical Insufficiencies}) OR TITLE-ABS ({Primary Adrenocortical Insufficiency}) OR TITLE-ABS ({Primary Hyperaldosteronism}) OR TITLE-ABS ({Primary Hyperthyroidism}) OR TITLE-ABS ({Primary Hypoadrenalism}) OR TITLE-ABS ({Primary Hypoadrenalisms}) OR TITLE-ABS ({Prolactin Hypersecretion Syndrome}) OR TITLE-ABS ({Prolactin Hypersecretion Syndromes}) OR TITLE-ABS ({Somatotropin Hypersecretion Syndrome}) OR TITLE-ABS ({Somatotropin Hypersecretion Syndromes}) OR TITLE-ABS ({struma hashimoto}) OR TITLE-ABS ({thyroid deficiency}) OR TITLE-ABS ({thyroid gland failure}) OR TITLE-ABS ({thyroid gland hyperfunction}) OR TITLE-ABS ({thyroid hyperfunction}) OR TITLE-ABS ({thyroid insufficiency}) OR TITLE-ABS ({Thyroid Stimulating Hormone Deficiency}) OR TITLE-ABS ({thyroideal hyperfunction}) OR TITLE-ABS ({Thyroid-Stimulating Hormone Deficiencies}) OR TITLE-ABS ({Thyroid-Stimulating Hormone Deficiency}) OR TITLE-ABS ({TSH Deficiencies}) OR TITLE-ABS ({TSH Deficiency}) OR TITLE-ABS ({Vasopressin Deficiency}) OR TITLE-ABS ({angioleiomyoma}) OR TITLE-ABS ({angiomyoma}) OR TITLE-ABS ({elastomyofibroma}) OR TITLE-ABS ({fibromyoma}) OR TITLE-ABS ({fibromyomas}) OR TITLE-ABS ({hemangioleiomyoma}) OR TITLE-ABS ({hemangiomyoma}) OR TITLE-ABS ({Impotence}) OR TITLE-ABS ({leimyoma}) OR TITLE-ABS ({Leiomyomas}) OR TITLE-ABS ({leyomyoma}) OR TITLE-ABS ({myofibroma}) OR TITLE-ABS ({myofibromatosis}) OR TITLE-ABS ({vascular leiomyoma}) OR TITLE-ABS ({addison anaemia}) OR TITLE-ABS ({addison anemia}) OR TITLE-ABS ({addisonian anaemia}) OR TITLE-ABS ({addisonian anemia}) OR TITLE-ABS ({ahf deficiency}) OR TITLE-ABS ({ahg deficiency}) OR TITLE-ABS ({anaemia perniciosa}) OR TITLE-ABS ({anemia perniciosa}) OR TITLE-ABS ({B12 deficiency anaemia}) OR TITLE-ABS ({B12 deficiency anemia}) OR TITLE-ABS ({B12 deficient anaemia}) OR TITLE-ABS ({B12 deficient anemia}) OR TITLE-ABS ({B12 vitamin deficiency anemia}) OR TITLE-ABS ({biermer anaemia}) OR TITLE-ABS ({biermer anemia}) OR TITLE-ABS ({biermer disease}) OR TITLE-ABS ({blood disease}) OR TITLE-ABS ({blood diseases}) OR TITLE-ABS ({blood disorder}) OR TITLE-ABS ({blood disorders}) OR TITLE-ABS ({blood dysfunction*}) OR TITLE-ABS ({classic haemophilia}) OR TITLE-ABS ({cobalamin deficiency anaemia}) OR TITLE-ABS ({cobalamin deficiency anemia}) OR TITLE-ABS ({congenital antihaemophilic factor deficiency}) OR TITLE-ABS ({congenital antihaemophilic globulin deficiency}) OR TITLE-ABS ({congenital antihemophilic factor deficiency}) OR TITLE-ABS ({congenital antihemophilic globulin deficiency}) OR TITLE-ABS ({drepanocytemia}) OR TITLE-ABS ({drepanocytic anaemia}) OR TITLE-ABS ({drepanocytic anemia}) OR TITLE-ABS ({drepanocytosis}) OR TITLE-ABS ({haematologic disease}) OR TITLE-ABS ({haematologic diseases}) OR TITLE-ABS ({haematologic disorder}) OR TITLE-ABS ({haematologic disorders}) OR TITLE-ABS ({haematologic dysfunction*}) OR TITLE-ABS ({haematological disease}) OR TITLE-ABS ({haematological diseases}) OR TITLE-ABS ({haematological disorder}) OR TITLE-ABS ({haematological disorders}) OR TITLE-ABS ({haematological dysfunction*}) OR TITLE-ABS ({haemochromatosis}) OR TITLE-ABS ({haemoglobin SS}) OR TITLE-ABS ({haemolytic anaemia}) OR TITLE-ABS ({haemolytic disease}) OR TITLE-ABS ({haemolytic syndrome}) OR TITLE-ABS ({haemophilia a}) OR TITLE-ABS ({haemophilia vera}) OR TITLE-ABS ({Hb SS disease}) OR TITLE-ABS ({heart hemochromatosis}) OR TITLE-ABS ({hemachromatosis}) OR TITLE-ABS ({hematologic disease}) OR TITLE-ABS ({hematologic diseases}) OR TITLE-ABS ({hematologic disorder}) OR TITLE-ABS ({hematologic disorders}) OR TITLE-ABS ({hematologic dysfunction*}) OR TITLE-ABS (

{hematopathy}) OR TITLE-ABS ({hemoglobin SS}) OR TITLE-ABS ({hemolytic anemia}) OR TITLE-ABS ({hemolytic disease}) OR TITLE-ABS ({hemolytic syndrome}) OR TITLE-ABS ({hemopathie}) OR TITLE-ABS ({hemopathies}) OR TITLE-ABS ({hemopathy}) OR TITLE-ABS ({hemophylia type a}) OR TITLE-ABS ({hereditary iron overload}) OR TITLE-ABS ({idiopathic hemochromatosis}) OR TITLE-ABS ({intermittent porphyria}) OR TITLE-ABS ({iron overload disease}) OR TITLE-ABS ({iron overload disorder}) OR TITLE-ABS ({iron overload syndrome}) OR TITLE-ABS ({latent porphyria}) OR TITLE-ABS ({lymphatic disease}) OR TITLE-ABS ({lymphatic diseases}) OR TITLE-ABS ({lymphatic disorder}) OR TITLE-ABS ({lymphatic disorders}) OR TITLE-ABS ({lymphatic dysfunction*}) OR TITLE-ABS ({macrocytic hyperchromic anaemia}) OR TITLE-ABS ({macrocytic hyperchromic anemia}) OR TITLE-ABS ({meniscocytosis}) OR TITLE-ABS ({pernicious anaemia}) OR TITLE-ABS ({pernicious anemia}) OR TITLE-ABS ({porphyrias}) OR TITLE-ABS ({porphyric disease}) OR TITLE-ABS ({primary anaemia}) OR TITLE-ABS ({primary anemia}) OR TITLE-ABS ({primary congenital hemochromatosis}) OR TITLE-ABS ({recklinghausen applebaum disease}) OR TITLE-ABS ({sickle anaemia}) OR TITLE-ABS ({sickle anemia}) OR TITLE-ABS ({sickle cell anaemia}) OR TITLE-ABS ({siderochromatosis}) OR TITLE-ABS ({sulfhemoglobinem*}) OR TITLE-ABS ({sulphaemoglobinaem*}) OR TITLE-ABS ({systemic porphyria}) OR TITLE-ABS ({thrombocytopaenia purpura}) OR TITLE-ABS ({thrombocytopenia purpura}) OR TITLE-ABS ({thrombotic purpura}) OR TITLE-ABS ({true haemophilia}) OR TITLE-ABS ({vitamin b 12 deficiency anaemia}) OR TITLE-ABS ({vitamin b 12 deficiency anemia}) OR TITLE-ABS ({a2 myeloma}) OR TITLE-ABS ({Acquired Hemolytic Anemias}) OR TITLE-ABS ({Acquired Immune Deficiency Syndrome Virus}) OR TITLE-ABS ({acquired immunodeficiency}) OR TITLE-ABS ({Addison Anemias}) OR TITLE-ABS ({Addisons Anemia}) OR TITLE-ABS ({Addison's Anemias}) OR TITLE-ABS ({AIDS}) OR TITLE-ABS ({aleukaemia}) OR TITLE-ABS ({aleukemia}) OR TITLE-ABS ({Arthriti*}) OR TITLE-ABS ({arthrochondritis}) OR TITLE-ABS ({arthrosynovitis}) OR TITLE-ABS ({B. burgdorferi Infection*}) OR TITLE-ABS ({bone marrow dysplasia}) OR TITLE-ABS ({Bone Marrow Fibros*}) OR TITLE-ABS ({Borrelia burgdorferi Infection*}) OR TITLE-ABS ({Bronze Diabete}) OR TITLE-ABS ({Bronzed Cirrhoses}) OR TITLE-ABS ({Chronic Hepatit*}) OR TITLE-ABS ({chronic myeloleukaemia}) OR TITLE-ABS ({chronic myeloleukemia}) OR TITLE-ABS ({Discitides}) OR TITLE-ABS ({discitis}) OR TITLE-ABS ({disk space infection}) OR TITLE-ABS ({Diskiti*}) OR TITLE-ABS ({DLBCL}) OR TITLE-ABS ({down disease}) OR TITLE-ABS ({Downs syndrome}) OR TITLE-ABS ({Down's syndrome}) OR TITLE-ABS ({Dysmyelopoietic Syndrome*}) OR TITLE-ABS ({endocardial inflammation}) OR TITLE-ABS ({Endocarditi*}) OR TITLE-ABS ({endo-carditis}) OR TITLE-ABS ({endocardium inflammation}) OR TITLE-ABS ({Erythremia*}) OR TITLE-ABS ({Factor 8 Deficienc*}) OR TITLE-ABS ({Factor VIII Deficienc*}) OR TITLE-ABS ({Factor VIII defiency}) OR TITLE-ABS ({Familial Hemochromatoses}) OR TITLE-ABS ({Familial Hemochromatosis}) OR TITLE-ABS ({Haemochromato}) OR TITLE-ABS ({Haemochromatos}) OR TITLE-ABS ({Haemolytic Anaemias}) OR TITLE-ABS ({HbS Disease}) OR TITLE-ABS ({Hematopoetic Myelodysplasias}) OR TITLE-ABS ({hemoblastoma}) OR TITLE-ABS ({Hemochromato}) OR TITLE-ABS ({Hemochromatos}) OR TITLE-ABS ({Hemoglobin S Disease}) OR TITLE-ABS ({Hemoglobin S Diseases}) OR TITLE-ABS ({Hemophilia}) OR TITLE-ABS ({hemophilias}) OR TITLE-ABS ({high-risk MDS}) OR TITLE-ABS ({Hodgkin disease*}) OR TITLE-ABS ({Hodgkin Granuloma*}) OR TITLE-ABS ({hodgkin sclerosis}) OR TITLE-ABS ({hodgkin's disease}) OR TITLE-ABS ({Hodgkins Disease*}) OR TITLE-ABS ({Hodgkins Granuloma}) OR TITLE-ABS ({Hodgkin's Granuloma}) OR TITLE-ABS ({HTLV-III}) OR TITLE-ABS ({Human Immunodeficiency Virus*}) OR TITLE-ABS ({Human T Cell Lymphotropic Virus Type III}) OR TITLE-ABS ({Human T Lymphotropic Virus Type III}) OR TITLE-ABS ({Human T-Cell Lymphotropic Virus Type III}) OR TITLE-ABS ({Human T-Lymphotropic Virus Type III}) OR TITLE-ABS ({infection by B. burgdorferi}) OR TITLE-ABS ({infection by Borrelia burgdorferi}) OR TITLE-ABS ({intervertebral disc infection}) OR TITLE-ABS ({intervertebral disk infection}) OR TITLE-ABS ({intervertebral disk inflammation}) OR TITLE-ABS ({Iron Storage Disorder*}) OR TITLE-ABS ({joint inflammation}) OR TITLE-ABS ({Koch Disease*}) OR TITLE-ABS (

{Kochs Disease*}) OR TITLE-ABS ({Koch's Disease*}) OR TITLE-ABS ({langdon down syndrome}) OR TITLE-ABS ({LAV-HTLV-III}) OR TITLE-ABS ({leucaemia}) OR TITLE-ABS ({leucemia}) OR TITLE-ABS ({Leucocythaemia*}) OR TITLE-ABS ({Leucocythemia*}) OR TITLE-ABS ({leukaemia}) OR TITLE-ABS ({Leukemia*}) OR TITLE-ABS ({Lyme borrelioses}) OR TITLE-ABS ({Lyme Borreliosis}) OR TITLE-ABS ({Lyme's borrelioses}) OR TITLE-ABS ({Lyme's borreliosis}) OR TITLE-ABS ({Lymes disease}) OR TITLE-ABS ({Lyme's disease}) OR TITLE-ABS ({Lymphadenopathy Associated Virus*}) OR TITLE-ABS ({Lymphadenopathy-Associated Virus*}) OR TITLE-ABS ({lymphogranuloma maligne}) OR TITLE-ABS ({lymphogranuloma malignum}) OR TITLE-ABS ({lymphogranulomatosis}) OR TITLE-ABS ({Malignant Granuloma*}) OR TITLE-ABS ({Malignant Lymphogranuloma}) OR TITLE-ABS ({Malignant Lymphogranulomas}) OR TITLE-ABS ({Microangiopathic Anemias}) OR TITLE-ABS ({Microangiopathic Hemolytic Anemias}) OR TITLE-ABS ({mongolian idiocy}) OR TITLE-ABS ({mongolism}) OR TITLE-ABS ({mongoloid idiocy}) OR TITLE-ABS ({mongoloidism}) OR TITLE-ABS ({morbus hodgkin}) OR TITLE-ABS ({myelodysplasia}) OR TITLE-ABS ({myelodysplastic disease}) OR TITLE-ABS ({myelodysplastic disorder}) OR TITLE-ABS ({Myelodysplastic Syndrome*}) OR TITLE-ABS ({Myelofibros*}) OR TITLE-ABS ({Myeloid Metaplasia*}) OR TITLE-ABS ({myeloplaxoma}) OR TITLE-ABS ({Myeloscleros*}) OR TITLE-ABS ({Nonleukemic Myelos*}) OR TITLE-ABS ({oligoarthritis}) OR TITLE-ABS ({Osler Vaquez Disease*}) OR TITLE-ABS ({Osler-Vaquez Disease*}) OR TITLE-ABS ({Pernicious Anemias}) OR TITLE-ABS ({Pigmentary Cirrhos*}) OR TITLE-ABS ({Polyarthrit*}) OR TITLE-ABS ({Polycythemia Ruba Vera*}) OR TITLE-ABS ({Polycythemia Rubra Vera*}) OR TITLE-ABS ({Primary Hemochromatosis}) OR TITLE-ABS ({Primary Polycythemia*}) OR TITLE-ABS ({reed sternberg disease}) OR TITLE-ABS ({Sickle Cell Anemia}) OR TITLE-ABS ({Sickle Cell Anemias}) OR TITLE-ABS ({Sickle Cell Disease}) OR TITLE-ABS ({Sickle Cell Diseases}) OR TITLE-ABS ({sickle cell disorder}) OR TITLE-ABS ({Sickle Cell Disorders}) OR TITLE-ABS ({Sickling Disorder Due to Hemoglobin S}) OR TITLE-ABS ({Spondylodisciti*}) OR TITLE-ABS ({Spondylodiskiti*}) OR TITLE-ABS ({TB disease}) OR TITLE-ABS ({TB infection}) OR TITLE-ABS ({Thrombocytopenic Purpura*}) OR TITLE-ABS ({Thrombopenic Purpura*}) OR TITLE-ABS ({Thrombotic Microangiopath*}) OR TITLE-ABS ({translocation 15 21 22}) OR TITLE-ABS ({trisomy 21 syndrome}) OR TITLE-ABS ({Troisier Hanot Chauffard Syndrome}) OR TITLE-ABS ({Troisier-Hanot-Chauffard Syndrome*}) OR TITLE-ABS ({Tuberculos*}) OR TITLE-ABS ({tuberculous infection}) OR TITLE-ABS ({tuberculous lesion}) OR TITLE-ABS ({Von Recklenhausen Applebaum Disease}) OR TITLE-ABS ({Von Recklenhausen-Applebaum Diseases}) OR TITLE-ABS ({Acute Autoimmune Neuropathies}) OR TITLE-ABS ({Acute Autoimmune Neuropathy}) OR TITLE-ABS ({Acute Cerebrovascular Accident*}) OR TITLE-ABS ({acute cerebrovascular lesion}) OR TITLE-ABS ({acute febrile polyneuritis}) OR TITLE-ABS ({acute focal cerebral vasculopathy}) OR TITLE-ABS ({Acute Infectious Polyneuritis}) OR TITLE-ABS ({Acute Inflammatory Demyelinating Polyneuropath*}) OR TITLE-ABS ({Acute Inflammatory Demyelinating Polyradiculoneuropath*}) OR TITLE-ABS ({Acute Inflammatory Polyneuropath*}) OR TITLE-ABS ({Acute Inflammatory Polyradiculoneuropath*}) OR TITLE-ABS ({acute postinfective polyradiculoneuropathy}) OR TITLE-ABS ({Acute Stroke*}) OR TITLE-ABS ({Alzeimer}) OR TITLE-ABS ({Alzheimer}) OR TITLE-ABS ({Alzeimers disease}) OR TITLE-ABS ({Alzeimer's disease}) OR TITLE-ABS ({Alzheimer's Disease}) OR TITLE-ABS ({Alzheimers Disease*}) OR TITLE-ABS ({Alzheimer's Diseases}) OR TITLE-ABS ({Alzheimer-Type Dementia}) OR TITLE-ABS ({Anti-MuSK Myasthenia Gravis}) OR TITLE-ABS ({Apoplex*}) OR TITLE-ABS ({Arteriosclerotic Dementia*}) OR TITLE-ABS ({Aura}) OR TITLE-ABS ({Auras}) OR TITLE-ABS ({autoimmune myasthenia gravis}) OR TITLE-ABS ({Binswanger Disease*}) OR TITLE-ABS ({Binswanger Encephalopath*}) OR TITLE-ABS ({Binswangers Disease*}) OR TITLE-ABS ({Binswanger's Disease*}) OR TITLE-ABS ({Binswangers Encephalopath*}) OR TITLE-ABS ({Binswanger's Encephalopath*}) OR TITLE-ABS ({brain accident}) OR TITLE-ABS ({brain attack}) OR TITLE-ABS ({brain blood flow disturbance}) OR TITLE-ABS ({brain insult}) OR TITLE-ABS ({brain insultus}) OR TITLE-ABS ({Brain TIA}) OR TITLE-ABS ({Brain Vascular Accident*}) OR TITLE-ABS (

{Cephalgia Syndrome}) OR TITLE-ABS ({Cephalgia Syndromes}) OR TITLE-ABS ({cerebral insult}) OR TITLE-ABS ({Cerebral Stroke*}) OR TITLE-ABS ({cerebral vascular accident}) OR TITLE-ABS ({cerebral vascular insufficiency}) OR TITLE-ABS ({cerebro vascular accident}) OR TITLE-ABS ({Cerebrovascular Accident}) OR TITLE-ABS ({Cerebrovascular Accidents}) OR TITLE-ABS ({cerebrovascular arrest}) OR TITLE-ABS ({cerebrovascular failure}) OR TITLE-ABS ({cerebrovascular injury}) OR TITLE-ABS ({cerebrovascular insufficiency}) OR TITLE-ABS ({cerebrovascular insult}) OR TITLE-ABS ({Cerebrovascular Stroke*}) OR TITLE-ABS ({cerebrum vascular accident}) OR TITLE-ABS ({Chronic Daily Headache}) OR TITLE-ABS ({Chronic Daily Headaches}) OR TITLE-ABS ({Chronic Headache}) OR TITLE-ABS ({Chronic Headaches}) OR TITLE-ABS ({Chronic Progressive Subcortical Encephalopath*}) OR TITLE-ABS ({comitial disease}) OR TITLE-ABS ({congenital atonic sclerotic muscular dystrophy}) OR TITLE-ABS ({congenital muscular dystrophy}) OR TITLE-ABS ({CVA}) OR TITLE-ABS ({CVAs}) OR TITLE-ABS ({diffuse cortical sclerosis}) OR TITLE-ABS ({Disseminated Sclerosis}) OR TITLE-ABS ({Epileps*}) OR TITLE-ABS ({epileptic}) OR TITLE-ABS ({Epileptiform Neuralgia*}) OR TITLE-ABS ({erb goldflam disease}) OR TITLE-ABS ({falling sickness}) OR TITLE-ABS ({Fisher syndrome}) OR TITLE-ABS ({Fothergill Disease}) OR TITLE-ABS ({Generalized Myasthenia Gravis}) OR TITLE-ABS ({Guillain Barre}) OR TITLE-ABS ({Guillain-Barré}) OR TITLE-ABS ({Guillain-Barre}) OR TITLE-ABS ({Guillain Barré Syndrome*}) OR TITLE-ABS ({Headache Disorder}) OR TITLE-ABS ({Headache Syndrome}) OR TITLE-ABS ({Headache Syndromes}) OR TITLE-ABS ({Idiopathic Parkinson Disease*}) OR TITLE-ABS ({idiopathic parkinsonism}) OR TITLE-ABS ({infectious neuronitis}) OR TITLE-ABS ({inflammatory acute polyradiculoneuropathy}) OR TITLE-ABS ({Inflammatory Polyneuropathy Acute}) OR TITLE-ABS ({insular sclerosis}) OR TITLE-ABS ({insultus cerebralis}) OR TITLE-ABS ({Intractable Headache}) OR TITLE-ABS ({Intractable Headaches}) OR TITLE-ABS ({ischaemic attack}) OR TITLE-ABS ({ischaemic cerebral attack}) OR TITLE-ABS ({ischaemic seizure}) OR TITLE-ABS ({ischemic attack}) OR TITLE-ABS ({ischemic cerebral attack}) OR TITLE-ABS ({ischemic seizure}) OR TITLE-ABS ({lacunar dementia}) OR TITLE-ABS ({Landry paralysis}) OR TITLE-ABS ({Landry syndrome}) OR TITLE-ABS ({Landry-Guillain-Barre Syndrome}) OR TITLE-ABS ({Lewy Body Parkinson Disease*}) OR TITLE-ABS ({maternal myasthenia gravis}) OR TITLE-ABS ({mini-stroke}) OR TITLE-ABS ({multiinfarct dementia}) OR TITLE-ABS ({multi-infarct dementia}) OR TITLE-ABS ({multiinfarction dementia}) OR TITLE-ABS ({multi-infarction dementia}) OR TITLE-ABS ({multiple sclerosis}) OR TITLE-ABS ({muscle dystrophia}) OR TITLE-ABS ({muscle dystrophy}) OR TITLE-ABS ({Muscle Specific Receptor Tyrosine Kinase Myasthenia Gravis}) OR TITLE-ABS ({Muscle Specific Tyrosine Kinase Antibody Positive Myasthenia Gravis}) OR TITLE-ABS ({Muscle-Specific Receptor Tyrosine Kinase Myasthenia Gravis}) OR TITLE-ABS ({Muscle-Specific Tyrosine Kinase Antibody Positive Myasthenia Gravis}) OR TITLE-ABS ({muscular dystrophia}) OR TITLE-ABS ({muscular dystrophies}) OR TITLE-ABS ({MuSK MG}) OR TITLE-ABS ({MuSK Myasthenia Gravis}) OR TITLE-ABS ({myasthenia gravis pseudoparalitica}) OR TITLE-ABS ({myasthenia gravis pseudoparalytica}) OR TITLE-ABS ({myodystrophia}) OR TITLE-ABS ({myodystrophy}) OR TITLE-ABS ({neonatal myasthenia gravis}) OR TITLE-ABS ({nervous disease}) OR TITLE-ABS ({nervous diseases}) OR TITLE-ABS ({nervous disorder}) OR TITLE-ABS ({nervous disorders}) OR TITLE-ABS ({nervous dysfunction*}) OR TITLE-ABS ({nervous system disease}) OR TITLE-ABS ({nervous system diseases}) OR TITLE-ABS ({nervous system disorder}) OR TITLE-ABS ({nervous system disorders}) OR TITLE-ABS ({nervous system dysfunction*}) OR TITLE-ABS ({neurologic disease}) OR TITLE-ABS ({neurologic diseases}) OR TITLE-ABS ({neurologic disorder}) OR TITLE-ABS ({neurologic disorders}) OR TITLE-ABS ({neurologic dysfunction*}) OR TITLE-ABS ({neurological disease}) OR TITLE-ABS ({neurological diseases}) OR TITLE-ABS ({neurological disorder}) OR TITLE-ABS ({neurological disorders}) OR TITLE-ABS ({neurological dysfunction*}) OR TITLE-ABS ({neuromuscular disease}) OR TITLE-ABS ({neuromuscular diseases}) OR TITLE-ABS ({neuromuscular disorder}) OR TITLE-ABS ({neuromuscular disorders}) OR TITLE-ABS ({neuromuscular dysfunction*}) OR TITLE-ABS ({Ocular Myasthenia Gravis}) OR TITLE-ABS ({Paralysis

Agitans}) OR TITLE-ABS ({Parkinson dementia complex}) OR TITLE-ABS ({Parkinsons disease}) OR TITLE-ABS ({Parkinson disease}) OR TITLE-ABS ({Parkinson's Disease*}) OR TITLE-ABS ({Presenile Dementia}) OR TITLE-ABS ({Primary Parkinsonism}) OR TITLE-ABS ({Primary Senile Degenerative Dementia}) OR TITLE-ABS ({sclerosis multiplex}) OR TITLE-ABS ({Seizure Disorder*}) OR TITLE-ABS ({Senile Dementia}) OR TITLE-ABS ({stroke}) OR TITLE-ABS ({Strokes}) OR TITLE-ABS ({Subcortical Arteriosclerotic Encephalopath*}) OR TITLE-ABS ({Subcortical Leukoencephalopathies}) OR TITLE-ABS ({Subcortical Leukoencephalopathy}) OR TITLE-ABS ({Tic Douloureux}) OR TITLE-ABS ({transient brain ischaemia}) OR TITLE-ABS ({transient brain ischemia}) OR TITLE-ABS ({Transient Brain Stem Ischemia*}) OR TITLE-ABS ({Transient Brainstem Ischemia*}) OR TITLE-ABS ({Transient Cerebral Ischemia*}) OR TITLE-ABS ({Transient Cerebral Ischaemia*}) OR TITLE-ABS ({transient ischaemic attack*}) OR TITLE-ABS ({transient ischaemic seizure*}) OR TITLE-ABS ({Transient Ischemic Attack*}) OR TITLE-ABS ({transient ischemic seizure*}) OR TITLE-ABS ({Trifacial Neuralgia*}) OR TITLE-ABS ({Trigeminal Neuralgia*}) OR TITLE-ABS ({Vascular Dementia*}) OR TITLE-ABS ({acute paranasal sinusitis}) OR TITLE-ABS ({airway disease}) OR TITLE-ABS ({airway diseases}) OR TITLE-ABS ({airway disorder*}) OR TITLE-ABS ({airway dysfunction*}) OR TITLE-ABS ({Asthma*}) OR TITLE-ABS ({Atrophic Rhinitides}) OR TITLE-ABS ({Atrophic Rhinitis}) OR TITLE-ABS ({Auditory Vertigo}) OR TITLE-ABS ({Auditory Vertigos}) OR TITLE-ABS ({Aural Vertigo}) OR TITLE-ABS ({benign paroxysmal postural vertigo}) OR TITLE-ABS ({benign postural paroxysmal vertigo}) OR TITLE-ABS ({bronchitis chronica}) OR TITLE-ABS ({cerebral vertigo}) OR TITLE-ABS ({Chronic Airflow Obstruction*}) OR TITLE-ABS ({chronic airway obstruction}) OR TITLE-ABS ({Chronic Bronchitis}) OR TITLE-ABS ({chronic bronchus infection}) OR TITLE-ABS ({chronic emphysema}) OR TITLE-ABS ({chronic obstructive bronchopulmonary disease}) OR TITLE-ABS ({Chronic Obstructive Lung Disease}) OR TITLE-ABS ({chronic obstructive lung disorder}) OR TITLE-ABS ({Chronic Obstructive Pulmonary Disease}) OR TITLE-ABS ({Chronic Obstructive Pulmonary Diseases}) OR TITLE-ABS ({chronic obstructive pulmonary disorder}) OR TITLE-ABS ({chronic pulmonary obstructive disease}) OR TITLE-ABS ({chronic pulmonary obstructive disorder}) OR TITLE-ABS ({COAD}) OR TITLE-ABS ({cochlea hydrops}) OR TITLE-ABS ({COPD}) OR TITLE-ABS ({Cystic Fibrosis of Pancreas}) OR TITLE-ABS ({diffuse parenchyma lung disease}) OR TITLE-ABS ({Diffuse Parenchymal Lung Disease}) OR TITLE-ABS ({Diffuse Parenchymal Lung Diseases}) OR TITLE-ABS ({diffuse parenchymal pulmonary disease}) OR TITLE-ABS ({diffuse parenchymal pulmonary disorder}) OR TITLE-ABS ({endolymphatic hydrops}) OR TITLE-ABS ({endolymphatic sac hydrops}) OR TITLE-ABS ({Ethmoid Sinusitides}) OR TITLE-ABS ({Ethmoid Sinusitis}) OR TITLE-ABS ({Ethmoidal Sinusitides}) OR TITLE-ABS ({Ethmoidal Sinusitis}) OR TITLE-ABS ({fibrocystic disease}) OR TITLE-ABS ({hydrops labyrinthi}) OR TITLE-ABS ({Hypersomnia with Periodic Respiration}) OR TITLE-ABS ({Interstitial Lung Disease}) OR TITLE-ABS ({Interstitial Lung Diseases}) OR TITLE-ABS ({interstitial lung disorder}) OR TITLE-ABS ({Interstitial Pneumonia}) OR TITLE-ABS ({Interstitial Pneumonias}) OR TITLE-ABS ({Interstitial Pneumonitides}) OR TITLE-ABS ({Interstitial Pneumonitis}) OR TITLE-ABS ({interstitial pneumopathy}) OR TITLE-ABS ({interstitial pulmonary disease}) OR TITLE-ABS ({interstitial pulmonary disorder}) OR TITLE-ABS ({labyrinth hydrops}) OR TITLE-ABS ({labyrinthal syndrome}) OR TITLE-ABS ({lung allergy}) OR TITLE-ABS ({lung chronic obstructive disease}) OR TITLE-ABS ({Meniere Disease}) OR TITLE-ABS ({Ménière Disease}) OR TITLE-ABS ({Ménière Diseases}) OR TITLE-ABS ({Meniere Syndrome}) OR TITLE-ABS ({Ménière Vertigo}) OR TITLE-ABS ({Menieres Disease}) OR TITLE-ABS ({Meniere's Disease}) OR TITLE-ABS ({Ménières Disease}) OR TITLE-ABS ({Ménière's Disease}) OR TITLE-ABS ({Ménière's Diseases}) OR TITLE-ABS ({Menieres Syndrome}) OR TITLE-ABS ({Meniere's Syndrome}) OR TITLE-ABS ({Ménières Vertigo}) OR TITLE-ABS ({Ménière's Vertigo}) OR TITLE-ABS ({Ménière's Vertigos}) OR TITLE-ABS ({Mucoviscidosis}) OR TITLE-ABS ({mucoviscoidosis}) OR TITLE-ABS ({nasal sinusitis}) OR TITLE-ABS ({nocturnal apnea}) OR TITLE-ABS ({nocturnal apnoea}) OR TITLE-ABS ({obstructive chronic lung disease}) OR TITLE-ABS ({obstructive chronic pulmonary disease}) OR TITLE-ABS ({Otogenic Vertigo}

) OR TITLE-ABS ({Otogenic Vertigos}) OR TITLE-ABS ({Otoscleroses}) OR TITLE-ABS ({otosclerosis surgery}) OR TITLE-ABS ({otosclerotic stapes}) OR TITLE-ABS ({otosphongiosis}) OR TITLE-ABS ({Otospongioses}) OR TITLE-ABS ({Otospongiosis}) OR TITLE-ABS ({Ozena}) OR TITLE-ABS ({Ozenas}) OR TITLE-ABS ({pancreas cystic disease}) OR TITLE-ABS ({pancreas cystic fibrosis}) OR TITLE-ABS ({Pancreas Fibrocystic Diseases}) OR TITLE-ABS ({pancreas fibrosis}) OR TITLE-ABS ({pancreatic cystic disease}) OR TITLE-ABS ({Pancreatic Cystic Fibrosis}) OR TITLE-ABS ({pancreatic fibrosis}) OR TITLE-ABS ({paroxysmal labyrinthine vertigo}) OR TITLE-ABS ({paroxysmal positional vertigo}) OR TITLE-ABS ({pneumatosis}) OR TITLE-ABS ({positional paroxysmal vertigo}) OR TITLE-ABS ({Pulmonary Cystic Fibrosis}) OR TITLE-ABS ({respiration disease}) OR TITLE-ABS ({respiration diseases}) OR TITLE-ABS ({respiration disorder}) OR TITLE-ABS ({respiration disorders}) OR TITLE-ABS ({respiration dysfunction*}) OR TITLE-ABS ({respiration tract disease}) OR TITLE-ABS ({respiration tract diseases}) OR TITLE-ABS ({respiration tract disorder}) OR TITLE-ABS ({respiration tract disorders}) OR TITLE-ABS ({respiration tract dysfunction*}) OR TITLE-ABS ({respiratory disease}) OR TITLE-ABS ({respiratory diseases}) OR TITLE-ABS ({respiratory disorder}) OR TITLE-ABS ({respiratory disorders}) OR TITLE-ABS ({respiratory illness*}) OR TITLE-ABS ({respiratory tract disease}) OR TITLE-ABS ({respiratory tract diseases}) OR TITLE-ABS ({respiratory tract disorder}) OR TITLE-ABS ({respiratory tract disorders}) OR TITLE-ABS ({respiratory tract dysfunction}) OR TITLE-ABS ({respiratory tract dysfunctioning}) OR TITLE-ABS ({respiratory tract dysfunctions}) OR TITLE-ABS ({rhinitis atrophica}) OR TITLE-ABS ({sinusitis nasalis}) OR TITLE-ABS ({Sleep Apnea}) OR TITLE-ABS ({Sleep Apneas}) OR TITLE-ABS ({sleep apnoea}) OR TITLE-ABS ({Sleep Disordered Breathing}) OR TITLE-ABS ({Sleep Hypopnea}) OR TITLE-ABS ({Sleep Hypopneas}) OR TITLE-ABS ({Sleep-Disordered Breathing}) OR TITLE-ABS ({vertiginous disease}) OR TITLE-ABS ({vertiginous disorder}) OR TITLE-ABS ({vertiginous syndrome}) OR TITLE-ABS ({vestibular vertigo}) OR TITLE-ABS ({Age Related Osteoporosis}) OR TITLE-ABS ({Age-Related Bone Loss}) OR TITLE-ABS ({Age-Related Bone Losses}) OR TITLE-ABS ({Age-Related Osteoporosis}) OR TITLE-ABS ({Akureyri disease}) OR TITLE-ABS ({Algodystrophic Syndrome}) OR TITLE-ABS ({Algodystrophies}) OR TITLE-ABS ({Algodystrophy}) OR TITLE-ABS ({alibert bazin disease}) OR TITLE-ABS ({arthropathic psoriasis}) OR TITLE-ABS ({Arthroses}) OR TITLE-ABS ({Arthrosis}) OR TITLE-ABS ({axial spondylarthritis}) OR TITLE-ABS ({Axial Spondyloarthritides}) OR TITLE-ABS ({Axial Spondyloarthritis}) OR TITLE-ABS ({AxSpA}) OR TITLE-ABS ({Back Ache}) OR TITLE-ABS ({Back Aches}) OR TITLE-ABS ({Back Pain*}) OR TITLE-ABS ({Backache}) OR TITLE-ABS ({Backaches}) OR TITLE-ABS ({backpain}) OR TITLE-ABS ({beauvais disease}) OR TITLE-ABS ({Bilateral Sciatica}) OR TITLE-ABS ({Bilateral Sciaticas}) OR TITLE-ABS ({Bone Paget Disease}) OR TITLE-ABS ({Bone Pagets Disease}) OR TITLE-ABS ({Calcium Pyrophosphate Deposition Disease}) OR TITLE-ABS ({Calcium Pyrophosphate Dihydrate Deposition}) OR TITLE-ABS ({Cervical Pain}) OR TITLE-ABS ({Cervical Pains}) OR TITLE-ABS ({Cervical Sympathetic Dystrophies}) OR TITLE-ABS ({Cervical Sympathetic Dystrophy}) OR TITLE-ABS ({Cervicalgia}) OR TITLE-ABS ({Cervicalgias}) OR TITLE-ABS ({Cervicodynia}) OR TITLE-ABS ({Cervicodynias}) OR TITLE-ABS ({chariot disease}) OR TITLE-ABS ({Chondrocalcinoses}) OR TITLE-ABS ({chronic articular rheumatism}) OR TITLE-ABS ({chronic fatigue}) OR TITLE-ABS ({Chronic Fatigue-Fibromyalgia Syndrome*}) OR TITLE-ABS ({Chronic Infectious Mononucleosis Like Syndrome}) OR TITLE-ABS ({Chronic Infectious Mononucleosis-Like Syndrome}) OR TITLE-ABS ({chronic rheumatism}) OR TITLE-ABS ({complex regional pain syndrome 1}) OR TITLE-ABS ({complex regional pain syndrome type 1}) OR TITLE-ABS ({CRPS 1}) OR TITLE-ABS ({CRPS I}) OR TITLE-ABS ({CRPS type 1}) OR TITLE-ABS ({CRPS Type I}) OR TITLE-ABS ({CRPS-I}) OR TITLE-ABS ({crystal arthropathies}) OR TITLE-ABS ({crystalline arthropathy}) OR TITLE-ABS ({degenerative joint disease}) OR TITLE-ABS ({Diffuse Myofascial Pain Syndrome}) OR TITLE-ABS ({disc hernia}) OR TITLE-ABS ({Disc Herniation}) OR TITLE-ABS ({Disc Herniations}) OR TITLE-ABS ({disc prolapse}) OR TITLE-ABS ({Disc Protrusion}) OR TITLE-ABS ({Disc Protrusions}) OR TITLE-ABS ({discal hernia}) OR TITLE-ABS ({discal herniation}) OR

TITLE-ABS ({discus hernia}) OR TITLE-ABS ({disk hernia}) OR TITLE-ABS ({Disk Herniation}) OR TITLE-ABS ({Disk Herniations}) OR TITLE-ABS ({Disk Prolapse}) OR TITLE-ABS ({Disk Prolapses}) OR TITLE-ABS ({Disk Protrusion}) OR TITLE-ABS ({Disk Protrusions}) OR TITLE-ABS ({dorsalgia}) OR TITLE-ABS ({epicondylalgia}) OR TITLE-ABS ({epidemic neuromyasthenia}) OR TITLE-ABS ({fatigue syndrome}) OR TITLE-ABS ({fibro myalgia}) OR TITLE-ABS ({Fibromyalgia Fibromyositis Syndrome}) OR TITLE-ABS ({Fibromyalgia-Fibromyositis Syndrome}) OR TITLE-ABS ({Fibromyalgia-Fibromyositis Syndromes}) OR TITLE-ABS ({Fibromyalgias}) OR TITLE-ABS ({Fibromyositis Fibromyalgia Syndrome}) OR TITLE-ABS ({Fibromyositis-Fibromyalgia Syndrome}) OR TITLE-ABS ({Fibromyositis-Fibromyalgia Syndromes}) OR TITLE-ABS ({fibrositic nodule}) OR TITLE-ABS ({Fibrositides}) OR TITLE-ABS ({Fibrositis}) OR TITLE-ABS ({hernia disci}) OR TITLE-ABS ({hernia nuclei pulposi}) OR TITLE-ABS ({Herniated Disc}) OR TITLE-ABS ({Herniated Discs}) OR TITLE-ABS ({Herniated Disk}) OR TITLE-ABS ({Herniated Disks}) OR TITLE-ABS ({herniated intervertebral disc}) OR TITLE-ABS ({herniated intervertebral disk}) OR TITLE-ABS ({herniated nucleus pulposus}) OR TITLE-ABS ({herniated vertebral disc}) OR TITLE-ABS ({herniated vertebral disk}) OR TITLE-ABS ({hypertrophic infiltrative tendinitis}) OR TITLE-ABS ({Iceland disease}) OR TITLE-ABS ({Intervertebral Disc Displacement}) OR TITLE-ABS ({Intervertebral Disc Displacements}) OR TITLE-ABS ({Intervertebral Disk Displacement}) OR TITLE-ABS ({Intervertebral Disk Displacements}) OR TITLE-ABS ({intervertebral disk perforation}) OR TITLE-ABS ({intervertebral disk rupture}) OR TITLE-ABS ({intervertebral prolapse}) OR TITLE-ABS ({Involutional Osteoporosis}) OR TITLE-ABS ({ischias}) OR TITLE-ABS ({ischiatic pain}) OR TITLE-ABS ({Lateral Epicondylitides}) OR TITLE-ABS ({Lateral Epicondylitis}) OR TITLE-ABS ({Lateral Humeral Epicondylitides}) OR TITLE-ABS ({Lateral Humeral Epicondylitis}) OR TITLE-ABS ({loin pain}) OR TITLE-ABS ({lowback pain}) OR TITLE-ABS ({Lumbago}) OR TITLE-ABS ({lumbal pain}) OR TITLE-ABS ({lumbal syndrome}) OR TITLE-ABS ({lumbalgesia}) OR TITLE-ABS ({lumbalgia}) OR TITLE-ABS ({lumbar pain}) OR TITLE-ABS ({lumbar spine syndrome}) OR TITLE-ABS ({lumbodynia}) OR TITLE-ABS ({lumbosacral pain}) OR TITLE-ABS ({lumbosacral root syndrome}) OR TITLE-ABS ({lumbosacroiliac strain}) OR TITLE-ABS ({Muscular Rheumatism}) OR TITLE-ABS ({Myalgic Encephalomyelitis}) OR TITLE-ABS ({Neck Ache}) OR TITLE-ABS ({Neck Aches}) OR TITLE-ABS ({Neck Pain*}) OR TITLE-ABS ({Neckache}) OR TITLE-ABS ({Neckaches}) OR TITLE-ABS ({neuralgic shoulder amyotrophy}) OR TITLE-ABS ({nodular tendinitis}) OR TITLE-ABS ({nucleus pulposus hernia}) OR TITLE-ABS ({Osseous Paget's Disease}) OR TITLE-ABS ({osteitis deformans}) OR TITLE-ABS ({Osteoarthritides}) OR TITLE-ABS ({osteo-arthritis}) OR TITLE-ABS ({Osteoarthroses}) OR TITLE-ABS ({Osteoarthrosis}) OR TITLE-ABS ({osteo-arthrosis}) OR TITLE-ABS ({Osteoporoses}) OR TITLE-ABS ({ostitis deformans}) OR TITLE-ABS ({Paget Disease of Bone}) OR TITLE-ABS ({Paget disease of the bone}) OR TITLE-ABS ({Pagets bone disease}) OR TITLE-ABS ({Paget's bone disease}) OR TITLE-ABS ({Pagets disease of bone}) OR TITLE-ABS ({Paget's Disease of Bone}) OR TITLE-ABS ({Paget's disease of the bone}) OR TITLE-ABS ({paralytic scoliosis}) OR TITLE-ABS ({Post Traumatic Osteoporosis}) OR TITLE-ABS ({posttraumatic dystrophy}) OR TITLE-ABS ({post-traumatic dystrophy}) OR TITLE-ABS ({Post-Traumatic Osteoporosis}) OR TITLE-ABS ({Postviral Fatigue Syndromes}) OR TITLE-ABS ({Primary Fibromyalgia}) OR TITLE-ABS ({primary osteoarthritis}) OR TITLE-ABS ({progressive scoliosis}) OR TITLE-ABS ({Prolapsed Disc}) OR TITLE-ABS ({Prolapsed Discs}) OR TITLE-ABS ({Prolapsed Disk}) OR TITLE-ABS ({Prolapsed Disks}) OR TITLE-ABS ({Protruded Disc}) OR TITLE-ABS ({Protruded Discs}) OR TITLE-ABS ({Protruded Disk}) OR TITLE-ABS ({Protruded Disks}) OR TITLE-ABS ({Pseudogout}) OR TITLE-ABS ({Psoriasis Arthropathica}) OR TITLE-ABS ({psoriasis pustulosa arthropathica}) OR TITLE-ABS ({Psoriatic Arthropathies}) OR TITLE-ABS ({Psoriatic Arthropathy}) OR TITLE-ABS ({psoriatic rheumatism}) OR TITLE-ABS ({Reflex Sympathetic Dystrophies}) OR TITLE-ABS ({Reflex Sympathetic Dystrophy}) OR TITLE-ABS ({rheumarthritis}) OR TITLE-ABS ({rheumatic disease}) OR TITLE-ABS ({rheumatic diseases}) OR TITLE-ABS ({rheumatoid disease}) OR TITLE-ABS ({rheumatoid diseases}) OR TITLE-ABS ({rheumatoid inflammation}) OR TITLE-ABS ({rheumatological

disease}) OR TITLE-ABS ({rheumatological diseases}) OR TITLE-ABS ({rheumatological disorder}) OR TITLE-ABS ({rheumatological disorders}) OR TITLE-ABS ({Royal Free Disease}) OR TITLE-ABS ({Sciatic Neuralgia}) OR TITLE-ABS ({Sciatic Neuralgias}) OR TITLE-ABS ({sciatic pain}) OR TITLE-ABS ({Scolioses}) OR TITLE-ABS ({Secondary Fibromyalgia}) OR TITLE-ABS ({Senile Osteoporosis}) OR TITLE-ABS ({shoulder arm syndrome}) OR TITLE-ABS ({Shoulder Hand Syndrome}) OR TITLE-ABS ({Shoulder-Hand Syndrome}) OR TITLE-ABS ({Shoulder-Hand Syndromes}) OR TITLE-ABS ({Slipped Disc}) OR TITLE-ABS ({Slipped Discs}) OR TITLE-ABS ({Slipped Disk}) OR TITLE-ABS ({Slipped Disks}) OR TITLE-ABS ({slipped intervertebral disc}) OR TITLE-ABS ({slipped vertebral disc}) OR TITLE-ABS ({spinal disk disease}) OR TITLE-ABS ({Sudek Atrophy}) OR TITLE-ABS ({Sudek's Atrophies}) OR TITLE-ABS ({Sudeks Atrophy}) OR TITLE-ABS ({Sudek's Atrophy}) OR TITLE-ABS ({sympathetic dystrophy syndrome}) OR TITLE-ABS ({Sympathetic Reflex Dystrophia}) OR TITLE-ABS ({Sympathetic Reflex Dystrophias}) OR TITLE-ABS ({sympathetic reflex dystrophy}) OR TITLE-ABS ({Systemic Exertion Intolerance Disease}) OR TITLE-ABS ({tendinopathy}) OR TITLE-ABS ({tendinosis}) OR TITLE-ABS ({tendonitis}) OR TITLE-ABS ({tendonopathy}) OR TITLE-ABS ({Tennis Elbow}) OR TITLE-ABS ({Tennis Elbows}) OR TITLE-ABS ({tenonitis}) OR TITLE-ABS ({tenontitis}) OR TITLE-ABS ({tenositis}) OR TITLE-ABS ({Type I Complex Regional Pain Syndrome}) OR TITLE-ABS ({Vertebrogenic Pain Syndrome}) OR TITLE-ABS ({Vertebrogenic Pain Syndromes}) OR TITLE-ABS ({Yuppie flu}) OR TITLE-ABS ({Acne Inversa*}) OR TITLE-ABS ({acne juvenilis}) OR TITLE-ABS ({Acne Rosacea}) OR TITLE-ABS ({Chronic Bullous Disease of Childhood}) OR TITLE-ABS ({cutaneous disease}) OR TITLE-ABS ({cutaneous diseases}) OR TITLE-ABS ({cutaneus disorder}) OR TITLE-ABS ({cutaneus disorders}) OR TITLE-ABS ({dermal disease}) OR TITLE-ABS ({dermal diseases}) OR TITLE-ABS ({dermal disorder}) OR TITLE-ABS ({dermal disorders}) OR TITLE-ABS ({Drug induced Linear IgA Bullous Dermatos*}) OR TITLE-ABS ({Drug-induced Linear IgA Bullous Dermatos*}) OR TITLE-ABS ({Eczema*}) OR TITLE-ABS ({Erythematotelangiectatic Rosacea}) OR TITLE-ABS ({Granulomatous Rosacea}) OR TITLE-ABS ({herpes}) OR TITLE-ABS ({hidradenitis suppurativa}) OR TITLE-ABS ({Hives}) OR TITLE-ABS ({juvenile acne}) OR TITLE-ABS ({Linear IgA Dermatos*}) OR TITLE-ABS ({Linear IgA IgG Bullous Dermatos*}) OR TITLE-ABS ({Linear IgA IgG Dermatos*}) OR TITLE-ABS ({Ocular Rosacea}) OR TITLE-ABS ({Palmoplantaris Pustulosis}) OR TITLE-ABS ({Papulopustular Rosacea}) OR TITLE-ABS ({Phymatous Rosacea}) OR TITLE-ABS ({Psoriases}) OR TITLE-ABS ({psoriasiform dermatitis}) OR TITLE-ABS ({psoriasiform dermatosis}) OR TITLE-ABS ({psoriasiform lesion}) OR TITLE-ABS ({psoriasiform rash}) OR TITLE-ABS ({psoriasiform skin rash}) OR TITLE-ABS ({psoriatic epidermis}) OR TITLE-ABS ({psoriatic skin}) OR TITLE-ABS ({Pustular Psoriasis of Palms and Soles}) OR TITLE-ABS ({Pustulosis of Palms and Soles}) OR TITLE-ABS ({Pustulosis Palmaris et Plantaris}) OR TITLE-ABS ({rhinophyma}) OR TITLE-ABS ({rozacea}) OR TITLE-ABS ({skin and connective tissue disease}) OR TITLE-ABS ({skin and connective tissue diseases}) OR TITLE-ABS ({skin and connective tissue disorder}) OR TITLE-ABS ({skin and connective tissue disorders}) OR TITLE-ABS ({skin disease}) OR TITLE-ABS ({skin diseases}) OR TITLE-ABS ({skin disorder}) OR TITLE-ABS ({skin disorders}) OR TITLE-ABS ({Suppurative Hidradeniti*}) OR TITLE-ABS ({urticary}) OR TITLE-ABS ({weal}) OR TITLE-ABS ({wheal}) OR TITLE-ABS ({whealing}) OR TITLE-ABS ({willan lepra}) OR TITLE-ABS ({active TB}) OR TITLE-ABS ({Adamantiades-Behcet Disease*}) OR TITLE-ABS ({Allergic Angiit*}) OR TITLE-ABS ({Allergic Granulomatos*}) OR TITLE-ABS ({Allergic Granulomatous and Angiitis}) OR TITLE-ABS ({Allergic Granulomatous Angiit*}) OR TITLE-ABS ({allergic granulomatous angitis}) OR TITLE-ABS ({anonymous artery occlusion}) OR TITLE-ABS ({Anti Phospholipid Antibody Syndrome*}) OR TITLE-ABS ({Anti Phospholipid Syndrome*}) OR TITLE-ABS ({Antiphospholipid Antibody Syndrome*}) OR TITLE-ABS ({Anti-Phospholipid Antibody Syndrome*}) OR TITLE-ABS ({antiphospholipid syndrome}) OR TITLE-ABS ({Anti-Phospholipid Syndrome*}) OR TITLE-ABS ({aorta arch syndrome}) OR TITLE-ABS ({aortic arch syndromes}) OR TITLE-ABS ({Aortitis Syndrome*}) OR TITLE-ABS ({APLA syndrome}) OR TITLE-ABS ({arteritis brachiocephalica}) OR TITLE-ABS ({arteritis nodosa}) OR TITLE-ABS (

{autoimmune disease}) OR TITLE-ABS ({autoimmune diseases}) OR TITLE-ABS ({Behcet Disease*}) OR TITLE-ABS ({Behçet Disease*}) OR TITLE-ABS ({Behcet syndrome}) OR TITLE-ABS ({behcet ulcer}) OR TITLE-ABS ({Behcets disease}) OR TITLE-ABS ({Behcet's Disease*}) OR TITLE-ABS ({Behcets syndrome}) OR TITLE-ABS ({Behcet's Syndrome*}) OR TITLE-ABS ({besnier boeck syndrome}) OR TITLE-ABS ({Besnier-Boeck Disease*}) OR TITLE-ABS ({Besnier-Boeck-Schaumann Syndrome*}) OR TITLE-ABS ({Boeck Disease*}) OR TITLE-ABS ({Boecks Disease*}) OR TITLE-ABS ({Boeck's Disease*}) OR TITLE-ABS ({brachiocephalic arteritis}) OR TITLE-ABS ({brachiocephalic artery occlusion}) OR TITLE-ABS ({brachiocephalic ischaemia}) OR TITLE-ABS ({brachiocephalic ischemia}) OR TITLE-ABS ({brachiocephalic trunk occlusion}) OR TITLE-ABS ({brachiocephalic vascular occlusion}) OR TITLE-ABS ({Church Strauss syndrome}) OR TITLE-ABS ({Churg Strauss}) OR TITLE-ABS ({Churg-Strauss Syndrome}) OR TITLE-ABS ({Cranial Arterit*}) OR TITLE-ABS ({cryoglobulinaemia}) OR TITLE-ABS ({Cryoglobulinemias}) OR TITLE-ABS ({cryoimmunoglobulinaemia}) OR TITLE-ABS ({cryoimmunoglobulinemia}) OR TITLE-ABS ({Cutis Elastica}) OR TITLE-ABS ({dacryosialoadenopathia atrophicans}) OR TITLE-ABS ({EDS IV}) OR TITLE-ABS ({Ehlers Danlos}) OR TITLE-ABS ({Ehlers-Danlos Disease*}) OR TITLE-ABS ({Ehlers-Danlos syndrome}) OR TITLE-ABS ({eosinophilic GPA}) OR TITLE-ABS ({eosinophilic granulomatosis polyangiitis}) OR TITLE-ABS ({eosinophilic granulomatosis polyangitis}) OR TITLE-ABS ({eosinophilic granulomatous angiitis}) OR TITLE-ABS ({Eosinophilic Granulomatous Vasculit*}) OR TITLE-ABS ({erythematodes visceralis}) OR TITLE-ABS ({Essential Polyarterit*}) OR TITLE-ABS ({generalised scleroderma}) OR TITLE-ABS ({generalized scleroderma}) OR TITLE-ABS ({Giant Cell Aortic Arteritis}) OR TITLE-ABS ({Giant Cell Aortiti*}) OR TITLE-ABS ({giant cell arteriitis}) OR TITLE-ABS ({Giant Cell Arteriti*}) OR TITLE-ABS ({gougerot houwer sjoegren syndrome}) OR TITLE-ABS ({gougerot mulock houwer sjoegren syndrome}) OR TITLE-ABS ({Gougerot Sjoegren syndrome}) OR TITLE-ABS ({Gougerot-Sjogren syndrome}) OR TITLE-ABS ({granulomatosis and polyangiitis}) OR TITLE-ABS ({granulomatosis and polyangitis}) OR TITLE-ABS ({Granulomatosis with Polyangiit*}) OR TITLE-ABS ({granulomatosis with polyangitis}) OR TITLE-ABS ({granulomatous allergic angitis}) OR TITLE-ABS ({granulomatous polyangiitis}) OR TITLE-ABS ({granulomatous polyangitis}) OR TITLE-ABS ({Horton arteritis}) OR TITLE-ABS ({Horton Disease}) OR TITLE-ABS ({Horton's arteritis}) OR TITLE-ABS ({Hortons Disease}) OR TITLE-ABS ({Horton's Disease}) OR TITLE-ABS ({Hughes Syndrome*}) OR TITLE-ABS ({innominate arterial ligation}) OR TITLE-ABS ({innominate artery ligation}) OR TITLE-ABS ({innominate artery occlusion}) OR TITLE-ABS ({jungling syndrome}) OR TITLE-ABS ({kussmaul maier disease}) OR TITLE-ABS ({kussmaul syndrome}) OR TITLE-ABS ({Libman Sacks Disease*}) OR TITLE-ABS ({Libman-Sacks Disease*}) OR TITLE-ABS ({lupovisceritis}) OR TITLE-ABS ({lymphogranuloma benignum}) OR TITLE-ABS ({malignant dermatovisceritism}) OR TITLE-ABS ({martorell syndrome}) OR TITLE-ABS ({Microscopic Polyangiitides}) OR TITLE-ABS ({microscopic polyarteritis}) OR TITLE-ABS ({mikulicz radecki syndrome}) OR TITLE-ABS ({mixed cryoglobulinemia}) OR TITLE-ABS ({morbus Wegener}) OR TITLE-ABS ({mucoserous dyssecretosis}) OR TITLE-ABS ({mukilicz radecki syndrome}) OR TITLE-ABS ({multisystem disease}) OR TITLE-ABS ({multisystem diseases}) OR TITLE-ABS ({multisystem disorder}) OR TITLE-ABS ({multisystem disorders}) OR TITLE-ABS ({multisystem dysfunction*}) OR TITLE-ABS ({necrotising respiratory granulomatosis}) OR TITLE-ABS ({Necrotizing Arterit*}) OR TITLE-ABS ({necrotizing respiratory granulomatosis}) OR TITLE-ABS ({nodular periarteritis}) OR TITLE-ABS ({nodular polyarteritis}) OR TITLE-ABS ({oculobuccopharyngeal dryness}) OR TITLE-ABS ({Old Silk Route Disease*}) OR TITLE-ABS ({panarteriitis nodosa}) OR TITLE-ABS ({panarteritis nodosa}) OR TITLE-ABS ({periarterial fibrosis}) OR TITLE-ABS ({periarteriitis nodosa}) OR TITLE-ABS ({Periarteritis Nodosa}) OR TITLE-ABS ({pneumogenic granulomatosis}) OR TITLE-ABS ({poliarteritis nodosa}) OR TITLE-ABS ({polyarteriitis nodosa}) OR TITLE-ABS ({progressive scleroderma}) OR TITLE-ABS ({progressive sclerodermia}) OR TITLE-ABS ({Pulseless Disease*}) OR TITLE-ABS ({reversed coarctation}) OR TITLE-ABS ({rheumatic sialosis}) OR TITLE-ABS ({sarcoid}) OR TITLE-ABS (

{sarcoidoses}) OR TITLE-ABS ({Schaumann Disease*}) OR TITLE-ABS ({Schaumann Syndrome*}) OR TITLE-ABS ({Schaumann's Syndrome*}) OR TITLE-ABS ({sicca syndrome}) OR TITLE-ABS ({sjoegren disease}) OR TITLE-ABS ({sjogren disease}) OR TITLE-ABS ({sjogren syndrome}) OR TITLE-ABS ({sjogren's syndrome}) OR TITLE-ABS ({lupus}) OR TITLE-ABS ({Strauss Churg syndrome}) OR TITLE-ABS ({systemic disease}) OR TITLE-ABS ({systemic diseases}) OR TITLE-ABS ({systemic disorder*}) OR TITLE-ABS ({systemic dysfunction*}) OR TITLE-ABS ({systemic progressive sclerosis}) OR TITLE-ABS ({Systemic Scleroderma}) OR TITLE-ABS ({Systemic Sclerosis}) OR TITLE-ABS ({takayasu arteriopathy}) OR TITLE-ABS ({Takayasu Arteritis}) OR TITLE-ABS ({Takayasu Disease*}) OR TITLE-ABS ({takayasu ohnishi syndrome}) OR TITLE-ABS ({Takayasu Syndrome*}) OR TITLE-ABS ({Takayasus Arteritis}) OR TITLE-ABS ({Takayasu's Arteritis}) OR TITLE-ABS ({Temporal Arterit*}) OR TITLE-ABS ({Triple Symptom Complex}) OR TITLE-ABS ({Triple Symptom Complices}) OR TITLE-ABS ({Triple-Symptom Complex}) OR TITLE-ABS ({Wegener disease}) OR TITLE-ABS ({Wegener granuloma}) OR TITLE-ABS ({Wegener Granulomatosis}) OR TITLE-ABS ({Wegener Klinger Churg syndrome}) OR TITLE-ABS ({Wegener Klinger granulomatosis}) OR TITLE-ABS ({Wegener syndrome}) OR TITLE-ABS ({Wegener's disease}) OR TITLE-ABS ({Wegener's GPA}) OR TITLE-ABS ({Wegener's granuloma}) OR TITLE-ABS ({Wegener's Granulomatosis}) OR TITLE-ABS ({Wegner granulomatosis}) OR TITLE-ABS ({Young Female Arterit*}) OR TITLE-ABS ({Anti GBM Disease*}) OR TITLE-ABS ({Anti Glomerular Basement Membrane Disease}) OR TITLE-ABS ({Anti-GBM Disease*}) OR TITLE-ABS ({bladder incontinence}) OR TITLE-ABS ({chronic disease kidney function}) OR TITLE-ABS ({Chronic Kidney Failure*}) OR TITLE-ABS ({chronic nephropathy}) OR TITLE-ABS ({Chronic Renal Failure*}) OR TITLE-ABS ({cystic kidney}) OR TITLE-ABS ({End Stage Kidney Disease*}) OR TITLE-ABS ({End Stage Renal Disease*}) OR TITLE-ABS ({End Stage Renal Failure*}) OR TITLE-ABS ({End-Stage Kidney Disease*}) OR TITLE-ABS ({End-Stage Renal Disease*}) OR TITLE-ABS ({End-Stage Renal Failure*}) OR TITLE-ABS ({ESRD}) OR TITLE-ABS ({familial nephrolithiasis}) OR TITLE-ABS ({goodpasture disease}) OR TITLE-ABS ({goodpasture renopulmonary syndrome}) OR TITLE-ABS ({Goodpasture Syndrome*}) OR TITLE-ABS ({Goodpastures Syndrome*}) OR TITLE-ABS ({Goodpasture's Syndrome*}) OR TITLE-ABS ({incontinentia urinae}) OR TITLE-ABS ({involuntary urinary loss}) OR TITLE-ABS ({involuntary urination}) OR TITLE-ABS ({involuntary urine loss}) OR TITLE-ABS ({Kidney Calcul*}) OR TITLE-ABS ({kidney calix stone}) OR TITLE-ABS ({kidney calyx stone}) OR TITLE-ABS ({kidney chronic failure}) OR TITLE-ABS ({kidney cystic disease}) OR TITLE-ABS ({kidney disease}) OR TITLE-ABS ({kidney diseases}) OR TITLE-ABS ({kidney disorder}) OR TITLE-ABS ({kidney disorders}) OR TITLE-ABS ({kidney failure}) OR TITLE-ABS ({kidney failures}) OR TITLE-ABS ({kidney insufficien*}) OR TITLE-ABS ({kidney lithiasis}) OR TITLE-ABS ({kidney multicystic aplasia}) OR TITLE-ABS ({kidney multicystic disease}) OR TITLE-ABS ({kidney pelvis stone}) OR TITLE-ABS ({kidney polycystosis}) OR TITLE-ABS ({Kidney Stone*}) OR TITLE-ABS ({leakage of urine}) OR TITLE-ABS ({Lung Purpura with Nephritis}) OR TITLE-ABS ({male genital disorder}) OR TITLE-ABS ({male genital disorders}) OR TITLE-ABS ({male infertility}) OR TITLE-ABS ({Nephrolith}) OR TITLE-ABS ({pneumorenal syndrome}) OR TITLE-ABS ({Polycystic Kidney}) OR TITLE-ABS ({Polycystic Kidneys}) OR TITLE-ABS ({prostate adenoma}) OR TITLE-ABS ({Renal Calcul*}) OR TITLE-ABS ({renal cystic disease}) OR TITLE-ABS ({renal disease}) OR TITLE-ABS ({renal diseases}) OR TITLE-ABS ({renal disorder}) OR TITLE-ABS ({renal disorders}) OR TITLE-ABS ({renal failure}) OR TITLE-ABS ({renal failures}) OR TITLE-ABS ({renal insufficien*}) OR TITLE-ABS ({renal pelvis stone}) OR TITLE-ABS ({renal polycystic disease}) OR TITLE-ABS ({renal stone}) OR TITLE-ABS ({renolithiasis}) OR TITLE-ABS ({unwanted urine loss}) OR TITLE-ABS ({urinary incontinence}) OR TITLE-ABS ({urinary leakage}) OR TITLE-ABS ({urine incontinence}) OR TITLE-ABS ({urine leakage}) OR TITLE-ABS ({urine wetting}) OR TITLE-ABS ({urologic disease*}) OR TITLE-ABS ({urologic disorder*}) OR TITLE-ABS ({urological disease*}) OR TITLE-ABS ({urological disorder*}) OR TITLE-ABS ({Age Related Macular Degeneration}) OR TITLE-ABS ({Age-Related Macular Degeneration*}) OR TITLE-ABS ({atrophia maculae luteae}) OR TITLE-

ABS ({bilateral macular degeneration}) OR TITLE-ABS ({Cataract*}) OR TITLE-ABS ({chronic uveitis}) OR TITLE-ABS ({corpus vitreum detachment}) OR TITLE-ABS ({Day Blindness}) OR TITLE-ABS ({degeneratio maculae luteae retinae}) OR TITLE-ABS ({detachment corporis vitrei}) OR TITLE-ABS ({detachment vitreous}) OR TITLE-ABS ({disciform macular degeneration}) OR TITLE-ABS ({Episclerit*}) OR TITLE-ABS ({eye disease}) OR TITLE-ABS ({eye diseases}) OR TITLE-ABS ({eye disorder}) OR TITLE-ABS ({eye disorders}) OR TITLE-ABS ({eye dysfunction}) OR TITLE-ABS ({eye dysfunctioning}) OR TITLE-ABS ({eye dysfunctions}) OR TITLE-ABS ({Hemeralopia*}) OR TITLE-ABS ({heredomacular degeneration}) OR TITLE-ABS ({immunogenic uveitis}) OR TITLE-ABS ({junius kuhnt disease}) OR TITLE-ABS ({lens clouding}) OR TITLE-ABS ({Lens Opacit*}) OR TITLE-ABS ({Macropsia*}) OR TITLE-ABS ({macula atrophy}) OR TITLE-ABS ({macula bilateral degeneration}) OR TITLE-ABS ({macula degeneration}) OR TITLE-ABS ({macula lutea atrophy}) OR TITLE-ABS ({macula lutea degeneration}) OR TITLE-ABS ({macula lutea disciform degeneration}) OR TITLE-ABS ({macula lutea retina atrophy}) OR TITLE-ABS ({macula lutea retina degeneration}) OR TITLE-ABS ({macula retina atrophy}) OR TITLE-ABS ({macula retina degeneration}) OR TITLE-ABS ({macular atrophy}) OR TITLE-ABS ({Macular Degenerations}) OR TITLE-ABS ({macular disciform degeneration}) OR TITLE-ABS ({Macular Dystroph*}) OR TITLE-ABS ({Maculopath*}) OR TITLE-ABS ({Metamorphopsia*}) OR TITLE-ABS ({Micropsia*}) OR TITLE-ABS ({Myopia*}) OR TITLE-ABS ({Nearsightedness*}) OR TITLE-ABS ({Necrotizing Sclerit*}) OR TITLE-ABS ({ocular disease}) OR TITLE-ABS ({ocular diseases}) OR TITLE-ABS ({ocular disorder}) OR TITLE-ABS ({ocular disorders}) OR TITLE-ABS ({ocular dysfunction*}) OR TITLE-ABS ({ophthalmic disease}) OR TITLE-ABS ({ophthalmic diseases}) OR TITLE-ABS ({ophthalmic disorder}) OR TITLE-ABS ({ophthalmic disorders}) OR TITLE-ABS ({ophthalmic dysfunction*}) OR TITLE-ABS ({ophthalmologic disease}) OR TITLE-ABS ({ophthalmologic diseases}) OR TITLE-ABS ({ophthalmologic disorder}) OR TITLE-ABS ({ophthalmologic disorders}) OR TITLE-ABS ({ophthalmologic dysfunction*}) OR TITLE-ABS ({panuveitis}) OR TITLE-ABS ({posterior capsule opacification}) OR TITLE-ABS ({posterior uveitis}) OR TITLE-ABS ({Presbyopias}) OR TITLE-ABS ({Pseudoaphakia*}) OR TITLE-ABS ({retina macula disciform degeneration}) OR TITLE-ABS ({retinal diseases}) OR TITLE-ABS ({Scleritides}) OR TITLE-ABS ({secondary scleritis}) OR TITLE-ABS ({suppurative uveitis}) OR TITLE-ABS ({Uveitides}) OR TITLE-ABS ({Vision Disabilit*}) OR TITLE-ABS ({vision disorder}) OR TITLE-ABS ({vision disorders}) OR TITLE-ABS ({vision disturbance}) OR TITLE-ABS ({visual disorder}) OR TITLE-ABS ({visual disorders}) OR TITLE-ABS ({visual disturbance}) OR TITLE-ABS ({Visual Impairment*}) OR TITLE-ABS ({vitreous detachment}) OR TITLE-ABS ({sarcoidosis}) OR TITLE-ABS ({neoplasia}) OR TITLE-ABS ({lymphoma*}) OR TITLE-ABS ({hypertension}) OR TITLE-ABS ({infarction*}) OR TITLE-ABS ({glaucom*}) OR TITLE-ABS ({cancer*}) OR TITLE-ABS ({carcinoma*}) OR TITLE-ABS ({neoplasm*}) OR TITLE-ABS ({tumor*}) OR TITLE-ABS ({tumour*}) OR TITLE-ABS ({Horton syndrome}) OR TITLE-ABS ({Horton's syndrome}) OR TITLE-ABS ({Gastro-esophageal reflux}) OR TITLE-ABS ({gastroesophageal reflux}) OR TITLE-ABS ({GORD})) AND (KEY ({dyspareunia}) OR KEY ({erectile dysfunction}) OR KEY ({libido}) OR KEY ({libido disorder}) OR KEY ({orgasm}) OR KEY ({orgasm disorder}) OR KEY ({psychosexual disorder}) OR KEY ({sexual arousal}) OR KEY ({sexual arousal disorder}) OR KEY ({sexual desire}) OR KEY ({sexual dysfunction}) OR KEY ({Sexual dysfunction, physiological}) OR KEY ({Sexual dysfunction, psychological}) OR KEY ({sexuality}) OR KEY ({vaginism}) OR KEY ({Vaginismus}) OR TITLE-ABS ({coitus}) OR TITLE-ABS ({desire}) OR TITLE-ABS ({dyspareunia}) OR TITLE-ABS ({ego-dystonic homosexuality}) OR TITLE-ABS ({ejaculatio praecox}) OR TITLE-ABS ({ejaculatio praecoxs}) OR TITLE-ABS ({ejaculatio precox}) OR TITLE-ABS ({erectile dysfunction*}) OR TITLE-ABS ({erection}) OR TITLE-ABS ({frigidities}) OR TITLE-ABS ({frigidity}) OR TITLE-ABS ({genital disorder}) OR TITLE-ABS ({genital disorders}) OR TITLE-ABS ({go-dystonic homosexuality}) OR TITLE-ABS ({libido}) OR TITLE-ABS ({orgasm}) OR TITLE-ABS ({orgasms}) OR TITLE-ABS ({premature ejaculation}) OR TITLE-ABS ({premature ejaculations}) OR TITLE-ABS (

{psychosexuality}) OR TITLE-ABS ({sex abnormalit*}) OR TITLE-ABS ({sex arousal}) OR TITLE-ABS ({sex disorder}) OR TITLE-ABS ({sex disorders}) OR TITLE-ABS ({sex drive}) OR TITLE-ABS ({sex dysfunction*}) OR TITLE-ABS ({sex insufficienc*}) OR TITLE-ABS ({sex problem}) OR TITLE-ABS ({sex problems}) OR TITLE-ABS ({vaginismus}) OR TITLE-ABS ({sexual}) OR TITLE-ABS ({psychosexual}) OR TITLE-ABS ({sexuality})) AND (KEY ({adult}) OR TITLE-ABS ({adult}) OR TITLE-ABS ({adults}) OR TITLE-ABS ({old people}) OR TITLE-ABS ({elderly}) OR TITLE-ABS ({frail}) OR TITLE-ABS ({frailness}) OR TITLE-ABS ({frailty}) OR TITLE-ABS ({old age}) OR TITLE-ABS ({old patients}) OR TITLE-ABS ({old person}) OR TITLE-ABS ({old persons}) OR TITLE-ABS ({older adult}) OR TITLE-ABS ({older adults}) OR TITLE-ABS ({older patient}) OR TITLE-ABS ({older patients}) OR TITLE-ABS ({older people}) OR TITLE-ABS ({older person}) OR TITLE-ABS ({older persons}) OR TITLE-ABS ({senior people}) OR TITLE-ABS ({senior person}) OR TITLE-ABS ({senior persons}) OR TITLE-ABS ({seniors})) AND (KEY ({systematic review}) OR KEY ({meta analysis}) OR KEY ({scoping review}) OR TITLE-ABS ({scoping review*}) OR TITLE-ABS ({systematic review*}) OR TITLE-ABS ({systematic literature review*}) OR TITLE-ABS ({systematic narrative review*}) OR TITLE-ABS ({systematic qualitative review*}) OR TITLE-ABS ({systematic evidence review*}) OR TITLE-ABS ({systematic quantitative review*}) OR TITLE-ABS ({systematic meta-review*}) OR TITLE-ABS ({systematic critical review*}) OR TITLE-ABS ({systematic mapping review*}) OR TITLE-ABS ({systematic cochrane review*}) OR TITLE-ABS ({systematic electronic literature search*}) OR TITLE-ABS ({PRISMA}) OR TITLE-ABS ({systematic descriptive review*}) OR TITLE-ABS ({systematic analys*}) OR TITLE-ABS ({targeted literature review*}) OR TITLE-ABS ({meta-synthes*}) OR TITLE-ABS ({comprehensive review*}) OR TITLE-ABS ({mixed studies review*}) OR TITLE-ABS ({sistematic review*}) OR TITLE-ABS ({umbrella review*}) OR TITLE-ABS ({mini-review*}) OR TITLE-ABS ({rapid literature review*}) OR TITLE-ABS ({rapid review*}) OR TITLE-ABS ({integrative review*}) OR TITLE-ABS ({systematic and critical analysis review*}) OR TITLE-ABS ({systematically review evidence}) OR TITLE-ABS ({systematic search*}) OR TITLE-ABS ({systematic methodological review*}) OR TITLE-ABS ({metaanalys*}) OR TITLE-ABS ({meta-analytic review*}) OR TITLE-ABS ({meta-analys*}) OR TITLE-ABS ({cochrane review*})).

CINAHL :

(MH"Cardiovascular Diseases+" OR MH"Hypercholesterolemia+" OR MH"Pacemaker, Artificial/adverse effects+" OR MH"brain neoplasms+" OR MH"Melanoma+" OR MH"Thyroid Diseases+" OR MH"Endocrine Diseases+" OR MH"Pheochromocytoma+" OR MH"Obesity+" OR MH"Respiratory Tract Diseases+" OR MH"Digestive System Diseases+" OR MH"Ethmoid Sinusitis+" OR MH"Vertigo+" OR MH"Colonic polyps+" OR MH"Digestive System Diseases+" OR MH"Back pain +" OR MH"Neck Pain+" OR MH"Intervertebral Disc Displacement+" OR MH"Arthritis+" OR MH"Sciatica+" OR MH"Axial Spondyloarthritis+" OR MH"Osteitis Deformans+" OR MH"Bursitis+" OR MH"Fatigue Syndrome, Chronic+" OR MH"Scoliosis+" OR MH"Reflex Sympathetic Dystrophy+" OR MH"Headache+" OR MH"Multiple sclerosis+" OR MH"Epilepsy+" OR MH"Dementia, Vascular+" OR MH"Parkinson Disease+" OR MH"Trigeminal Neuralgia+" OR MH"Guillain-Barre Syndrome+" OR MH"Nervous System Diseases+" OR MH"Stroke+" OR MH"Cerebral Ischemia, Transient+" OR MH"Muscular Diseases+" OR MH"Myasthenia Gravis+" OR MH"Renal Insufficiency, Chronic +" OR MH"Kidney Diseases+" OR MH"Skin Diseases+" OR MH"Herpes Simplex+" OR MH"eye diseases+" OR MH"Lupus Erythematosus, Systemic+" OR MH"Antiphospholipid Syndrome+" OR MH"Scleroderma, Systemic+" OR MH"Wegener's Granulomatosis+" OR MH"Microscopic Polyangiitis+" OR MH"Blood Protein Disorders+" OR MH"Sarcoidosis+" OR MH"Histiocytosis+" OR MH"Ehlers-Danlos Syndrome+" OR MH"Tuberculosis+" OR MH"Acquired Immunodeficiency Syndrome+" OR MH"Hepatitis, Chronic+" OR MH"Lyme Disease+" OR MH"Discitis+" OR MH"Leukemia+" OR MH"Hodgkin Disease+" OR

MH"Lymphoma, Large B-Cell, Diffuse+" OR MH"Multiple Myeloma+" OR MH"Down Syndrome+" OR MH"Myelodysplastic Syndromes+" OR MH"Anemia, Hemolytic+" OR MH"Anemia, Pernicious+" OR MH"Purpura, Thrombocytopenic+" OR MH"Thrombotic Microangiopathies+" OR MH"Hemochromatosis +" OR MH"Porphyrias+" OR MH"Hemophilia A+" OR MH"Fatigue Syndrome, Chronic+" OR MH"Chronic Pain+" OR MH"Bone Marrow Diseases+" OR TI "Basal Cell Epithelioma" OR TI "Basal Cell Epitheliomas" OR TI "Brain Metastase" OR TI "Brain Metastases" OR TI "cerebroma" OR TI "Colon Adenocarcinoma" OR TI "Colon Adenocarcinomas" OR TI "dermatoma" OR TI "encephalophyma" OR TI "hypophysoma" OR TI "kidney mass" OR TI "kidney neoplastic mass" OR TI "malignant melanomatosis" OR TI "melanocarcinoma" OR TI "Melanoma" OR TI "melanomalignoma" OR TI "Melanomas" OR TI "naevocarcinoma" OR TI "neoplastic lung" OR TI "neoplastic mammary gland" OR TI "nephroma" OR TI "nevocarcinoma" OR TI "Pituitary Adenoma" OR TI "Pituitary Adenomas" OR TI "rectal mass" OR TI "rectum mass" OR TI "renal mass" OR TI "renal neoplastic mass" OR TI "reninoma" OR TI "Rodent Ulcer" OR TI "Rodent Ulcers" OR TI "squamous cell epithelioma" OR TI "squamous epithelioma" OR TI "Thyroid Adenoma" OR TI "Thyroid Adenomas" OR TI "angina" OR TI "anginal attack" OR TI "angiocardiopathy" OR TI "angiocardiovascular disease" OR TI "angiocardiovascular diseases" OR TI "Angor Pectori" OR TI "Aortic Valve Diseases" OR TI "Aortic Valve Disorder" OR TI "Aortic Valve Disorders" OR TI "Arrhythmia" OR TI "Arrythmia" OR TI "Artificial Cardiac Pacemaker" OR TI "Artificial Cardiac Pacemakers" OR TI "Artificial Pacemaker" OR TI "Artificial Pacemakers" OR TI "Atrial Fibrillation" OR TI "Atrial Fibrillations" OR TI "Auricular Fibrillation" OR TI "Auricular Fibrillations" OR TI "backward failure heart" OR TI "backward failure, heart" OR TI "Cardiac Arrest" OR TI "Cardiac Arrhythmias" OR TI "cardiac backward failure" OR TI "cardiac conduction system disease" OR TI "cardiac decompensation" OR TI "Cardiac Disorders" OR TI "cardiac disrhythmia" OR TI "Cardiac Dysrhythmia" OR TI "cardiac failure" OR TI "cardiac failures" OR TI "cardiac incompetence" OR TI "cardiac incompetences" OR TI "cardiac infarct" OR TI "cardiac insufficien*" OR TI "cardiac stand still" OR TI "cardiac valve defect" OR TI "cardiac valve disease" OR TI "cardial decompensation" OR TI "cardial infarct" OR TI "cardial insufficiency" OR TI "cardial valve disease" OR TI "cardiovascular complication*" OR TI "cardiovascular decompensation" OR TI "cardiovascular disease" OR TI "cardiovascular diseases" OR TI "cardiovascular disorder" OR TI "cardiovascular disorders" OR TI "cardiovascular disturbance" OR TI "cardiovascular disturbances" OR TI "cardiovascular disturbancescardiovascular lesion*" OR TI "cardiovascular failure" OR TI "cardiovascular failures" OR TI "cardiovascular incompetence" OR TI "cardiovascular incompetences" OR TI "cardiovascular insufficien*" OR TI "cardiovascular lesion*" OR TI "cardiovascular syndrome" OR TI "cardiovascular syndromes" OR TI "cardiovascular vegetative disorder" OR TI "cardiovascular vegetative disorders" OR TI "cholesteremia" OR TI "cholesterinemia" OR TI "cholesterolemia" OR TI "Circulatory Disorders" OR TI "complication cardiovascular" OR TI "conduction defect" OR TI "conduction defects" OR TI "conduction disease" OR TI "conduction diseases" OR TI "conduction disorder" OR TI "conduction disorders" OR TI "conduction disturbance" OR TI "conduction disturbances" OR TI "Coronary Disorders" OR TI "decompensatio cordis" OR TI "decompensation, heart" OR TI "ectopic heart rhythm" OR TI "ectopic rhythm" OR TI "Elevated Cholesterol" OR TI "Elevated Cholesterols" OR TI "heart aberrant conduction" OR TI "heart arrhytmia" OR TI "Heart Attack" OR TI "Heart Attacks" OR TI "heart backward failure" OR TI "heart decompensation" OR TI "heart disease" OR TI "heart diseases" OR TI "heart disorder" OR TI "heart disorders" OR TI "heart dysrhythmia" OR TI "heart ectopic beat" OR TI "heart ectopic ventricle contraction" OR TI "heart failure" OR TI "heart failures" OR TI "heart incompetence" OR TI "heart infarct" OR TI "heart insufficien*" OR TI "heart rhythm disorder" OR TI "heart valve abnormalities" OR TI "heart valve abnormality" OR TI "heart valve defect" OR TI "heart valve degeneration" OR TI "Heart Valve Disease" OR TI "heart valve diseases" OR TI "heart valve lesion" OR TI "Heart Valvular Disease" OR TI "Heart Valvular Diseases" OR TI "High Blood Pressure" OR TI "High Blood Pressures" OR TI "High Cholesterol

Level" OR TI "High Cholesterol Levels" OR TI "Hypercholesteremia" OR TI "Hypercholesteremias" OR TI "hypercholesterinaemia" OR TI "hypercholesterinemia" OR TI "hypercholesterolaemia" OR TI "Hypercholesterolemias" OR TI "hypertensive disease" OR TI "hypertensive effect" OR TI "hypertensive pulmonary vascular disease" OR TI "hypertensive response" OR TI "insufficientia cardis" OR TI "insufficientio cardis" OR TI "lung embolism" OR TI "lung embolization" OR TI "lung embolus" OR TI "lung emboly" OR TI "lung microembolism" OR TI "lung microembolization" OR TI "lung microembolus" OR TI "lung thromboembolism" OR TI "major adverse cardiovascular event*" OR TI "Myocardial Failure" OR TI "Myocardial Infarct" OR TI "myocardial insufficiency" OR TI "Myocardial lnfarcts" OR TI "myocardium infarct" OR TI "Periphlebitides" OR TI "Periphlebitis" OR TI "perivenous infection" OR TI "Phlebitides" OR TI "phlebitis superficialis" OR TI "post phlebitis syndrome" OR TI "postphlebitic disease" OR TI "postphlebitis syndrome" OR TI "Pulmonary Embolism" OR TI "Pulmonary Embolisms" OR TI "pulmonary embolization" OR TI "pulmonary embolus" OR TI "pulmonary hypertensive diseases" OR TI "pulmonary hypertensive disorder" OR TI "pulmonary hypertensive disorders" OR TI "pulmonary microembolism" OR TI "pulmonary thromboembolic disease" OR TI "Pulmonary Thromboembolism" OR TI "Pulmonary Thromboembolisms" OR TI "Raynauds Disease" OR TI "Stenocardia" OR TI "Stenocardias" OR TI "superficial phlebitis" OR TI "valvulopathies" OR TI "valvulopathy" OR TI "Vascular Disorders" OR TI "Vasculitis" OR TI "vein inflammation" OR TI "venitis" OR TI "venous inflammation" OR TI "vitium cordis" OR TI "Chronic Infectious Mononucleosis-Like Syndromes" OR TI "chronic intractable pain" OR TI "Royal Free Diseases" OR TI "Systemic Exertion Intolerance Diseases" OR TI "Chronic Pain" OR TI "Chronic Pains" OR TI "Achalasia" OR TI "Achalasias" OR TI "alcohol liver injury" OR TI "alcoholic liver" OR TI "Atrophic Gastritides" OR TI "Atrophic Gastritis" OR TI "autoimmune gastritis" OR TI "bowel syndrome" OR TI "cardiac herniation" OR TI "cardioesophageal reflux" OR TI "cardiooesophageal reflux" OR TI "Cardiospasm" OR TI "Cardiospasms" OR TI "Celiac Disease" OR TI "celiac syndrome" OR TI "chronic inflammatory bowel diseases" OR TI "chronic ulceration colon" OR TI "cirrhosis" OR TI "cleron disease" OR TI "coeliac disease" OR TI "coeliac syndrome" OR TI "coeliaky" OR TI "Colitis Gravis" OR TI "colitis ulcerativa" OR TI "colitis ulcerosa" OR TI "colon disease" OR TI "colon diseases" OR TI "colon disorder" OR TI "colon disorders" OR TI "colon juvenile polyp" OR TI "colon polypoid lesion" OR TI "colon spasm" OR TI "chronic colon ulceration" OR TI "Colonic Polyp" OR TI "Colonic Polyps" OR TI "colonospasm" OR TI "Crohns Disease" OR TI "Crohn's Disease" OR TI "Crohn's Enteritis" OR TI "digestive disease" OR TI "digestive diseases" OR TI "digestive disorder" OR TI "digestive disorders" OR TI "digestive system disorders" OR TI "enteritis regionalis" OR TI "Esophageal Hernia*" OR TI "Esophageal Reflux" OR TI "esophageal regurgitation" OR TI "esophagogastric reflux" OR TI "esophagus hernia" OR TI "esophagus reflux" OR TI "functional colonic diseases" OR TI "Gastric Acid Reflux" OR TI "gastric atrophy" OR TI "gastric regurgitation" OR TI "Gastroduodenal Ulcer" OR TI "Gastroduodenal Ulcers" OR TI "gastroesophageal reflex" OR TI "gastroesophageal regurgitation" OR TI "gastroesophagus reflux" OR TI "gastrointestinal disease" OR TI "gastrointestinal diseases" OR TI "gastrointestinal disorder" OR TI "gastrointestinal disorders" OR TI "gastrointestinal dysfunction*" OR TI "gastrooesophageal reflex" OR TI "gastrooesophageal reflux" OR TI "Gastro-oesophageal Reflux" OR TI "gastrooesophageal regurgitation" OR TI "gee herter disease" OR TI "gee thaysen disease" OR TI "GERD" OR TI "Gluten Enteropathies" OR TI "Gluten Enteropathy" OR TI "gluten induced enteropathy" OR TI "gluten intolerance" OR TI "Gluten Sensitive Enteropathy" OR TI "Gluten-Sensitive Enteropathies" OR TI "Gluten-Sensitive Enteropathy" OR TI "Granulomatous Colitis" OR TI "Granulomatous Enteritis" OR TI "hernia hiatus esophagi" OR TI "hiatal diaphragmatic hernia" OR TI "Hiatal Hernia*" OR TI "hiatus diaphragmatic hernia" OR TI "hiatus esophagi hernia" OR TI "Hiatus Hernia*" OR TI "hiatus oesophageal hernia" OR TI "hiatus oesophagus hernia" OR TI "huebner herter disease" OR TI "Idiopathic Proctocolitis" OR TI "Ileocolitis" OR TI "Inflammatory Bowel Disease" OR TI "intestinal infantilism" OR TI "Irritable Bowel Syndromes" OR TI "Irritable Colon" OR TI "Liver Fibrosis"

OR TI "Marginal Ulcer" OR TI "Marginal Ulcers" OR TI "Megaesophagus" OR TI "morbus crohn" OR TI "mucomembraneous colitis" OR TI "mucomembranous colitis" OR TI "mucosal colitis" OR TI "Mucous Colitides" OR TI "Mucous Colitis" OR TI "oesophageal reflux" OR TI "oesophageal regurgitation" OR TI "oesophagogastric reflux" OR TI "oesophagus reflux" OR TI "Paraesophageal Hernia" OR TI "para-esophageal hernia" OR TI "Paraesophageal Hernias" OR TI "paraesophageal herniation" OR TI "para-esophageal herniation" OR TI "paraoesophageal hernia" OR TI "para-oesophageal hernia" OR TI "paraoesophageal herniation" OR TI "para-oesophageal herniation" OR TI "Peptic Ulcers" OR TI "Regional Enteritis" OR TI "regional enterocolitis" OR TI "Regional Ileitides" OR TI "Regional Ileitis" OR TI "regurgitation, gastroesophageal" OR TI "spastic colitis" OR TI "spastic colon" OR TI "Sprue" OR TI "stomach hernia" OR TI "Terminal Ileitis" OR TI "Ulcerative Colitis" OR TI "ulcerative colorectitis" OR TI "ulcerative procto colitis" OR TI "ulcerative proctocolitis" OR TI "ulcerous colitis" OR TI "unstable colon" OR TI "acromegalia" OR TI "Acromegalies" OR TI "acromegalism" OR TI "Acromegaly" OR TI "Addison Disease" OR TI "Addisons Disease" OR TI "Addison's Disease" OR TI "adipose tissue hyperplasia" OR TI "adipositas" OR TI "adiposity" OR TI "adrenal cortex hyperplasia" OR TI "adrenal cortical hyperplasia" OR TI "adrenal failure" OR TI "adrenal failures" OR TI "adrenal gland disorders" OR TI "adrenocortical hyperplasia" OR TI "adrenocorticohyperplasia" OR TI "akromegalia" OR TI "Aldosteronism" OR TI "antidiuretic hormone insufficiency" OR TI "arenocortical hyperplasia" OR TI "Basedow Disease" OR TI "basedow syndrome" OR TI "Basedows Disease" OR TI "Basedow's Disease" OR TI "chromaffin paraganglioma" OR TI "chronic autoimmune thyroiditis" OR TI "Chronic Lymphocytic Thyroiditides" OR TI "Chronic Lymphocytic Thyroiditis" OR TI "Conn Syndrome" OR TI "Conns Syndrome" OR TI "Conn's Syndrome" OR TI "corpulency" OR TI "Cushing syndrome" OR TI "Cushings syndrome" OR TI "Cushing's Syndrome" OR TI "diabetes" OR TI "diabetic" OR TI "endocrinal disease" OR TI "endocrinal diseases" OR TI "endocrinal disorder" OR TI "endocrinal disorders" OR TI "endocrinal disturbance" OR TI "endocrinal disturbances" OR TI "endocrinal dysfunction*" OR TI "endocrine disease" OR TI "endocrine diseases" OR TI "endocrine disorder" OR TI "Endocrine Disorders" OR TI "endocrine disturbance" OR TI "endocrine disturbances" OR TI "endocrine dysfunction*" OR TI "endocrine gland disease" OR TI "endocrine gland diseases" OR TI "endocrine gland disorder" OR TI "endocrine gland disorders" OR TI "endocrine gland dysfunction*" OR TI "endocrine syndrome" OR TI "endocrine syndromes" OR TI "endocrine system disease" OR TI "endocrine system diseases" OR TI "endocrine system disorder" OR TI "endocrine system disorders" OR TI "endocrine system dysfunction*" OR TI "endocrinological disease" OR TI "endocrinological diseases" OR TI "endocrinological disorder" OR TI "endocrinological disorders" OR TI "endocrinological dysfunction*" OR TI "excess body weight" OR TI "Exophthalmic Goiter" OR TI "Exophthalmic Goiters" OR TI "exophthalmic goitre" OR TI "exophthalmic hyperthyroidism" OR TI "Extra Adrenal Pheochromocytoma" OR TI "Extra-Adrenal Pheochromocytoma" OR TI "familial hypoadrenocorticism" OR TI "fat overload syndrome" OR TI "feline hyperthyroidism" OR TI "Graves Disease" OR TI "Graves' Disease" OR TI "Graves hyperthyroidism" OR TI "graves syndrome" OR TI "Graves's disease" OR TI "Hashimoto Disease" OR TI "hashimoto goiter" OR TI "Hashimoto Struma" OR TI "Hashimoto Syndrome" OR TI "Hashimoto Thyroiditides" OR TI "Hashimoto Thyroiditis" OR TI "hashimoto thyroidosis" OR TI "Hashimotos Disease" OR TI "Hashimoto's Disease" OR TI "Hashimoto's Struma" OR TI "Hashimotos Syndrome" OR TI "Hashimoto's Syndrome" OR TI "Hashimoto's Syndromes" OR TI "Hashimotos thyroiditis" OR TI "Hashimoto's thyroiditis" OR TI "hormonal disease" OR TI "hormonal diseases" OR TI "hormonal disorder" OR TI "hormonal disorders" OR TI "hormonal dysfunction*" OR TI "hormone imbalance" OR TI "hyperaldosterone" OR TI "Hypercortisolism" OR TI "hypermineralocorticidism" OR TI "hypermineralocorticism" OR TI "Hyperprolactinaemia*" OR TI "Hyperprolactinemia" OR TI "Hyperprolactinemias" OR TI "hyperthyreoidism" OR TI "hyperthyreosis" OR TI "Hyperthyroid" OR TI "hyperthyroidea" OR TI "hyperthyroidosis" OR TI "Hyperthyroids" OR TI "hypogonadism" OR TI "hypothyreoidism" OR TI

"hypothyreosis" OR TI "hypothyroidea" OR TI "hypothyroidism" OR TI "Hypothyroidisms" OR TI "hypothyroidosis" OR TI "hypothyrosis" OR TI "Inappropriate GH Secretion Syndrome" OR TI "Inappropriate GH Secretion Syndromes" OR TI "Inappropriate Growth Hormone Secretion Syndrome" OR TI "Inappropriate Growth Hormone Secretion Syndromes" OR TI "Inappropriate Prolactin Secretion" OR TI "Inappropriate Secretion Prolactin" OR TI "lymphadenoid goiter" OR TI "megalakria" OR TI "mineralcorticoid excess syndrome" OR TI "obesitas" OR TI "obesity" OR TI "overweight" OR TI "phaeochromoblastoma" OR TI "phaeochromocytoma" OR TI "pheochromoblastoma" OR TI "Pheochromocytomas" OR TI "pheochromocytomata" OR TI "pheochromocytomatosis" OR TI "pheochromocytosis" OR TI "polycystic ovary syndrome" OR TI "polycystic ovary syndromes" OR TI "Primary Adrenal Insufficiency" OR TI "Primary Adrenocortical Insufficiencies" OR TI "Primary Adrenocortical Insufficiency" OR TI "Primary Hyperaldosteronism" OR TI "Primary Hyperthyroidism" OR TI "Primary Hypoadrenalism" OR TI "Primary Hypoadrenalisms" OR TI "Prolactin Hypersecretion Syndrome" OR TI "Prolactin Hypersecretion Syndromes" OR TI "Somatotropin Hypersecretion Syndrome" OR TI "Somatotropin Hypersecretion Syndromes" OR TI "struma hashimoto" OR TI "thyroid deficiency" OR TI "thyroid gland failure" OR TI "thyroid gland hyperfunction" OR TI "thyroid hyperfunction" OR TI "thyroid insufficiency" OR TI "Thyroid Stimulating Hormone Deficiency" OR TI "thyroideal hyperfunction" OR TI "Thyroid-Stimulating Hormone Deficiencies" OR TI "Thyroid-Stimulating Hormone Deficiency" OR TI "TSH Deficiencies" OR TI "TSH Deficiency" OR TI "Vasopressin Deficiency" OR TI "angioleiomyoma" OR TI "angiomyoma" OR TI "elastomyofibroma" OR TI "fibromyoma" OR TI "fibromyomas" OR TI "hemangioleiomyoma" OR TI "hemangiomyoma" OR TI "Impotence" OR TI "leimyoma" OR TI "Leiomyomas" OR TI "leyomyoma" OR TI "myofibroma" OR TI "myofibromatosis" OR TI "vascular leiomyoma" OR TI "addison anaemia" OR TI "addison anemia" OR TI "addisonian anaemia" OR TI "addisonian anemia" OR TI "ahf deficiency" OR TI "ahg deficiency" OR TI "anaemia perniciosa" OR TI "anemia perniciosa" OR TI "B12 deficiency anaemia" OR TI "B12 deficiency anemia" OR TI "B12 deficient anaemia" OR TI "B12 deficient anemia" OR TI "B12 vitamin deficiency anemia" OR TI "biermer anaemia" OR TI "biermer anemia" OR TI "biermer disease" OR TI "blood disease" OR TI "blood diseases" OR TI "blood disorder" OR TI "blood disorders" OR TI "blood dysfunction*" OR TI "classic haemophilia" OR TI "cobalamin deficiency anaemia" OR TI "cobalamin deficiency anemia" OR TI "congenital antihaemophilic factor deficiency" OR TI "congenital antihaemophilic globulin deficiency" OR TI "congenital antihemophilic factor deficiency" OR TI "congenital antihemophilic globulin deficiency" OR TI "drepanocytemia" OR TI "drepanocytic anaemia" OR TI "drepanocytic anemia" OR TI "drepanocytosis" OR TI "haematologic disease" OR TI "haematologic diseases" OR TI "haematologic disorder" OR TI "haematologic disorders" OR TI "haematologic dysfunction*" OR TI "haematological disease" OR TI "haematological diseases" OR TI "haematological disorder" OR TI "haematological disorders" OR TI "haematological dysfunction*" OR TI "haemochromatosis" OR TI "haemoglobin SS" OR TI "haemolytic anaemia" OR TI "haemolytic disease" OR TI "haemolytic syndrome" OR TI "haemophilia a" OR TI "haemophilia vera" OR TI "Hb SS disease" OR TI "heart hemochromatosis" OR TI "hemachromatosis" OR TI "hematologic disease" OR TI "hematologic diseases" OR TI "hematologic disorder" OR TI "hematologic disorders" OR TI "hematologic dysfunction*" OR TI "hematopathy" OR TI "hemoglobin SS" OR TI "hemolytic anemia" OR TI "hemolytic disease" OR TI "hemolytic syndrome" OR TI "hemopathie" OR TI "hemopathies" OR TI "hemopathy" OR TI "hemophylia type a" OR TI "hereditary iron overload" OR TI "idiopathic hemochromatosis" OR TI "intermittent porphyria" OR TI "iron overload disease" OR TI "iron overload disorder" OR TI "iron overload syndrome" OR TI "latent porphyria" OR TI "lymphatic disease" OR TI "lymphatic diseases" OR TI "lymphatic disorder" OR TI "lymphatic disorders" OR TI "lymphatic dysfunction*" OR TI "macrocytic hyperchromic anaemia" OR TI "macrocytic hyperchromic anemia" OR TI "meniscocytosis" OR TI "pernicious anaemia" OR TI "pernicious anemia" OR TI "porphyrias" OR TI "porphyric disease" OR TI "primary anaemia" OR TI "primary anemia" OR TI

"primary congenital hemochromatosis" OR TI "recklinghausen applebaum disease" OR TI "sickle anaemia" OR TI "sickle anemia" OR TI "sickle cell anaemia" OR TI "siderochromatosis" OR TI "sulfhemoglobinem*" OR TI "sulphaemoglobinaem*" OR TI "systemic porphyria" OR TI "thrombocytopaenia purpura" OR TI "thrombocytopenia purpura" OR TI "thrombotic purpura" OR TI "true haemophilia" OR TI "vitamin b 12 deficiency anaemia" OR TI "vitamin b 12 deficiency anemia" OR TI "a2 myeloma" OR TI "Acquired Hemolytic Anemias" OR TI "Acquired Immune Deficiency Syndrome Virus" OR TI "acquired immunodeficiency" OR TI "Addison Anemias" OR TI "Addisons Anemia" OR TI "Addison's Anemias" OR TI "AIDS" OR TI "aleukaemia" OR TI "aleukemia" OR TI "Arthriti*" OR TI "arthrochondritis" OR TI "arthrosynovitis" OR TI "B. burgdorferi Infection*" OR TI "bone marrow dysplasia" OR TI "Bone Marrow Fibros*" OR TI "Borrelia burgdorferi Infection*" OR TI "Bronze Diabete" OR TI "Bronzed Cirrhoses" OR TI "Chronic Hepatit*" OR TI "chronic myeloleukaemia" OR TI "chronic myeloleukemia" OR TI "Discitides" OR TI "discitis" OR TI "disk space infection" OR TI "Diskiti*" OR TI "DLBCL" OR TI "down disease" OR TI "Downs syndrome" OR TI "Down's syndrome" OR TI "Dysmyelopoietic Syndrome*" OR TI "endocardial inflammation" OR TI "Endocarditi*" OR TI "endo-carditis" OR TI "endocardium inflammation" OR TI "Erythremia*" OR TI "Factor 8 Deficienc*" OR TI "Factor VIII Deficienc*" OR TI "Factor VIII defiency" OR TI "Familial Hemochromatoses" OR TI "Familial Hemochromatosis" OR TI "Haemochromato" OR TI "Haemochromatos" OR TI "Haemolytic Anaemias" OR TI "HbS Disease" OR TI "Hematopoetic Myelodysplasias" OR TI "hemoblastoma" OR TI "Hemochromato" OR TI "Hemochromatos" OR TI "Hemoglobin S Disease" OR TI "Hemoglobin S Diseases" OR TI "Hemophilia" OR TI "hemophilias" OR TI "high-risk MDS" OR TI "Hodgkin disease*" OR TI "Hodgkin Granuloma*" OR TI "hodgkin sclerosis" OR TI "hodgkin's disease" OR TI "Hodgkins Disease*" OR TI "Hodgkins Granuloma" OR TI "Hodgkin's Granuloma" OR TI "HTLV-III" OR TI "Human Immunodeficiency Virus*" OR TI "Human T Cell Lymphotropic Virus Type III" OR TI "Human T Lymphotropic Virus Type III" OR TI "Human T-Cell Lymphotropic Virus Type III" OR TI "Human T-Lymphotropic Virus Type III" OR TI "infection by B. burgdorferi" OR TI "infection by Borrelia burgdorferi" OR TI "intervertebral disc infection" OR TI "intervertebral disk infection" OR TI "intervertebral disk inflammation" OR TI "Iron Storage Disorder*" OR TI "joint inflammation" OR TI "Koch Disease*" OR TI "Kochs Disease*" OR TI "Koch's Disease*" OR TI "langdon down syndrome" OR TI "LAV-HTLV-III" OR TI "leucaemia" OR TI "leucemia" OR TI "Leucocythaemia*" OR TI "Leucocythemia*" OR TI "leukaemia" OR TI "Leukemia*" OR TI "Lyme borrelioses" OR TI "Lyme Borreliosis" OR TI "Lyme's borrelioses" OR TI "Lyme's borreliosis" OR TI "Lymes disease" OR TI "Lyme's disease" OR TI "Lymphadenopathy Associated Virus*" OR TI "Lymphadenopathy-Associated Virus*" OR TI "lymphogranuloma maligne" OR TI "lymphogranuloma malignum" OR TI "lymphogranulomatosis" OR TI "Malignant Granuloma*" OR TI "Malignant Lymphogranuloma" OR TI "Malignant Lymphogranulomas" OR TI "Microangiopathic Anemias" OR TI "Microangiopathic Hemolytic Anemias" OR TI "mongolian idiocy" OR TI "mongolism" OR TI "mongoloid idiocy" OR TI "mongoloidism" OR TI "morbus hodgkin" OR TI "myelodysplasia" OR TI "myelodysplastic disease" OR TI "myelodysplastic disorder" OR TI "Myelodysplastic Syndrome*" OR TI "Myelofibros*" OR TI "Myeloid Metaplasia*" OR TI "myeloplaxoma" OR TI "Myeloscleros*" OR TI "Nonleukemic Myelos*" OR TI "oligoarthritis" OR TI "Osler Vaquez Disease*" OR TI "Osler-Vaquez Disease*" OR TI "Pernicious Anemias" OR TI "Pigmentary Cirrhos*" OR TI "Polyarthrit*" OR TI "Polycythemia Ruba Vera*" OR TI "Polycythemia Rubra Vera*" OR TI "Primary Hemochromatosis" OR TI "Primary Polycythemia*" OR TI "reed sternberg disease" OR TI "Sickle Cell Anemia" OR TI "Sickle Cell Anemias" OR TI "Sickle Cell Disease" OR TI "Sickle Cell Diseases" OR TI "sickle cell disorder" OR TI "Sickle Cell Disorders" OR TI "Sickling Disorder Due to Hemoglobin S" OR TI "Spondylodisciti*" OR TI "Spondylodiskiti*" OR TI "TB disease" OR TI "TB infection" OR TI "Thrombocytopenic Purpura*" OR TI "Thrombopenic Purpura*" OR TI "Thrombotic Microangiopath*" OR TI "translocation 15 21 22" OR TI "trisomy 21 syndrome" OR TI "Troisier Hanot Chauffard Syndrome" OR TI "Troisier-Hanot-Chauffard

Syndrome*" OR TI "Tuberculos*" OR TI "tuberculous infection" OR TI "tuberculous lesion" OR TI "Von Recklenhausen Applebaum Disease" OR TI "Von Recklenhausen-Applebaum Diseases" OR TI "Acute Autoimmune Neuropathies" OR TI "Acute Autoimmune Neuropathy" OR TI "Acute Cerebrovascular Accident*" OR TI "acute cerebrovascular lesion" OR TI "acute febrile polyneuritis" OR TI "acute focal cerebral vasculopathy" OR TI "Acute Infectious Polyneuritis" OR TI "Acute Inflammatory Demyelinating Polyneuropath*" OR TI "Acute Inflammatory Demyelinating Polyradiculoneuropath*" OR TI "Acute Inflammatory Polyneuropath*" OR TI "Acute Inflammatory Polyradiculoneuropath*" OR TI "acute postinfective polyradiculoneuropathy" OR TI "Acute Stroke*" OR TI "Alzeimer" OR TI "Alzheimer" OR TI "Alzeimers disease" OR TI "Alzeimer's disease" OR TI "Alzheimer's Disease" OR TI "Alzheimers Disease*" OR TI "Alzheimer's Diseases" OR TI "Alzheimer-Type Dementia" OR TI "Anti-MuSK Myasthenia Gravis" OR TI "Apoplex*" OR TI "Arteriosclerotic Dementia*" OR TI "Aura" OR TI "Auras" OR TI "autoimmune myasthenia gravis" OR TI "Binswanger Disease*" OR TI "Binswanger Encephalopath*" OR TI "Binswangers Disease*" OR TI "Binswanger's Disease*" OR TI "Binswangers Encephalopath*" OR TI "Binswanger's Encephalopath*" OR TI "brain accident" OR TI "brain attack" OR TI "brain blood flow disturbance" OR TI "brain insult" OR TI "brain insultus" OR TI "Brain TIA" OR TI "Brain Vascular Accident*" OR TI "Cephalgia Syndrome" OR TI "Cephalgia Syndromes" OR TI "cerebral insult" OR TI "Cerebral Stroke*" OR TI "cerebral vascular accident" OR TI "cerebral vascular insufficiency" OR TI "cerebro vascular accident" OR TI "Cerebrovascular Accident" OR TI "Cerebrovascular Accidents" OR TI "cerebrovascular arrest" OR TI "cerebrovascular failure" OR TI "cerebrovascular injury" OR TI "cerebrovascular insufficiency" OR TI "cerebrovascular insult" OR TI "Cerebrovascular Stroke*" OR TI "cerebrum vascular accident" OR TI "Chronic Daily Headache" OR TI "Chronic Daily Headaches" OR TI "Chronic Headache" OR TI "Chronic Headaches" OR TI "Chronic Progressive Subcortical Encephalopath*" OR TI "comitial disease" OR TI "congenital atonic sclerotic muscular dystrophy" OR TI "congenital muscular dystrophy" OR TI "CVA" OR TI "CVAs" OR TI "diffuse cortical sclerosis" OR TI "Disseminated Sclerosis" OR TI "Epileps*" OR TI "epileptic" OR TI "Epileptiform Neuralgia*" OR TI "erb goldflam disease" OR TI "falling sickness" OR TI "Fisher syndrome" OR TI "Fothergill Disease" OR TI "Generalized Myasthenia Gravis" OR TI "Guillain Barre" OR TI "Guillain-Barré" OR TI "Guillain-Barre" OR TI "Guillain Barré Syndrome*" OR TI "Headache Disorder" OR TI "Headache Syndrome" OR TI "Headache Syndromes" OR TI "Idiopathic Parkinson Disease*" OR TI "idiopathic parkinsonism" OR TI "infectious neuronitis" OR TI "inflammatory acute polyradiculoneuropathy" OR TI "Inflammatory Polyneuropathy Acute" OR TI "insular sclerosis" OR TI "insultus cerebralis" OR TI "Intractable Headache" OR TI "Intractable Headaches" OR TI "ischaemic attack" OR TI "ischaemic cerebral attack" OR TI "ischaemic seizure" OR TI "ischemic attack" OR TI "ischemic cerebral attack" OR TI "ischemic seizure" OR TI "lacunar dementia" OR TI "Landry paralysis" OR TI "Landry syndrome" OR TI "Landry-Guillain-Barre Syndrome" OR TI "Lewy Body Parkinson Disease*" OR TI "maternal myasthenia gravis" OR TI "mini-stroke" OR TI "multiinfarct dementia" OR TI "multi-infarct dementia" OR TI "multiinfarction dementia" OR TI "multi-infarction dementia" OR TI "multiple sclerosis" OR TI "muscle dystrophia" OR TI "muscle dystrophy" OR TI "Muscle Specific Receptor Tyrosine Kinase Myasthenia Gravis" OR TI "Muscle Specific Tyrosine Kinase Antibody Positive Myasthenia Gravis" OR TI "Muscle-Specific Receptor Tyrosine Kinase Myasthenia Gravis" OR TI "Muscle-Specific Tyrosine Kinase Antibody Positive Myasthenia Gravis" OR TI "muscular dystrophia" OR TI "muscular dystrophies" OR TI "MuSK MG" OR TI "MuSK Myasthenia Gravis" OR TI "myasthenia gravis pseudoparalitica" OR TI "myasthenia gravis pseudoparalytica" OR TI "myodystrophia" OR TI "myodystrophy" OR TI "neonatal myasthenia gravis" OR TI "nervous disease" OR TI "nervous diseases" OR TI "nervous disorder" OR TI "nervous disorders" OR TI "nervous dysfunction*" OR TI "nervous system disease" OR TI "nervous system diseases" OR TI "nervous system disorder" OR TI "nervous system disorders" OR TI "nervous system dysfunction*" OR TI "neurologic disease" OR TI "neurologic diseases" OR TI "neurologic disorder" OR TI "neurologic

disorders" OR TI "neurologic dysfunction*" OR TI "neurological disease" OR TI "neurological diseases" OR TI "neurological disorder" OR TI "neurological disorders" OR TI "neurological dysfunction*" OR TI "neuromuscular disease" OR TI "neuromuscular diseases" OR TI "neuromuscular disorder" OR TI "neuromuscular disorders" OR TI "neuromuscular dysfunction*" OR TI "Ocular Myasthenia Gravis" OR TI "Paralysis Agitans" OR TI "Parkinson dementia complex" OR TI "Parkinsons disease" OR TI "Parkinson disease" OR TI "Parkinson's Disease*" OR TI "Presenile Dementia" OR TI "Primary Parkinsonism" OR TI "Primary Senile Degenerative Dementia" OR TI "sclerosis multiplex" OR TI "Seizure Disorder*" OR TI "Senile Dementia" OR TI "stroke" OR TI "Strokes" OR TI "Subcortical Arteriosclerotic Encephalopath*" OR TI "Subcortical Leukoencephalopathies" OR TI "Subcortical Leukoencephalopathy" OR TI "Tic Douloureux" OR TI "transient brain ischaemia" OR TI "transient brain ischemia" OR TI "Transient Brain Stem Ischemia*" OR TI "Transient Brainstem Ischemia*" OR TI "Transient Cerebral Ischemia*" OR TI "Transient Cerebral Ischaemia*" OR TI "transient ischaemic attack*" OR TI "transient ischaemic seizure*" OR TI "Transient Ischemic Attack*" OR TI "transient ischemic seizure*" OR TI "Trifacial Neuralgia*" OR TI "Trigeminal Neuralgia*" OR TI "Vascular Dementia*" OR TI "acute paranasal sinusitis" OR TI "airway disease" OR TI "airway diseases" OR TI "airway disorder*" OR TI "airway dysfunction*" OR TI "Asthma*" OR TI "Atrophic Rhinitides" OR TI "Atrophic Rhinitis" OR TI "Auditory Vertigo" OR TI "Auditory Vertigos" OR TI "Aural Vertigo" OR TI "benign paroxysmal postural vertigo" OR TI "benign postural paroxysmal vertigo" OR TI "bronchitis chronica" OR TI "cerebral vertigo" OR TI "Chronic Airflow Obstruction*" OR TI "chronic airway obstruction" OR TI "Chronic Bronchitis" OR TI "chronic bronchus infection" OR TI "chronic emphysema" OR TI "chronic obstructive bronchopulmonary disease" OR TI "Chronic Obstructive Lung Disease" OR TI "chronic obstructive lung disorder" OR TI "Chronic Obstructive Pulmonary Disease" OR TI "Chronic Obstructive Pulmonary Diseases" OR TI "chronic obstructive pulmonary disorder" OR TI "chronic pulmonary obstructive disease" OR TI "chronic pulmonary obstructive disorder" OR TI "COAD" OR TI "cochlea hydrops" OR TI "COPD" OR TI "Cystic Fibrosis of Pancreas" OR TI "diffuse parenchyma lung disease" OR TI "Diffuse Parenchymal Lung Disease" OR TI "Diffuse Parenchymal Lung Diseases" OR TI "diffuse parenchymal pulmonary disease" OR TI "diffuse parenchymal pulmonary disorder" OR TI "endolymphatic hydrops" OR TI "endolymphatic sac hydrops" OR TI "Ethmoid Sinusitides" OR TI "Ethmoid Sinusitis" OR TI "Ethmoidal Sinusitides" OR TI "Ethmoidal Sinusitis" OR TI "fibrocystic disease" OR TI "hydrops labyrinthi" OR TI "Hypersomnia with Periodic Respiration" OR TI "Interstitial Lung Disease" OR TI "Interstitial Lung Diseases" OR TI "interstitial lung disorder" OR TI "Interstitial Pneumonia" OR TI "Interstitial Pneumonias" OR TI "Interstitial Pneumonitides" OR TI "Interstitial Pneumonitis" OR TI "interstitial pneumopathy" OR TI "interstitial pulmonary disease" OR TI "interstitial pulmonary disorder" OR TI "labyrinth hydrops" OR TI "labyrinthal syndrome" OR TI "lung allergy" OR TI "lung chronic obstructive disease" OR TI "Meniere Disease" OR TI "Ménière Disease" OR TI "Ménière Diseases" OR TI "Meniere Syndrome" OR TI "Ménière Vertigo" OR TI "Menieres Disease" OR TI "Meniere's Disease" OR TI "Ménières Disease" OR TI "Ménière's Disease" OR TI "Ménière's Diseases" OR TI "Menieres Syndrome" OR TI "Meniere's Syndrome" OR TI "Ménières Vertigo" OR TI "Ménière's Vertigo" OR TI "Ménière's Vertigos" OR TI "Mucoviscidosis" OR TI "mucoviscoidosis" OR TI "nasal sinusitis" OR TI "nocturnal apnea" OR TI "nocturnal apnoea" OR TI "obstructive chronic lung disease" OR TI "obstructive chronic pulmonary disease" OR TI "Otogenic Vertigo" OR TI "Otogenic Vertigos" OR TI "Otoscleroses" OR TI "otosclerosis surgery" OR TI "otosclerotic stapes" OR TI "otosphongiosis" OR TI "Otospongioses" OR TI "Otospongiosis" OR TI "Ozena" OR TI "Ozenas" OR TI "pancreas cystic disease" OR TI "pancreas cystic fibrosis" OR TI "Pancreas Fibrocystic Diseases" OR TI "pancreas fibrosis" OR TI "pancreatic cystic disease" OR TI "Pancreatic Cystic Fibrosis" OR TI "pancreatic fibrosis" OR TI "paroxysmal labyrinthine vertigo" OR TI "paroxysmal positional vertigo" OR TI "pneumatosis" OR TI "positional paroxysmal vertigo" OR TI "Pulmonary Cystic Fibrosis" OR TI "respiration disease" OR TI "respiration diseases" OR

TI "respiration disorder" OR TI "respiration disorders" OR TI "respiration dysfunction*" OR TI "respiration tract disease" OR TI "respiration tract diseases" OR TI "respiration tract disorder" OR TI "respiration tract disorders" OR TI "respiration tract dysfunction*" OR TI "respiratory disease" OR TI "respiratory diseases" OR TI "respiratory disorder" OR TI "respiratory disorders" OR TI "respiratory illness*" OR TI "respiratory tract disease" OR TI "respiratory tract diseases" OR TI "respiratory tract disorder" OR TI "respiratory tract disorders" OR TI "respiratory tract dysfunction" OR TI "respiratory tract dysfunctioning" OR TI "respiratory tract dysfunctions" OR TI "rhinitis atrophica" OR TI "sinusitis nasalis" OR TI "Sleep Apnea" OR TI "Sleep Apneas" OR TI "sleep apnoea" OR TI "Sleep Disordered Breathing" OR TI "Sleep Hypopnea" OR TI "Sleep Hypopneas" OR TI "Sleep-Disordered Breathing" OR TI "vertiginous disease" OR TI "vertiginous disorder" OR TI "vertiginous syndrome" OR TI "vestibular vertigo" OR TI "Age Related Osteoporosis" OR TI "Age-Related Bone Loss" OR TI "Age-Related Bone Losses" OR TI "Age-Related Osteoporosis" OR TI "Akureyri disease" OR TI "Algodystrophic Syndrome" OR TI "Algodystrophies" OR TI "Algodystrophy" OR TI "alibert bazin disease" OR TI "arthropathic psoriasis" OR TI "Arthroses" OR TI "Arthrosis" OR TI "axial spondylarthritis" OR TI "Axial Spondyloarthritides" OR TI "Axial Spondyloarthritis" OR TI "AxSpA" OR TI "Back Ache" OR TI "Back Aches" OR TI "Back Pain*" OR TI "Backache" OR TI "Backaches" OR TI "backpain" OR TI "beauvais disease" OR TI "Bilateral Sciatica" OR TI "Bilateral Sciaticas" OR TI "Bone Paget Disease" OR TI "Bone Pagets Disease" OR TI "Calcium Pyrophosphate Deposition Disease" OR TI "Calcium Pyrophosphate Dihydrate Deposition" OR TI "Cervical Pain" OR TI "Cervical Pains" OR TI "Cervical Sympathetic Dystrophies" OR TI "Cervical Sympathetic Dystrophy" OR TI "Cervicalgia" OR TI "Cervicalgias" OR TI "Cervicodynia" OR TI "Cervicodynias" OR TI "chariot disease" OR TI "Chondrocalcinoses" OR TI "chronic articular rheumatism" OR TI "chronic fatigue" OR TI "Chronic Fatigue-Fibromyalgia Syndrome*" OR TI "Chronic Infectious Mononucleosis Like Syndrome" OR TI "Chronic Infectious Mononucleosis-Like Syndrome" OR TI "chronic rheumatism" OR TI "complex regional pain syndrome 1" OR TI "complex regional pain syndrome type 1" OR TI "CRPS 1" OR TI "CRPS I" OR TI "CRPS type 1" OR TI "CRPS Type I" OR TI "CRPS-I" OR TI "crystal arthropathies" OR TI "crystalline arthropathy" OR TI "degenerative joint disease" OR TI "Diffuse Myofascial Pain Syndrome" OR TI "disc hernia" OR TI "Disc Herniation" OR TI "Disc Herniations" OR TI "disc prolapse" OR TI "Disc Protrusion" OR TI "Disc Protrusions" OR TI "discal hernia" OR TI "discal herniation" OR TI "discus hernia" OR TI "disk hernia" OR TI "Disk Herniation" OR TI "Disk Herniations" OR TI "Disk Prolapse" OR TI "Disk Prolapses" OR TI "Disk Protrusion" OR TI "Disk Protrusions" OR TI "dorsalgia" OR TI "epicondylalgia" OR TI "epidemic neuromyasthenia" OR TI "fatigue syndrome" OR TI "fibro myalgia" OR TI "Fibromyalgia Fibromyositis Syndrome" OR TI "Fibromyalgia-Fibromyositis Syndrome" OR TI "Fibromyalgia-Fibromyositis Syndromes" OR TI "Fibromyalgias" OR TI "Fibromyositis Fibromyalgia Syndrome" OR TI "Fibromyositis-Fibromyalgia Syndrome" OR TI "Fibromyositis-Fibromyalgia Syndromes" OR TI "fibrositic nodule" OR TI "Fibrositides" OR TI "Fibrositis" OR TI "hernia disci" OR TI "hernia nuclei pulposi" OR TI "Herniated Disc" OR TI "Herniated Discs" OR TI "Herniated Disk" OR TI "Herniated Disks" OR TI "herniated intervertebral disc" OR TI "herniated intervertebral disk" OR TI "herniated nucleus pulposus" OR TI "herniated vertebral disc" OR TI "herniated vertebral disk" OR TI "hypertrophic infiltrative tendinitis" OR TI "Iceland disease" OR TI "Intervertebral Disc Displacement" OR TI "Intervertebral Disc Displacements" OR TI "Intervertebral Disk Displacement" OR TI "Intervertebral Disk Displacements" OR TI "intervertebral disk perforation" OR TI "intervertebral disk rupture" OR TI "intervertebral prolapse" OR TI "Involutional Osteoporosis" OR TI "ischias" OR TI "ischiatic pain" OR TI "Lateral Epicondylitides" OR TI "Lateral Epicondylitis" OR TI "Lateral Humeral Epicondylitides" OR TI "Lateral Humeral Epicondylitis" OR TI "loin pain" OR TI "lowback pain" OR TI "Lumbago" OR TI "lumbal pain" OR TI "lumbal syndrome" OR TI "lumbalgesia" OR TI "lumbalgia" OR TI "lumbar pain" OR TI "lumbar spine syndrome" OR TI "lumbodynia" OR TI "lumbosacral pain" OR TI "lumbosacral root syndrome" OR TI "lumbosacroiliac strain" OR TI "Muscular Rheumatism" OR TI

"Myalgic Encephalomyelitis" OR TI "Neck Ache" OR TI "Neck Aches" OR TI "Neck Pain*" OR TI "Neckache" OR TI "Neckaches" OR TI "neuralgic shoulder amyotrophy" OR TI "nodular tendinitis" OR TI "nucleus pulposus hernia" OR TI "Osseous Paget's Disease" OR TI "osteitis deformans" OR TI "Osteoarthritides" OR TI "osteo-arthritis" OR TI "Osteoarthroses" OR TI "Osteoarthrosis" OR TI "osteo-arthrosis" OR TI "Osteoporoses" OR TI "ostitis deformans" OR TI "Paget Disease of Bone" OR TI "Paget disease of the bone" OR TI "Pagets bone disease" OR TI "Paget's bone disease" OR TI "Pagets disease of bone" OR TI "Paget's Disease of Bone" OR TI "Paget's disease of the bone" OR TI "paralytic scoliosis" OR TI "Post Traumatic Osteoporosis" OR TI "posttraumatic dystrophy" OR TI "post-traumatic dystrophy" OR TI "Post-Traumatic Osteoporosis" OR TI "Postviral Fatigue Syndromes" OR TI "Primary Fibromyalgia" OR TI "primary osteoarthritis" OR TI "progressive scoliosis" OR TI "Prolapsed Disc" OR TI "Prolapsed Discs" OR TI "Prolapsed Disk" OR TI "Prolapsed Disks" OR TI "Protruded Disc" OR TI "Protruded Discs" OR TI "Protruded Disk" OR TI "Protruded Disks" OR TI "Pseudogout" OR TI "Psoriasis Arthropathica" OR TI "psoriasis pustulosa arthropathica" OR TI "Psoriatic Arthropathies" OR TI "Psoriatic Arthropathy" OR TI "psoriatic rheumatism" OR TI "Reflex Sympathetic Dystrophies" OR TI "Reflex Sympathetic Dystrophy" OR TI "rheumarthritis" OR TI "rheumatic disease" OR TI "rheumatic diseases" OR TI "rheumatoid disease" OR TI "rheumatoid diseases" OR TI "rheumatoid inflammation" OR TI "rheumatological disease" OR TI "rheumatological diseases" OR TI "rheumatological disorder" OR TI "rheumatological disorders" OR TI "Royal Free Disease" OR TI "Sciatic Neuralgia" OR TI "Sciatic Neuralgias" OR TI "sciatic pain" OR TI "Scolioses" OR TI "Secondary Fibromyalgia" OR TI "Senile Osteoporosis" OR TI "shoulder arm syndrome" OR TI "Shoulder Hand Syndrome" OR TI "Shoulder-Hand Syndrome" OR TI "Shoulder-Hand Syndromes" OR TI "Slipped Disc" OR TI "Slipped Discs" OR TI "Slipped Disk" OR TI "Slipped Disks" OR TI "slipped intervertebral disc" OR TI "slipped vertebral disc" OR TI "spinal disk disease" OR TI "Sudek Atrophy" OR TI "Sudek's Atrophies" OR TI "Sudeks Atrophy" OR TI "Sudek's Atrophy" OR TI "sympathetic dystrophy syndrome" OR TI "Sympathetic Reflex Dystrophia" OR TI "Sympathetic Reflex Dystrophias" OR TI "sympathetic reflex dystrophy" OR TI "Systemic Exertion Intolerance Disease" OR TI "tendinopathy" OR TI "tendinosis" OR TI "tendonitis" OR TI "tendonopathy" OR TI "Tennis Elbow" OR TI "Tennis Elbows" OR TI "tenonitis" OR TI "tenontitis" OR TI "tenositis" OR TI "Type I Complex Regional Pain Syndrome" OR TI "Vertebrogenic Pain Syndrome" OR TI "Vertebrogenic Pain Syndromes" OR TI "Yuppie flu" OR TI "Acne Inversa*" OR TI "acne juvenilis" OR TI "Acne Rosacea" OR TI "Chronic Bullous Disease of Childhood" OR TI "cutaneous disease" OR TI "cutaneous diseases" OR TI "cutaneus disorder" OR TI "cutaneus disorders" OR TI "dermal disease" OR TI "dermal diseases" OR TI "dermal disorder" OR TI "dermal disorders" OR TI "Drug induced Linear IgA Bullous Dermatos*" OR TI "Drug-induced Linear IgA Bullous Dermatos*" OR TI "Eczema*" OR TI "Erythematotelangiectatic Rosacea" OR TI "Granulomatous Rosacea" OR TI "herpes" OR TI "hidradenitis suppurativa" OR TI "Hives" OR TI "juvenile acne" OR TI "Linear IgA Dermatos*" OR TI "Linear IgA IgG Bullous Dermatos*" OR TI "Linear IgA IgG Dermatos*" OR TI "Ocular Rosacea" OR TI "Palmoplantaris Pustulosis" OR TI "Papulopustular Rosacea" OR TI "Phymatous Rosacea" OR TI "Psoriases" OR TI "psoriasiform dermatitis" OR TI "psoriasiform dermatosis" OR TI "psoriasiform lesion" OR TI "psoriasiform rash" OR TI "psoriasiform skin rash" OR TI "psoriatic epidermis" OR TI "psoriatic skin" OR TI "Pustular Psoriasis of Palms and Soles" OR TI "Pustulosis of Palms and Soles" OR TI "Pustulosis Palmaris et Plantaris" OR TI "rhinophyma" OR TI "rozacea" OR TI "skin and connective tissue disease" OR TI "skin and connective tissue diseases" OR TI "skin and connective tissue disorder" OR TI "skin and connective tissue disorders" OR TI "skin disease" OR TI "skin diseases" OR TI "skin disorder" OR TI "skin disorders" OR TI "Suppurative Hidradeniti*" OR TI "urticary" OR TI "weal" OR TI "wheal" OR TI "whealing" OR TI "willan lepra" OR TI "active TB" OR TI "Adamantiades-Behcet Disease*" OR TI "Allergic Angiit*" OR TI "Allergic Granulomatos*" OR TI "Allergic Granulomatous and Angiitis" OR TI "Allergic Granulomatous Angiit*" OR TI "allergic granulomatous angitis" OR TI "anonymous artery

occlusion" OR TI "Anti Phospholipid Antibody Syndrome*" OR TI "Anti Phospholipid Syndrome*" OR TI "Antiphospholipid Antibody Syndrome*" OR TI "Anti-Phospholipid Antibody Syndrome*" OR TI "antiphospholipid syndrome" OR TI "Anti-Phospholipid Syndrome*" OR TI "aorta arch syndrome" OR TI "aortic arch syndromes" OR TI "Aortitis Syndrome*" OR TI "APLA syndrome" OR TI "arteritis brachiocephalica" OR TI "arteritis nodosa" OR TI "autoimmune disease" OR TI "autoimmune diseases" OR TI "Behcet Disease*" OR TI "Behçet Disease*" OR TI "Behcet syndrome" OR TI "behcet ulcer" OR TI "Behcets disease" OR TI "Behcet's Disease*" OR TI "Behcets syndrome" OR TI "Behcet's Syndrome*" OR TI "besnier boeck syndrome" OR TI "Besnier-Boeck Disease*" OR TI "Besnier-Boeck-Schaumann Syndrome*" OR TI "Boeck Disease*" OR TI "Boecks Disease*" OR TI "Boeck's Disease*" OR TI "brachiocephalic arteritis" OR TI "brachiocephalic artery occlusion" OR TI "brachiocephalic ischaemia" OR TI "brachiocephalic ischemia" OR TI "brachiocephalic trunk occlusion" OR TI "brachiocephalic vascular occlusion" OR TI "Church Strauss syndrome" OR TI "Churg Strauss" OR TI "Churg-Strauss Syndrome" OR TI "Cranial Arterit*" OR TI "cryoglobulinaemia" OR TI "Cryoglobulinemias" OR TI "cryoimmunoglobulinaemia" OR TI "cryoimmunoglobulinemia" OR TI "Cutis Elastica" OR TI "dacryosialoadenopathia atrophicans" OR TI "EDS IV" OR TI "Ehlers Danlos" OR TI "Ehlers-Danlos Disease*" OR TI "Ehlers-Danlos syndrome" OR TI "eosinophilic GPA" OR TI "eosinophilic granulomatosis polyangiitis" OR TI "eosinophilic granulomatosis polyangitis" OR TI "eosinophilic granulomatous angiitis" OR TI "Eosinophilic Granulomatous Vasculit*" OR TI "erythematodes visceralis" OR TI "Essential Polyarterit*" OR TI "generalised scleroderma" OR TI "generalized scleroderma" OR TI "Giant Cell Aortic Arteritis" OR TI "Giant Cell Aortiti*" OR TI "giant cell arteriitis" OR TI "Giant Cell Arteriti*" OR TI "gougerot houwer sjoegren syndrome" OR TI "gougerot mulock houwer sjoegren syndrome" OR TI "Gougerot Sjoegren syndrome" OR TI "Gougerot-Sjogren syndrome" OR TI "granulomatosis and polyangiitis" OR TI "granulomatosis and polyangitis" OR TI "Granulomatosis with Polyangiit*" OR TI "granulomatosis with polyangitis" OR TI "granulomatous allergic angitis" OR TI "granulomatous polyangiitis" OR TI "granulomatous polyangitis" OR TI "Horton arteritis" OR TI "Horton Disease" OR TI "Horton's arteritis" OR TI "Hortons Disease" OR TI "Horton's Disease" OR TI "Hughes Syndrome*" OR TI "innominate arterial ligation" OR TI "innominate artery ligation" OR TI "innominate artery occlusion" OR TI "jungling syndrome" OR TI "kussmaul maier disease" OR TI "kussmaul syndrome" OR TI "Libman Sacks Disease*" OR TI "Libman-Sacks Disease*" OR TI "lupovisceritis" OR TI "lymphogranuloma benignum" OR TI "malignant dermatovisceritism" OR TI "martorell syndrome" OR TI "Microscopic Polyangiitides" OR TI "microscopic polyarteritis" OR TI "mikulicz radecki syndrome" OR TI "mixed cryoglobulinemia" OR TI "morbus Wegener" OR TI "mucoserous dyssecretosis" OR TI "mukilicz radecki syndrome" OR TI "multisystem disease" OR TI "multisystem diseases" OR TI "multisystem disorder" OR TI "multisystem disorders" OR TI "multisystem dysfunction*" OR TI "necrotising respiratory granulomatosis" OR TI "Necrotizing Arterit*" OR TI "necrotizing respiratory granulomatosis" OR TI "nodular periarteritis" OR TI "nodular polyarteritis" OR TI "oculobuccopharyngeal dryness" OR TI "Old Silk Route Disease*" OR TI "panarteriitis nodosa" OR TI "panarteritis nodosa" OR TI "periarterial fibrosis" OR TI "periarteriitis nodosa" OR TI "Periarteritis Nodosa" OR TI "pneumogenic granulomatosis" OR TI "poliarteritis nodosa" OR TI "polyarteriitis nodosa" OR TI "progressive scleroderma" OR TI "progressive sclerodermia" OR TI "Pulseless Disease*" OR TI "reversed coarctation" OR TI "rheumatic sialosis" OR TI "sarcoid" OR TI "sarcoidoses" OR TI "Schaumann Disease*" OR TI "Schaumann Syndrome*" OR TI "Schaumann's Syndrome*" OR TI "sicca syndrome" OR TI "sjoegren disease" OR TI "sjogren disease" OR TI "sjogren syndrome" OR TI "sjogren's syndrome" OR TI "lupus" OR TI "Strauss Churg syndrome" OR TI "systemic disease" OR TI "systemic diseases" OR TI "systemic disorder*" OR TI "systemic dysfunction*" OR TI "systemic progressive sclerosis" OR TI "Systemic Scleroderma" OR TI "Systemic Sclerosis" OR TI "takayasu arteriopathy" OR TI "Takayasu Arteritis" OR TI "Takayasu Disease*" OR TI "takayasu ohnishi syndrome" OR TI "Takayasu Syndrome*" OR TI "Takayasus Arteritis" OR TI

"Takayasu's Arteritis" OR TI "Temporal Arterit*" OR TI "Triple Symptom Complex" OR TI "Triple Symptom Complices" OR TI "Triple-Symptom Complex" OR TI "Wegener disease" OR TI "Wegener granuloma" OR TI "Wegener Granulomatosis" OR TI "Wegener Klinger Churg syndrome" OR TI "Wegener Klinger granulomatosis" OR TI "Wegener syndrome" OR TI "Wegener's disease" OR TI "Wegener's GPA" OR TI "Wegener's granuloma" OR TI "Wegener's Granulomatosis" OR TI "Wegner granulomatosis" OR TI "Young Female Arterit*" OR TI "Anti GBM Disease*" OR TI "Anti Glomerular Basement Membrane Disease" OR TI "Anti-GBM Disease*" OR TI "bladder incontinence" OR TI "chronic disease kidney function" OR TI "Chronic Kidney Failure*" OR TI "chronic nephropathy" OR TI "Chronic Renal Failure*" OR TI "cystic kidney" OR TI "End Stage Kidney Disease*" OR TI "End Stage Renal Disease*" OR TI "End Stage Renal Failure*" OR TI "End-Stage Kidney Disease*" OR TI "End-Stage Renal Disease*" OR TI "End-Stage Renal Failure*" OR TI "ESRD" OR TI "familial nephrolithiasis" OR TI "goodpasture disease" OR TI "goodpasture renopulmonary syndrome" OR TI "Goodpasture Syndrome*" OR TI "Goodpastures Syndrome*" OR TI "Goodpasture's Syndrome*" OR TI "incontinentia urinae" OR TI "involuntary urinary loss" OR TI "involuntary urination" OR TI "involuntary urine loss" OR TI "Kidney Calcul*" OR TI "kidney calix stone" OR TI "kidney calyx stone" OR TI "kidney chronic failure" OR TI "kidney cystic disease" OR TI "kidney disease" OR TI "kidney diseases" OR TI "kidney disorder" OR TI "kidney disorders" OR TI "kidney failure" OR TI "kidney failures" OR TI "kidney insufficien*" OR TI "kidney lithiasis" OR TI "kidney multicystic aplasia" OR TI "kidney multicystic disease" OR TI "kidney pelvis stone" OR TI "kidney polycystosis" OR TI "Kidney Stone*" OR TI "leakage of urine" OR TI "Lung Purpura with Nephritis" OR TI "male genital disorder" OR TI "male genital disorders" OR TI "male infertility" OR TI "Nephrolith" OR TI "pneumorenal syndrome" OR TI "Polycystic Kidney" OR TI "Polycystic Kidneys" OR TI "prostate adenoma" OR TI "Renal Calcul*" OR TI "renal cystic disease" OR TI "renal disease" OR TI "renal diseases" OR TI "renal disorder" OR TI "renal disorders" OR TI "renal failure" OR TI "renal failures" OR TI "renal insufficien*" OR TI "renal pelvis stone" OR TI "renal polycystic disease" OR TI "renal stone" OR TI "renolithiasis" OR TI "unwanted urine loss" OR TI "urinary incontinence" OR TI "urinary leakage" OR TI "urine incontinence" OR TI "urine leakage" OR TI "urine wetting" OR TI "urologic disease*" OR TI "urologic disorder*" OR TI "urological disease*" OR TI "urological disorder*" OR TI "Age Related Macular Degeneration" OR TI "Age-Related Macular Degeneration*" OR TI "atrophia maculae luteae" OR TI "bilateral macular degeneration" OR TI "Cataract*" OR TI "chronic uveitis" OR TI "corpus vitreum detachment" OR TI "Day Blindness" OR TI "degeneratio maculae luteae retinae" OR TI "detachment corporis vitrei" OR TI "detachment vitreous" OR TI "disciform macular degeneration" OR TI "Episclerit*" OR TI "eye disease" OR TI "eye diseases" OR TI "eye disorder" OR TI "eye disorders" OR TI "eye dysfunction" OR TI "eye dysfunctioning" OR TI "eye dysfunctions" OR TI "Hemeralopia*" OR TI "heredomacular degeneration" OR TI "immunogenic uveitis" OR TI "junius kuhnt disease" OR TI "lens clouding" OR TI "Lens Opacit*" OR TI "Macropsia*" OR TI "macula atrophy" OR TI "macula bilateral degeneration" OR TI "macula degeneration" OR TI "macula lutea atrophy" OR TI "macula lutea degeneration" OR TI "macula lutea disciform degeneration" OR TI "macula lutea retina atrophy" OR TI "macula lutea retina degeneration" OR TI "macula retina atrophy" OR TI "macula retina degeneration" OR TI "macular atrophy" OR TI "Macular Degenerations" OR TI "macular disciform degeneration" OR TI "Macular Dystroph*" OR TI "Maculopath*" OR TI "Metamorphopsia*" OR TI "Micropsia*" OR TI "Myopia*" OR TI "Nearsightedness*" OR TI "Necrotizing Sclerit*" OR TI "ocular disease" OR TI "ocular diseases" OR TI "ocular disorder" OR TI "ocular disorders" OR TI "ocular dysfunction*" OR TI "ophthalmic disease" OR TI "ophthalmic diseases" OR TI "ophthalmic disorder" OR TI "ophthalmic disorders" OR TI "ophthalmic dysfunction*" OR TI "ophthalmologic disease" OR TI "ophthalmologic diseases" OR TI "ophthalmologic disorder" OR TI "ophthalmologic disorders" OR TI "ophthalmologic dysfunction*" OR TI "panuveitis" OR TI "posterior capsule opacification" OR TI "posterior uveitis" OR TI "Presbyopias" OR TI "Pseudoaphakia*" OR TI "retina macula disciform

degeneration" OR TI "retinal diseases" OR TI "Scleritides" OR TI "secondary scleritis" OR TI "suppurative uveitis" OR TI "Uveitides" OR TI "Vision Disabilit*" OR TI "vision disorder" OR TI "vision disorders" OR TI "vision disturbance" OR TI "visual disorder" OR TI "visual disorders" OR TI "visual disturbance" OR TI "Visual Impairment*" OR TI "vitreous detachment" OR TI "sarcoidosis" OR TI "neoplasia" OR TI "lymphoma*" OR TI "hypertension" OR TI "infarction*" OR TI "glaucom*" OR TI "cancer*" OR TI "carcinoma*" OR TI "neoplasm*" OR TI "tumor*" OR TI "tumour*" OR TI "Horton syndrome" OR TI "Horton's syndrome" OR TI "Gastro-esophageal reflux" OR TI "gastroesophageal reflux" OR TI "GORD" OR AB "Basal Cell Epithelioma" OR AB "Basal Cell Epitheliomas" OR AB "Brain Metastase" OR AB "Brain Metastases" OR AB "cerebroma" OR AB "Colon Adenocarcinoma" OR AB "Colon Adenocarcinomas" OR AB "dermatoma" OR AB "encephalophyma" OR AB "hypophysoma" OR AB "kidney mass" OR AB "kidney neoplastic mass" OR AB "malignant melanomatosis" OR AB "melanocarcinoma" OR AB "Melanoma" OR AB "melanomalignoma" OR AB "Melanomas" OR AB "naevocarcinoma" OR AB "neoplastic lung" OR AB "neoplastic mammary gland" OR AB "nephroma" OR AB "nevocarcinoma" OR AB "Pituitary Adenoma" OR AB "Pituitary Adenomas" OR AB "rectal mass" OR AB "rectum mass" OR AB "renal mass" OR AB "renal neoplastic mass" OR AB "reninoma" OR AB "Rodent Ulcer" OR AB "Rodent Ulcers" OR AB "squamous cell epithelioma" OR AB "squamous epithelioma" OR AB "Thyroid Adenoma" OR AB "Thyroid Adenomas" OR AB "angina" OR AB "anginal attack" OR AB "angiocardiopathy" OR AB "angiocardiovascular disease" OR AB "angiocardiovascular diseases" OR AB "Angor Pectori" OR AB "Aortic Valve Diseases" OR AB "Aortic Valve Disorder" OR AB "Aortic Valve Disorders" OR AB "Arrhythmia" OR AB "Arrythmia" OR AB "Artificial Cardiac Pacemaker" OR AB "Artificial Cardiac Pacemakers" OR AB "Artificial Pacemaker" OR AB "Artificial Pacemakers" OR AB "Atrial Fibrillation" OR AB "Atrial Fibrillations" OR AB "Auricular Fibrillation" OR AB "Auricular Fibrillations" OR AB "backward failure heart" OR AB "backward failure, heart" OR AB "Cardiac Arrest" OR AB "Cardiac Arrhythmias" OR AB "cardiac backward failure" OR AB "cardiac conduction system disease" OR AB "cardiac decompensation" OR AB "Cardiac Disorders" OR AB "cardiac disrhythmia" OR AB "Cardiac Dysrhythmia" OR AB "cardiac failure" OR AB "cardiac failures" OR AB "cardiac incompetence" OR AB "cardiac incompetences" OR AB "cardiac infarct" OR AB "cardiac insufficien*" OR AB "cardiac stand still" OR AB "cardiac valve defect" OR AB "cardiac valve disease" OR AB "cardial decompensation" OR AB "cardial infarct" OR AB "cardial insufficiency" OR AB "cardial valve disease" OR AB "cardiovascular complication*" OR AB "cardiovascular decompensation" OR AB "cardiovascular disease" OR AB "cardiovascular diseases" OR AB "cardiovascular disorder" OR AB "cardiovascular disorders" OR AB "cardiovascular disturbance" OR AB "cardiovascular disturbances" OR AB "cardiovascular disturbancescardiovascular lesion*" OR AB "cardiovascular failure" OR AB "cardiovascular failures" OR AB "cardiovascular incompetence" OR AB "cardiovascular incompetences" OR AB "cardiovascular insufficien*" OR AB "cardiovascular lesion*" OR AB "cardiovascular syndrome" OR AB "cardiovascular syndromes" OR AB "cardiovascular vegetative disorder" OR AB "cardiovascular vegetative disorders" OR AB "cholesteremia" OR AB "cholesterinemia" OR AB "cholesterolemia" OR AB "Circulatory Disorders" OR AB "complication cardiovascular" OR AB "conduction defect" OR AB "conduction defects" OR AB "conduction disease" OR AB "conduction diseases" OR AB "conduction disorder" OR AB "conduction disorders" OR AB "conduction disturbance" OR AB "conduction disturbances" OR AB "Coronary Disorders" OR AB "decompensatio cordis" OR AB "decompensation, heart" OR AB "ectopic heart rhythm" OR AB "ectopic rhythm" OR AB "Elevated Cholesterol" OR AB "Elevated Cholesterols" OR AB "heart aberrant conduction" OR AB "heart arrhytmia" OR AB "Heart Attack" OR AB "Heart Attacks" OR AB "heart backward failure" OR AB "heart decompensation" OR AB "heart disease" OR AB "heart diseases" OR AB "heart disorder" OR AB "heart disorders" OR AB "heart dysrhythmia" OR AB "heart ectopic beat" OR AB "heart ectopic ventricle contraction" OR AB "heart failure" OR AB "heart failures" OR AB "heart incompetence" OR AB "heart infarct" OR AB "heart insufficien*" OR AB "heart rhythm

disorder" OR AB "heart valve abnormalities" OR AB "heart valve abnormality" OR AB "heart valve defect" OR AB "heart valve degeneration" OR AB "Heart Valve Disease" OR AB "heart valve diseases" OR AB "heart valve lesion" OR AB "Heart Valvular Disease" OR AB "Heart Valvular Diseases" OR AB "High Blood Pressure" OR AB "High Blood Pressures" OR AB "High Cholesterol Level" OR AB "High Cholesterol Levels" OR AB "Hypercholesteremia" OR AB "Hypercholesteremias" OR AB "hypercholesterinaemia" OR AB "hypercholesterinemia" OR AB "hypercholesterolaemia" OR AB "Hypercholesterolemias" OR AB "hypertensive disease" OR AB "hypertensive effect" OR AB "hypertensive pulmonary vascular disease" OR AB "hypertensive response" OR AB "insufficientia cardis" OR AB "insufficientio cardis" OR AB "lung embolism" OR AB "lung embolization" OR AB "lung embolus" OR AB "lung emboly" OR AB "lung microembolism" OR AB "lung microembolization" OR AB "lung microembolus" OR AB "lung thromboembolism" OR AB "major adverse cardiovascular event*" OR AB "Myocardial Failure" OR AB "Myocardial Infarct" OR AB "myocardial insufficiency" OR AB "Myocardial lnfarcts" OR AB "myocardium infarct" OR AB "Periphlebitides" OR AB "Periphlebitis" OR AB "perivenous infection" OR AB "Phlebitides" OR AB "phlebitis superficialis" OR AB "post phlebitis syndrome" OR AB "postphlebitic disease" OR AB "postphlebitis syndrome" OR AB "Pulmonary Embolism" OR AB "Pulmonary Embolisms" OR AB "pulmonary embolization" OR AB "pulmonary embolus" OR AB "pulmonary hypertensive diseases" OR AB "pulmonary hypertensive disorder" OR AB "pulmonary hypertensive disorders" OR AB "pulmonary microembolism" OR AB "pulmonary thromboembolic disease" OR AB "Pulmonary Thromboembolism" OR AB "Pulmonary Thromboembolisms" OR AB "Raynauds Disease" OR AB "Stenocardia" OR AB "Stenocardias" OR AB "superficial phlebitis" OR AB "valvulopathies" OR AB "valvulopathy" OR AB "Vascular Disorders" OR AB "Vasculitis" OR AB "vein inflammation" OR AB "venitis" OR AB "venous inflammation" OR AB "vitium cordis" OR AB "Chronic Infectious Mononucleosis-Like Syndromes" OR AB "chronic intractable pain" OR AB "Royal Free Diseases" OR AB "Systemic Exertion Intolerance Diseases" OR AB "Chronic Pain" OR AB "Chronic Pains" OR AB "Achalasia" OR AB "Achalasias" OR AB "alcohol liver injury" OR AB "alcoholic liver" OR AB "Atrophic Gastritides" OR AB "Atrophic Gastritis" OR AB "autoimmune gastritis" OR AB "bowel syndrome" OR AB "cardiac herniation" OR AB "cardioesophageal reflux" OR AB "cardiooesophageal reflux" OR AB "Cardiospasm" OR AB "Cardiospasms" OR AB "Celiac Disease" OR AB "celiac syndrome" OR AB "chronic inflammatory bowel diseases" OR AB "chronic ulceration colon" OR AB "cirrhosis" OR AB "cleron disease" OR AB "coeliac disease" OR AB "coeliac syndrome" OR AB "coeliaky" OR AB "Colitis Gravis" OR AB "colitis ulcerativa" OR AB "colitis ulcerosa" OR AB "colon disease" OR AB "colon diseases" OR AB "colon disorder" OR AB "colon disorders" OR AB "colon juvenile polyp" OR AB "colon polypoid lesion" OR AB "colon spasm" OR AB "chronic colon ulceration" OR AB "Colonic Polyp" OR AB "Colonic Polyps" OR AB "colonospasm" OR AB "Crohns Disease" OR AB "Crohn's Disease" OR AB "Crohn's Enteritis" OR AB "digestive disease" OR AB "digestive diseases" OR AB "digestive disorder" OR AB "digestive disorders" OR AB "digestive system disorders" OR AB "enteritis regionalis" OR AB "Esophageal Hernia*" OR AB "Esophageal Reflux" OR AB "esophageal regurgitation" OR AB "esophagogastric reflux" OR AB "esophagus hernia" OR AB "esophagus reflux" OR AB "functional colonic diseases" OR AB "Gastric Acid Reflux" OR AB "gastric atrophy" OR AB "gastric regurgitation" OR AB "Gastroduodenal Ulcer" OR AB "Gastroduodenal Ulcers" OR AB "gastroesophageal reflex" OR AB "gastroesophageal regurgitation" OR AB "gastroesophagus reflux" OR AB "gastrointestinal disease" OR AB "gastrointestinal diseases" OR AB "gastrointestinal disorder" OR AB "gastrointestinal disorders" OR AB "gastrointestinal dysfunction*" OR AB "gastrooesophageal reflex" OR AB "gastrooesophageal reflux" OR AB "Gastro-oesophageal Reflux" OR AB "gastrooesophageal regurgitation" OR AB "gee herter disease" OR AB "gee thaysen disease" OR AB "GERD" OR AB "Gluten Enteropathies" OR AB "Gluten Enteropathy" OR AB "gluten induced enteropathy" OR AB "gluten intolerance" OR AB "Gluten Sensitive Enteropathy" OR AB "Gluten-Sensitive Enteropathies" OR AB "Gluten-Sensitive

Enteropathy" OR AB "Granulomatous Colitis" OR AB "Granulomatous Enteritis" OR AB "hernia hiatus esophagi" OR AB "hiatal diaphragmatic hernia" OR AB "Hiatal Hernia*" OR AB "hiatus diaphragmatic hernia" OR AB "hiatus esophagi hernia" OR AB "Hiatus Hernia*" OR AB "hiatus oesophageal hernia" OR AB "hiatus oesophagus hernia" OR AB "huebner herter disease" OR AB "Idiopathic Proctocolitis" OR AB "Ileocolitis" OR AB "Inflammatory Bowel Disease" OR AB "intestinal infantilism" OR AB "Irritable Bowel Syndromes" OR AB "Irritable Colon" OR AB "Liver Fibrosis" OR AB "Marginal Ulcer" OR AB "Marginal Ulcers" OR AB "Megaesophagus" OR AB "morbus crohn" OR AB "mucomembraneous colitis" OR AB "mucomembranous colitis" OR AB "mucosal colitis" OR AB "Mucous Colitides" OR AB "Mucous Colitis" OR AB "oesophageal reflux" OR AB "oesophageal regurgitation" OR AB "oesophagogastric reflux" OR AB "oesophagus reflux" OR AB "Paraesophageal Hernia" OR AB "para-esophageal hernia" OR AB "Paraesophageal Hernias" OR AB "paraesophageal herniation" OR AB "para-esophageal herniation" OR AB "paraoesophageal hernia" OR AB "para-oesophageal hernia" OR AB "paraoesophageal herniation" OR AB "para-oesophageal herniation" OR AB "Peptic Ulcers" OR AB "Regional Enteritis" OR AB "regional enterocolitis" OR AB "Regional Ileitides" OR AB "Regional Ileitis" OR AB "regurgitation, gastroesophageal" OR AB "spastic colitis" OR AB "spastic colon" OR AB "Sprue" OR AB "stomach hernia" OR AB "Terminal Ileitis" OR AB "Ulcerative Colitis" OR AB "ulcerative colorectitis" OR AB "ulcerative procto colitis" OR AB "ulcerative proctocolitis" OR AB "ulcerous colitis" OR AB "unstable colon" OR AB "acromegalia" OR AB "Acromegalies" OR AB "acromegalism" OR AB "Acromegaly" OR AB "Addison Disease" OR AB "Addisons Disease" OR AB "Addison's Disease" OR AB "adipose tissue hyperplasia" OR AB "adipositas" OR AB "adiposity" OR AB "adrenal cortex hyperplasia" OR AB "adrenal cortical hyperplasia" OR AB "adrenal failure" OR AB "adrenal failures" OR AB "adrenal gland disorders" OR AB "adrenocortical hyperplasia" OR AB "adrenocorticohyperplasia" OR AB "akromegalia" OR AB "Aldosteronism" OR AB "antidiuretic hormone insufficiency" OR AB "arenocortical hyperplasia" OR AB "Basedow Disease" OR AB "basedow syndrome" OR AB "Basedows Disease" OR AB "Basedow's Disease" OR AB "chromaffin paraganglioma" OR AB "chronic autoimmune thyroiditis" OR AB "Chronic Lymphocytic Thyroiditides" OR AB "Chronic Lymphocytic Thyroiditis" OR AB "Conn Syndrome" OR AB "Conns Syndrome" OR AB "Conn's Syndrome" OR AB "corpulency" OR AB "Cushing syndrome" OR AB "Cushings syndrome" OR AB "Cushing's Syndrome" OR AB "diabetes" OR AB "diabetic" OR AB "endocrinal disease" OR AB "endocrinal diseases" OR AB "endocrinal disorder" OR AB "endocrinal disorders" OR AB "endocrinal disturbance" OR AB "endocrinal disturbances" OR AB "endocrinal dysfunction*" OR AB "endocrine disease" OR AB "endocrine diseases" OR AB "endocrine disorder" OR AB "Endocrine Disorders" OR AB "endocrine disturbance" OR AB "endocrine disturbances" OR AB "endocrine dysfunction*" OR AB "endocrine gland disease" OR AB "endocrine gland diseases" OR AB "endocrine gland disorder" OR AB "endocrine gland disorders" OR AB "endocrine gland dysfunction*" OR AB "endocrine syndrome" OR AB "endocrine syndromes" OR AB "endocrine system disease" OR AB "endocrine system diseases" OR AB "endocrine system disorder" OR AB "endocrine system disorders" OR AB "endocrine system dysfunction*" OR AB "endocrinological disease" OR AB "endocrinological diseases" OR AB "endocrinological disorder" OR AB "endocrinological disorders" OR AB "endocrinological dysfunction*" OR AB "excess body weight" OR AB "Exophthalmic Goiter" OR AB "Exophthalmic Goiters" OR AB "exophthalmic goitre" OR AB "exophthalmic hyperthyroidism" OR AB "Extra Adrenal Pheochromocytoma" OR AB "Extra-Adrenal Pheochromocytoma" OR AB "familial hypoadrenocorticism" OR AB "fat overload syndrome" OR AB "feline hyperthyroidism" OR AB "Graves Disease" OR AB "Graves' Disease" OR AB "Graves hyperthyroidism" OR AB "graves syndrome" OR AB "Graves's disease" OR AB "Hashimoto Disease" OR AB "hashimoto goiter" OR AB "Hashimoto Struma" OR AB "Hashimoto Syndrome" OR AB "Hashimoto Thyroiditides" OR AB "Hashimoto Thyroiditis" OR AB "hashimoto thyroidosis" OR AB "Hashimotos Disease" OR AB "Hashimoto's Disease" OR AB "Hashimoto's Struma" OR AB "Hashimotos Syndrome" OR AB

"Hashimoto's Syndrome" OR AB "Hashimoto's Syndromes" OR AB "Hashimotos thyroiditis" OR AB "Hashimoto's thyroiditis" OR AB "hormonal disease" OR AB "hormonal diseases" OR AB "hormonal disorder" OR AB "hormonal disorders" OR AB "hormonal dysfunction*" OR AB "hormone imbalance" OR AB "hyperaldosterone" OR AB "Hypercortisolism" OR AB "hypermineralocorticidism" OR AB "hypermineralocorticism" OR AB "Hyperprolactinaemia*" OR AB "Hyperprolactinemia" OR AB "Hyperprolactinemias" OR AB "hyperthyreoidism" OR AB "hyperthyreosis" OR AB "Hyperthyroid" OR AB "hyperthyroidea" OR AB "hyperthyroidosis" OR AB "Hyperthyroids" OR AB "hypogonadism" OR AB "hypothyreoidism" OR AB "hypothyreosis" OR AB "hypothyroidea" OR AB "hypothyroidism" OR AB "Hypothyroidisms" OR AB "hypothyroidosis" OR AB "hypothyrosis" OR AB "Inappropriate GH Secretion Syndrome" OR AB "Inappropriate GH Secretion Syndromes" OR AB "Inappropriate Growth Hormone Secretion Syndrome" OR AB "Inappropriate Growth Hormone Secretion Syndromes" OR AB "Inappropriate Prolactin Secretion" OR AB "Inappropriate Secretion Prolactin" OR AB "lymphadenoid goiter" OR AB "megalakria" OR AB "mineralcorticoid excess syndrome" OR AB "obesitas" OR AB "obesity" OR AB "overweight" OR AB "phaeochromoblastoma" OR AB "phaeochromocytoma" OR AB "pheochromoblastoma" OR AB "Pheochromocytomas" OR AB "pheochromocytomata" OR AB "pheochromocytomatosis" OR AB "pheochromocytosis" OR AB "polycystic ovary syndrome" OR AB "polycystic ovary syndromes" OR AB "Primary Adrenal Insufficiency" OR AB "Primary Adrenocortical Insufficiencies" OR AB "Primary Adrenocortical Insufficiency" OR AB "Primary Hyperaldosteronism" OR AB "Primary Hyperthyroidism" OR AB "Primary Hypoadrenalism" OR AB "Primary Hypoadrenalisms" OR AB "Prolactin Hypersecretion Syndrome" OR AB "Prolactin Hypersecretion Syndromes" OR AB "Somatotropin Hypersecretion Syndrome" OR AB "Somatotropin Hypersecretion Syndromes" OR AB "struma hashimoto" OR AB "thyroid deficiency" OR AB "thyroid gland failure" OR AB "thyroid gland hyperfunction" OR AB "thyroid hyperfunction" OR AB "thyroid insufficiency" OR AB "Thyroid Stimulating Hormone Deficiency" OR AB "thyroideal hyperfunction" OR AB "Thyroid-Stimulating Hormone Deficiencies" OR AB "Thyroid-Stimulating Hormone Deficiency" OR AB "TSH Deficiencies" OR AB "TSH Deficiency" OR AB "Vasopressin Deficiency" OR AB "angioleiomyoma" OR AB "angiomyoma" OR AB "elastomyofibroma" OR AB "fibromyoma" OR AB "fibromyomas" OR AB "hemangioleiomyoma" OR AB "hemangiomyoma" OR AB "Impotence" OR AB "leimyoma" OR AB "Leiomyomas" OR AB "leyomyoma" OR AB "myofibroma" OR AB "myofibromatosis" OR AB "vascular leiomyoma" OR AB "addison anaemia" OR AB "addison anemia" OR AB "addisonian anaemia" OR AB "addisonian anemia" OR AB "ahf deficiency" OR AB "ahg deficiency" OR AB "anaemia perniciosa" OR AB "anemia perniciosa" OR AB "B12 deficiency anaemia" OR AB "B12 deficiency anemia" OR AB "B12 deficient anaemia" OR AB "B12 deficient anemia" OR AB "B12 vitamin deficiency anemia" OR AB "biermer anaemia" OR AB "biermer anemia" OR AB "biermer disease" OR AB "blood disease" OR AB "blood diseases" OR AB "blood disorder" OR AB "blood disorders" OR AB "blood dysfunction*" OR AB "classic haemophilia" OR AB "cobalamin deficiency anaemia" OR AB "cobalamin deficiency anemia" OR AB "congenital antihaemophilic factor deficiency" OR AB "congenital antihaemophilic globulin deficiency" OR AB "congenital antihemophilic factor deficiency" OR AB "congenital antihemophilic globulin deficiency" OR AB "drepanocytemia" OR AB "drepanocytic anaemia" OR AB "drepanocytic anemia" OR AB "drepanocytosis" OR AB "haematologic disease" OR AB "haematologic diseases" OR AB "haematologic disorder" OR AB "haematologic disorders" OR AB "haematologic dysfunction*" OR AB "haematological disease" OR AB "haematological diseases" OR AB "haematological disorder" OR AB "haematological disorders" OR AB "haematological dysfunction*" OR AB "haemochromatosis" OR AB "haemoglobin SS" OR AB "haemolytic anaemia" OR AB "haemolytic disease" OR AB "haemolytic syndrome" OR AB "haemophilia a" OR AB "haemophilia vera" OR AB "Hb SS disease" OR AB "heart hemochromatosis" OR AB "hemachromatosis" OR AB "hematologic disease" OR AB "hematologic diseases" OR AB "hematologic disorder" OR AB "hematologic disorders" OR AB "hematologic dysfunction*" OR AB "hematopathy" OR AB "hemoglobin SS" OR AB "hemolytic anemia" OR AB

"hemolytic disease" OR AB "hemolytic syndrome" OR AB "hemopathie" OR AB "hemopathies" OR AB "hemopathy" OR AB "hemophylia type a" OR AB "hereditary iron overload" OR AB "idiopathic hemochromatosis" OR AB "intermittent porphyria" OR AB "iron overload disease" OR AB "iron overload disorder" OR AB "iron overload syndrome" OR AB "latent porphyria" OR AB "lymphatic disease" OR AB "lymphatic diseases" OR AB "lymphatic disorder" OR AB "lymphatic disorders" OR AB "lymphatic dysfunction*" OR AB "macrocytic hyperchromic anaemia" OR AB "macrocytic hyperchromic anemia" OR AB "meniscocytosis" OR AB "pernicious anaemia" OR AB "pernicious anemia" OR AB "porphyrias" OR AB "porphyric disease" OR AB "primary anaemia" OR AB "primary anemia" OR AB "primary congenital hemochromatosis" OR AB "recklinghausen applebaum disease" OR AB "sickle anaemia" OR AB "sickle anemia" OR AB "sickle cell anaemia" OR AB "siderochromatosis" OR AB "sulfhemoglobinem*" OR AB "sulphaemoglobinaem*" OR AB "systemic porphyria" OR AB "thrombocytopaenia purpura" OR AB "thrombocytopenia purpura" OR AB "thrombotic purpura" OR AB "true haemophilia" OR AB "vitamin b 12 deficiency anaemia" OR AB "vitamin b 12 deficiency anemia" OR AB "a2 myeloma" OR AB "Acquired Hemolytic Anemias" OR AB "Acquired Immune Deficiency Syndrome Virus" OR AB "acquired immunodeficiency" OR AB "Addison Anemias" OR AB "Addisons Anemia" OR AB "Addison's Anemias" OR AB "AIDS" OR AB "aleukaemia" OR AB "aleukemia" OR AB "Arthriti*" OR AB "arthrochondritis" OR AB "arthrosynovitis" OR AB "B. burgdorferi Infection*" OR AB "bone marrow dysplasia" OR AB "Bone Marrow Fibros*" OR AB "Borrelia burgdorferi Infection*" OR AB "Bronze Diabete" OR AB "Bronzed Cirrhoses" OR AB "Chronic Hepatit*" OR AB "chronic myeloleukaemia" OR AB "chronic myeloleukemia" OR AB "Discitides" OR AB "discitis" OR AB "disk space infection" OR AB "Diskiti*" OR AB "DLBCL" OR AB "down disease" OR AB "Downs syndrome" OR AB "Down's syndrome" OR AB "Dysmyelopoietic Syndrome*" OR AB "endocardial inflammation" OR AB "Endocarditi*" OR AB "endo-carditis" OR AB "endocardium inflammation" OR AB "Erythremia*" OR AB "Factor 8 Deficienc*" OR AB "Factor VIII Deficienc*" OR AB "Factor VIII defiency" OR AB "Familial Hemochromatoses" OR AB "Familial Hemochromatosis" OR AB "Haemochromato" OR AB "Haemochromatos" OR AB "Haemolytic Anaemias" OR AB "HbS Disease" OR AB "Hematopoetic Myelodysplasias" OR AB "hemoblastoma" OR AB "Hemochromato" OR AB "Hemochromatos" OR AB "Hemoglobin S Disease" OR AB "Hemoglobin S Diseases" OR AB "Hemophilia" OR AB "hemophilias" OR AB "high-risk MDS" OR AB "Hodgkin disease*" OR AB "Hodgkin Granuloma*" OR AB "hodgkin sclerosis" OR AB "hodgkin's disease" OR AB "Hodgkins Disease*" OR AB "Hodgkins Granuloma" OR AB "Hodgkin's Granuloma" OR AB "HTLV-III" OR AB "Human Immunodeficiency Virus*" OR AB "Human T Cell Lymphotropic Virus Type III" OR AB "Human T Lymphotropic Virus Type III" OR AB "Human T-Cell Lymphotropic Virus Type III" OR AB "Human T-Lymphotropic Virus Type III" OR AB "infection by B. burgdorferi" OR AB "infection by Borrelia burgdorferi" OR AB "intervertebral disc infection" OR AB "intervertebral disk infection" OR AB "intervertebral disk inflammation" OR AB "Iron Storage Disorder*" OR AB "joint inflammation" OR AB "Koch Disease*" OR AB "Kochs Disease*" OR AB "Koch's Disease*" OR AB "langdon down syndrome" OR AB "LAV-HTLV-III" OR AB "leucaemia" OR AB "leucemia" OR AB "Leucocythaemia*" OR AB "Leucocythemia*" OR AB "leukaemia" OR AB "Leukemia*" OR AB "Lyme borrelioses" OR AB "Lyme Borreliosis" OR AB "Lyme's borrelioses" OR AB "Lyme's borreliosis" OR AB "Lymes disease" OR AB "Lyme's disease" OR AB "Lymphadenopathy Associated Virus*" OR AB "Lymphadenopathy-Associated Virus*" OR AB "lymphogranuloma maligne" OR AB "lymphogranuloma malignum" OR AB "lymphogranulomatosis" OR AB "Malignant Granuloma*" OR AB "Malignant Lymphogranuloma" OR AB "Malignant Lymphogranulomas" OR AB "Microangiopathic Anemias" OR AB "Microangiopathic Hemolytic Anemias" OR AB "mongolian idiocy" OR AB "mongolism" OR AB "mongoloid idiocy" OR AB "mongoloidism" OR AB "morbus hodgkin" OR AB "myelodysplasia" OR AB "myelodysplastic disease" OR AB "myelodysplastic disorder" OR AB "Myelodysplastic Syndrome*" OR AB "Myelofibros*" OR AB "Myeloid Metaplasia*" OR AB "myeloplaxoma" OR AB "Myeloscleros*" OR AB "Nonleukemic

Myelos*" OR AB "oligoarthritis" OR AB "Osler Vaquez Disease*" OR AB "Osler-Vaquez Disease*" OR AB "Pernicious Anemias" OR AB "Pigmentary Cirrhos*" OR AB "Polyarthrit*" OR AB "Polycythemia Ruba Vera*" OR AB "Polycythemia Rubra Vera*" OR AB "Primary Hemochromatosis" OR AB "Primary Polycythemia*" OR AB "reed sternberg disease" OR AB "Sickle Cell Anemia" OR AB "Sickle Cell Anemias" OR AB "Sickle Cell Disease" OR AB "Sickle Cell Diseases" OR AB "sickle cell disorder" OR AB "Sickle Cell Disorders" OR AB "Sickling Disorder Due to Hemoglobin S" OR AB "Spondylodisciti*" OR AB "Spondylodiskiti*" OR AB "TB disease" OR AB "TB infection" OR AB "Thrombocytopenic Purpura*" OR AB "Thrombopenic Purpura*" OR AB "Thrombotic Microangiopath*" OR AB "translocation 15 21 22" OR AB "trisomy 21 syndrome" OR AB "Troisier Hanot Chauffard Syndrome" OR AB "Troisier-Hanot-Chauffard Syndrome*" OR AB "Tuberculos*" OR AB "tuberculous infection" OR AB "tuberculous lesion" OR AB "Von Recklenhausen Applebaum Disease" OR AB "Von Recklenhausen-Applebaum Diseases" OR AB "Acute Autoimmune Neuropathies" OR AB "Acute Autoimmune Neuropathy" OR AB "Acute Cerebrovascular Accident*" OR AB "acute cerebrovascular lesion" OR AB "acute febrile polyneuritis" OR AB "acute focal cerebral vasculopathy" OR AB "Acute Infectious Polyneuritis" OR AB "Acute Inflammatory Demyelinating Polyneuropath*" OR AB "Acute Inflammatory Demyelinating Polyradiculoneuropath*" OR AB "Acute Inflammatory Polyneuropath*" OR AB "Acute Inflammatory Polyradiculoneuropath*" OR AB "acute postinfective polyradiculoneuropathy" OR AB "Acute Stroke*" OR AB "Alzeimer" OR AB "Alzheimer" OR AB "Alzeimers disease" OR AB "Alzeimer's disease" OR AB "Alzheimer's Disease" OR AB "Alzheimers Disease*" OR AB "Alzheimer's Diseases" OR AB "Alzheimer-Type Dementia" OR AB "Anti-MuSK Myasthenia Gravis" OR AB "Apoplex*" OR AB "Arteriosclerotic Dementia*" OR AB "Aura" OR AB "Auras" OR AB "autoimmune myasthenia gravis" OR AB "Binswanger Disease*" OR AB "Binswanger Encephalopath*" OR AB "Binswangers Disease*" OR AB "Binswanger's Disease*" OR AB "Binswangers Encephalopath*" OR AB "Binswanger's Encephalopath*" OR AB "brain accident" OR AB "brain attack" OR AB "brain blood flow disturbance" OR AB "brain insult" OR AB "brain insultus" OR AB "Brain TIA" OR AB "Brain Vascular Accident*" OR AB "Cephalgia Syndrome" OR AB "Cephalgia Syndromes" OR AB "cerebral insult" OR AB "Cerebral Stroke*" OR AB "cerebral vascular accident" OR AB "cerebral vascular insufficiency" OR AB "cerebro vascular accident" OR AB "Cerebrovascular Accident" OR AB "Cerebrovascular Accidents" OR AB "cerebrovascular arrest" OR AB "cerebrovascular failure" OR AB "cerebrovascular injury" OR AB "cerebrovascular insufficiency" OR AB "cerebrovascular insult" OR AB "Cerebrovascular Stroke*" OR AB "cerebrum vascular accident" OR AB "Chronic Daily Headache" OR AB "Chronic Daily Headaches" OR AB "Chronic Headache" OR AB "Chronic Headaches" OR AB "Chronic Progressive Subcortical Encephalopath*" OR AB "comitial disease" OR AB "congenital atonic sclerotic muscular dystrophy" OR AB "congenital muscular dystrophy" OR AB "CVA" OR AB "CVAs" OR AB "diffuse cortical sclerosis" OR AB "Disseminated Sclerosis" OR AB "Epileps*" OR AB "epileptic" OR AB "Epileptiform Neuralgia*" OR AB "erb goldflam disease" OR AB "falling sickness" OR AB "Fisher syndrome" OR AB "Fothergill Disease" OR AB "Generalized Myasthenia Gravis" OR AB "Guillain Barre" OR AB "Guillain-Barré" OR AB "Guillain-Barre" OR AB "Guillain Barré Syndrome*" OR AB "Headache Disorder" OR AB "Headache Syndrome" OR AB "Headache Syndromes" OR AB "Idiopathic Parkinson Disease*" OR AB "idiopathic parkinsonism" OR AB "infectious neuronitis" OR AB "inflammatory acute polyradiculoneuropathy" OR AB "Inflammatory Polyneuropathy Acute" OR AB "insular sclerosis" OR AB "insultus cerebralis" OR AB "Intractable Headache" OR AB "Intractable Headaches" OR AB "ischaemic attack" OR AB "ischaemic cerebral attack" OR AB "ischaemic seizure" OR AB "ischemic attack" OR AB "ischemic cerebral attack" OR AB "ischemic seizure" OR AB "lacunar dementia" OR AB "Landry paralysis" OR AB "Landry syndrome" OR AB "Landry-Guillain-Barre Syndrome" OR AB "Lewy Body Parkinson Disease*" OR AB "maternal myasthenia gravis" OR AB "mini-stroke" OR AB "multiinfarct dementia" OR AB "multi-infarct dementia" OR AB "multiinfarction dementia" OR AB "multi-infarction dementia" OR AB "multiple sclerosis" OR AB "muscle dystrophia" OR AB "muscle

dystrophy" OR AB "Muscle Specific Receptor Tyrosine Kinase Myasthenia Gravis" OR AB "Muscle Specific Tyrosine Kinase Antibody Positive Myasthenia Gravis" OR AB "Muscle-Specific Receptor Tyrosine Kinase Myasthenia Gravis" OR AB "Muscle-Specific Tyrosine Kinase Antibody Positive Myasthenia Gravis" OR AB "muscular dystrophia" OR AB "muscular dystrophies" OR AB "MuSK MG" OR AB "MuSK Myasthenia Gravis" OR AB "myasthenia gravis pseudoparalitica" OR AB "myasthenia gravis pseudoparalytica" OR AB "myodystrophia" OR AB "myodystrophy" OR AB "neonatal myasthenia gravis" OR AB "nervous disease" OR AB "nervous diseases" OR AB "nervous disorder" OR AB "nervous disorders" OR AB "nervous dysfunction*" OR AB "nervous system disease" OR AB "nervous system diseases" OR AB "nervous system disorder" OR AB "nervous system disorders" OR AB "nervous system dysfunction*" OR AB "neurologic disease" OR AB "neurologic diseases" OR AB "neurologic disorder" OR AB "neurologic disorders" OR AB "neurologic dysfunction*" OR AB "neurological disease" OR AB "neurological diseases" OR AB "neurological disorder" OR AB "neurological disorders" OR AB "neurological dysfunction*" OR AB "neuromuscular disease" OR AB "neuromuscular diseases" OR AB "neuromuscular disorder" OR AB "neuromuscular disorders" OR AB "neuromuscular dysfunction*" OR AB "Ocular Myasthenia Gravis" OR AB "Paralysis Agitans" OR AB "Parkinson dementia complex" OR AB "Parkinsons disease" OR AB "Parkinson disease" OR AB "Parkinson's Disease*" OR AB "Presenile Dementia" OR AB "Primary Parkinsonism" OR AB "Primary Senile Degenerative Dementia" OR AB "sclerosis multiplex" OR AB "Seizure Disorder*" OR AB "Senile Dementia" OR AB "stroke" OR AB "Strokes" OR AB "Subcortical Arteriosclerotic Encephalopath*" OR AB "Subcortical Leukoencephalopathies" OR AB "Subcortical Leukoencephalopathy" OR AB "Tic Douloureux" OR AB "transient brain ischaemia" OR AB "transient brain ischemia" OR AB "Transient Brain Stem Ischemia*" OR AB "Transient Brainstem Ischemia*" OR AB "Transient Cerebral Ischemia*" OR AB "Transient Cerebral Ischaemia*" OR AB "transient ischaemic attack*" OR AB "transient ischaemic seizure*" OR AB "Transient Ischemic Attack*" OR AB "transient ischemic seizure*" OR AB "Trifacial Neuralgia*" OR AB "Trigeminal Neuralgia*" OR AB "Vascular Dementia*" OR AB "acute paranasal sinusitis" OR AB "airway disease" OR AB "airway diseases" OR AB "airway disorder*" OR AB "airway dysfunction*" OR AB "Asthma*" OR AB "Atrophic Rhinitides" OR AB "Atrophic Rhinitis" OR AB "Auditory Vertigo" OR AB "Auditory Vertigos" OR AB "Aural Vertigo" OR AB "benign paroxysmal postural vertigo" OR AB "benign postural paroxysmal vertigo" OR AB "bronchitis chronica" OR AB "cerebral vertigo" OR AB "Chronic Airflow Obstruction*" OR AB "chronic airway obstruction" OR AB "Chronic Bronchitis" OR AB "chronic bronchus infection" OR AB "chronic emphysema" OR AB "chronic obstructive bronchopulmonary disease" OR AB "Chronic Obstructive Lung Disease" OR AB "chronic obstructive lung disorder" OR AB "Chronic Obstructive Pulmonary Disease" OR AB "Chronic Obstructive Pulmonary Diseases" OR AB "chronic obstructive pulmonary disorder" OR AB "chronic pulmonary obstructive disease" OR AB "chronic pulmonary obstructive disorder" OR AB "COAD" OR AB "cochlea hydrops" OR AB "COPD" OR AB "Cystic Fibrosis of Pancreas" OR AB "diffuse parenchyma lung disease" OR AB "Diffuse Parenchymal Lung Disease" OR AB "Diffuse Parenchymal Lung Diseases" OR AB "diffuse parenchymal pulmonary disease" OR AB "diffuse parenchymal pulmonary disorder" OR AB "endolymphatic hydrops" OR AB "endolymphatic sac hydrops" OR AB "Ethmoid Sinusitides" OR AB "Ethmoid Sinusitis" OR AB "Ethmoidal Sinusitides" OR AB "Ethmoidal Sinusitis" OR AB "fibrocystic disease" OR AB "hydrops labyrinthi" OR AB "Hypersomnia with Periodic Respiration" OR AB "Interstitial Lung Disease" OR AB "Interstitial Lung Diseases" OR AB "interstitial lung disorder" OR AB "Interstitial Pneumonia" OR AB "Interstitial Pneumonias" OR AB "Interstitial Pneumonitides" OR AB "Interstitial Pneumonitis" OR AB "interstitial pneumopathy" OR AB "interstitial pulmonary disease" OR AB "interstitial pulmonary disorder" OR AB "labyrinth hydrops" OR AB "labyrinthal syndrome" OR AB "lung allergy" OR AB "lung chronic obstructive disease" OR AB "Meniere Disease" OR AB "Ménière Disease" OR AB "Ménière Diseases" OR AB "Meniere Syndrome" OR AB "Ménière Vertigo" OR AB "Menieres Disease" OR AB "Meniere's Disease" OR AB "Ménières Disease" OR AB

"Ménière's Disease" OR AB "Ménière's Diseases" OR AB "Menieres Syndrome" OR AB "Meniere's Syndrome" OR AB "Ménières Vertigo" OR AB "Ménière's Vertigo" OR AB "Ménière's Vertigos" OR AB "Mucoviscidosis" OR AB "mucoviscoidosis" OR AB "nasal sinusitis" OR AB "nocturnal apnea" OR AB "nocturnal apnoea" OR AB "obstructive chronic lung disease" OR AB "obstructive chronic pulmonary disease" OR AB "Otogenic Vertigo" OR AB "Otogenic Vertigos" OR AB "Otoscleroses" OR AB "otosclerosis surgery" OR AB "otosclerotic stapes" OR AB "otosphongiosis" OR AB "Otospongioses" OR AB "Otospongiosis" OR AB "Ozena" OR AB "Ozenas" OR AB "pancreas cystic disease" OR AB "pancreas cystic fibrosis" OR AB "Pancreas Fibrocystic Diseases" OR AB "pancreas fibrosis" OR AB "pancreatic cystic disease" OR AB "Pancreatic Cystic Fibrosis" OR AB "pancreatic fibrosis" OR AB "paroxysmal labyrinthine vertigo" OR AB "paroxysmal positional vertigo" OR AB "pneumatosis" OR AB "positional paroxysmal vertigo" OR AB "Pulmonary Cystic Fibrosis" OR AB "respiration disease" OR AB "respiration diseases" OR AB "respiration disorder" OR AB "respiration disorders" OR AB "respiration dysfunction*" OR AB "respiration tract disease" OR AB "respiration tract diseases" OR AB "respiration tract disorder" OR AB "respiration tract disorders" OR AB "respiration tract dysfunction*" OR AB "respiratory disease" OR AB "respiratory diseases" OR AB "respiratory disorder" OR AB "respiratory disorders" OR AB "respiratory illness*" OR AB "respiratory tract disease" OR AB "respiratory tract diseases" OR AB "respiratory tract disorder" OR AB "respiratory tract disorders" OR AB "respiratory tract dysfunction" OR AB "respiratory tract dysfunctioning" OR AB "respiratory tract dysfunctions" OR AB "rhinitis atrophica" OR AB "sinusitis nasalis" OR AB "Sleep Apnea" OR AB "Sleep Apneas" OR AB "sleep apnoea" OR AB "Sleep Disordered Breathing" OR AB "Sleep Hypopnea" OR AB "Sleep Hypopneas" OR AB "Sleep-Disordered Breathing" OR AB "vertiginous disease" OR AB "vertiginous disorder" OR AB "vertiginous syndrome" OR AB "vestibular vertigo" OR AB "Age Related Osteoporosis" OR AB "Age-Related Bone Loss" OR AB "Age-Related Bone Losses" OR AB "Age-Related Osteoporosis" OR AB "Akureyri disease" OR AB "Algodystrophic Syndrome" OR AB "Algodystrophies" OR AB "Algodystrophy" OR AB "alibert bazin disease" OR AB "arthropathic psoriasis" OR AB "Arthroses" OR AB "Arthrosis" OR AB "axial spondylarthritis" OR AB "Axial Spondyloarthritides" OR AB "Axial Spondyloarthritis" OR AB "AxSpA" OR AB "Back Ache" OR AB "Back Aches" OR AB "Back Pain*" OR AB "Backache" OR AB "Backaches" OR AB "backpain" OR AB "beauvais disease" OR AB "Bilateral Sciatica" OR AB "Bilateral Sciaticas" OR AB "Bone Paget Disease" OR AB "Bone Pagets Disease" OR AB "Calcium Pyrophosphate Deposition Disease" OR AB "Calcium Pyrophosphate Dihydrate Deposition" OR AB "Cervical Pain" OR AB "Cervical Pains" OR AB "Cervical Sympathetic Dystrophies" OR AB "Cervical Sympathetic Dystrophy" OR AB "Cervicalgia" OR AB "Cervicalgias" OR AB "Cervicodynia" OR AB "Cervicodynias" OR AB "chariot disease" OR AB "Chondrocalcinoses" OR AB "chronic articular rheumatism" OR AB "chronic fatigue" OR AB "Chronic Fatigue-Fibromyalgia Syndrome*" OR AB "Chronic Infectious Mononucleosis Like Syndrome" OR AB "Chronic Infectious Mononucleosis-Like Syndrome" OR AB "chronic rheumatism" OR AB "complex regional pain syndrome 1" OR AB "complex regional pain syndrome type 1" OR AB "CRPS 1" OR AB "CRPS I" OR AB "CRPS type 1" OR AB "CRPS Type I" OR AB "CRPS-I" OR AB "crystal arthropathies" OR AB "crystalline arthropathy" OR AB "degenerative joint disease" OR AB "Diffuse Myofascial Pain Syndrome" OR AB "disc hernia" OR AB "Disc Herniation" OR AB "Disc Herniations" OR AB "disc prolapse" OR AB "Disc Protrusion" OR AB "Disc Protrusions" OR AB "discal hernia" OR AB "discal herniation" OR AB "discus hernia" OR AB "disk hernia" OR AB "Disk Herniation" OR AB "Disk Herniations" OR AB "Disk Prolapse" OR AB "Disk Prolapses" OR AB "Disk Protrusion" OR AB "Disk Protrusions" OR AB "dorsalgia" OR AB "epicondylalgia" OR AB "epidemic neuromyasthenia" OR AB "fatigue syndrome" OR AB "fibro myalgia" OR AB "Fibromyalgia Fibromyositis Syndrome" OR AB "Fibromyalgia-Fibromyositis Syndrome" OR AB "Fibromyalgia-Fibromyositis Syndromes" OR AB "Fibromyalgias" OR AB "Fibromyositis Fibromyalgia Syndrome" OR AB "Fibromyositis-Fibromyalgia Syndrome" OR AB "Fibromyositis-Fibromyalgia Syndromes" OR AB "fibrositic nodule" OR AB "Fibrositides" OR AB

"Fibrositis" OR AB "hernia disci" OR AB "hernia nuclei pulposi" OR AB "Herniated Disc" OR AB "Herniated Discs" OR AB "Herniated Disk" OR AB "Herniated Disks" OR AB "herniated intervertebral disc" OR AB "herniated intervertebral disk" OR AB "herniated nucleus pulposus" OR AB "herniated vertebral disc" OR AB "herniated vertebral disk" OR AB "hypertrophic infiltrative tendinitis" OR AB "Iceland disease" OR AB "Intervertebral Disc Displacement" OR AB "Intervertebral Disc Displacements" OR AB "Intervertebral Disk Displacement" OR AB "Intervertebral Disk Displacements" OR AB "intervertebral disk perforation" OR AB "intervertebral disk rupture" OR AB "intervertebral prolapse" OR AB "Involutional Osteoporosis" OR AB "ischias" OR AB "ischiatic pain" OR AB "Lateral Epicondylitides" OR AB "Lateral Epicondylitis" OR AB "Lateral Humeral Epicondylitides" OR AB "Lateral Humeral Epicondylitis" OR AB "loin pain" OR AB "lowback pain" OR AB "Lumbago" OR AB "lumbal pain" OR AB "lumbal syndrome" OR AB "lumbalgesia" OR AB "lumbalgia" OR AB "lumbar pain" OR AB "lumbar spine syndrome" OR AB "lumbodynia" OR AB "lumbosacral pain" OR AB "lumbosacral root syndrome" OR AB "lumbosacroiliac strain" OR AB "Muscular Rheumatism" OR AB "Myalgic Encephalomyelitis" OR AB "Neck Ache" OR AB "Neck Aches" OR AB "Neck Pain*" OR AB "Neckache" OR AB "Neckaches" OR AB "neuralgic shoulder amyotrophy" OR AB "nodular tendinitis" OR AB "nucleus pulposus hernia" OR AB "Osseous Paget's Disease" OR AB "osteitis deformans" OR AB "Osteoarthritides" OR AB "osteo-arthritis" OR AB "Osteoarthroses" OR AB "Osteoarthrosis" OR AB "osteo-arthrosis" OR AB "Osteoporoses" OR AB "ostitis deformans" OR AB "Paget Disease of Bone" OR AB "Paget disease of the bone" OR AB "Pagets bone disease" OR AB "Paget's bone disease" OR AB "Pagets disease of bone" OR AB "Paget's Disease of Bone" OR AB "Paget's disease of the bone" OR AB "paralytic scoliosis" OR AB "Post Traumatic Osteoporosis" OR AB "posttraumatic dystrophy" OR AB "post-traumatic dystrophy" OR AB "Post-Traumatic Osteoporosis" OR AB "Postviral Fatigue Syndromes" OR AB "Primary Fibromyalgia" OR AB "primary osteoarthritis" OR AB "progressive scoliosis" OR AB "Prolapsed Disc" OR AB "Prolapsed Discs" OR AB "Prolapsed Disk" OR AB "Prolapsed Disks" OR AB "Protruded Disc" OR AB "Protruded Discs" OR AB "Protruded Disk" OR AB "Protruded Disks" OR AB "Pseudogout" OR AB "Psoriasis Arthropathica" OR AB "psoriasis pustulosa arthropathica" OR AB "Psoriatic Arthropathies" OR AB "Psoriatic Arthropathy" OR AB "psoriatic rheumatism" OR AB "Reflex Sympathetic Dystrophies" OR AB "Reflex Sympathetic Dystrophy" OR AB "rheumarthritis" OR AB "rheumatic disease" OR AB "rheumatic diseases" OR AB "rheumatoid disease" OR AB "rheumatoid diseases" OR AB "rheumatoid inflammation" OR AB "rheumatological disease" OR AB "rheumatological diseases" OR AB "rheumatological disorder" OR AB "rheumatological disorders" OR AB "Royal Free Disease" OR AB "Sciatic Neuralgia" OR AB "Sciatic Neuralgias" OR AB "sciatic pain" OR AB "Scolioses" OR AB "Secondary Fibromyalgia" OR AB "Senile Osteoporosis" OR AB "shoulder arm syndrome" OR AB "Shoulder Hand Syndrome" OR AB "Shoulder-Hand Syndrome" OR AB "Shoulder-Hand Syndromes" OR AB "Slipped Disc" OR AB "Slipped Discs" OR AB "Slipped Disk" OR AB "Slipped Disks" OR AB "slipped intervertebral disc" OR AB "slipped vertebral disc" OR AB "spinal disk disease" OR AB "Sudek Atrophy" OR AB "Sudek's Atrophies" OR AB "Sudeks Atrophy" OR AB "Sudek's Atrophy" OR AB "sympathetic dystrophy syndrome" OR AB "Sympathetic Reflex Dystrophia" OR AB "Sympathetic Reflex Dystrophias" OR AB "sympathetic reflex dystrophy" OR AB "Systemic Exertion Intolerance Disease" OR AB "tendinopathy" OR AB "tendinosis" OR AB "tendonitis" OR AB "tendonopathy" OR AB "Tennis Elbow" OR AB "Tennis Elbows" OR AB "tenonitis" OR AB "tenontitis" OR AB "tenositis" OR AB "Type I Complex Regional Pain Syndrome" OR AB "Vertebrogenic Pain Syndrome" OR AB "Vertebrogenic Pain Syndromes" OR AB "Yuppie flu" OR AB "Acne Inversa*" OR AB "acne juvenilis" OR AB "Acne Rosacea" OR AB "Chronic Bullous Disease of Childhood" OR AB "cutaneous disease" OR AB "cutaneous diseases" OR AB "cutaneus disorder" OR AB "cutaneus disorders" OR AB "dermal disease" OR AB "dermal diseases" OR AB "dermal disorder" OR AB "dermal disorders" OR AB "Drug induced Linear IgA Bullous Dermatos*" OR AB "Drug-induced Linear IgA Bullous Dermatos*" OR AB "Eczema*" OR AB "Erythematotelangiectatic Rosacea" OR AB

"Granulomatous Rosacea" OR AB "herpes" OR AB "hidradenitis suppurativa" OR AB "Hives" OR AB "juvenile acne" OR AB "Linear IgA Dermatos*" OR AB "Linear IgA IgG Bullous Dermatos*" OR AB "Linear IgA IgG Dermatos*" OR AB "Ocular Rosacea" OR AB "Palmoplantaris Pustulosis" OR AB "Papulopustular Rosacea" OR AB "Phymatous Rosacea" OR AB "Psoriases" OR AB "psoriasiform dermatitis" OR AB "psoriasiform dermatosis" OR AB "psoriasiform lesion" OR AB "psoriasiform rash" OR AB "psoriasiform skin rash" OR AB "psoriatic epidermis" OR AB "psoriatic skin" OR AB "Pustular Psoriasis of Palms and Soles" OR AB "Pustulosis of Palms and Soles" OR AB "Pustulosis Palmaris et Plantaris" OR AB "rhinophyma" OR AB "rozacea" OR AB "skin and connective tissue disease" OR AB "skin and connective tissue diseases" OR AB "skin and connective tissue disorder" OR AB "skin and connective tissue disorders" OR AB "skin disease" OR AB "skin diseases" OR AB "skin disorder" OR AB "skin disorders" OR AB "Suppurative Hidradeniti*" OR AB "urticary" OR AB "weal" OR AB "wheal" OR AB "whealing" OR AB "willan lepra" OR AB "active TB" OR AB "Adamantiades-Behcet Disease*" OR AB "Allergic Angiit*" OR AB "Allergic Granulomatos*" OR AB "Allergic Granulomatous and Angiitis" OR AB "Allergic Granulomatous Angiit*" OR AB "allergic granulomatous angitis" OR AB "anonymous artery occlusion" OR AB "Anti Phospholipid Antibody Syndrome*" OR AB "Anti Phospholipid Syndrome*" OR AB "Antiphospholipid Antibody Syndrome*" OR AB "Anti-Phospholipid Antibody Syndrome*" OR AB "antiphospholipid syndrome" OR AB "Anti-Phospholipid Syndrome*" OR AB "aorta arch syndrome" OR AB "aortic arch syndromes" OR AB "Aortitis Syndrome*" OR AB "APLA syndrome" OR AB "arteritis brachiocephalica" OR AB "arteritis nodosa" OR AB "autoimmune disease" OR AB "autoimmune diseases" OR AB "Behcet Disease*" OR AB "Behçet Disease*" OR AB "Behcet syndrome" OR AB "behcet ulcer" OR AB "Behcets disease" OR AB "Behcet's Disease*" OR AB "Behcets syndrome" OR AB "Behcet's Syndrome*" OR AB "besnier boeck syndrome" OR AB "Besnier-Boeck Disease*" OR AB "Besnier-Boeck-Schaumann Syndrome*" OR AB "Boeck Disease*" OR AB "Boecks Disease*" OR AB "Boeck's Disease*" OR AB "brachiocephalic arteritis" OR AB "brachiocephalic artery occlusion" OR AB "brachiocephalic ischaemia" OR AB "brachiocephalic ischemia" OR AB "brachiocephalic trunk occlusion" OR AB "brachiocephalic vascular occlusion" OR AB "Church Strauss syndrome" OR AB "Churg Strauss" OR AB "Churg-Strauss Syndrome" OR AB "Cranial Arterit*" OR AB "cryoglobulinaemia" OR AB "Cryoglobulinemias" OR AB "cryoimmunoglobulinaemia" OR AB "cryoimmunoglobulinemia" OR AB "Cutis Elastica" OR AB "dacryosialoadenopathia atrophicans" OR AB "EDS IV" OR AB "Ehlers Danlos" OR AB "Ehlers-Danlos Disease*" OR AB "Ehlers-Danlos syndrome" OR AB "eosinophilic GPA" OR AB "eosinophilic granulomatosis polyangiitis" OR AB "eosinophilic granulomatosis polyangitis" OR AB "eosinophilic granulomatous angiitis" OR AB "Eosinophilic Granulomatous Vasculit*" OR AB "erythematodes visceralis" OR AB "Essential Polyarterit*" OR AB "generalised scleroderma" OR AB "generalized scleroderma" OR AB "Giant Cell Aortic Arteritis" OR AB "Giant Cell Aortiti*" OR AB "giant cell arteriitis" OR AB "Giant Cell Arteriti*" OR AB "gougerot houwer sjoegren syndrome" OR AB "gougerot mulock houwer sjoegren syndrome" OR AB "Gougerot Sjoegren syndrome" OR AB "Gougerot-Sjogren syndrome" OR AB "granulomatosis and polyangiitis" OR AB "granulomatosis and polyangitis" OR AB "Granulomatosis with Polyangiit*" OR AB "granulomatosis with polyangitis" OR AB "granulomatous allergic angitis" OR AB "granulomatous polyangiitis" OR AB "granulomatous polyangitis" OR AB "Horton arteritis" OR AB "Horton Disease" OR AB "Horton's arteritis" OR AB "Hortons Disease" OR AB "Horton's Disease" OR AB "Hughes Syndrome*" OR AB "innominate arterial ligation" OR AB "innominate artery ligation" OR AB "innominate artery occlusion" OR AB "jungling syndrome" OR AB "kussmaul maier disease" OR AB "kussmaul syndrome" OR AB "Libman Sacks Disease*" OR AB "Libman-Sacks Disease*" OR AB "lupovisceritis" OR AB "lymphogranuloma benignum" OR AB "malignant dermatovisceritism" OR AB "martorell syndrome" OR AB "Microscopic Polyangiitides" OR AB "microscopic polyarteritis" OR AB "mikulicz radecki syndrome" OR AB "mixed cryoglobulinemia" OR AB "morbus Wegener" OR AB "mucoserous dyssecretosis" OR AB "mukilicz radecki syndrome" OR AB "multisystem disease" OR AB

"multisystem diseases" OR AB "multisystem disorder" OR AB "multisystem disorders" OR AB "multisystem dysfunction*" OR AB "necrotising respiratory granulomatosis" OR AB "Necrotizing Arterit*" OR AB "necrotizing respiratory granulomatosis" OR AB "nodular periarteritis" OR AB "nodular polyarteritis" OR AB "oculobuccopharyngeal dryness" OR AB "Old Silk Route Disease*" OR AB "panarteriitis nodosa" OR AB "panarteritis nodosa" OR AB "periarterial fibrosis" OR AB "periarteriitis nodosa" OR AB "Periarteritis Nodosa" OR AB "pneumogenic granulomatosis" OR AB "poliarteritis nodosa" OR AB "polyarteriitis nodosa" OR AB "progressive scleroderma" OR AB "progressive sclerodermia" OR AB "Pulseless Disease*" OR AB "reversed coarctation" OR AB "rheumatic sialosis" OR AB "sarcoid" OR AB "sarcoidoses" OR AB "Schaumann Disease*" OR AB "Schaumann Syndrome*" OR AB "Schaumann's Syndrome*" OR AB "sicca syndrome" OR AB "sjoegren disease" OR AB "sjogren disease" OR AB "sjogren syndrome" OR AB "sjogren's syndrome" OR AB "lupus" OR AB "Strauss Churg syndrome" OR AB "systemic disease" OR AB "systemic diseases" OR AB "systemic disorder*" OR AB "systemic dysfunction*" OR AB "systemic progressive sclerosis" OR AB "Systemic Scleroderma" OR AB "Systemic Sclerosis" OR AB "takayasu arteriopathy" OR AB "Takayasu Arteritis" OR AB "Takayasu Disease*" OR AB "takayasu ohnishi syndrome" OR AB "Takayasu Syndrome*" OR AB "Takayasus Arteritis" OR AB "Takayasu's Arteritis" OR AB "Temporal Arterit*" OR AB "Triple Symptom Complex" OR AB "Triple Symptom Complices" OR AB "Triple-Symptom Complex" OR AB "Wegener disease" OR AB "Wegener granuloma" OR AB "Wegener Granulomatosis" OR AB "Wegener Klinger Churg syndrome" OR AB "Wegener Klinger granulomatosis" OR AB "Wegener syndrome" OR AB "Wegener's disease" OR AB "Wegener's GPA" OR AB "Wegener's granuloma" OR AB "Wegener's Granulomatosis" OR AB "Wegner granulomatosis" OR AB "Young Female Arterit*" OR AB "Anti GBM Disease*" OR AB "Anti Glomerular Basement Membrane Disease" OR AB "Anti-GBM Disease*" OR AB "bladder incontinence" OR AB "chronic disease kidney function" OR AB "Chronic Kidney Failure*" OR AB "chronic nephropathy" OR AB "Chronic Renal Failure*" OR AB "cystic kidney" OR AB "End Stage Kidney Disease*" OR AB "End Stage Renal Disease*" OR AB "End Stage Renal Failure*" OR AB "End-Stage Kidney Disease*" OR AB "End-Stage Renal Disease*" OR AB "End-Stage Renal Failure*" OR AB "ESRD" OR AB "familial nephrolithiasis" OR AB "goodpasture disease" OR AB "goodpasture renopulmonary syndrome" OR AB "Goodpasture Syndrome*" OR AB "Goodpastures Syndrome*" OR AB "Goodpasture's Syndrome*" OR AB "incontinentia urinae" OR AB "involuntary urinary loss" OR AB "involuntary urination" OR AB "involuntary urine loss" OR AB "Kidney Calcul*" OR AB "kidney calix stone" OR AB "kidney calyx stone" OR AB "kidney chronic failure" OR AB "kidney cystic disease" OR AB "kidney disease" OR AB "kidney diseases" OR AB "kidney disorder" OR AB "kidney disorders" OR AB "kidney failure" OR AB "kidney failures" OR AB "kidney insufficien*" OR AB "kidney lithiasis" OR AB "kidney multicystic aplasia" OR AB "kidney multicystic disease" OR AB "kidney pelvis stone" OR AB "kidney polycystosis" OR AB "Kidney Stone*" OR AB "leakage of urine" OR AB "Lung Purpura with Nephritis" OR AB "male genital disorder" OR AB "male genital disorders" OR AB "male infertility" OR AB "Nephrolith" OR AB "pneumorenal syndrome" OR AB "Polycystic Kidney" OR AB "Polycystic Kidneys" OR AB "prostate adenoma" OR AB "Renal Calcul*" OR AB "renal cystic disease" OR AB "renal disease" OR AB "renal diseases" OR AB "renal disorder" OR AB "renal disorders" OR AB "renal failure" OR AB "renal failures" OR AB "renal insufficien*" OR AB "renal pelvis stone" OR AB "renal polycystic disease" OR AB "renal stone" OR AB "renolithiasis" OR AB "unwanted urine loss" OR AB "urinary incontinence" OR AB "urinary leakage" OR AB "urine incontinence" OR AB "urine leakage" OR AB "urine wetting" OR AB "urologic disease*" OR AB "urologic disorder*" OR AB "urological disease*" OR AB "urological disorder*" OR AB "Age Related Macular Degeneration" OR AB "Age-Related Macular Degeneration*" OR AB "atrophia maculae luteae" OR AB "bilateral macular degeneration" OR AB "Cataract*" OR AB "chronic uveitis" OR AB "corpus vitreum detachment" OR AB "Day Blindness" OR AB "degeneratio maculae luteae retinae" OR AB "detachment corporis vitrei" OR AB "detachment vitreous" OR AB

"disciform macular degeneration" OR AB "Episclerit*" OR AB "eye disease" OR AB "eye diseases" OR AB "eye disorder" OR AB "eye disorders" OR AB "eye dysfunction" OR AB "eye dysfunctioning" OR AB "eye dysfunctions" OR AB "Hemeralopia*" OR AB "heredomacular degeneration" OR AB "immunogenic uveitis" OR AB "junius kuhnt disease" OR AB "lens clouding" OR AB "Lens Opacit*" OR AB "Macropsia*" OR AB "macula atrophy" OR AB "macula bilateral degeneration" OR AB "macula degeneration" OR AB "macula lutea atrophy" OR AB "macula lutea degeneration" OR AB "macula lutea disciform degeneration" OR AB "macula lutea retina atrophy" OR AB "macula lutea retina degeneration" OR AB "macula retina atrophy" OR AB "macula retina degeneration" OR AB "macular atrophy" OR AB "Macular Degenerations" OR AB "macular disciform degeneration" OR AB "Macular Dystroph*" OR AB "Maculopath*" OR AB "Metamorphopsia*" OR AB "Micropsia*" OR AB "Myopia*" OR AB "Nearsightedness*" OR AB "Necrotizing Sclerit*" OR AB "ocular disease" OR AB "ocular diseases" OR AB "ocular disorder" OR AB "ocular disorders" OR AB "ocular dysfunction*" OR AB "ophthalmic disease" OR AB "ophthalmic diseases" OR AB "ophthalmic disorder" OR AB "ophthalmic disorders" OR AB "ophthalmic dysfunction*" OR AB "ophthalmologic disease" OR AB "ophthalmologic diseases" OR AB "ophthalmologic disorder" OR AB "ophthalmologic disorders" OR AB "ophthalmologic dysfunction*" OR AB "panuveitis" OR AB "posterior capsule opacification" OR AB "posterior uveitis" OR AB "Presbyopias" OR AB "Pseudoaphakia*" OR AB "retina macula disciform degeneration" OR AB "retinal diseases" OR AB "Scleritides" OR AB "secondary scleritis" OR AB "suppurative uveitis" OR AB "Uveitides" OR AB "Vision Disabilit*" OR AB "vision disorder" OR AB "vision disorders" OR AB "vision disturbance" OR AB "visual disorder" OR AB "visual disorders" OR AB "visual disturbance" OR AB "Visual Impairment*" OR AB "vitreous detachment" OR AB "sarcoidosis" OR AB "neoplasia" OR AB "lymphoma*" OR AB "hypertension" OR AB "infarction*" OR AB "glaucom*" OR AB "cancer*" OR AB "carcinoma*" OR AB "neoplasm*" OR AB "tumor*" OR AB "tumour*" OR AB "Horton syndrome" OR AB "Horton's syndrome" OR AB "Gastro-esophageal reflux" OR AB "gastroesophageal reflux" OR AB "GORD") AND (MH"Sexual Dysfunction, Male+" OR MH"Sexual Dysfunction, Female+" OR MH"Sexual Dysfunction, Psychological+" OR MH"Sexual Dysfunction (Saba CCC)+" OR MH"Sexual Dysfunction (NANDA)+" OR MH "Sexuality" OR MH"Sexual Arousal+" OR MH"Libido +" OR MH"Orgasm+" OR TI "coitus" OR TI "desire" OR TI "dyspareunia" OR TI "ego-dystonic homosexuality" OR TI "ejaculatio praecox" OR TI "ejaculatio praecoxs" OR TI "ejaculatio precox" OR TI "erectile dysfunction*" OR TI "erection" OR TI "frigidities" OR TI "frigidity" OR TI "genital disorder" OR TI "genital disorders" OR TI "go-dystonic homosexuality" OR TI "libido" OR TI "orgasm" OR TI "orgasms" OR TI "premature ejaculation" OR TI "premature ejaculations" OR TI "psychosexuality" OR TI "sex abnormalit*" OR TI "sex arousal" OR TI "sex disorder" OR TI "sex disorders" OR TI "sex drive" OR TI "sex dysfunction*" OR TI "sex insufficienc*" OR TI "sex problem" OR TI "sex problems" OR TI "vaginismus" OR TI "sexual" OR TI "psychosexual" OR TI "sexuality" OR AB "coitus" OR AB "desire" OR AB "dyspareunia" OR AB "ego-dystonic homosexuality" OR AB "ejaculatio praecox" OR AB "ejaculatio praecoxs" OR AB "ejaculatio precox" OR AB "erectile dysfunction*" OR AB "erection" OR AB "frigidities" OR AB "frigidity" OR AB "genital disorder" OR AB "genital disorders" OR AB "go-dystonic homosexuality" OR AB "libido" OR AB "orgasm" OR AB "orgasms" OR AB "premature ejaculation" OR AB "premature ejaculations" OR AB "psychosexuality" OR AB "sex abnormalit*" OR AB "sex arousal" OR AB "sex disorder" OR AB "sex disorders" OR AB "sex drive" OR AB "sex dysfunction*" OR AB "sex insufficienc*" OR AB "sex problem" OR AB "sex problems" OR AB "vaginismus" OR AB "sexual" OR AB "psychosexual" OR AB "sexuality") AND (MH"Adult+" OR TI "adult" OR TI "adults" OR TI "old people" OR TI "elderly" OR TI "frail" OR TI "frailness" OR TI "frailty" OR TI "old age" OR TI "old patients" OR TI "old person" OR TI "old persons" OR TI "older adult" OR TI "older adults" OR TI "older patient" OR TI "older patients" OR TI "older people" OR TI "older person" OR TI "older persons" OR TI "senior people" OR TI "senior person" OR TI "senior persons" OR TI "seniors" OR AB "adult" OR AB "adults" OR AB "old people" OR AB "elderly" OR AB "frail" OR AB

"frailness" OR AB "frailty" OR AB "old age" OR AB "old patients" OR AB "old person" OR AB "old persons" OR AB "older adult" OR AB "older adults" OR AB "older patient" OR AB "older patients" OR AB "older people" OR AB "older person" OR AB "older persons" OR AB "senior people" OR AB "senior person" OR AB "senior persons" OR AB "seniors") AND (PT "Systematic Review" OR TI "scoping review*" OR TI "systematic review*" OR TI "systematic literature review*" OR TI "systematic narrative review*" OR TI "systematic qualitative review*" OR TI "systematic evidence review*" OR TI "systematic quantitative review*" OR TI "systematic meta-review*" OR TI "systematic critical review*" OR TI "systematic mapping review*" OR TI "systematic electronic literature search*" OR TI "PRISMA" OR TI "systematic descriptive review*" OR TI "systematic analys*" OR TI "targeted literature review*" OR TI "meta-synthes*" OR TI "comprehensive review*" OR TI "mixed studies review*" OR TI "sistematic review*" OR TI "umbrella review*" OR TI "mini-review*" OR TI "rapid literature review*" OR TI "rapid review*" OR TI "integrative review*" OR TI "systematic and critical analysis review*" OR TI "systematically review evidence" OR TI "systematic search*" OR TI "systematic methodological review*" OR TI "metaanalys*" OR TI "meta-analytic review*" OR TI "meta-analys*" OR TI "cochrane review*" OR AB "scoping review*" OR AB "systematic review*" OR AB "systematic literature review*" OR AB "systematic narrative review*" OR AB "systematic qualitative review*" OR AB "systematic evidence review*" OR AB "systematic quantitative review*" OR AB "systematic meta-review*" OR AB "systematic critical review*" OR AB "systematic mapping review*" OR AB "systematic electronic literature search*" OR AB "PRISMA" OR AB "systematic descriptive review*" OR AB "systematic analys*" OR AB "targeted literature review*" OR AB "meta-synthes*" OR AB "comprehensive review*" OR AB "mixed studies review*" OR AB "sistematic review*" OR AB "umbrella review*" OR AB "mini-review*" OR AB "rapid literature review*" OR AB "rapid review*" OR AB "integrative review*" OR AB "systematic and critical analysis review*" OR AB "systematically review evidence" OR AB "systematic search*" OR AB "systematic methodological review*" OR AB "metaanalys*" OR AB "meta-analytic review*" OR AB "meta-analys*" OR AB "cochrane review*")
